# Cord blood epigenome-wide meta-analysis in six European-based child cohorts identifies signatures linked to rapid weight growth

**DOI:** 10.1186/s12916-022-02685-7

**Published:** 2023-01-11

**Authors:** Rossella Alfano, Daniela Zugna, Henrique Barros, Mariona Bustamante, Leda Chatzi, Akram Ghantous, Zdenko Herceg, Pekka Keski-Rahkonen, Theo M. de Kok, Tim S Nawrot, Caroline L Relton, Oliver Robinson, Theano Roumeliotaki, Augustin Scalbert, Martine Vrijheid, Paolo Vineis, Lorenzo Richiardi, Michelle Plusquin

**Affiliations:** 1 Medical Research Council Centre for Environment and Health, Department of Epidemiology and Biostatistics, The School of Public Health, Imperial College London, London, UK; 2 Centre for Environmental Sciences, Hasselt University, Agoralaan Building D, 3590 Diepenbeek, Belgium; 3 Department of Medical Sciences, University of Turin and CPO-Piemonte, Turin, Italy; 4 Institute of Public Health, University of Porto, Porto, Portugal; 5 ISGlobal, Institute of Global Health, Barcelona, Spain; 6 Universitat Pompeu Fabra (UPF), Barcelona, Spain; 7 CIBER Epidemiología y Salud Pública, Madrid, Spain; 8 Department of Preventive Medicine, University of Southern California, Los Angeles, USA; 9 International Agency for Research on Cancer (IARC), 150 Cours Albert Thomas, 69008 Lyon, France; 10 Department of Toxicogenomics, Maastricht University, Maastricht, The Netherlands; 11 Μedical Research Council Integrative Epidemiology Unit, University of Bristol, Bristol, UK; 12 Mohn Centre for Children’s Health and Well-being, The School of Public Health, Imperial College London, London, UK; 13 Department of Social Medicine, Faculty of Medicine, University of Crete, Heraklion, Greece

**Keywords:** Rapid weight growth, Weight gain, DNA methylation, Gestational age acceleration, Childhood overweight, *AURKC*, Gene expression

## Abstract

**Background:**

Rapid postnatal growth may result from exposure *in utero* or early life to adverse conditions and has been associated with diseases later in life and, in particular, with childhood obesity. DNA methylation, interfacing early-life exposures and subsequent diseases, is a possible mechanism underlying early-life programming.

**Methods:**

Here, a meta-analysis of Illumina HumanMethylation 450K/EPIC-array associations of cord blood DNA methylation at single CpG sites and CpG genomic regions with rapid weight growth at 1 year of age (defined with reference to WHO growth charts) was conducted in six European-based child cohorts (ALSPAC, ENVIR*ON*AGE, Generation XXI, INMA, Piccolipiù, and RHEA, *N* = 2003). The association of gestational age acceleration (calculated using the Bohlin epigenetic clock) with rapid weight growth was also explored via meta-analysis. Follow-up analyses of identified DNA methylation signals included prediction of rapid weight growth, mediation of the effect of conventional risk factors on rapid weight growth, integration with transcriptomics and metabolomics, association with overweight in childhood (between 4 and 8 years), and comparison with previous findings.

**Results:**

Forty-seven CpGs were associated with rapid weight growth at suggestive *p*-value <1e−05 and, among them, three CpGs (cg14459032, cg25953130 annotated to *ARID5B*, and cg00049440 annotated to *KLF9*) passed the genome-wide significance level (*p*-value <1.25e−07). Sixteen differentially methylated regions (DMRs) were identified as associated with rapid weight growth at false discovery rate (FDR)-adjusted/Siddak *p*-values < 0.01. Gestational age acceleration was associated with decreasing risk of rapid weight growth (*p*-value = 9.75e−04). Identified DNA methylation signals slightly increased the prediction of rapid weight growth in addition to conventional risk factors. Among the identified signals, three CpGs partially mediated the effect of gestational age on rapid weight growth. Both CpGs (*N*=3) and DMRs (*N*=3) were associated with differential expression of transcripts (*N*=10 and 7, respectively), including long non-coding RNAs. An *AURKC* DMR was associated with childhood overweight. We observed enrichment of CpGs previously reported associated with birthweight.

**Conclusions:**

Our findings provide evidence of the association between cord blood DNA methylation and rapid weight growth and suggest links with prenatal exposures and association with childhood obesity providing opportunities for early prevention.

**Supplementary Information:**

The online version contains supplementary material available at 10.1186/s12916-022-02685-7.

## Background

Childhood obesity was declared an epidemic by the World Health Organization (WHO) more than two decades ago [1]. Forty million children below 5 years of age were affected by overweight or obesity in 2016 [2]. The changes in eating behaviors, available food choices, and physical activity observed during the COVID-19 pandemic have further amplified this global health issue [3]. Being obese during childhood is associated with both short-term health consequences (psychosocial, including social stigma, depression, and anxiety [4], and physical, including asthma [5]) and long-term health consequences (including obesity, cardiovascular diseases, diabetes, and cancers in adulthood), which can lead to disability and premature death [6, 7]. There is increasing evidence that the path to childhood obesity is established early in life, and rapid weight growth (RWG) in infancy has emerged as a major early risk factor [8–10], consisting of an upward centile crossing in weight growth charts and defined as a change greater than 0.67 in weight standard deviation (SD) scores. *In utero* and early life are critical time windows due to developmental plasticity. During these periods, exposure to detrimental factors may induce long-lasting alterations increasing susceptibility to diseases later in life, as postulated by the developmental origin of health and disease (DOHaD) theory [11]. RWG represents an early phenotype occurring as a thrifty adaptive response compensating the effects of adverse exposures, which in the long term becomes detrimental [12].

Biological mechanisms underlying RWG are poorly understood. Epigenetics, the mitotically inheritable changes in gene function not explained by changes in the DNA sequence, is a possible mechanism through which *in utero* exposures influence health and disease later in life [13]. Epigenome-wide association studies (EWAS) in large population-based child cohorts coordinated by the Pregnancy And Childhood Epigenetics (PACE) consortium found that several DNA methylation marks (*N*=914) at birth are associated with birthweight [14], in contrast with only one association with weight in childhood [15]. Previous studies investigating cord blood DNA methylation and early infancy weight and weight growth were limited to candidate genes (*IGF2* [16], *TACSTD2* [17], *MEG3* [18]) and gestational age acceleration (representing the difference between epigenetic gestational age predicted by CpG targets and actual gestational age determined using the last menstruation and/or ultrasounds) [19], and one previous EWAS was conducted in a small case-control study (*N*=40 children with RWG versus 40 without) [20]. Furthermore, none investigated concomitant changes spanning entire genomic regions, known as differentially methylated regions (DMRs), nor investigated which DNA methylation marks predict RWG. A better understanding of biological mechanisms acting at birth and underlying RWG is critical to creating effective policy and developing workable early-life prevention programs.

Therefore, in this study, we conducted a meta-analysis of six European-based child cohort (*N*=2003) EWAS to test the association of cord blood DNA methylation with RWG at 1 year at single CpG sites and CpG genomic regions. Furthermore, we investigated the association of gestational age acceleration with rapid weight growth via a fixed-effect meta-analysis similar to the main analysis. Then, we tested if the DNA methylation marks identified as related to RWG (i) improved the prediction of RWG by assessment of the predictive performance of models incorporating identified DNA methylation levels and conventional risk factors, including maternal education level [21], age at delivery [22], smoking during the index pregnancy [23], pre-pregnancy body mass index (BMI) [24], parity [25], and child gestational age [26]; (ii) were mediators of the effect of conventional risk factors on RWG; (iii) were associated with transcriptome and the metabolome, to guide functional interpretation; (iv) were associated with childhood overweight phenotype (between 4 and 8 years); and (v) overlapped with the previous literature findings.

## Methods

### Study population

The study population includes six European ancestry children cohorts: (i) the Avon Longitudinal Study of Parents And Children (ALSPAC) [27], (ii) the ENVironmental Influences ON early AGEing (ENVIR*ON*AGE) study [28], (iii) the Generation XXI (GXXI) study [29], (iv) the INfancia y Medio Ambiente (INMA) cohort [30], (v) the Piccolipiù cohort [31], and (vi) the Rhea cohort [32, 33]. However, the EWAS analyses are performed within four studies: (i) the ALSPAC, (ii) the ENVIR*ON*AGE study, (iii) the GXXI study, and (iv) the EXPOsOMICS study [34], because the latter study is a combination of samples from four cohorts (ENVIR*ON*AGE, INMA, Piccolipiù, and RHEA). EXPOsOMICS samples have been analyzed at the same moment, in the same laboratory, randomized across the different arrays. The ENVIR*ON*AGE cohort is part of EXPOsOMICS and has additionally performed separate DNA methylation arrays; nevertheless, there are no samples in common between the two studies. Multiple births (non-singleton) were excluded from the analyses. Participants of the cohorts included in the main analyses had a combined sample size of 2003 children with completed information (Table 1).

**Table 1:** Characteristics of the study population included in the meta-analysis of EWAS of rapid weight growth

Study population	***N***	Girls	Rapid weight growth	Gestational age, weeks	Birthweight, grams	Caesarian section	Nulliparity	Maternal age at delivery, years	Low maternal education	Maternal smoking during pregnancy	Maternal pre-pregnancy BMI, kg/m^**2**^
**ALSPAC**	729	355 (48.70)	227 (31.14)	39.55 (1.52)	3486.35 (479.98)	71 (9.81)	344 (47.19)	29.72 (4.41)	358 (49.11)	281 (38.55)	22.83 (3.71)
**ENVIR*ON*AGE**	247	121 (48.99)	69 (27.94)	39.23 (1.40)	3446.60 (433.75)	13 (5.26)	134 (54.25)	30.40 (4.12)	14 (5.67)	26 (10.53)	23.90 (4.28)
**EXPOsOMICS**	362	186 (51.38)	102 (28.18)	39.20 (1.57)	3296.17 (450.11)	97 (26.87)	171 (47.24)	30.94 (4.56)	40 (11.05)	64 (17.68)	23.96 (4.64)
**GXXI**	665	356 (53.53)	279 (41.95)	38.87 (1.39)	3230.72 (447.88)	200 (30.08)	375 (56.39)	29.58 (5.22)	326 (49.02)	142 (21.35)	24.08 (4.41)
**Meta-analysis**	2003	1018 (50.82)	677 (33.80)	39.22 (1.50)	3362.21 (472.21)	381 (19.08)	1024 (51.12)	29.98 (4.71)	738 (36.84)	513 (25.61)	23.58 (4.23)

The table shows counts (and percentages) and means (and standard deviations) for categorical and continuous variables, respectively. *EWAS* epigenome-wide association study

Written informed consent was obtained from participating mothers/parents in all cohorts. Information on inclusion criteria and protocols are described in Additional file 1: Supplementary Methods [27–38] and fully detailed in the respective references [27–33].

### DNA methylation

Cord blood DNA methylation was measured using the Infinium HumanMethylation450 BeadChip in ARIES and EXPOsOMICS [39, 40] and using Infinium MethylationEPIC BeadChip in ENVIR*ON*AGE and GXXI studies. Each cohort independently performed quality control and normalization of DNA methylation data, as reported in Additional file 1: Supplementary Methods [27–38]. DNA methylation levels were trimmed using the Tukey method if the removal of outliers had not been performed by cohort-specific preprocessing, and were expressed as beta values. CpGs were annotated to the nearest gene by the annotation provided by Illumina.

#### Cell type estimation

Cell types were estimated through established de-convolution approaches using Gervin’s in for ARIES and GENXXI [41] studies and Bakulski’s method in ENVIR*ON*AGE and EXPOsOMICS studies [42].

#### DNA methylation gestational age estimation and gestational age acceleration

DNA methylation gestational age was estimated using the Bohlin [43] and Knigh’s [44] epigenetic clocks via the methylclock R package (version 1.0.0) [45]. We used Spearman’s correlation to select the clock that predicted best the chronological gestational age. Gestational age acceleration was calculated as the residuals of the regression of chronological gestational age on DNA methylation gestational age.

### Rapid weight growth

In all the cohorts, weight measurements were obtained from obstetric records at birth, while measurements at later times were measured by trained staff (in ALSPAC and GXXI) or measured by trained staff or self-reported from parents (in ENVIR*ON*AGE and EXPOsOMICS) as detailed in Additional file 1: Supplementary Methods [27–38]. A two-step prediction approach was used for calculating sex- and age-specific weight at exactly 1 year, using fractional polynomials of age by gender in each cohort, as previously described [46]. Weight gain (WG) at 1 year was calculated as the difference between sex- and age-adjusted WHO-SD scores of birthweight and predicted weight at 1 year. Children were classified as having RWG if WG was > 0.67 SD scores according to Ong et al. [47].

### Covariates

In all the models, the following covariates, identified as *a priori* confounders or potential predictors depending on the performed analysis, were considered: maternal tobacco smoke during pregnancy [23] (categorized as smoker or non-smoker), education level at delivery [21] (categorized as low, medium, and high education based on cohort-specific preferential classification), pre-pregnancy BMI [24] (in kg/m^2^), age at delivery [22] (in years), parity [25] (categorized as nulli- and multiparous), and child gestational age [26] (in weeks) based on last menstrual period or ultrasound, sex, and cohort membership (for EXPOsOMICS only). Bead array row and bisulfite conversion batch were considered as technical confounders. All the analyses were adjusted for cell types estimated as described before. Infants with low birthweight are more likely to experience RWG, but birthweight could be either a mediator or a confounder in the association between cord blood DNA methylation and RWG, as it is measured at the same time as DNA methylation. Hence, birthweight was not included as a confounder in the main analyses but only in sensitivity analyses, along with delivery mode [48] (categorized as natural delivery or cesarean section) which could also be a potential confounder. Additional covariates, which may also confound the association under study, were used to restrict analyses and included maternal gestational diabetes [49] (categorized as having or not gestational diabetes) and ethnicity [50] (categorized as white European or non-white European children). A detailed description of the covariates in each cohort is reported in Additional file 1: Supplementary Methods [27–38].

### Gene expression

Gene expression levels were measured in the 200 cord blood samples of the ENVIR*ON*AGE cohort participating in the EXPOsOMICS project [34]. Total RNA was extracted using the total RNA miRNeasy mini kit (Qiagen, Venlo, Netherlands) according to the manufacturer’s protocol, as detailed previously [39, 51]. In brief, samples were quality checked and further hybridized onto Agilent Whole Human Genome 8×60 K microarrays, and microarray signals were detected by an Agilent DNA G2505C Microarray Scanner. After preprocessing and quality control were performed using an in-house developed R pipeline, gene expression was log_2_ transformed and normalized by quantile normalization using arrayQC. Residuals from linear regression between transcripts and hybridization date and leucocyte were used instead of the actual measures of gene expression to account for technical noise for a final sample size left available for further analysis of 29,164 transcripts for 165 children.

### Metabolomics

Untargeted metabolomics was measured in 500 cord blood samples of EXPOsOMICS, as previously described [52, 53]. Briefly, a reversed-phase liquid chromatography-quadrupole time-of-flight mass spectrometry system was used in positive ion mode with 499 of the 500 EXPOsOMICS samples successfully analyzed. After data preprocessing, 4712 features for 499 samples were left available for the subsequent analysis. Data were log-transformed and missing values were imputed using the impute. QRILC function within the imputeLCMD R package.

### Childhood overweight

Weight and height measurements were available during childhood, between 4 and 8 years of age, in ALSPAC, GXXI, and EXPOsOMICS studies. BMI was determined as the ratio of weight (in kilograms) over squared height (in meters) self-reported by parents or measured by trained staff. When multiple BMI measurements were available, the closest measurement to 6 years of age was considered to assess childhood overweight. Childhood overweight (including obesity) was defined if the child’s sex- and age-adjusted WHO-SD BMI score was >2 in children below 5 years of age and >1 in children older than 5 years of age, according to the WHO cut-offs [54].

### Statistical analysis

**Fig. 1:**
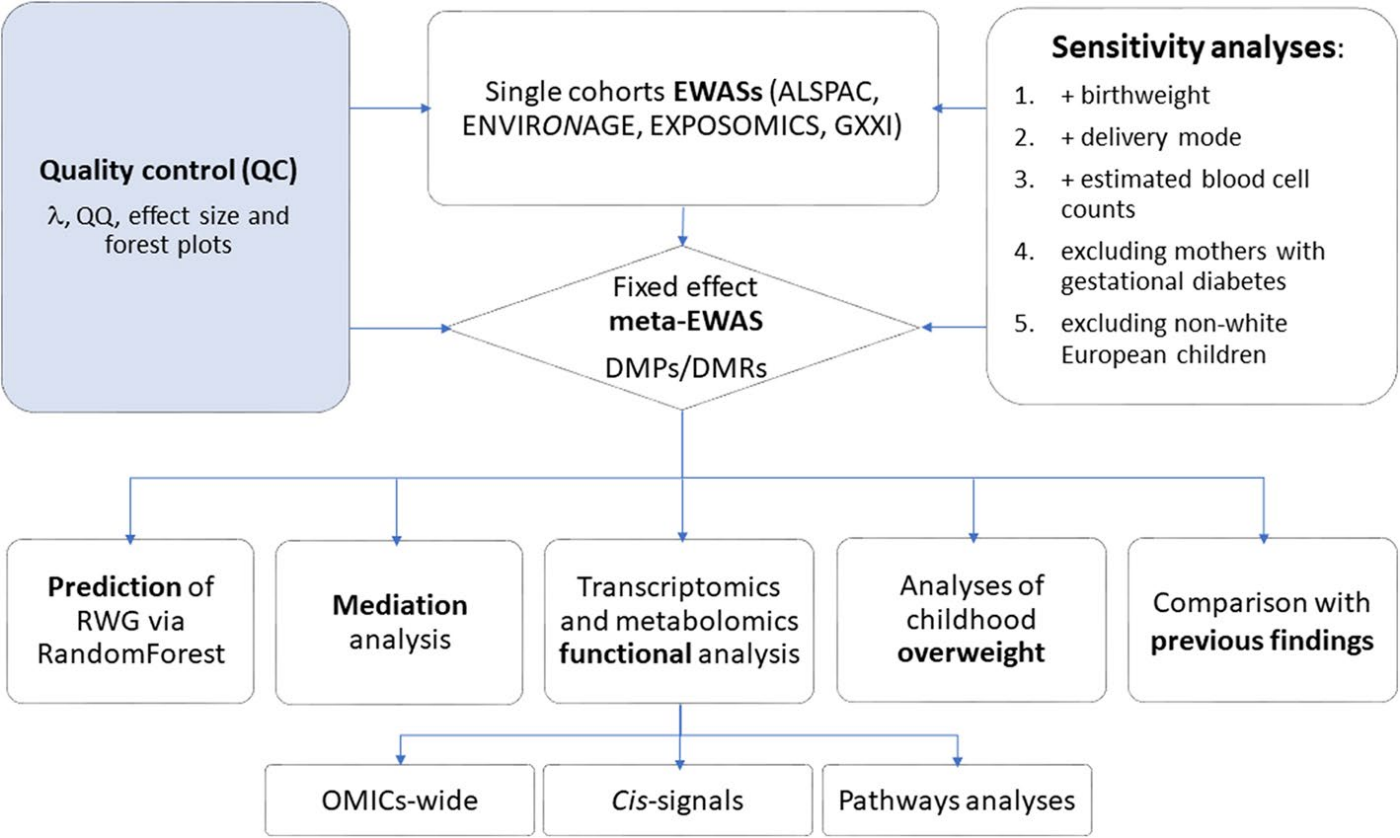
Flow chart depicts the study workflow

The study workflow is depicted in Fig. 1.

#### Epigenome-wide association studies of rapid weight growth

First, EWAS were conducted for the single cohorts separately, or in the case of the EXPOsOMICS cohorts together as the separate sample sizes were too small. Logistic mixed models with random effects on bead array row and bisulfite conversion batch were used to test the association between the cord blood CpGs (as explanatory variables) and RWG (as dependent variable) via the lme4 R package (version 1.1.26). Models were adjusted for maternal tobacco smoke during pregnancy, maternal education level at delivery, pre-pregnancy BMI, age at delivery, parity, child gestational age, sex, cohort membership (for EXPOsOMICS only), and cell proportions. Models were adjusted for child sex, although outcomes were based on sex- and age-adjusted SD weight scores, because sex is known to be a major source of variation in DNA methylation. Quality control of the results was performed by visual inspection of scatter plots of coefficients and standard errors, quantile-quantile plots (QQ plots) of *p*-values, and calculating inflation using the bacon method (*λ*_bacon_) via the R package bacon (version 1.14.0) [55].

#### Meta-analysis of EWAS of rapid weight growth

Then, single EWAS results were meta-analyzed via fixed-effects meta-analysis weighted by the inverse of the variance using the R package metafor (version 2.4.0). Meta-analysis was performed only on DNA methylation probes available in at least three EWAS (for a total of 398,036 CpG probes). Associations were deemed to be significant at the genome-wide level if *p*-values were below the Bonferroni-adjustment threshold (*p*_Bonferroni_) of 1.25e−07 (0.05/398,036). We also investigated CpGs at a less stringent *p*-value threshold (*p*_Suggestive_) of 1e−05. Results were presented as mean (and standard error) differences in log odds of RWG per 10% increase in methylation at each CpG. Quality control of the results was performed by visual inspection of coefficients, standard errors and *p*-values, and calculation of *λ*_bacon_. To assess the genome-wide robustness of the findings to inter-study heterogeneity, we used QQ plots of the *p*-values for heterogeneity (*I*^2^). Probes with *I*^2^ > 50% were excluded from further analyses. Results were inspected by forest plots, and meta-analyses leaving out one cohort at a time were performed.

DMRs were identified by using ENmix-comb-p (version 1.22.6) [56] and DMRcate (version 2.0.7) [57] R packages. We used the results from the meta-analysis of EWAS (estimated coefficients, *p*-values and *z*-values) as inputs for DMR analyses. The default setting values were used for DMRcate (e.g., minimum number of CpGs in a region = 2 and minimum length of nucleotides = 1000). To correct for multiple comparisons, we used FDR correction in DMRcate and 1-step Siddak correction in ENmix-comb-p. DMRs were considered to be statistically significant if both FDR-adjusted *p*-values in DMRcate and Siddak *p*-values in ENmix-comb-p were < 0.01.

Sensitivity analyses were also performed by repeating the meta-EWAS, adding birthweight and delivery mode to the confounders, and removing estimated cell counts from the confounders. Furthermore, we restricted meta-EWASs by excluding children born from mothers affected by gestational diabetes and of non-white European ethnicity.

#### Analysis of DNA methylation gestational age acceleration and rapid weight growth

In each study, we analyzed the association between gestational age acceleration and RWG using logistic models adjusted for maternal tobacco smoke during pregnancy, maternal education level at delivery, pre-pregnancy BMI, age at delivery, parity, child sex, cohort membership (for EXPOsOMICS only), and blood cell estimations. Then, similarly to the analyses described above, results were meta-analyzed via fixed-effects meta-analysis weighted by the inverse of the variance. Results were presented as odds ratios (ORs) (and 95% confidence intervals (95% CI)) of RWG per 1-week increase in gestational age acceleration. We performed sensitivity analyses by adding the mode of delivery and removing estimated cell counts from the confounders. Analyses of DNA methylation gestational age acceleration were further restricted by excluding children born from mothers affected by gestational diabetes and children of non-white European ethnicity.

#### Prediction of rapid weight growth

We estimated how well RWG was predicted using identified signatures compared to conventional risk factors using the RandomForest R package (version 4.7.1.1). We used three sets of variables: (1) DNA methylation levels of CpGs significantly associated in the meta-analysis of EWAS with RWG at *p*_Suggestive_<1e−05, (2) conventional risk factors (including maternal education level [21], age at delivery [22], smoking during the index pregnancy [23], pre-pregnancy BMI [24], parity [25], and child gestational age [26] and sex), (3) DNA methylation levels of CpGs significantly associated in the meta-analysis of EWAS with RWG at *p*_Suggestive_<1e−05 in combination with conventional risk factors. Residuals from linear regression between DNA methylation levels and bead array row, bisulfite conversion batch, and cell proportions were used instead of the actual measures of DNA methylation levels to account for technical noise. Missing values were imputed using the missForest package. Data were split into training (including ALSPAC, ENVIR*ON*AGE, and GXXI, being approximately 80% of the total study population) and test (including EXPOsOMICS cohorts for a total of approximately 20% of the total study population) sets. The model was trained on the training set with 10,000 trees. In the test set, the model performance was evaluated using the area under the curve (AUROC) to assess the classifier’s goodness of fit. The model calibration was performed by visualizing the agreement between the observed and predicted values [58].

Using the same methodology, we also tested the prediction of RWG by all the CpG sites belonging to each DMR identified as associated with RWG with FDR-adjusted *p*-values in DMRcate and Siddak *p*-values in ENmix-comb-p < 0.01.

#### Mediation analysis

For each CpG associated in the meta-analysis of EWAS with RWG at *p*_Suggestive_ < 1e−05, we tested in each cohort separately, or in the case of the EXPOsOMICS cohorts together as the separate sample sizes are too small, if DNA methylation was mediator (M) of the effect of conventional risk factors (including maternal education level [21], age at delivery [22], smoking during the index pregnancy [23], pre-pregnancy BMI [24], parity [25], and child gestational age [26]) here named exposures (E) on RWG (Y) via model-based single mediation analysis using the imputation approach [59] via the medflex R package (version 0.6.7). We accounted for technically induced variation by using the residuals from a preliminary linear model of each CpG (as outcome variable) adjusted for bead array row and bisulfite conversion batch, instead of the levels of DNA methylation. Mediation models were adjusted for conventional risk factors other than the exposure of interest, child sex and cohort membership (for EXPOsOMICS only) and blood cell estimations. Child gestational age was excluded from confounders as it could be a mediator of the effect of the other prenatal exposures. Mediation analysis of maternal education was adjusted only for child sex and cohort membership (for EXPOsOMICS only) as the other prenatal exposures may act as mediators of its effect. We estimated the total effect (TE), which was further decomposed into the natural indirect effect (NIE) operating via each mediator M and the natural direct effect (NDE). These effects were meta-analyzed via fixed-effects meta-analysis weighted by the inverse of the variance using the R package metafor and considered significant if *p*_Bonferroni_ was < 1.14e−03 (0.05/44). Results were reported as ORs (and 95% CI). The TE ORs express the effects of Y on E. The NIE and NDE ORs express the effects of E on Y, mediated and unmediated by M.

We applied the same methodology to explore mediation via the DMRs associated with RWG (with FDR-adjusted *p*-values in DMRcate and Siddak *p*-values in ENmix-comb-p < 0.01) using all the CpGs belonging to each DMR as joint multiple mediators. Results were considered significant if *p*_Bonferroni_ was< 3.12e−03 (0.05/16). Finally, we explored mediation via gestational age acceleration and considered significant results with a *p*-value <0.05.

#### Transcriptomics and metabolomics functional analysis

To better characterize the functional role of the CpG associated in the meta-analysis of EWAS with RWG at *p*_Suggestive_ < 1e−05, we integrated CpG measurements with the levels of the transcriptome available for the children of ENVIR*ON*AGE participating in EXPOsOMICS (*N* = 152) and of the metabolome available in the entire set of EXPOsOMICS participants (*N* = 444).

CpG methylation levels were regressed against transcript (*n*= 29,164 transcripts) and metabolite (*n* = 4712 metabolic features) signals (as outcomes) in linear mixed models with random effects on bead array row and bisulfite conversion batch and adjusted for maternal tobacco smoke during pregnancy, maternal education level at delivery and pre-pregnancy BMI, age at delivery, and parity, gestational age, child sex, cohort membership (for metabolomics analyses), and blood cell estimations. Results were presented as mean (and standard error) differences in transcript and metabolite levels per 10% increase in methylation level at each CpG. Results were considered significant if *p*_Bonferroni_ was< 3.90e−08 (0.05/(44×29,164)) for transcriptomics analyses and < 2.41e−07 (0.05/(44×4712)) for metabolomics analyses.

Using the same methodology, we evaluated the functional role of all the CpGs in the identified DMRs having FDR-adjusted *p*-values in DMRcate and Siddak *p*-values in ENmix-comb-p < 0.01. Results from these analyses were considered significant if *p*_Bonferroni_ was < 1.79e−08 (0.05/(96×29,164)) for transcriptomics analyses and < 1.10e−07 (0.05/(96×4712)) for metabolomics analyses.

Transcriptomics analyses were further restricted to *cis* transcripts (on the same chromosome up to 10Kb in both directions from CpGs’ position).

Overrepresentation analyses (ORA) of the transcripts associated with CpGs in the functional analyses below the *p*_Suggestive_ of 1e−05 were performed using ConsensuspathDB online tool (http://consensuspathdb.org/), with a pathway considered significantly enriched if the *p*-value was smaller than 0.05 and included at least three genes.

#### Analyses of childhood overweight

To assess if the identified CpGs were associated with childhood overweight, we used information on overweight in childhood available in ALSPAC, GXXI, and EXPOsOMICS children (*N*=1916).

Association between cord blood DNA methylation and childhood overweight was assessed via single study-specific logistic regression models that were integrated via fixed-effect meta-analysis using the same methodology adopted in the main analysis and adding age at the measurement of BMI in childhood among the confounders. Results were presented as mean (and standard error) differences in log odds of childhood overweight per 10% increase in methylation for each CpG. We performed a look-up of the differentially methylated CpGs in the meta-analysis of EWAS at *p*_Suggestive_ < 1e−05, which were considered to be statistically significant if *p*_Bonferroni_< 1.14e−03 (0.05/44), while DMRs were considered if both FDR-adjusted *p*-values in DMRcate and Siddak *p*-values in comb-p were < 0.01.

#### Comparison with previous findings

We investigated whether CpG sites associated with childhood anthropometrics, as identified by our recent systematic review [60], were associated with RWG by a look-up of these hits (*N*=1526 CpGs) in our study population. Results were considered significant if *p*_Bonferroni_< 3.28e−05 (0.05/1526).

We compared our results at single CpG sites with those of a previous meta-EWAS of birthweight (*N*=8825 children) and we tested the enriched CpGs significantly overlapped with our analysis using the chi-square test.

## Results

### Population

Of the total 2003 participants, 34% (677) were classified as having RWG (Table 1). In most of the studies, children born at a shorter gestational age, with lower birthweight, that were first born, and with mothers who smoked during the pregnancy were more likely to show RWG (Additional file 2: Table S1).

### Association between DNA methylation and rapid growth

**Fig. 2:**
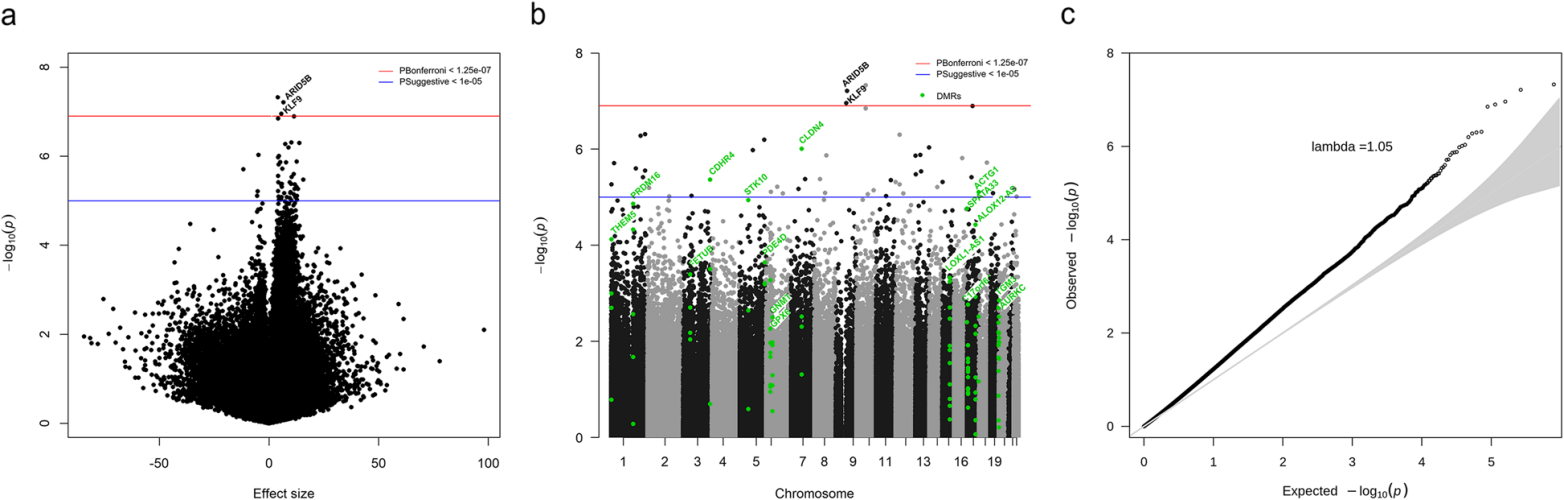
Results from the meta-analysis of EWAS of rapid weight growth. **a** Volcano plot shows the −log_10_*p*-values (vertical axis) against the estimates (horizontal axis) of each CpG site. **b** Manhattan plot shows the −log_10_*p*-values (vertical axis) against the chromosomal position (horizontal axis) of each CpG site. **c** Quantile-quantile plots of the observed log_10_*p*-values (vertical axis) against the expected log10 *p*-values (horizontal axis) of each CpG site. Estimates are reported per 10% increase in methylation levels

Based on the meta-analyses, 49 CpGs were associated with RWG at *p*_Suggestive_ < 1e−05 (Fig. 2a, b and Supplementary Table 2), and among them three (cg14459032, cg25953130 annotated to *ARID5B*, and cg00049440 annotated to *KLF9*) had *p*_Bonferroni_< 1.25e−07 (Table 2). Forty-five (92%) of these CpGs showed a positive association with RWG (regression coefficients range=0.4−1.6%). Sixteen DMRs were identified as being associated with RWG with FDR- and Siddak adjusted *p*-value <0.01 in DMRcate and ENmix-comb-p, respectively (Fig. 2b and Table 3).

**Table 2:** Genome-wide significant CpGs in the meta-analysis of EWAS of rapid weight growth

CpG	Gene name	Gene region	Coefficient	Standard error	***p***-value	***I***^**2**^	***I***^**2**^***p***-value
cg14459032	-	-	4.03	0.74	4.71e−08	0.00	0.68
cg25953130	*ARID5B*	Body	6.61	1.22	6.14e−08	43.42	0.15
cg00049440	*KLF9*	Body	5.56	1.05	1.10e−07	14.15	0.32

Coefficients are reported per 10% increase in methylation levels. *I*^2^ heterogeneity. CpGs depicted have *p* Bonferroni < 1.25e-07

**Table 3:** Differentially methylated regions with rapid weight growth

Gene annotation	ENmix-comb-p					DMRcate				
	Genomic coordinates	Gene region	UTR	***N***	Siddak ***p***-value	Genomic coordinates	Gene region	UTR	***N***	FDR ***p***-value
*THEM5*	chr1:151825973-151826205	Overlaps 5′	Overlaps 5′ UTR	4	6.75e−08	chr1:151825974-151826533	Overlaps 5′	Overlaps 5′ UTR	5	1.97e−05
*CLDN4*	chr7:73241728-73242028	Upstream	-	4	2.65e−07	chr7:73241729-73242028	Upstream	-	4	7.02e−05
*FETUB*	chr3:186353574-186353721	Promoter	-	2	2.65e−07	chr3:186353350-186353721	Promoter	-	3	3.01e−04
*LOXL1-AS1*	chr15:74218696-74218921	Covers exon(s)	Inside transcription region	7	2.65e−07	chr15:74218418-74219307	Covers exon(s)	Inside transcription region	11	1.97e−05
*PRDM16*	chr1:3239991-3240227	Inside intron	Inside transcription region	3	2.65e−07	chr1:3239712-3240227	Inside intron	Inside transcription region	4	2.85e−04
*STK10*	chr5:171616387-171616574	Promoter	-	3	2.65e−07	chr5:171616388-171616574	Promoter	-	3	1.03e−04
*C17orf64*	chr17:58499678-58499911	Overlaps 5′	5′ UTR	7	7.06e−07	chr17:58499300-58500186	Overlaps 5′	Overlaps 5′ UTR	9	1.54e−04
*TGM3*	chr20:2276434-2276663	Overlaps 5′	5′ UTR	5	8.59e−07	chr20:2275700-2277040	Overlaps 5′	Overlaps 5′ UTR	8	1.93e−04
*GPX6*	chr6:29454671-29454954	Upstream	-	5	1.30e−06	chr6:29454557-29455256	Upstream	-	8	3.91e−04
*ACTG1*	chr17:79485528-79485709	Upstream	-	2	1.33e−06	chr17:79485529-79485934	Upstream	-	3	5.59e−04
*PDE4D*	chr5:58883391-58883430	Inside intron	Inside transcription region	2	1.33e−06	chr5:58882939-58883430	Inside intron	Inside transcription region	3	4.03e−04
*CDHR4*	chr3:49837347-49837654	Promoter	-	3	2.35e−06	chr3:49837348-49837689	Promoter	-	4	8.14e−04
*SPATA33*	chr16:89734985-89735184	Inside intron	Inside transcription region	2	2.35e−06	chr16:89734986-89735184	Inside intron	Inside transcription region	2	2.54e−03
*ALOX12-AS1*	chr17:6899296-6899380	Inside intron	3′ UTR	6	3.13e−05	chr17:6898738-6899758	Inside intron	3′ UTR	13	1.22e−03
*AURKC*	chr19:57742344-57742421	Overlaps 5′	5′ UTR	3	5.09e−05	chr19:57741988-57742444	Overlaps 5′	5′ UTR	9	9.22e−04
*GNMT*	chr6:42927939-42927959	Promoter	-	2	6.49e−03	chr6:42927940-42928079	Promoter	-	7	4.31e−03

*N* number of CpGs in each DMR, *FDR* false discovery rate, *UTR* untranslated regions. DMRs depicted have FDR-adjusted in DMRcate and Siddak-adjusted *p*-values in ENmix-comb-p of < 0.01)

**Fig. 3:**
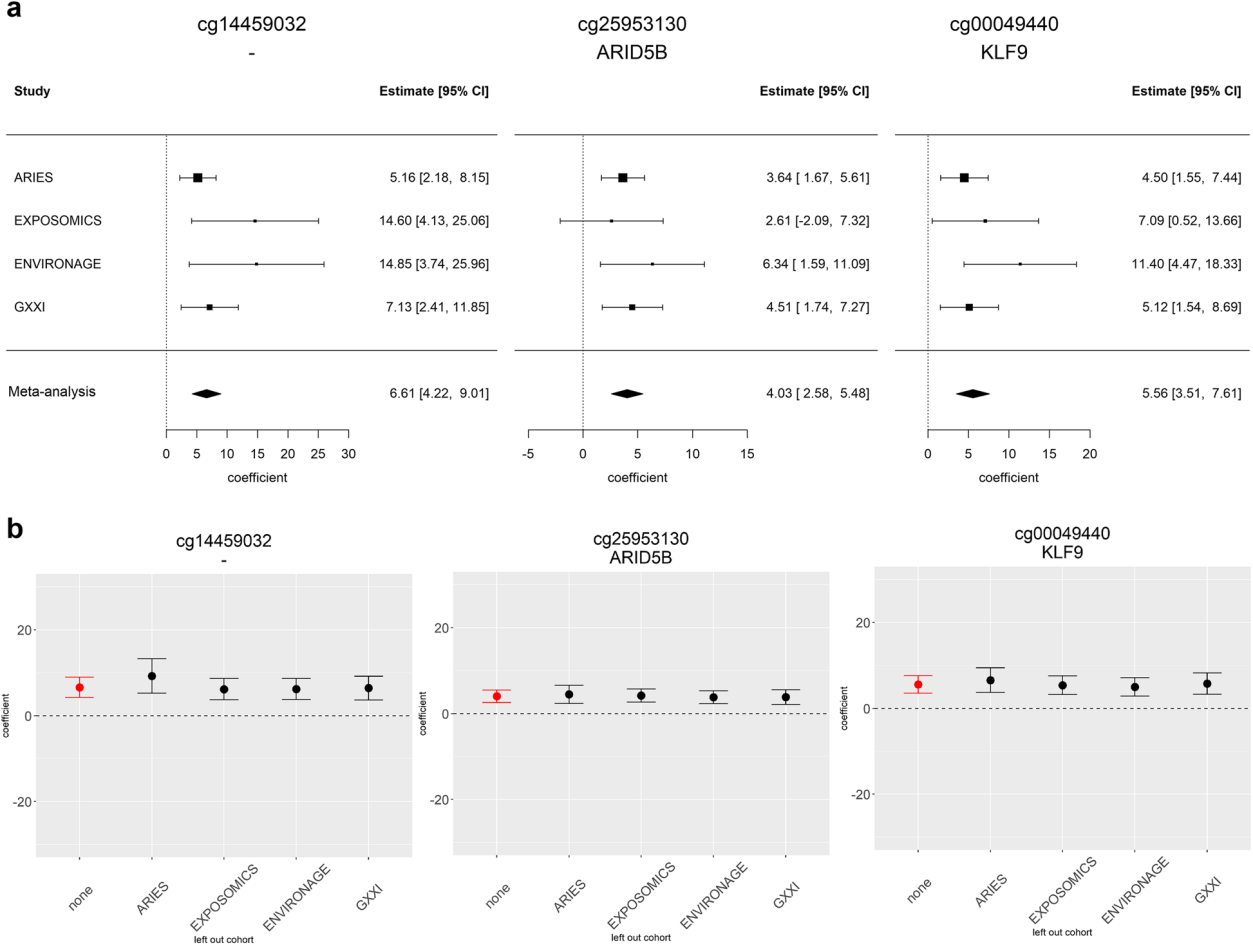
CpGs associated with rapid weight growth at *p*_Bonferroni_<1.25e−07 in the meta-analysis of EWAS. **a** Forest plots show log odds of showing rapid weight growth per 10% increase of methylation levels and 95% confidence intervals from single EWAS and pooled in the meta-analysis. **b** Plots show log odds of showing rapid weight growth per 10% increase of methylation levels and 95% confidence intervals from leave out one cohort at time analyses. 95% CI 95% confidence interval

Forest plots showing coefficients per each single study did not show evidence of heterogeneity among CpGs with *p*_Bonferroni_ < 1.25e−07 (Fig. 3). Visualization of *I*^2^*p*-values via QQ plot did not reveal heterogeneity (Additional file 3: Fig. S1). Evidence of high between-study heterogeneity (*I*^2^> 50%) was detected for five (cg04677123, cg22341513, cg22807187 annotated to *SNORD115-15*, cg24335751 annotated to *PRDM16*, and cg07780199 annotated to *CRCT1*) of the 49 CpGs with *p*_Suggestive_ < 1e−05 (Additional file 2: Table S2 and Additional file 3: Fig. S2), which were removed from subsequent analyses. Analyses leaving out one cohort at a time indicated that no single study had an influential effect on meta-analysis results (Fig. 3 and Additional file 3: Fig. S2). Quality control of a single EWAS of RWG revealed the inflation measured by the *λ*_bacon_ ranged from 0.92 to 1.05 indicating little deflation or inflation from the expected *p*-values, as detailed in Additional file 3: Fig. S3. In the meta-analysis of EWAS, *λ*_bacon_ was 1.05 suggesting little evidence of inflation (Fig. 2c).

In sensitivity analyses, we found that the direction of the associations at the 49 CpGs with *p*_Suggestive_ < 1e−05 was stable, while *p*-values were attenuated. Adding birthweight as a confounder, the significance of all the associations faded above *p*_Suggestive_ of 1e−05, except for six CpGs (cg13710814, cg05455036, cg00747477, cg26682904, cg23404711, cg06846833; Additional file 2: Table S3). Adding delivery mode as a covariate, *p*-values for the association of all the CpGs were still below *p*_Suggestive_ of 1e−05, with the exception of one CpG (cg16072126), and *p*-values of all the three genome-wide significant CpGs in the main analyses were still below the *p*_Bonferroni_ significance level of 1.25e−07 (Additional file 2: Table S3). Removing cell types from confounders, 21 CpGs had still *p*_Suggestive_ < 1e−05, and the *p*-values of two of the genome-wide significant CpGs (cg25953130 and cg14459032) were still below the *p*_Bonferroni_ significance level of 1.25e−07 (Additional file 2: Table S3). Excluding subjects with mothers having had gestational diabetes or unknown information (*N* = 113) and children that were non-white European or unknown information (*N*=53) (Additional file 2: Table S4), *p*-values of the association of 28 and 35 CpGs were still below *p*_Suggestive_ of 1e−05, respectively, in each analysis, and *p*-value of one out of the three genome-wide significant CpGs faded above the *p*_Bonferroni_ threshold of 1.25e−07 in the analyses excluding gestational diabetes cases (cg00049440) and non-white European children (cg14459032) (Additional file 2: Table S3). In sensitivity analyses of the DMR analyses, when birthweight was added as a confounder, the significance of all the DMRs faded above the FDR- and Siddak-adjusted *p*-value threshold of 0.01 in DMRcate and ENmix-comb-p, respectively, except for the *THEM5* and *AURKC* DMRs (Additional file 2: Table S5). When adding delivery mode, all the regions, except for the *AURKC* DMR had FDR- and Siddak-adjusted *p*-values below of 0.01 in DMRcate and ENmix-comb-p, respectively (Additional file 2: Table S5). Excluding mothers with gestational diabetes or unknown information and children that were non-white European or unknown information (Additional file 2: Table S4), three (*LOXL1-AS1*, *ACTG1*, and *ALOX12-AS1*) and one (*FETUB*) DMRs, respectively, faded above the FDR- and Siddak-adjusted *p*-values of 0.01 in DMRcate and ENmix-comb-p, respectively (Additional file 2: Table S5). The *p*-value of the associations with rapid growth remained below FDR- and Siddak-adjusted *p*-value threshold of 0.01 in DMRcate and ENmix-comb-p, respectively, for all the regions, apart from seven (located at *FETUB*, *LOXL1-AS1*, *PRDM16*, *C17orf64, STK10* and *ALOX12-AS1*, and *GNMT*), in models not adjusted for cell types (Additional file 2: Table S5).

### Association between gestational age acceleration and rapid weight growth

**Fig. 4:**
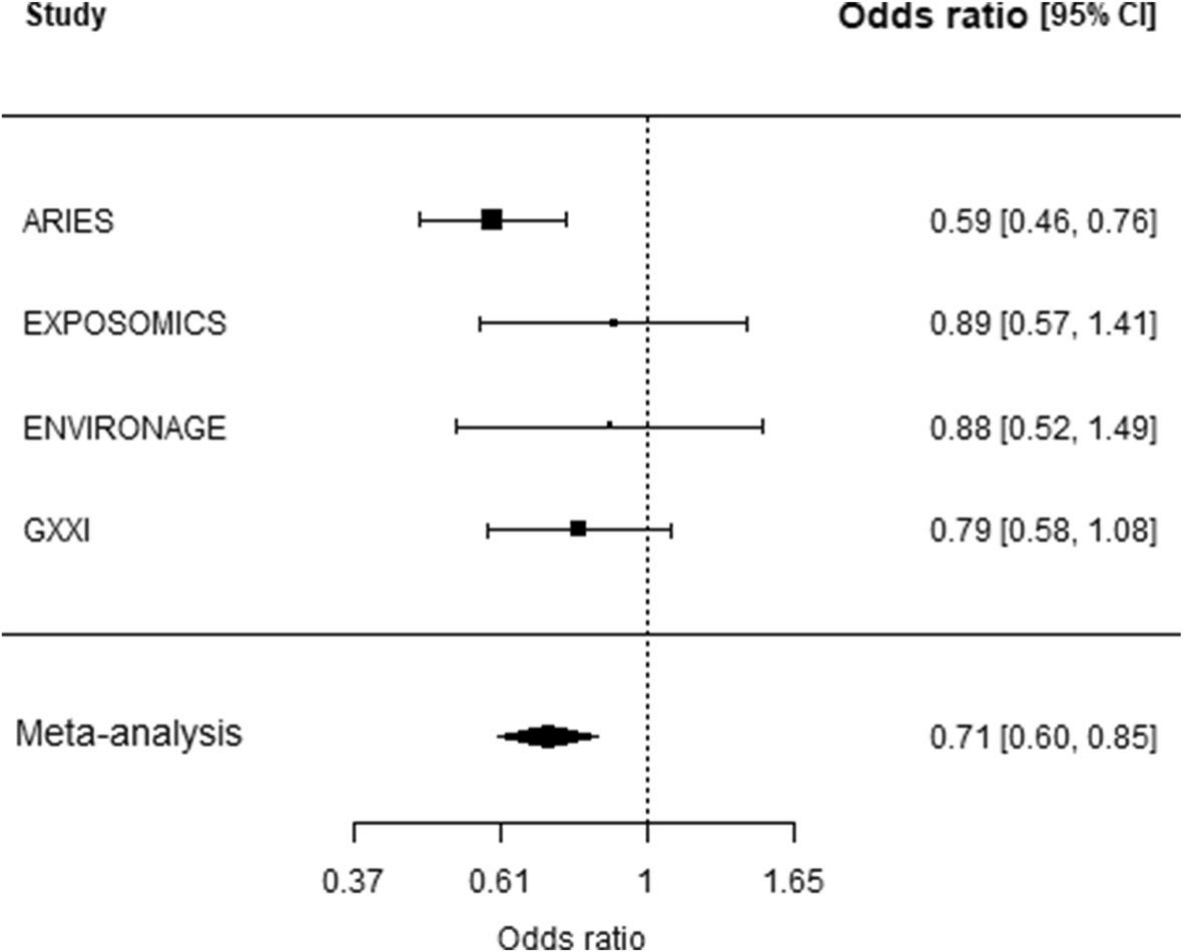
Association of gestational age acceleration and rapid weight growth. Forest plot show odds ratio and 95% confidence intervals of showing rapid weight growth for an increase of 1 week of gestational age acceleration from the single study analyses and pooled in the meta-analysis. 95% CI 95% confidence interval

In all the studies, few CpGs (ranging from 0 to 8) were missing for the calculation of gestational age acceleration using both clocks (Additional file 2: Table S6). The correlation of Bohlin’s clock with chronological gestational age was stronger (correlation coefficient *r* range= 0.63–0.73) than Knight’s (correlation coefficient *r* range = 0.33–0.55), which was excluded in the subsequent analyses (Additional file 2: Table S6 and Additional file 3: Fig. S4). The meta-analysis found that gestational age acceleration was associated with decreasing risk of showing RWG (OR per one gestational age acceleration week=0.71, 95% CI= 0.60–0.85, *p*-value=9.75e–04) (Fig. 4). Adding delivery mode, removing estimated cells from confounders, excluding mothers with gestational diabetes or unknown information and children that were non-white European or with unknown information, did not mitigate the results (Additional file 3: Fig. S5).

### Rapid weight growth prediction

**Fig. 5:**
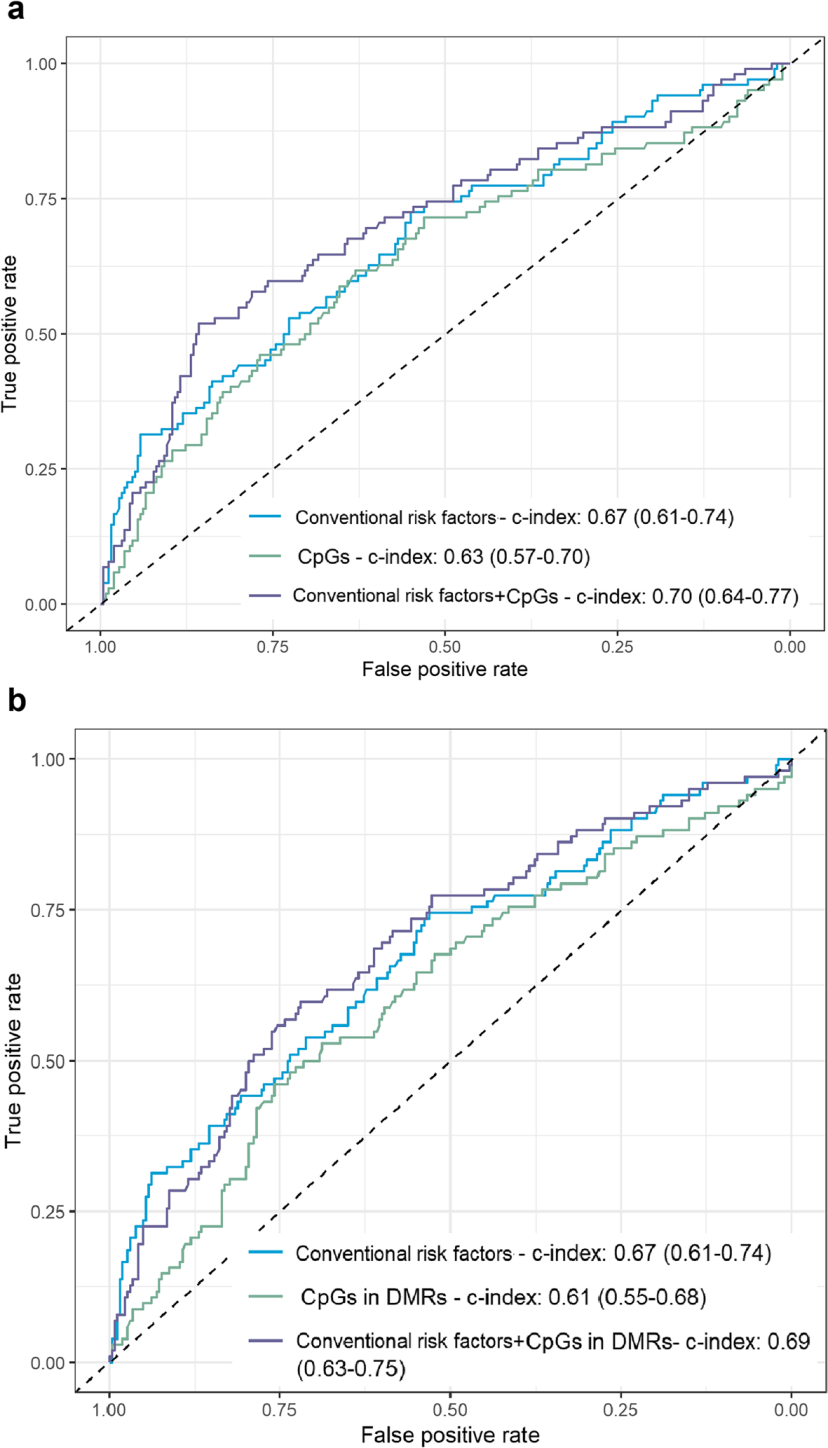
Receiver operating characteristic (ROC) mean value of Random Forest prediction models of rapid weight growth. **a** ROC curves of models including conventional risk factors, 44 CpGs associated with rapid weight growth at *p*_Suggestive_< 1e−05 in the meta-analysis of EWAS, and both as identified by the color legend. **b** ROC curves of models including conventional risk factors, 96 CpGs belonging to the 16 differentially methylated regions associated with rapid weight growth at FDR-adjusted *p*-values in DMRcate and Siddak *p*-values in ENmix-comb-p were < 0.01, and both as identified by the color legend. DMRs differentially methylated regions; conventional risk factors include maternal tobacco smoke during pregnancy, education level at delivery, pre-pregnancy BMI, age, parity, and child sex and gestational age

Using Random Forest classification, the rapid growth prediction model showed a predictive ability of an AUROC value of (Fig. 5) 0.63 (95% CI=0.57–0.70) by including to the model the 44 CpGs related to RWG at *p*_Suggestive_ < 1e−05; 0.61 (95% CI= 0.55–0.68) by including the 96 CpGs belonging to the 16 DMRs related to RWG at FDR-adjusted *p*-value in DMRcate and Siddak *p*-values in ENmix-comb-p < 0.01; 0.67 (95% CI= 0.61–0.74) by including conventional risk factors; 0.70 (0.64–0.77) when both the 44 CpGs related to RWG at *p*_Suggestive_ < 1e−05 and the conventional risk factors were incorporated in the model; and 0.69 (0.63–0.75) when both the 96 CpGs belonging to the 16 DMRs associated with RWG at FDR-adjusted *p*-value in DMRcate and Siddak *p*-values in ENmix-comb-p <0.01 and the conventional risk factors were incorporated in the model. Prediction models developed with the CpGs related to RWG (Additional file 3: Fig. S6) or CpGs belonging to DMRs related to rapid growth in DMRcate and Siddak *p*-values in ENmix-comb-p (Additional file 3: Fig. S7) in addition to the conventional risk factors showed moderately low classification of RWG.

### Mediation analysis

**Fig. 6:**
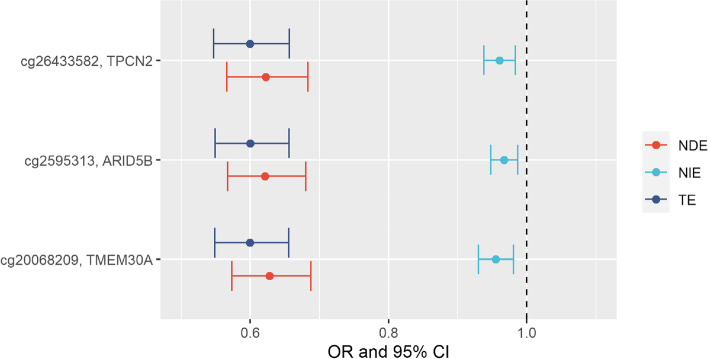
Effects of gestational age on rapid weight growth mediated by three CpGs. The plot represents on the *x*-axis the point estimates odds ratio (dots) and 95% confidence intervals (bars) of the mediation analysis of the 1-week increase of gestation on rapid weight gain via DNA methylation. Only CpGs with *p*-values of the natural indirect effects below *p*_Bonferroni_ threshold of 1.14e−03 (0.05/44 CpGs associated with rapid weight growth at *p*_Suggestive_< 1e−05 in the meta-analysis of EWAs) are shown. NDE natural direct effect, NIE natural indirect effect, TE total effect

Among the 44 CpGs associated with RWG at *p*_Suggestive_ < 1e−05, three CpGs (cg20068209, located on *TMEM30A*; cg25953130, located on *ARID5B*; and cg26433582, located on *TPCN2*) partly mediated the effect of gestational age on RWG with *p*_Bonferroni_< 1.14e−03 (0.05/44), yet the total effect was mainly explained through other pathways (Fig. 6). While some of the 44 CpGs were highly correlated between themselves, these three CpGs were not (Additional file 3: Fig. S8). No mediation effect on RWG was found at the other CpGs or DMRs, for any exposure under study (all *p*_Bonferroni_ for DMRs > 3.11e−03 (0.05/16)). Gestational age acceleration did not mediate the effect of any exposure under study on rapid weight growth (Additional file 2: Table S7, all *p*-values>0.05).

### Downstream analysis

In the ENVIR*ON*AGE cohort, among the 44 CpGs associated with RWG at *p*_Suggestive_ < 1e−05, cg20068209 (located on *TMEM30A*) and cg20076442 were negatively associated with ten transcripts (of *XLOC_008311*, *LOC102724800*, *XLOC_014512*, *AADACP1*, *SSTR5-AS1*, *TTTY16*, *CHMP7*, and *FLJ32756*) (at *p*_Bonferroni_ < 3.90e−08) (Additional file 3: Fig. S9A). Restricting the analyses to *cis* signals, cg20038219 was found positively associated with the expression of *WBSCR27* and cg20938359 (located on *SLC6A13*) was negatively associated with a SLC6A13 transcript (at *p*_Bonferroni_ < 2.63e−03) (Additional file 3: Fig. S9B). Identified gene expression signals below the *p*_Suggestive_ < 1e−05 (*N*=315) were mapped to 176 unique genes which were involved in seven significant pathways (*p*-values < 0.05), including MAPK signaling and cell-cell communication pathways (Additional file 2: Table S8).

Among the 96 CpGs belonging to the 16 DMRs (Additional file 2: Table S9) associated with RWG at FDR-adjusted *p*-value in DMRcate and Siddak *p*-values in ENmix-comb-p <0.01, ten CpGs (located in *SPATA33*, *CLDN4*, and *GNMT* DMRs) were associated with seven transcripts (of *PEX6*, *HMGB4*, *ETNK2*, *MCAT*, and *WBSCR27*) (Additional file 3: Fig. S9C). Restricting the analyses to *cis* signals, 11 CpGs (located in *CLDN4*, *GNMT*, and *THEM5* DMRs) were associated with five transcripts (of *WBSCR27*, *PEX6*, *GNMT*, and *THEM5*) (at *p*_Bonferroni_ < 4.42e−03) (Additional file 3: Fig. S9D). Notably, WBSCR27 and PEX6 transcripts were identified both at the transcriptome-wide level and in the *cis*-restricted analyses (Additional file 3: Fig. S8C, 8D). Identified gene expression signals below the *p*_Suggestive_ < 1e−05 (*N* = 297) were mapped to 175 unique genes and were involved in 32 significant pathways (*p*-values <0.05), including type II diabetes mellitus, insulin signaling, sphingolipid signaling, growth hormone synthesis, secretion and action, immune system, and nervous system development pathways (Additional file 2: Table S8).

No association was found between the 44 CpGs associated with RWG at *p*_Suggestive_ < 1e−05 and the metabolome in the EXPOsOMICS population (minimum *p*-value= 8.36e−06 versus *p*-value Bonferroni threshold=2.41e−07, Additional file 3: Fig. S10A). The 96 CpGs belonging to the 16 DMRs associated with RWG at FDR-adjusted *p*-value in DMRcate and Siddak *p*-values in ENmix-comb-p <0.01 were not associated with the metabolome (minimum *p*-value= 1.01e−06 versus *p*-value Bonferroni threshold = 1.10e−07, Additional file 3: Fig. S10B).

### Association between DNA methylation and childhood overweight

The study population for the analyses of childhood overweight included 1916 children, with a prevalence of overweight of 23.4% (*N*=435) at a mean age of 6.5 years (Additional file 2: Table S10). Among the 44 CpGs that were associated with RWG at *p*_Suggestive_ < 1e−05, none was associated with childhood overweight at *p*_Bonferroni_ < 1.14e−03 (0.05/44) (cg21844291 had the smallest *p*-value being equal to 0.05, Additional file 2: Table S11). Of the 16 DMRs associated with RWG at FDR-adjusted *p*-value in DMRcate and Siddak *p*-values in ENmix-comb-p <0.01, the *AURKC* DMR was also associated with childhood overweight (FDR-adjusted *p*-value in DMRcate=2.64e−13 and Siddak *p*-values in ENmix-comb-p=1.86e−15) (Additional file 2: Table S12). Gestational age acceleration was not associated with childhood overweight (OR per one gestational age acceleration week = 0.85, 95% CI= 0.70–1.04, *p*-value=0.12) (Additional file 3: Fig. S11).

### Comparison with previous findings

In the look-up of the 1526 CpG sites associated with anthropometrics in children by a previous systematic review [60], no signal was significantly associated with RWG in our meta-analysis (Additional file 3: Fig. S12 [60], minimum *p*-value for cg00753924 located on *RXRA* = 8.72e−05 versus *p*_Bonferroni_< 3.28e−05 (0.05/1526)). Out of the 44 CpGs we identified as associated with RWG, 19 were among the 2423 CpGs (chi-squared *p*-value<1e−16) associated with birthweight with *p*-value<1e−05 (out of 473,864 CpGs) in the previous PACE meta-EWAS [14].

## Discussion

In this meta-EWAS of six population-based cohorts, including a total of more than 2000 children, we found evidence of an association of cord blood DNA methylation with RWG during the first year of life at the DMR (*N*=16) level (using FDR-adjusted *p*-values in DMRcate and Siddak *p*-values in ENmix-comb-p thresholds of 0.01, respectively), although only three CpGs reached genome-wide significance at the single CpG level (*p*_Bonferroni_ threshold of 1.25e−07). Considering a less stringent significance level (*p*_Suggestive_ threshold of 1e−05), our findings of 44 CpGs were enriched in CpGs previously reported in relation to birthweight. However, six were associated with RWG independently of birthweight, indicating that some epigenetic signatures might only be related to RWG while others are a shared mechanism with birthweight (or a correlate of this measure). Our findings also showed that higher gestational age acceleration was associated with a lower risk of experiencing RWG. A small improvement in predicting RWG was obtained by coupling the top CpGs associated with RWG with conventional risk factors. Translating our findings to the metabolomic and gene expression level indicated that the identified CpGs were associated with differential expression of different genes and long non-coding RNAs, but not with metabolic changes. Exploring a set of prenatal exposures previously associated with RWG [21–26], we found that gestational age was inversely associated with RWG and part of this association was mediated by the methylation level at three CpGs. *AURKC* DMR was associated with RWG and childhood obesity between 4 and 8 years old.

The identified genome-wide significant CpGs (cg14459032, cg25953130, and cg00049440) are here related to RWG for the first time; nevertheless, they have been previously associated with birthweight [14, 61, 62], which is not surprising given the fact that RWG is calculated based on the difference between weight at 1 year and birthweight. cg14459032 was not mapped to any gene, but enhancing annotation as previously described [63], we found the nearest known gene within 10 MB is *PCSK5*, encoding for a proprotein convertase subtilisin/kexin type 5, which genome-wide association studies have associated to height [64] and high-density lipoprotein cholesterol [65]. cg25953130 is located on the AT-rich interactive domain-containing protein 5B (*ARID5B*), which encodes for a transcriptional repressor of beige adipocyte biogenesis leading to a shift towards increasing energy-storing white adipocytes [66]. cg00049440 is located on the Kruppel-like factor 9 (*KLF9*), which encodes a zinc-finger transcription factor that regulates the adipocyte differentiation by binding to peroxisome proliferator-activated receptor γ2 (PPARγ2) promoter [67] and was associated with BMI in adults in a genome-wide association study [68]. We found that increasing cord blood DNA methylation at these sites was associated with a higher occurrence of RWG, which is consistent with the previous findings of an inverse relationship with birthweight, which in turn is inversely associated with RWG [61]. Since the temporal co-occurrence of birthweight and DNA methylation impedes assessing whether birthweight is a mediator or a confounder in the association between DNA methylation and RWG, our main analyses were not adjusted for birthweight. The involvement of birthweight in these associations was further demonstrated in sensitivity analyses, where adjustment for birthweight revealed that the strength of association decreased for the three CpG sites, and the significance faded at a crude *p*-value of 0.05 at cg25953130. Similar to single CpGs, only two DMRs were associated with RWG independently of birthweight. Our findings suggest that birthweight mostly has an influence on DNA methylation, rather than being a mediator of its effect on RWG. Studies in adults and children supported this hypothesis [69, 70]. Along this line, DNA methylation between 10 and 18 years at the one gene (*RPH3AL*) was found to be predicted by BMI of 10-year-old children [71]. *THEM5* and *AURKC* DMRs (among the 16 DMRs associated with RWG at FDR-adjusted *p*-values in DMRcate and Siddak *p*-values in ENmix-comb-p< 0.01) and six CpGs (among the 44 CpGs associated with RWG at *p*_Suggestive_ < 1e−05) were associated with RWG independently from birthweight. This finding may indicate that some epigenetic signatures are shared by birthweight and RGW, while others are only related to RWG. Thioesterase superfamily member 5 (*THEM5*) encodes a thioesterase involved in the cardiolipin remodeling process, which in animal models has a critical role in the development of fatty liver [72]. Aurora kinase C (*AURKC*) encodes a protein kinase that regulates multiple key steps during the mitotic cell division process and whose expression levels in placentas are reduced in early-onset fetal growth-restricted pregnancies [73].

Prediction of RWG was slightly improved by adding the CpGs associated with RWG with *p*_Suggestive_<1e−05 or the CpGs belonging to DMRs associated with RWG (using FDR-adjusted *p*-values in DMRcate and Siddak *p*-values in ENmix-comb-p < 0.01) to conventional risk factors (c-index increased from 0.67 in models including only conventional risk factors to 0.70 and 0.69 in models including also the CpGs and the CpGs belonging to DMRs, respectively), which could be due to the CpGs laying on the casual paths linking the prenatal exposures to RWG. Our mediation analysis supports this hypothesis by the finding that three CpGs (g25953130, previously described, cg20068209 located on *TMEM30A*, and cg26433582 located on *TPCN2*) mediate the effect of gestational age on RWG, although most of the effect of gestational age on RWG is direct. Methylation levels of these CpGs at birth have been previously associated with gestational age [43] and birthweight [14]. cg25953130 in cord blood was also associated with maternal hypertensive disorders in pregnancy [74]. Our analyses could not exclude the possibility that maternal hypertensive disorders or other unmeasured confounders affect DNA methylation at birth, gestational age, and RWG later in infancy.

We identified 16 DMRs associated with RWG (at FDR-adjusted *p*-values in DMRcate and Siddak *p*-values in ENmix-comb-p < 0.01), by applying two independent methods (ENmix-comb-p [56] and DMRcate [57]), compared to three only single CpG sites. The difference in the number of identified signals may be due to the greater statistical power of DMR analyses. However, by identifying DMRs and CpGs, the mechanisms underlying the propensity to RWG involve multiple CpGs. In accordance with this finding, a previous study found that at birth DMRs but not single CpG were predictive of the waist-to-hip ratio of children at 5 years, including the *PRMD16* DMR [75], which we also found in our study associated with RWG. *PRMD16* encodes for a transcriptional regulatory factor that controls the differentiation of brown adipose tissue, and promotes the transition from white to beige adipose tissue and is closely related to obesity [76]. Methylation at single CpG sites of this gene was previously reported to be associated cross-sectionally to childhood obesity and severe obesity [15, 77], and more recently with overweight and obesity in adulthood, both at single CpG sites and methylation haplotypes [78]. However, in our study, we were not able to identify an association of this DMR with childhood overweight. *AURKC* DMR was the only one we identified significantly associated with both RWG and childhood overweight.

Among the identified DMRs, two, *LOXL1-AS1* and *ALOX12-AS1 DMR*, were mapped to genes putatively encoding long non-coding RNAs. DMRs are generally regarded as regions with functional roles. While most studies assume that DNA methylation influences proximal genes, recent multi-omics studies demonstrated that changes in DNA methylation could be associated with distal expression changes [79, 80]. Here, we explored the relationships between identified DNA methylation signals and gene expression at the genome-wide level, finding that three DMRs were associated with seven transcripts at the transcriptome-wide level, three of which were *cis* transcripts (located in the proximity of the DMRs). In addition, investigating the functional relevance of single CpG sites associated with RWG, we found that two CpGs, including cg20068209 (identified as a possible mediator in mediation analyses), were negatively associated with ten transcripts at the transcriptome-wide level, including several long non-coding RNAs. All these transcripts were located far from the identified CpGs (only one transcript shared the same chromosomal location of the associated CpG), both of which are enhancers. This finding suggests that the regulation of transcription goes beyond classical repression of promoter methylation and suggests a possible and more complex interplay between multiple epigenetic layers (methylation, chromatin, and non-coding RNAs). This hypothesis is consistent with findings from a previous study that miRNA expression levels in the placenta were correlated with methylation levels, which in turn were associated with the offspring’s anthropometry at 6 years [81].

Pathway analyses confirmed a functional role of the identified DNA methylation signals by identification of gene expression being involved in several pathways, including type II diabetes mellitus, insulin signaling*,* growth hormone synthesis, immune system, and cell signaling pathways.

Finally, our results indicate that children with greater gestational age acceleration have a lower risk of RWG (*p*-value<0.05), which is consistent with previous studies finding that greater gestational age acceleration was associated with higher birthweight [82] and lower weight growth from birth to 10 years (although this last association was not significant) [19]. These findings suggest that gestational age acceleration reflects factors related to gestation, such as growth and development, rather than general processes of aging (as adult age acceleration), and is advantageous in childhood. Maternal smoking and socioeconomic position have been associated with infant’s DNA methylation age at birth [23, 83] and RWG [21]. Our analysis found that maternal tobacco smoke during pregnancy, maternal age, and primiparity were associated with a higher risk of children showing RWG, but gestational age acceleration did not mediate these associations.

The main strengths of this study are the large sample size, the investigation of regional patterns of DNA methylation in addition to the single CpG sites, the incorporation of multiple omic layers in the analyses to explore downstream functionality of DNA methylation targets, and the mediation analyses that we have undertaken to disentangle the role of DNA methylation in the association between conventional risk factors and RWG. We first studied RWG independently of birthweight, as birthweight is part of the definition of RWG. Nevertheless, we tested all associations also taking birthweight into account and hereby further clarifying the role of birthweight in the epigenetic programming of RWG.

We acknowledge some limitations. We used DNA methylation in cord blood as an accessible collectible tissue for large populations, but we acknowledge that cord blood is a mix of cell types. We used estimated cell counts to account for cell variability using two reference methods specific to the umbilical cord that can be combined across studies [41, 42, 84]. DNA methylation was measured in the participating studies by the Illumina EPIC and 450K BeadChip arrays. We decided to meta-analyze DNA methylation levels measured in at least three studies which reduced the total number of CpG included in the analysis to less than 400,000, representing a small fraction (~1.5%) of the 28.3 million of total CpG sites in the genome [85]. Furthermore, genome-wide technologies are mainly employed to discover biomarkers that in turn require validation by other testing methods (e.g., pyrosequencing, quantitative methylation-specific polymerase chain reaction). Further validation of the identified signals is warranted. RWG was defined based on a fixed threshold (0.67 SD scores), which is well established in the literature [47]. Future studies analyzing the association of DNA methylation and the continuous increase of RWG are needed to robustly replicate these results and may provide further biological insights. Nevertheless, we performed sensitivity analyses for prenatal exposures (adjusting for delivery mode or excluding mothers affected gestational diabetes and children with non-white European ethnicity) known to affect both DNA methylation and RWG and most of the signals we identified were stable to sensitivity analyses, we cannot exclude the possibility of unmeasured confounding by genetic and other prenatal factors, including paternal factors. Finally, in the mediation analysis of single CpGs, we considered each potential mediator as independent, while genome-wide techniques are available for correlated high-dimensional data [86]. However, the three CpGs identified as mediators in our analysis were not correlated between themselves.

## Conclusions

In conclusion, our findings show that DNA methylation of regions of DNA, gestational age acceleration, and to a lesser extent single CpGs in cord blood were associated with RWG. The DNA methylation signatures identified showed a slight improvement in the prediction of RWG in addition to conventional risk factors. Furthermore, our mediation analysis indicated that some of the identified DNA methylation signatures were mediating the effect of gestational age on RWG. Finally, throughout the analysis incorporating RWG, we identified the AURKC DMR as predictive of overweight in childhood. Results were enriched in CpGs previously reported associated with birthweight. By increasing knowledge of the molecular mechanisms underlying RWG, our results can contribute to identifying target groups and developing prevention strategies already at birth.

## Supplementary Information

**Additional file 1.** Supplementary methods [27–38].

**Additional file 2: Table S1.** Comparison of characteristics of the ALSPAC, ENVIR*ON*AGE, EXPOsOMICS and GXXI cohorts by rapid weight growth status. **Table S2.** 49 CpGs associated with rapid weight growth in the meta-analysis of EWASs with P_Suggestive_ <1e-05. **Table S3.** Results from sensitivity analyses adding birthweight and delivery mode, removing cell types from confounders, excluding mothers with gestational diabetes and non-white European children, for the CpGs with P_Suggestive_ <1e-05 in the meta-analysis of EWASs of rapid weight growth. **Table S4.** Information available on maternal gestational diabetes, and white European ethnicity in the study population. **Table S5.** Results from sensitivity analyses of differentially methylated regions adding birthweight and delivery mode, removing cell types from confounders, excluding mothers with gestational diabetes and non-white European children. Results are shown only if the region has FDR- and Siddak adjusted *p*-value < 0.01 in DMRcate and ENmix-comb-p, respectively, in sensitivity analyses. **Table S6.** Count of missing CpGs for calculation of gestational age clocks and Spearman’s correlation coefficients between DNA methylation and chronological gestational age. **Table S7.** Results from meta-analyses of total, natural direct and indirect effect of prenatal exposures on rapid weight growth via gestational age acceleration. **Table S8.** Results from overrepresentation analyses (ORA) of transcripts associated at P_Suggestive_ < 1e-05 with the 44 CpGs related to RWG and the 96 CpGs belonging to the 16 DMRs related to RWG. **Table S9.** List of CpGs belonging to each of the 16 DMRs associated with RWG with FDR- and Siddak adjusted *p*-value <0.01 in DMRcate and ENmix-comb-p, respectively. **Table S10.** Study population characteristics of the four studies (ALSPAC, ENVIR*ON*AGE, EXPOsOMICS and GXXI) and total population included in the meta-analysis of EWAS of childhood overweight. **Table S11.** Look-up analysis of childhood overweight for the 44 CpGs associated at P_Suggestive_ <1e-05 in the meta-analysis of EWASs of rapid weight growth. **Table S12.** DMR that is associated with rapid growth and which is also significantly associated with childhood overweight.

**Additional file 3: Figures S1-S12** [60]. **Fig. S1.** QQ-plot of I_2_*p*-values of the meta-analysis of EWASs of rapid weight growth. **Fig. S2.** Forrest plots and plots from leave out one cohort at time meta-analysis from EWASs of rapid weight growth for the CpGs with P_Suggestive_ < 1e-05. **Fig. S3.** Quality control of cohort specific EWAS of rapid weight growth. Coefficients, standard errors and *p*-values distributions visualized via box plots and QQ-plot. **Fig. S4.** Cohort specific correlation plots between chronological and DNA methylation gestational age. **Fig. S5.** Forrest plot of the meta-analysis of gestational age acceleration and rapid weight growth in sensitivity analyses adding (A) delivery mode, (B) removing cell types from confounders, excluding mothers with (C) gestational diabetes and (D) non-white European children. **Fig. S6.** Calibration plots of Random Forest models of rapid weight growth including (A) conventional risk factors, (B) CpGs related to rapid weight growth and (C) both. **Fig. S7.** Calibration plots of Random Forest models of rapid weight growth including (A) conventional risk factors, (B) CpGs belonging to DMRs related to rapid weight growth and (C) both. **Fig. S8.** Heatmap shows Pearson’s correlation between methylation levels of the 44 CpGs associated with P_Suggestive_ <1e-05 in the meta-analysis of EWAS of rapid weight growth. Red boxes indicate the three CpGs identified in mediation analyses. **Fig. S9.** Volcano plots of the association between the 44 CpGs associated with rapid weight growth at P_Suggestive_ < 1e-05/the 96 CpGs belonging to the 16 DMRs associated with rapid weight growth at FDR-adjusted *p*-value in DMRcate and Siddak *p*-values in ENmix-comb-p <0.01 and the entire transcriptome (A/C) and restricted to cis transcripts (B/D). Red lines represent Bonferroni-significant threshold, and black lines suggestive threshold (*p*-value=10-e05). For analyses restricted to cis transcripts only Bonferroni-significant threshold is represented. **Fig. S10.** Volcano plots of the association between the 44 CpGs associated with rapid weight growth at P_Suggestive_ < 1e-05/the 96 CpGs belonging to the 16 DMRs associated with rapid weight growth at FDR-adjusted *p*-value in DMRcate and Siddak *p*-values in ENmix-comb-p <0.01 and the entire metabolome (A/B). Red lines represent Bonferroni-significant threshold, and black lines suggestive threshold (*p*-value=10-e05). **Fig. S11.** Forrest plot of the meta-analysis of the analysis of gestational age acceleration and childhood overweight. **Fig. S12.** Volcano plot from the look-up in the study population of the CpG sites associated with child anthropometrics in a previous systematic review by Alfano et al. [60].

## Data Availability

EXPOsOMICS data analyzed during the current study are available via NCBI Gene Expression Omnibus (GEO) repository with the accession nos. GSE151042 (https://www.ncbi.nlm.nih.gov/geo/query/acc.cgi?acc=GSE151042, for the methylome) and GSE151373 (https://www.ncbi.nlm.nih.gov/geo/query/acc.cgi?&acc=GSE151373, for the transcriptome) and via the MetaboLights repository with the accession no. MTBLS1684 (https://www.ebi.ac.uk/metabolights/MTBLS1684, for the metabolome). DNA methylation data from other cohorts that are not publicly available can be obtained from the authors upon reasonable request and subject to appropriate approvals. Code relevant to the analyses is available upon request to the authors.

## Abbreviations

*λ*_bacon_: Inflation using the bacon method
95% CI: 95% confidence intervals
ALSPAC: Avon Longitudinal Study of Parents And Children
ARID5B: AT-rich interactive domain-containing protein 5B
AURKC: Aurora kinase C
AUROC: Area under the curve
BMI: Body mass index
DMRs: Differentially methylated regions
DOHaD: Developmental origin of health and disease
ENVIR*ON*AGE: ENVironmental Influence ON early AGEing
EWAS: Epigenome-wide association study
GXXI: Generation XXI
*I*^2^: Heterogeneity
INMA: INfancia y Medio Ambiente
KLF9: Kruppel-like factor 9
OR: Odds ratio
ORA: Overrepresentation analysis
PACE: Pregnancy And Childhood Epigenetics
*p*_Bonferroni_ threshold: *p*-value Bonferroni-adjusted threshold set at 0.05/number of tests
PPARγ2: Peroxisome proliferator-activated receptor γ2
*p*_Suggestive_ threshold: *p*-value suggestive threshold set at 1e−05
QQ plot: Quantile-quantile plot
RWG: Rapid weight growth
SD: Standard deviation
THEM5: Thioesterase superfamily member 5
WG: Weight gain
WHO: World Health Organization

## References

[1] WHO Consultation on Obesity (1999: Geneva, Switzerland) & World Health Organization (2000). Obesity: preventing and managing the global epidemic: report of a WHO consultation.

[2] Development Initiatives (2018). 2018 Global Nutrition Report: Shining a Light to Spur Action on Nutrition.

[3] Cena H, Fiechtner L, Vincenti A, Magenes VC, De Giuseppe R, Manuelli M, et al. COVID-19 pandemic as risk factors for excessive weight gain in pediatrics: the role of changes in nutrition behavior. A narrative review. Nutrients. 2021;13(12):4255. 10.3390/nu13124255 PMC8707175 34959805

[4] Rankin J, Matthews L, Cobley S, Han A, Sanders R, Wiltshire HD (2016). Psychological consequences of childhood obesity: psychiatric comorbidity and prevention. Adolesc Health Med Ther.

[5] Lang JE, Bunnell HT, Hossain MJ, Wysocki T, Lima JJ, Finkel TH, et al. Being overweight or obese and the development of asthma. Pediatrics. 2018;142(6):e20182119. 10.1542/peds.2018-2119 30478238

[6] Lindberg L, Danielsson P, Persson M, Marcus C, Hagman E (2020). Association of childhood obesity with risk of early all-cause and cause-specific mortality: a Swedish prospective cohort study. PLoS Med.

[7] Reilly JJ, Kelly J (2011). Long-term impact of overweight and obesity in childhood and adolescence on morbidity and premature mortality in adulthood: systematic review. Int J Obes.

[8] Zheng M, Lamb KE, Grimes C, Laws R, Bolton K, Ong KK (2018). Rapid weight gain during infancy and subsequent adiposity: a systematic review and meta-analysis of evidence. Obes Rev.

[9] Li YF, Lin SJ, Chiang TL (2020). Timing of rapid weight gain and its effect on subsequent overweight or obesity in childhood: findings from a longitudinal birth cohort study. BMC Pediatr.

[10] Lu Y, Pearce A, Li L (2020). Weight gain in early years and subsequent body mass index trajectories across birth weight groups: a prospective longitudinal study. Eur J Pub Health.

[11] Barker DJ (2007). The origins of the developmental origins theory. J Intern Med.

[12] Dulloo AG, Jacquet J, Seydoux J, Montani JP (2006). The thrifty ‘catch-up fat’ phenotype: its impact on insulin sensitivity during growth trajectories to obesity and metabolic syndrome. Int J Obes.

[13] Waterland RA, Michels KB (2007). Epigenetic epidemiology of the developmental origins hypothesis. Annu Rev Nutr.

[14] Küpers LK, Monnereau C, Sharp GC, Yousefi P, Salas LA, Ghantous A (2019). Meta-analysis of epigenome-wide association studies in neonates reveals widespread differential DNA methylation associated with birthweight. Nat Commun.

[15] Vehmeijer FOL, Küpers LK, Sharp GC, Salas LA, Lent S, Jima DD (2020). DNA methylation and body mass index from birth to adolescence: meta-analyses of epigenome-wide association studies. Genome Med.

[16] Bouwland-Both MI, van Mil NH, Stolk L, Eilers PH, Verbiest MM, Heijmans BT (2013). DNA methylation of IGF2DMR and H19 is associated with fetal and infant growth: the generation R study. PLoS One.

[17] Groom A, Potter C, Swan DC, Fatemifar G, Evans DM, Ring SM (2012). Postnatal growth and DNA methylation are associated with differential gene expression of the TACSTD2 gene and childhood fat mass. Diabetes..

[18] Prats-Puig A, Carreras-Badosa G, Bassols J, Cavelier P, Magret A, Sabench C (2017). The placental imprinted DLK1-DIO3 domain: a new link to prenatal and postnatal growth in humans. Am J Obstet Gynecol.

[19] Bright HD, Howe LD, Khouja JN, Simpkin AJ, Suderman M, O’Keeffe LM (2019). Epigenetic gestational age and trajectories of weight and height during childhood: a prospective cohort study. Clin Epigenetics.

[20] Quilter CR, Cooper WN, Cliffe KM, Skinner BM, Prentice PM, Nelson L (2014). Impact on offspring methylation patterns of maternal gestational diabetes mellitus and intrauterine growth restraint suggest common genes and pathways linked to subsequent type 2 diabetes risk. FASEB J.

[21] Van Den Berg G, Van Eijsden M, Galindo-Garre F, Vrijkotte T, Gemke R (2013). Low maternal education is associated with increased growth velocity in the first year of life and in early childhood: the ABCD study. Eur J Pediatr.

[22] Yu SH, Mason J, Crum J, Cappa C, Hotchkiss DR (2016). Differential effects of young maternal age on child growth. Glob Health Action.

[23] Zheng W, Suzuki K, Shinohara R, Sato M, Yokomichi H, Yamagata Z (2015). Maternal smoking during pregnancy and growth in infancy: a covariance structure analysis. J Epidemiol.

[24] Zheng M, Bowe SJ, Hesketh KD, Bolton K, Laws R, Kremer P (2019). Relative effects of postnatal rapid growth and maternal factors on early childhood growth trajectories. Paediatr Perinat Epidemiol.

[25] Gaillard R, Rurangirwa AA, Williams MA, Hofman A, Mackenbach JP, Franco OH (2014). Maternal parity, fetal and childhood growth, and cardiometabolic risk factors. Hypertension..

[26] Regnault N, Botton J, Forhan A, Hankard R, Thiebaugeorges O, Hillier TA (2010). Determinants of early ponderal and statural growth in full-term infants in the EDEN mother-child cohort study. Am J Clin Nutr.

[27] Fraser A, Macdonald-Wallis C, Tilling K, Boyd A, Golding J, Davey Smith G (2013). Cohort profile: the Avon Longitudinal Study of Parents and Children: ALSPAC mothers cohort. Int J Epidemiol.

[28] Janssen BG, Madlhoum N, Gyselaers W, Bijnens E, Clemente DB, Cox B (2017). Cohort profile: the ENVIRonmental influence ON early AGEing (ENVIRONAGE): a birth cohort study. Int J Epidemiol.

[29] Kana MA, Rodrigues C, Fonseca MJ, Santos AC, Barros H (2018). Effect of maternal country of birth on breastfeeding practices: results from Portuguese GXXI birth cohort. Int Breastfeed J.

[30] Guxens M, Ballester F, Espada M, Fernandez MF, Grimalt JO, Ibarluzea J (2012). Cohort profile: the INMA--INfancia y Medio Ambiente--(Environment and Childhood) Project. Int J Epidemiol.

[31] Farchi S, Forastiere F, Vecchi Brumatti L, Alviti S, Arnofi A, Bernardini T (2014). Piccolipiu, a multicenter birth cohort in Italy: protocol of the study. BMC Pediatr.

[32] Chatzi L, Leventakou V, Vafeiadi M, Koutra K, Roumeliotaki T, Chalkiadaki G (2017). Cohort profile: the mother-child cohort in Crete, Greece (Rhea study). Int J Epidemiol.

[33] Chatzi L, Plana E, Daraki V, Karakosta P, Alegkakis D, Tsatsanis C (2009). Metabolic syndrome in early pregnancy and risk of preterm birth. Am J Epidemiol.

[34] Vineis P, Chadeau-Hyam M, Gmuender H, Gulliver J, Herceg Z, Kleinjans J (2017). The exposome in practice: design of the EXPOsOMICS project. Int J Hyg Environ Health.

[35] Relton CL, Gaunt T, McArdle W, Ho K, Duggirala A, Shihab H (2015). Data resource profile: Accessible Resource for Integrated Epigenomic Studies (ARIES). Int J Epidemiol.

[36] Aryee MJ, Jaffe AE, Corrada-Bravo H, Ladd-Acosta C, Feinberg AP, Hansen KD (2014). Minfi: a flexible and comprehensive Bioconductor package for the analysis of Infinium DNA methylation microarrays. Bioinformatics (Oxford, England).

[37] Fortin J-P, Labbe A, Lemire M, Zanke BW, Hudson TJ, Fertig EJ (2014). Functional normalization of 450k methylation array data improves replication in large cancer studies. Genome Biol.

[38] Lehne B, Drong AW, Loh M, Zhang W, Scott WR, Tan S-T (2015). A coherent approach for analysis of the Illumina HumanMethylation450 BeadChip improves data quality and performance in epigenome-wide association studies. Genome Biol.

[39] Alfano R, Chadeau-Hyam M, Ghantous A, Keski-Rahkonen P, Chatzi L, Perez AE (2020). A multi-omic analysis of birthweight in newborn cord blood reveals new underlying mechanisms related to cholesterol metabolism. Metabolism..

[40] Alfano R, Chadeau-Hyam M, Ghantous A, Keski-Rahkonen P, Chatzi L, Perez AE (2020). A cord blood multi-omic analysis of birthweight reveals new underlying mechanisms related to cholesterol metabolism.

[41] Gervin K, Page CM, Aass HC, Jansen MA, Fjeldstad HE, Andreassen BK (2016). Cell type specific DNA methylation in cord blood: A 450K-reference data set and cell count-based validation of estimated cell type composition. Epigenetics..

[42] Bakulski KM, Feinberg JI, Andrews SV, Yang J, Brown S (2016). DNA methylation of cord blood cell types: applications for mixed cell birth studies. Epigenetics..

[43] Bohlin J, Håberg SE, Magnus P, Reese SE, Gjessing HK, Magnus MC (2016). Prediction of gestational age based on genome-wide differentially methylated regions. Genome Biol.

[44] Knight AK, Craig JM, Theda C, Bækvad-Hansen M, Bybjerg-Grauholm J, Hansen CS (2016). An epigenetic clock for gestational age at birth based on blood methylation data. Genome Biol.

[45] Pelegí-Sisó D, de Prado P, Ronkainen J, Bustamante M, González JR (2020). methylclock: a Bioconductor package to estimate DNA methylation age. Bioinformatics..

[46] Handakas E, Keski-Rahkonen P, Chatzi L, Alfano R, Roumeliotaki T, Plusquin M (2021). Cord blood metabolic signatures predictive of childhood overweight and rapid growth. Int J Obes.

[47] Ong KK, Ahmed ML, Emmett PM, Preece MA, Dunger DB (2000). Association between postnatal catch-up growth and obesity in childhood: prospective cohort study. Bmj..

[48] Mueller NT, Zhang M, Hoyo C, Østbye T, Benjamin-Neelon SE (2019). Does cesarean delivery impact infant weight gain and adiposity over the first year of life?. Int J Obes.

[49] Manerkar K, Harding J, Conlon C, McKinlay C (2020). Maternal gestational diabetes and infant feeding, nutrition and growth: a systematic review and meta-analysis. Br J Nutr.

[50] Andrea SB, Hooker ER, Messer LC, Tandy T, Boone-Heinonen J (2017). Does the association between early life growth and later obesity differ by race/ethnicity or socioeconomic status? A systematic review. Ann Epidemiol.

[51] Alfano R, Chadeau-Hyam M, Ghantous A, Keski-Rahkonen P, Chatzi L, Perez AE (2020). A cord blood multi-omic analysis of birthweight reveals new underlying mechanisms related to cholesterol metabolism [gene expression].

[52] Robinson O, Keski-Rahkonen P, Chatzi L, Kogevinas M, Nawrot T, Pizzi C (2018). Cord blood metabolic signatures of birth weight: a population-based study. J Proteome Res.

[53] Vineis P, Alfano R, Chadeau-Hyam M, Keski-Rahkonen P, Robinot N, Scalbert A, et al. A multi-omic analysis of birthweight in newborn cord blood reveals new underlying mechanisms related to cholesterol metabolism. MetaboLights. https://www.ebi.ac.uk/metabolights/MTBLS1684/descriptors. 2020. 10.1016/j.metabol.2020.154292 PMC7450273 32553738

[54] WHO Child Growth Standards (2006). Length/height-for-age, weight-for-age, weight-for-length, weight-for-height and body mass index-for-age: methods and development.

[55] van Iterson M, van Zwet EW, Heijmans BT (2017). Controlling bias and inflation in epigenome- and transcriptome-wide association studies using the empirical null distribution. Genome Biol.

[56] Pedersen BS, Schwartz DA, Yang IV, Kechris KJ (2012). Comb-p: software for combining, analyzing, grouping and correcting spatially correlated P-values. Bioinformatics (Oxford, England).

[57] Peters TJ, Buckley MJ, Statham AL, Pidsley R, Samaras K, Lord V (2015). De novo identification of differentially methylated regions in the human genome. Epigenetics Chromatin.

[58] Vickers AJ, Elkin EB (2006). Decision curve analysis: a novel method for evaluating prediction models. Med Decis Mak.

[59] Stijn V, Maarten B, Theis L (2012). Imputation strategies for the estimation of natural direct and indirect effects. Epidemiol Methods.

[60] Alfano R, Robinson O, Handakas E, Nawrot TS, Vineis P, Plusquin M (2021). Perspectives and challenges of epigenetic determinants of childhood obesity: a systematic review. Obes Rev.

[61] Engel SM, Joubert BR, Wu MC, Olshan AF, Håberg SE, Ueland PM (2014). Neonatal genome-wide methylation patterns in relation to birth weight in the Norwegian mother and child cohort. Am J Epidemiol.

[62] Simpkin AJ, Suderman M, Gaunt TR, Lyttleton O, McArdle WL, Ring SM (2015). Longitudinal analysis of DNA methylation associated with birth weight and gestational age. Hum Mol Genet.

[63] Joubert BR, Felix JF, Yousefi P, Bakulski KM, Just AC, Breton C (2016). DNA methylation in newborns and maternal smoking in pregnancy: genome-wide consortium meta-analysis. Am J Hum Genet.

[64] Lango Allen H, Estrada K, Lettre G, Berndt SI, Weedon MN, Rivadeneira F (2010). Hundreds of variants clustered in genomic loci and biological pathways affect human height. Nature..

[65] Iatan I, Dastani Z, Do R, Weissglas-Volkov D, Ruel I, Lee JC (2009). Genetic variation at the proprotein convertase subtilisin/kexin type 5 gene modulates high-density lipoprotein cholesterol levels. Circ Cardiovasc Genet.

[66] Claussnitzer M, Dankel SN, Kim KH, Quon G, Meuleman W, Haugen C (2015). FTO obesity variant circuitry and adipocyte browning in humans. N Engl J Med.

[67] Pei H, Yao Y, Yang Y, Liao K, Wu JR (2011). Krüppel-like factor KLF9 regulates PPARγ transactivation at the middle stage of adipogenesis. Cell Death Differ.

[68] Okada Y, Kubo M, Ohmiya H, Takahashi A, Kumasaka N, Hosono N (2012). Common variants at CDKAL1 and KLF9 are associated with body mass index in east Asian populations. Nat Genet.

[69] Reed ZE, Suderman MJ, Relton CL, Davis OSP, Hemani G (2020). The association of DNA methylation with body mass index: distinguishing between predictors and biomarkers. Clin Epigenetics.

[70] Sun D, Zhang T, Su S, Hao G, Chen T, Li QZ (2019). Body mass index drives changes in DNA methylation: a longitudinal study. Circ Res.

[71] Han L, Zhang H, Kaushal A, Rezwan FI, Kadalayil L, Karmaus W (2019). Changes in DNA methylation from pre- to post-adolescence are associated with pubertal exposures. Clin Epigenetics.

[72] Zhuravleva E, Gut H, Hynx D, Marcellin D, Bleck CK, Genoud C (2012). Acyl coenzyme A thioesterase Them5/Acot15 is involved in cardiolipin remodeling and fatty liver development. Mol Cell Biol.

[73] Beard S, Pritchard N, Binder N, Schindler K, De Alwis N, Kaitu'u-Lino TJ (2020). Aurora kinase mRNA expression is reduced with increasing gestational age and in severe early onset fetal growth restriction. Placenta..

[74] Kazmi N, Sharp GC, Reese SE, Vehmeijer FO, Lahti J, Page CM (2019). Hypertensive disorders of pregnancy and DNA methylation in newborns. Hypertension..

[75] van Dijk SJ, Peters TJ, Buckley M, Zhou J, Jones PA, Gibson RA (2018). DNA methylation in blood from neonatal screening cards and the association with BMI and insulin sensitivity in early childhood. Int J Obes.

[76] Cohen P, Levy JD, Zhang Y, Frontini A, Kolodin DP, Svensson KJ (2014). Ablation of PRDM16 and beige adipose causes metabolic dysfunction and a subcutaneous to visceral fat switch. Cell..

[77] Fradin D, Boëlle PY, Belot MP, Lachaux F, Tost J, Besse C (2017). Genome-wide methylation analysis identifies specific epigenetic marks in severely obese children. Sci Rep.

[78] Liu L, Chen Y, Chen J, Lu M, Guo R, Han J (2021). The relationship between PRDM16 promoter methylation in abdominal subcutaneous and omental adipose tissue and obesity. Clin Nutr.

[79] Kennedy EM, Goehring GN, Nichols MH, Robins C, Mehta D, Klengel T (2018). An integrated -omics analysis of the epigenetic landscape of gene expression in human blood cells. BMC Genomics.

[80] Lancaster EE, Vladimirov VI, Riley BP, Landry JW, Roberson-Nay R, York TP. Large-scale integration of DNA methylation and gene expression array platforms identifies both cis and trans relationships. Epigenetics. 2022;17(12):1753–4255. 10.1080/15592294.2022.2079293 PMC9621057 35608069

[81] Prats-Puig A, Xargay-Torrent S, Carreras-Badosa G, Mas-Parés B, Bassols J, Petry CJ (2020). Methylation of the C19MC microRNA locus in the placenta: association with maternal and chilhood body size. Int J Obes.

[82] Khouja JN, Simpkin AJ, O'Keeffe LM, Wade KH, Houtepen LC, Relton CL (2018). Epigenetic gestational age acceleration: a prospective cohort study investigating associations with familial, sociodemographic and birth characteristics. Clin Epigenetics.

[83] Javed R, Chen W, Lin F, Liang H (2016). Infant’s DNA methylation age at birth and epigenetic aging accelerators. Biomed Res Int.

[84] Gervin K, Salas LA, Bakulski KM, van Zelm MC, Koestler DC, Wiencke JK (2019). Systematic evaluation and validation of reference and library selection methods for deconvolution of cord blood DNA methylation data. Clin Epigenetics.

[85] Luo Y, Lu X, Xie H (2014). Dynamic Alu methylation during normal development, aging, and tumorigenesis. Biomed Res Int.

[86] Blum MGB, Valeri L, François O, Cadiou S, Siroux V, Lepeule J (2020). Challenges raised by mediation analysis in a high-dimension setting. Environ Health Perspect.

