# Supplementary material for: Cord blood epigenome-wide meta-analysis in six European-based child cohorts identifies signatures linked to rapid weight growth
Source: BMC Med. 2023 Jan 11;21:17. doi: 10.1186/s12916-022-02685-7 (PMC9831885; doi:10.1186/s12916-022-02685-7)
Supplement: Supplementary file 1 — Additional file 1. Supplementary methods [27–38]. [file 12916_2022_2685_MOESM1_ESM.docx]

Additional File 1: Supplementary methods to

**Cord blood epigenome-wide meta-analysis in six European-based child cohorts identifies signatures linked to rapid weight growth**

Alfano R et al

**Table of Contents**

[Supplementary methods 2](#_Toc120521593)

[Cohort specific description 2](#_Toc120521594)

[ALSPAC 2](#_Toc120521595)

[ENVIR*ON*AGE 3](#_Toc120521596)

[EXPOsOMICS 4](#_Toc120521597)

[Generation XXI 6](#_Toc120521598)

[DNA methylation cohort specific description 7](#_Toc120521599)

[ALSPAC 7](#_Toc120521600)

[ENVIR*ON*AGE 8](#_Toc120521601)

[EXPOsOMICS 8](#_Toc120521602)

[Generation XXI 9](#_Toc120521603)

[Cohort specific acknowledgment 10](#_Toc120521604)

[ALSPAC 10](#_Toc120521605)

[ENVIR*ON*AGE 10](#_Toc120521606)

[EXPOsOMICS 10](#_Toc120521607)

[Generation XXI 11](#_Toc120521608)

[References 12](#_Toc120521609)

# Supplementary methods

## Cohort specific description

## ALSPAC

ALSPAC is a prospective birth cohort study of 14,541 mother-child pairs recruited between April 1991 and December 1992 in Avon, UK[1]. Ethical approval for the study was obtained from the ALSPAC Ethics and Law Committee and the Local Research Ethics Committees, and informed consent was obtained from all subjects. The ALSPAC study website contains details of all the data that is available through a fully searchable data dictionary (http://www.bris.ac.uk/alspac/researchers/data-access/data-dictionary/). ALSPAC’s eligible sample is defined as all pregnant women living in and around the city of Bristol (south-west UK) and due to deliver between April 1991 and December 1992.

Information from mothers and children was collected from questionnaires or medical data during the recruitment at birth and the follow-up in childhood. Maternal smoking status during pregnancy was dichotomized into smokers or non-smokers according to the self-reported declarations from 18-week antenatal and 8-week postnatal questionnaires; maternal education was collected from a self-reported questionnaire at 32 weeks of gestation, and was coded in three categories according to educational achievement: (i) low: Certificate of Secondary Education (CSE), Vocational or Ordinary- (O-) level, educational qualifications generally obtained at 16 years of age; (ii) intermediate: Advanced- (A-) level, subject-specific qualification most commonly attained at 18 years of age and required for admission to higher education; (iii) high: university degree and above; sex of the newborns was collected from birth notifications; pre-pregnancy BMI (at 12 weeks of gestation) was calculated from the self-reported pre-pregnancy weight (in kilograms) divided by the square of the height (in meters); maternal age at delivery was collected from a self-reported postnatal (8-week) questionnaire; parity (dichotomized into having never or at least once been pregnant before the current pregnancy) was self-reported at 18 weeks of gestation; gestational age (based on last menstrual period based when the mother was certain, or, if not, on ultrasound assessment) was coded by the number of weeks at delivery; birth weight (in grams) was assessed using obstetric data, birth notification data and birth weight as recorded by the ALSPAC measurers and retaining the lowest measure, except in the case of disagreement between the sources by more than 100 grams in which case the data was omitted. Birth weight was extracted from routine hospital birth records, and children weight and height later in life were measured by trained staff during clinical visits during childhood. Maternal pregnancy diabetes was categorized into present or not based on clinical diagnosis of gestational diabetes; deliver mode was coded into having a caesarean section or not. No information was available about European ethnicity, but ethnicity was categorized into white or non-white. Population participating in this study encompasses children of the ALSPAC cohort part of the Accessible Resource for Integrated Epigenomics Studies (ARIES) project[2], providing a DNA sample extracted from cord blood drawn from the umbilical cord upon delivery, for a total of 914 participants.

## ENVIR*ON*AGE

ENVIR*ON*AGE is an ongoing population-based prospective study of more than 2,000 mother-child pairs recruited from 2010 onward upon arrival for delivery at the East-Limburg Hospital in Genk, Flanders, Belgium [3]. The study was approved by the Ethical Committee of Hasselt University and the East-Limburg Hospital, and informed consent was obtained by the mothers. Inclusion criteria were: delivery without planned caesarean section and ability to fill out a Dutch language questionnaire. During the recruitment at birth, information on maternal tobacco smoke during the pregnancy (coded as smoker or non-smoker), maternal education (coded as low if mothers had a no diploma or primary school diploma; medium if mothers had secondary school diploma; and high: degree or higher education classification), maternal pre-pregnancy BMI, maternal age at delivery, parity (coded as nulliparous or multiparous) were collected via questionnaires. Child gestational age was determined from ultrasound estimation, sex and birthweight were obtained from obstetric records. Child weight and height later in life were self-reported by parents or measured by trained staff at the 4-year follow-up. Maternal pregnancy diabetes was categorized into present or not based on medical information from obstetric data or if self-reported by the mothers; deliver mode was coded into having a caesarean section or not based on obstetric data; child ethnicity was defined based on based on self-reported grandparent origins in European or non-European (including India, Algeria, Suriname). Before the placenta was delivered, whole blood was withdrawn from cord vessels and processed into buffy coat and plasma (via EDTA tubes) and immediately frozen at -80 °C. To retrieve RNA, samples were collected via PAXgene tubes (QIAGEN Benelux B.V., Antwerp, Belgium). The population participating in this study encompasses children for which cord blood DNA methylation was available, including 377 neonates, 22 of which participated in the EXPOsOMICS project and were hence excluded from the ENVIR*ON*AGE samples.

## EXPOsOMICS

EXPOsOMICS is a European Union-funded project which includes multiple mother-child cohorts[4], 4 of which took part in the present study: ENVIR*ON*AGE, INMA, Piccolipiù and Rhea. EXPOsOMICS population participating in this study encompasses 499 children for which cord blood DNA methylation (200, 99, 99 and 101 neonates of the ENVIR*ON*AGE, INMA, Piccolipiù and Rhea cohorts, respectively). Within each cohort, data on maternal tobacco smoke during the pregnancy (coded as smoker or non-smoker), maternal education (coded as low if mothers had a no diploma or primary school diploma; medium if mothers had secondary school diploma; and high: degree or higher education classification), maternal pre-pregnancy BMI, maternal age at delivery, and parity (coded as nulliparous or multiparous) was collected from questionnaires at birth, and child gestational age (determined from ultrasound estimations in ENVIR*ON*AGE, from last menstrual period and confirmed by ultrasound, or if conflicting results, from last menstrual period estimated based on crown-rump length in INMA, from last menstrual period in Piccolipiù, from the last menstrual period and if missing from ultrasound estimations in Rhea), and sex were collected from clinical medical records. Birthweight was obtained from obstetric records, and child weight and height later in life were self-reported by parents or measured by trained staff during the follow-ups in childhood. Maternal pregnancy diabetes was categorized into present or not; deliver mode was coded into having a caesarean section or not; child ethnicity was available in ENVIR*ON*AGE cohort while no information was available the other cohorts. As previously described, in ENVIR*ON*AGE, ethnicity was defined on based on self-reported grandparent origins and coded as European or non-European, however no child was classified as non-European in ENVIR*ON*AGE EXPOsOMICS subset for this study.

While ENVIR*ON*AGE cohort has been described in the previous paragraph, a brief description of the remaining cohorts is reported below.

INMA is a multicentric Spanish population-based birth cohort including 3,768 mother-child pairs enrolled between 1996 and 2008 during the first pregnancy trimester visits to different primary health care centres in Spain[5]. The study was approved by the ethics committee of the Hospital del Mar Medical Research Institute and conducted according to the principles of the Helsinki Declaration. Mothers provided written informed consent. A set of 99 children from two health care centres in Sabadell is part of the EXPOsOMICS children project and has available DNA methylome data that have been used in the present study.

Piccolipiù is a multicentric Italian birth cohort in which 3,358 mother-child pairs were recruited in 5 Italian centers (Turin, Trieste, Florence, Viareggio and Rome) between 2011 and 2015[6]. Ethical approvals have been obtained from the Ethics Committees of the Local Health Unit Roma E (management centre), of the Istituto Superiore di Sanità (National Institute of Public Health) and of each local centre. Parents provided written informed consent. A set of 99 children from the Turin centre of Piccolipiù is part of the EXPOsOMICS children project and has available DNA methylome data that have been used in the present study.

The Rhea study is a prospective mother-child cohort including 1,458 mother-child pairs enrolled between 2007 and 2008during the pregnancy visits or at the delivery in Heraklion (Greece)[7, 8]. The Ethics Committee of the University Hospital at Heraklion approved the study protocols and written informed consent was obtained from the mothers. A set of 101 children from the Rhea cohort is part of the EXPOsOMICS children project and has available DNA methylome data that have been used in the present study.

## Generation XXI

Generation XXI (GXXI) is a population-based prospective birth cohort of 8,647 mother-child enrolled between April 2005 and August 2006 from all five public maternity units of the metropolitan area of Porto, Portugal[9]. Ethical approval for the project was received by the Ethical Committee of São João Hospital/ University of Porto Medical School and complied with the Helsinki Declaration and the current national legislation. The Portuguese Data Protection Authority also approved the study. Written informed consent was obtained from all parents or legal guardians. Population participating to this study encompasses children for which cord blood DNA methylation was available 751 neonates of the GXXI cohort.

At baseline, face-to-face interviews were conducted during the hospital stay by trained interviewers, 24–72 hours after delivery. Data on socio-demographic characteristics, including maternal smoking during pregnancy, maternal education, maternal pre-pregnancy anthropometrics, and age at delivery were collected. Information concerning the delivery (parity) and the newborn, including birthweight and gestational age, was retrieved from clinical records by the same interviewers. Gestational age was derived by ultrasound measurements (if performed up to the 20th gestational week) and, if no such data was available it was based on last menstrual period. In all subsequent GXXI follow-up evaluations, mothers were asked to bring their Child Health Book, in which every anthropometric measurement performed on the child as part of standard childcare in Portugal was recorded by health professionals. Self-reported maternal pregnancy diabetes was categorized into present or not based on medical records and baseline questionnaires administered to the mothers; delivery mode was coded into having a caesarean section or not; child ethnicity was defined based on based on maternal origin and coded as European or non-European (including Angola, Mozambique, Sao Tome and Principe, Canada, United States of America, Brazil, Chile and Cape Verde).

## DNA methylation cohort specific description

Methylation was measured using the Infinium HumanMethylation450 BeadChip in ARIES and EXPOsOMICS, and using Infinium MethylationEPIC BeadChip, except in ENVIR*ON*AGE and GXXI studies. After cohort-specific quality control and preprocessing, 861 samples were available for participation in this study in ARIES, 350 in ENVIR*ON*AGE, 472 in EXPOsOMICS (197 samples in ENVIRONAGE, 85 in INMA, 98 in Piccolipiù, 92 in Rhea), and 732 in GXXI, respectively. For each cohort probes with sample call rate lower than 80%, cross-reactive probes and probes located on sex chromosomes were removed, leaving 433,702 probes in ARIES, 797,220 in ENVIRONAGE, 420,159 in EXPOsOMICS, and 778,453 in GXXI. Before conducting the EWASs, DNA methylation were trimmed using the Tukey method if removal of outlier was not performed yet by cohort specific preprocessing. A brief description of methods for DNA methylation analyses for each of the cohort is reported below.

## ALSPAC

For the ARIES subset, DNA was extracted from whole blood or buffy coat according to the standard protocol and subjected to bisulfite conversion using the Zymo EZ-96 DNA MethylationTM kit (Zymo, Irvine, CA). Whole genome amplification hybridization was performed using the Illumina HumanMethylation450 BeadChip. Arrays were scanned using an Illumina iScan, with initial quality review using GenomeStudio. Samples from different time points were distributed across slides in a semi-random approach to minimise the potential relationship between batch effects and other variables as previously described[2]. During the data acquisition, a wide range of technical covariates were collected in a purpose-built laboratory information management system (LIMS). The LIMS also reported quality control (QC) metrics from the standard control probes on the 450k BeadChip[2]. Samples failing quality control (average probe detection p-value ≥0.01) were excluded from further analyses and scheduled for repeat assay. Methylation levels were measured by the β values calculated as the ratios of intensities arising from methylated probes over those arising from the sum of methylated and unmethylated probes. ARIES data were preprocessed in R, with the meffil package, trimmed, and a total of 482,454 CpG loci were finally retained for subsequent analyses.

## ENVIR*ON*AGE

In a subset of 377 children from the ENVIRONAGE cohort cord blood DNA was extracted from buffy coats according to standard protocol. DNA concentration was measured using the Quant-IT assay from Thermo Fisher. Gel-electrophoresis was performed to assess the integrity of DNA samples. Methylation data was produced at GenomeScan in Netherland. Bisulphite conversion using 100-500 ng genomic DNA inputs was performed using the EZ DNA Methylation Gold kit (Zymo Research, Irvine, CA, USA). The converted samples (4µl) were amplified and hybridized on the Illumina HumanMethylation 850K BeadChip arrays and scanned using an Illumina iScan. Data quality was assessed using the R script MethylAid using analysis default settings and 1 sample was removed because of low quality (sample call rate <99%). DNA methylation data were preprocessed using the minfi package in R[10]. Briefly, 4 samples were removed because of wrongly predicted sex using shinyMethyl[11], the data were normalized using functional normalization, measurements with detection p-values >10e-16[12] were set to missing leading to removal of 7,961 probes with a call rate <95%. No additional sample had a call rate <98%. For each CpG site methylation levels were expressed as beta values calculated as the ratios of intensities arising from methylated probes over those arising from the sum of methylated and unmethylated probes. No probe filtering was applied, leaving 857,898 CpGs available measured for 372 samples of the subsequent analysis. Among these samples, 22 were already included in the EXPOsOMICS study and hence were excluded from this ENVIR*ON*AGE subset in analyses in which both cohorts are participating.

## EXPOsOMICS

DNA methylation levels of EXPOsOMICS were measured at the International Agency for Research on Cancer of Lyon in France (ENVIR*ON*AGE, Rhea, Piccolipiu) and at ̀ the Genome Analysis Facility of the University Medical Center Groningen, The Netherlands (INMA). DNA was extracted from buffy coats according to standard protocol, followed by bisulphite conversion using the Zymo EZ DNA methylationTM kit (Zymo, Irvine, CA, USA), hybridization to Illumina HumanMethylation 450K BeadChip arrays and scanning using an Illumina iScan. Data quality was initially reviewed using GenomeStudio. An in-house software written for the R statistical computing environment was used to preprocess the data as follows: removal of probes based on signal intensities and control probes, background subtraction and dye bias correction (for probes using the Infinium II design). Additionally, data were trimmed removing the outliers using Turkey method. After removals of samples failed facility control and with mismatched sex, 472 samples were left available for the analysis. For each CpG site methylation levels were expressed as beta values calculated as the ratios of intensities arising from methylated probes over those arising from the sum of methylated and unmethylated probes. No filtering was applied, leaving 485,577 CpGs available for the cohort specific analysis.

## Generation XXI

DNA methylation levels in Generation XXI cohort were measured in 751 samples using Illumina HumanMethylation 850K BeadChip arrays. Raw fluorescence intensities data were extracted from ‘idat’ files using the ‘minfi’ package in R statistical environment[10]. Data pre-processing was carried out using in-house software written for the R statistical computing environment. For each sample and each probe, measurements were set to missing if obtained by averaging intensities over less than three beads, or if averaged intensities were below detection thresholds estimated from negative control probes. Pre-processing procedure also include back-ground subtraction, colour bias adjustment, and fluorescence intensities normalization. For each CpG site methylation levels were expressed as beta values. Samples failed facility control and with mismatched sex were excluded. Probes and samples were excluded if the call rate was lower than 95%, leaving a total of 867,867 probes for 732 samples for subsequent analyses.

## Cohort specific acknowledgment

## ALSPAC

We are extremely grateful to all the families who took part in this study, the midwives for their help in recruiting them, and the whole ALSPAC team, which includes interviewers, computer and laboratory technicians, clerical workers, research scientists, volunteers, managers, receptionists, and nurses. We would like to acknowledge Tom Gaunt, Oliver Lyttleton, Sue Ring, Nabila Kazmi, and Geoff Woodward for their earlier contributions to the generation of ARIES data (ALSPAC methylation data).

## ENVIR*ON*AGE

We are extremely grateful to the participating women and neonates, as well as the staff of the maternity ward, midwives and the staff of the clinical laboratory of East-Limburg Hospital in Genk.

## EXPOsOMICS

ENVIR*ON*AGE: ENVIR*ON*AGE researchers are extremely grateful to the participating women and neonates, as well as the staff of the maternity ward, midwives and the staff of the clinical laboratory of East-Limburg Hospital in Genk.

INMA: INMA researchers would like to thank all the participants for their generous collaboration. A full roster of the INMA Project Investigators can be found at http://www.proyectoinma.org/presentacioninma/listado-investigadores/en_listado-investigadores.html.

Piccolipiù: Our thanks go to all the families who took part in this study, to the midwives for their help in recruiting them, and to the whole PICCOLIPIU’ team, which includes doctors, nurses, research scientists and computer/laboratory technicians.

RHEA: We are extremely grateful to all the families who took part in the Rhea study, the midwives, research assistants and psychologists for recruiting and following them, and the whole Rhea team, including research scientists, biologists and technicians for their commitment and their role in the success of the study. We thank Mariona Bustamante (CREAL, Barcelona, Spain) for maintaining the Rhea DNA biobank.

## Generation XXI

We gratefully acknowledge the families enrolled in Generation XXI for their kindness, all members of the research team for their enthusiasm and perseverance and the participating hospitals and their staff for their help and support.

## References

1. Fraser A, Macdonald-Wallis C, Tilling K, Boyd A, Golding J, Davey Smith G, et al. Cohort Profile: the Avon Longitudinal Study of Parents and Children: ALSPAC mothers cohort. Int J Epidemiol. 2013;42(1):97-110.

2. Relton CL, Gaunt T, McArdle W, Ho K, Duggirala A, Shihab H, et al. Data Resource Profile: Accessible Resource for Integrated Epigenomic Studies (ARIES). Int J Epidemiol. 2015;44(4):1181-90.

3. Janssen BG, Madlhoum N, Gyselaers W, Bijnens E, Clemente DB, Cox B, et al. Cohort Profile: The ENVIRonmental influence ON early AGEing (ENVIRONAGE): a birth cohort study. Int J Epidemiol. 2017;46(5):1386-7m.

4. Vineis P, Chadeau-Hyam M, Gmuender H, Gulliver J, Herceg Z, Kleinjans J, et al. The exposome in practice: Design of the EXPOsOMICS project. Int J Hyg Environ Health. 2017;220(2 Pt A):142-51.

5. Guxens M, Ballester F, Espada M, Fernandez MF, Grimalt JO, Ibarluzea J, et al. Cohort Profile: the INMA--INfancia y Medio Ambiente--(Environment and Childhood) Project. Int J Epidemiol. 2012;41(4):930-40.

6. Farchi S, Forastiere F, Vecchi Brumatti L, Alviti S, Arnofi A, Bernardini T, et al. Piccolipiu, a multicenter birth cohort in Italy: protocol of the study. BMC Pediatr. 2014;14:36.

7. Chatzi L, Leventakou V, Vafeiadi M, Koutra K, Roumeliotaki T, Chalkiadaki G, et al. Cohort Profile: The Mother-Child Cohort in Crete, Greece (Rhea Study). Int J Epidemiol. 2017;46(5):1392-3k.

8. Chatzi L, Plana E, Daraki V, Karakosta P, Alegkakis D, Tsatsanis C, et al. Metabolic syndrome in early pregnancy and risk of preterm birth. Am J Epidemiol. 2009;170(7):829-36.

9. Kana MA, Rodrigues C, Fonseca MJ, Santos AC, Barros H. Effect of maternal country of birth on breastfeeding practices: results from Portuguese GXXI birth cohort. Int Breastfeed J. 2018;13:15.

10. Aryee MJ, Jaffe AE, Corrada-Bravo H, Ladd-Acosta C, Feinberg AP, Hansen KD, et al. Minfi: a flexible and comprehensive Bioconductor package for the analysis of Infinium DNA methylation microarrays. Bioinformatics (Oxford, England). 2014;30(10):1363-9.

11. Fortin J-P, Labbe A, Lemire M, Zanke BW, Hudson TJ, Fertig EJ, et al. Functional normalization of 450k methylation array data improves replication in large cancer studies. Genome Biol. 2014;15(11):503.

12. Lehne B, Drong AW, Loh M, Zhang W, Scott WR, Tan S-T, et al. A coherent approach for analysis of the Illumina HumanMethylation450 BeadChip improves data quality and performance in epigenome-wide association studies. Genome Biol. 2015;16(1):37.
