# Supplementary material for: Cord blood epigenome-wide meta-analysis in six European-based child cohorts identifies signatures linked to rapid weight growth
Source: BMC Med. 2023 Jan 11;21:17. doi: 10.1186/s12916-022-02685-7 (PMC9831885; doi:10.1186/s12916-022-02685-7)
Supplement: Supplementary file 2 — Additional file 2: Table S1. Comparison of characteristics of the ALSPAC, ENVIRONAGE, EXPOsOMICS and GXXI cohorts by rapid weight growth status. Table S2. 49 CpGs associated with rapid weight growth in the meta-analysis of EWASs with PSuggestive <1e-05. Table S3. Results from sensitivity analyses adding birthweight and delivery mode, removing cell types from confounders, excluding mothers with gestational diabetes and non-white European children, for the CpGs with PSuggestive <1e-05 in the meta-analysis of EWASs of rapid weight growth. Table S4. Information available on maternal gestational diabetes, and white European ethnicity in the study population. Table S5. Results from sensitivity analyses of differentially methylated regions adding birthweight and delivery mode, removing cell types from confounders, excluding mothers with gestational diabetes and non-white European children. Results are shown only if the region has FDR- and Siddak adjusted p-value < 0.01 in DMRcate and ENmix-comb-p, respectively, in sensitivity analyses. Table S6. Count of missing CpGs for calculation of gestational age clocks and Spearman’s correlation coefficients between DNA methylation and chronological gestational age. Table S7. Results from meta-analyses of total, natural direct and indirect effect of prenatal exposures on rapid weight growth via gestational age acceleration. Table S8. Results from overrepresentation analyses (ORA) of transcripts associated at PSuggestive < 1e-05 with the 44 CpGs related to RWG and the 96 CpGs belonging to the 16 DMRs related to RWG. Table S9. List of CpGs belonging to each of the 16 DMRs associated with RWG with FDR- and Siddak adjusted p-value <0.01 in DMRcate and ENmix-comb-p, respectively. Table S10. Study population characteristics of the four studies (ALSPAC, ENVIRONAGE, EXPOsOMICS and GXXI) and total population included in the meta-analysis of EWAS of childhood overweight. Table S11. Look-up analysis of childhood overweight for the 44 CpGs assoc [file 12916_2022_2685_MOESM2_ESM.docx]

Additional File 2: Tables S1-S12 of

**Cord blood epigenome-wide meta-analysis in six European-based child cohorts identifies signatures linked to rapid weight growth**

Alfano R et al

**Table of Contents**

[Table S1. Comparison of characteristics of the ALSPAC, ENVIR*ON*AGE, EXPOsOMICS and GXXI cohorts by rapid weight growth status. 2](#_Toc120522215)

[Table S2. 49 CpGs associated with rapid weight growth in the meta-analysis of EWASs with P_Suggestive_ <1e-05. 4](#_Toc120522216)

[Table S3. Results from sensitivity analyses adding birthweight and delivery mode, removing cell types from confounders, excluding mothers with gestational diabetes and non-white European children, for the CpGs with P_Suggestive_<1e-05 in the meta-analysis of EWASs of rapid weight growth. 6](#_Toc120522217)

[Table S4. Information available on maternal gestational diabetes, and white European ethnicity in the study population. 12](#_Toc120522218)

[Table S5. Results from sensitivity analyses of differentially methylated regions adding birthweight and delivery mode, removing cell types from confounders, excluding mothers with gestational diabetes and non-white European children. Results are shown only if the region has FDR- and Siddak adjusted p-value < 0.01 in DMRcate and ENmix-comb-p, respectively, in sensitivity analyses. 13](#_Toc120522219)

[Table S6. Count of missing CpGs for calculation of gestational age clocks and Spearman’s correlation coefficients between DNA methylation and chronological gestational age. 18](#_Toc120522220)

[Table S7. Results from meta-analyses of total, natural direct and indirect effect of prenatal exposures on rapid weight growth via gestational age acceleration. 19](#_Toc120522221)

[Table S8. Results from overrepresentation analyses (ORA) of transcripts associated at P_Suggestive_ < 1e-05 with the 44 CpGs related to RWG and the 96 CpGs belonging to the 16 DMRs related to RWG. 20](#_Toc120522222)

[Table S9. List of CpGs belonging to each of the 16 DMRs associated with RWG with FDR- and Siddak adjusted p-value <0.01 in DMRcate and ENmix-comb-p, respectively. 22](#_Toc120522223)

[Table S10. Study population characteristics of the four studies (ALSPAC, ENVIRONAGE, EXPOsOMICS and GXXI) and total population included in the meta-analysis of EWAS of childhood overweight. 23](#_Toc120522224)

[Table S11. Look-up analysis of childhood overweight for the 44 CpGs associated at P_Suggestive_ <1e-05 in the meta-analysis of EWASs of rapid weight growth. 24](#_Toc120522225)

[Table S12. DMR that is associated with rapid growth and which is also significantly associated with childhood overweight. 25](#_Toc120522226)

# Table S1. Comparison of characteristics of the ALSPAC, ENVIR*ON*AGE, EXPOsOMICS and GXXI cohorts by rapid weight growth status.

|  | **ALSPAC**  729 | | | **ENVIR*ON*AGE**  247 | | | **EXPOsOMICS**  362 | | | **GXXI**  665 | | |
| --- | --- | --- | --- | --- | --- | --- | --- | --- | --- | --- | --- | --- |
|  | Non-RWG | RWG | p-value | Non-RWG | RWG | p-value | Non-RWG | RWG | p-value | Non-RWG | RWG | p-value |
| *N* | 502 (68.86) | 227 (31.14) |  | 178 (72.06) | 69 (26.94) |  | 260 (71. 82) | 102 (28.18) |  | 386 (58.05) | 279 (41.95) |  |
| *Sex, boys* | 237 (47.21) | 118 (51.98) | 0.27 | 97 (51.05) | 34 (45.33) | 0.48 | 142 (54.62) | 44 (43.14) | 0.06 | 218 (56.48) | 138 (49.46) | 0.09 |
| *Gestational age, weeks* | 39.86 (1.24) | 38.86 (1.82) | **<0.01** | 39.45 (1.17) | 38.65 (1.75) | **<0.01** | 39.54 (1.36) | 38.34 (1.75) | **<0.01** | 39.25 (1.10) | 38.34 (1.56) | **<0.01** |
| *Birthweight, grams* | 3683.84 (373.11) | 3049.63 (394.09) | **<0.01** | 3572.10 (382.83) | 3122.86 (389.28) | **<0.01** | 3406.56 (421.19) | 3014.80 (397.36) | **<0.01** | 3403.96 (385.95) | 2991.04 (416.53) | **<0.01** |
| *Parity, nulliparous* | 202 (40.24) | 142 (62.56) | **<0.01** | 85 (47.75) | 49 (71.01) | **0.01** | 115 (44.23) | 56 (54.90) | 0.09 | 200 (51.81) | 175 (62.72) | **0.01** |
| *Maternal age, years* | 29.84 (4.38) | 29.43 (4.47) | 0.24 | 30.44 (4.08) | 30.28 (4.25) | 0.77 | 31.25 (4.51) | 30.18 (4.61) | **0.04** | 29.53 (5.21) | 29.65 (5.24) | 0.77 |
| *Maternal education,* |  |  | 0.08 |  |  | 0.20 |  |  | **0.04** |  |  | 0.55 |
| *Low* | 240 (47.81) | 118 (51.98) |  | 9 (5.06) | 5 (7.25) |  | 27 (10.38) | 13 (12.75) |  | 185 (47.93) | 141 (50.54) |  |
| *Medium* | 146 (29.08) | 73 (32.16) |  | 56 (31.46) | 14 (20.29) |  | 101 (38.85) | 52 (50.98) |  | 94 (24.35) | 58 (20.79) |  |
| *High* | 116 (23.11) | 36 (15.86) |  | 113 (63.48) | 50 (72.46) |  | 132 (50.77) | 37 (36.27) |  | 107 (27.72) | 80 (28.67) |  |
| *Maternal smoking, yes* | 189 (37.65) | 92 (40.53) | 0.51 | 16 (8.99) | 10 (14.49) | 0.30 | 39 (15.00) | 25 (24.51) | **0.05** | 67 (17.36) | 75 (26.88) | **0.01** |
| *Maternal BMI, kg/m^2^* | 22.85 (3.60) | 22.79 (3.96) | 0.84 | 23.78 (4.20) | 24.20 (4.50) | 0.48 | 23.71 (4.50) | 24.58 (4.92) | 0.11 | 24.36 (4.53) | 23.68 (4.22) | **0.05** |
| *Maternal gestational diabetes, yes* | 5 (1.00) | 2 (0.88) | 1 | 11 (6.18) | 3 (4.35) | 0.80 | 17 (6.59) | 6 (5.94) | 1 | 10 (2.70) | 18 (6.87) | **0.02** |
| *Mode of delivery, caesarean section* | 38 (7.65) | 33 (14.54) | **<0.01** | 13 (7.30) | 0 (0.00) | **0.05** | 64 (24.71) | 33 (32.35) | 0.18 | 123 (31.87) | 77 (27.60) | 0.27 |
| *Ethnicity, non-white European* | - | - | - | 1 (0.57) | 0 (0.00) | 1 | - | - | - | 20 (5.19) | 19 (6.83) | 0.47 |

RWG= rapid weight growth

Counts (percentages) and means ± standard deviations are reported for categorical and continuous variables, respectively.

P-values from analysis of variance between RWG and non-RWG in each cohort was performed using chi-square or t-test for categorical and continuous variables, respectively.

# Table S2. 49 CpGs associated with rapid weight growth in the meta-analysis of EWASs with P_Suggestive_ <1e-05.

| **CpG** | **Gene name** | **Gene Region** | **coefficient** | **se** | **p-value** | **I^2^** | **I^2^ p-value** |
| --- | --- | --- | --- | --- | --- | --- | --- |
| cg25953130 | *ARID5B* | Body | 4.03 | 0.74 | 4.71E-08 | 0.00 | 0.68 |
| cg14459032 | *-* | - | 6.61 | 1.22 | 6.14E-08 | 43.42 | 0.15 |
| cg00049440 | *KLF9* | Body | 5.56 | 1.05 | 1.10E-07 | 14.15 | 0.32 |
| cg00442282 | *RARA* | 5'UTR | 11.41 | 2.16 | 1.27E-07 | 0.00 | 0.72 |
| cg02863179 | *ARID5B* | Body | 4.14 | 0.79 | 1.41E-07 | 41.54 | 0.16 |
| cg15203632 | *KCNK1* | Body | 10.20 | 2.03 | 4.87E-07 | 0.00 | 0.77 |
| cg13710814 | *ARNTL2* | Body | 13.84 | 2.75 | 5.03E-07 | 45.62 | 0.14 |
| cg05455036 | *-* | - | 7.36 | 1.47 | 5.29E-07 | 0.00 | 0.60 |
| cg11061434 | *ODZ2* | Body | 8.86 | 1.78 | 6.33E-07 | 40.31 | 0.17 |
| cg09294084 | *MCF2L* | Body | -4.80 | 0.98 | 9.24E-07 | 0.00 | 0.43 |
| cg20038219 | *-* | - | 4.51 | 0.92 | 9.77E-07 | 0.00 | 0.70 |
| cg04677123 | *-* | - | 4.18 | 0.86 | 1.05E-06 | 72.62 | 0.01 |
| cg20810396 | *-* | - | 12.57 | 2.60 | 1.31E-06 | 0.00 | 0.77 |
| cg02573176 | *SLC10A5* | TSS1500 | 5.07 | 1.05 | 1.35E-06 | 0.00 | 0.57 |
| cg00494183 | *-* | - | 7.80 | 1.61 | 1.37E-06 | 0.00 | 0.84 |
| cg00531137 | *-* | - | 4.38 | 0.91 | 1.53E-06 | 0.00 | 0.69 |
| cg22341513 | *-* | - | 6.04 | 1.27 | 1.91E-06 | 52.16 | 0.10 |
| cg24455365 | *PINK1* | Body | -11.75 | 2.47 | 1.95E-06 | 34.19 | 0.21 |
| cg06176471 | *ATP1B1* | Body | 11.30 | 2.40 | 2.51E-06 | 0.00 | 0.45 |
| cg04031093 | *-* | - | 4.16 | 0.89 | 2.77E-06 | 0.00 | 0.90 |
| cg26306530 | *-* | - | 10.29 | 2.20 | 2.91E-06 | 0.00 | 0.47 |
| cg05439724 | *-* | - | 15.70 | 3.38 | 3.32E-06 | 0.00 | 0.42 |
| cg00747477 | *ACCN1* | 3'UTR | 9.53 | 2.06 | 3.81E-06 | 0.00 | 0.53 |
| cg07810039 | *TGFB2* | Body | 4.32 | 0.94 | 3.84E-06 | 0.00 | 0.70 |
| cg20782117 | *SLC25A13* | Body | 5.82 | 1.27 | 4.17E-06 | 0.00 | 0.99 |
| cg19311055 | *RNF5P1* | TSS1500 | 10.05 | 2.18 | 4.18E-06 | 0.00 | 0.85 |
| cg15061231 | *-* | - | 11.76 | 2.56 | 4.26E-06 | 0.00 | 0.96 |
| cg26682904 | *ANGPTL5* | TSS200 | 5.61 | 1.22 | 4.41E-06 | 34.17 | 0.21 |
| cg20938359 | *SLC6A12* | 5'UTR | 5.90 | 1.29 | 4.83E-06 | 2.60 | 0.38 |
| cg22807187 | *SNORD115-15* | Body | 12.50 | 2.73 | 4.83E-06 | 54.42 | 0.09 |
| cg24335751 | *PRDM16* | Body | 11.66 | 2.56 | 5.39E-06 | 75.00 | 0.01 |
| cg00813135 | *ITPR2* | Body | 13.26 | 2.91 | 5.41E-06 | 19.80 | 0.29 |
| cg20068209 | *TMEM30A* | Body | 4.12 | 0.91 | 6.03E-06 | 0.00 | 0.73 |
| cg23404711 | *FOS* | Body | -5.21 | 1.15 | 6.12E-06 | 15.73 | 0.31 |
| cg21844291 | *-* | - | 9.90 | 2.19 | 6.39E-06 | 0.00 | 0.39 |
| cg08228249 | *YPEL1* | Body | 9.68 | 2.15 | 6.65E-06 | 16.40 | 0.31 |
| cg07807757 | *-* | - | 6.97 | 1.55 | 6.74E-06 | 0.00 | 0.55 |
| cg00355235 | *-* | - | 11.24 | 2.50 | 7.12E-06 | 0.00 | 0.67 |
| cg07353306 | *TAP2* | Body | 12.70 | 2.84 | 7.74E-06 | 38.84 | 0.18 |
| cg24718197 | *-* | - | -5.35 | 1.20 | 7.76E-06 | 0.00 | 0.74 |
| cg20076442 | *-* | - | 4.37 | 0.98 | 8.09E-06 | 0.00 | 0.80 |
| cg07780199 | *CRTC1* | Body | 8.16 | 1.83 | 8.24E-06 | 55.14 | 0.08 |
| cg16072126 | *-* | - | 11.98 | 2.69 | 8.29E-06 | 0.00 | 0.41 |
| cg03747456 | *KRT80* | TSS1500 | 5.76 | 1.29 | 8.30E-06 | 0.00 | 0.60 |
| cg12614113 | *KCNMA1* | Body | 9.44 | 2.12 | 8.59E-06 | 0.00 | 0.40 |
| cg04246167 | *C3orf67* | 5'UTR | 5.45 | 1.23 | 9.29E-06 | 0.00 | 0.75 |
| cg26433582 | *TPCN2* | Body | 4.53 | 1.02 | 9.48E-06 | 0.00 | 0.46 |
| cg06846833 | *LYPD6B* | TSS1500 | 12.80 | 2.89 | 9.51E-06 | 0.00 | 0.48 |
| cg21731239 | *MAFF* | 5'UTR | 10.19 | 2.30 | 9.70E-06 | 0.00 | 0.85 |

P values are bolded if P_Bonferroni_ <1.25e-07 and italics if P_Suggestive_ <1e-05 p-values. Result are coloured in red have high heterogeneity with I^2^ >50%. I^2^= heterogeneity; TSS=transcriptional start site; UTR=untranslated region

# Table S3. Results from sensitivity analyses adding birthweight and delivery mode, removing cell types from confounders, excluding mothers with gestational diabetes and non-white European children, for the CpGs with P_Suggestive_<1e-05 in the meta-analysis of EWASs of rapid weight growth.

|  |  |  | **Meta-analysis additionally adjusted for birthweight** | | | | | **Meta-analysis adjusted for delivery mode** | | | | |
| --- | --- | --- | --- | --- | --- | --- | --- | --- | --- | --- | --- | --- |
| **CpG** | **Gene name** | **Gene Region** | **coefficient** | **se** | **p-value** | **I^2^** | **I^2^ p-value** | **coefficient** | **se** | **p-value** | **I^2^** | **I^2^ p-value** |
| cg25953130 | *ARID5B* | Body | 0.17 | 0.09 | 0.05 | 30.26 | 0.23 | 0.40 | 0.07 | **7.07E-08** | 0.00 | 0.66 |
| cg14459032 | *-* | - | 0.45 | 0.14 | 1.72E-03 | 5.89 | 0.36 | 0.67 | 0.12 | **6.02E-08** | 53.75 | 0.09 |
| cg00049440 | *KLF9* | Body | 0.32 | 0.12 | 9.52E-03 | 0.00 | 0.43 | 0.57 | 0.11 | **6.39E-08** | 24.37 | 0.27 |
| cg00442282 | *RARA* | 5'UTR | 0.89 | 0.25 | 4.60E-04 | 0.00 | 0.80 | 1.13 | 0.22 | *1.97E-07* | 0.00 | 0.76 |
| cg02863179 | *ARID5B* | Body | 0.34 | 0.09 | 1.69E-04 | 42.71 | 0.16 | 0.42 | 0.08 | *1.34E-07* | 47.53 | 0.13 |
| cg15203632 | *KCNK1* | Body | 1.07 | 0.24 | 1.09E-05 | 33.20 | 0.21 | 1.03 | 0.20 | *3.59E-07* | 0.00 | 0.79 |
| cg13710814 | *ARNTL2* | Body | 1.54 | 0.31 | **5.74E-07** | 30.65 | 0.23 | 1.40 | 0.28 | *4.53E-07* | 47.05 | 0.13 |
| cg05455036 | *-* | - | 0.80 | 0.17 | *2.63E-06* | 0.00 | 0.54 | 0.72 | 0.15 | *1.25E-06* | 0.00 | 0.62 |
| cg11061434 | *ODZ2* | Body | 0.81 | 0.20 | 6.42E-05 | 14.94 | 0.32 | 0.91 | 0.18 | *3.81E-07* | 33.76 | 0.21 |
| cg09294084 | *MCF2L* | Body | -0.30 | 0.11 | 7.02E-03 | 0.00 | 0.60 | -0.48 | 0.10 | *8.71E-07* | 0.00 | 0.41 |
| cg20038219 | *-* | - | 0.44 | 0.11 | 4.76E-05 | 0.00 | 0.82 | 0.46 | 0.09 | *6.93E-07* | 0.00 | 0.69 |
| cg20810396 | *-* | - | 1.25 | 0.29 | 1.80E-05 | 0.00 | 0.67 | 1.28 | 0.26 | *8.70E-07* | 0.00 | 0.81 |
| cg02573176 | *SLC10A5* | TSS1500 | 0.50 | 0.12 | 4.05E-05 | 0.00 | 0.58 | 0.52 | 0.11 | *1.02E-06* | 0.00 | 0.58 |
| cg00494183 | *-* | - | 0.76 | 0.19 | 3.85E-05 | 0.00 | 0.75 | 0.83 | 0.16 | *4.06E-07* | 0.00 | 0.84 |
| cg00531137 | *-* | - | 0.25 | 0.11 | 2.02E-02 | 0.00 | 0.62 | 0.44 | 0.09 | *1.29E-06* | 0.00 | 0.59 |
| cg24455365 | *PINK1* | Body | -1.10 | 0.27 | 3.15E-05 | 54.23 | 0.09 | -1.20 | 0.25 | *1.37E-06* | 34.82 | 0.20 |
| cg06176471 | *ATP1B1* | Body | 1.20 | 0.27 | 1.04E-05 | 29.12 | 0.24 | 1.15 | 0.24 | *2.10E-06* | 0.00 | 0.49 |
| cg04031093 | *-* | - | 0.24 | 0.10 | 0.02 | 0.00 | 0.96 | 0.41 | 0.09 | *3.27E-06* | 0.00 | 0.87 |
| cg26306530 | *-* | - | 1.01 | 0.25 | 5.56E-05 | 37.34 | 0.19 | 1.06 | 0.22 | *1.74E-06* | 0.00 | 0.45 |
| cg05439724 | *-* | - | 1.73 | 0.39 | 1.03E-05 | 0.00 | 0.42 | 1.62 | 0.34 | *1.79E-06* | 0.00 | 0.49 |
| cg00747477 | *ACCN1* | 3'UTR | 1.15 | 0.24 | *1.25E-06* | 0.00 | 0.87 | 0.95 | 0.21 | *3.88E-06* | 0.00 | 0.45 |
| cg07810039 | *TGFB2* | Body | 0.29 | 0.11 | 8.23E-03 | 0.00 | 0.68 | 0.43 | 0.09 | *4.18E-06* | 0.00 | 0.68 |
| cg20782117 | *SLC25A13* | Body | 0.28 | 0.14 | 4.79E-02 | 0.00 | 0.70 | 0.58 | 0.13 | *4.68E-06* | 0.00 | 0.99 |
| cg19311055 | *RNF5P1* | TSS1500 | 1.05 | 0.25 | 2.21E-05 | 0.00 | 0.90 | 1.02 | 0.22 | *3.55E-06* | 0.00 | 0.88 |
| cg15061231 | *-* | - | 1.23 | 0.29 | 2.12E-05 | 0.00 | 0.63 | 1.20 | 0.26 | *3.25E-06* | 0.00 | 0.96 |
| cg26682904 | *ANGPTL5* | TSS200 | 0.68 | 0.14 | **9.62E-07** | 0.00 | 0.95 | 0.57 | 0.12 | *3.78E-06* | 39.65 | 0.17 |
| cg20938359 | *SLC6A12* | 5'UTR | 0.47 | 0.15 | 1.89E-03 | 0.00 | 0.49 | 0.58 | 0.13 | *7.60E-06* | 11.32 | 0.34 |
| cg00813135 | *ITPR2* | Body | 1.26 | 0.33 | 1.61E-04 | 0.00 | 0.54 | 1.36 | 0.29 | *3.72E-06* | 22.49 | 0.28 |
| cg20068209 | *TMEM30A* | Body | 0.19 | 0.11 | 0.08 | 0.00 | 0.51 | 0.41 | 0.09 | *9.37E-06* | 0.00 | 0.66 |
| cg23404711 | *FOS* | Body | -0.63 | 0.14 | *6.48E-06* | 0.00 | 0.96 | -0.52 | 0.12 | *7.03E-06* | 17.04 | 0.31 |
| cg21844291 | *-* | - | 0.96 | 0.26 | 1.69E-04 | 40.58 | 0.17 | 1.00 | 0.22 | *5.47E-06* | 14.82 | 0.32 |
| cg08228249 | *YPEL1* | Body | 1.04 | 0.25 | 3.95E-05 | 0.00 | 0.49 | 0.98 | 0.22 | *5.85E-06* | 17.63 | 0.30 |
| cg07807757 | *-* | - | 0.53 | 0.18 | 3.62E-03 | 42.95 | 0.15 | 0.70 | 0.16 | *6.10E-06* | 0.00 | 0.57 |
| cg00355235 | *-* | - | 1.02 | 0.28 | 2.17E-04 | 23.39 | 0.27 | 1.14 | 0.25 | *5.44E-06* | 0.00 | 0.62 |
| cg07353306 | *TAP2* | Body | 1.31 | 0.32 | 4.19E-05 | 67.77 | 0.03 | 1.27 | 0.28 | *7.57E-06* | 35.51 | 0.20 |
| cg24718197 | *-* | - | -0.50 | 0.13 | 1.40E-04 | 0.00 | 0.70 | -0.54 | 0.12 | *7.31E-06* | 0.00 | 0.73 |
| cg20076442 | *-* | - | 0.21 | 0.11 | 0.07 | 2.26 | 0.38 | 0.44 | 0.10 | *8.23E-06* | 0.00 | 0.80 |
| cg16072126 | *-* | - | 1.26 | 0.31 | 3.63E-05 | 0.00 | 0.49 | 1.18 | 0.27 | 1.27E-05 | 8.28 | 0.35 |
| cg03747456 | *KRT80* | TSS1500 | 0.41 | 0.15 | 7.32E-03 | 0.00 | 0.39 | 0.58 | 0.13 | *7.62E-06* | 0.00 | 0.64 |
| cg12614113 | *KCNMA1* | Body | 0.90 | 0.24 | 2.06E-04 | 30.65 | 0.23 | 0.96 | 0.21 | *6.76E-06* | 0.00 | 0.39 |
| cg04246167 | *C3orf67* | 5'UTR | 0.45 | 0.15 | 1.87E-03 | 0.00 | 0.63 | 0.55 | 0.12 | *8.69E-06* | 0.00 | 0.73 |
| cg26433582 | *TPCN2* | Body | 0.41 | 0.12 | 8.07E-04 | 37.29 | 0.19 | 0.46 | 0.10 | *8.17E-06* | 0.00 | 0.43 |
| cg06846833 | *LYPD6B* | TSS1500 | 1.50 | 0.33 | *4.46E-06* | 0.00 | 0.62 | 1.33 | 0.29 | *5.11E-06* | 0.00 | 0.48 |
| cg21731239 | *MAFF* | 5'UTR | 1.03 | 0.27 | 1.29E-04 | 0.00 | 0.69 | 1.03 | 0.23 | *8.35E-06* | 0.00 | 0.87 |

P values are bolded if P_Bonferroni_ <1.25e-07 and italics if P_Significant_ <1e-05 p-values. Result are coloured in red have high heterogeneity with I^2^ >50%. I^2^= heterogeneity; TSS=transcriptional start site; UTR=untranslated region

|  |  |  | **Meta-analysis not adjusted for cell types** | | | | | **Meta-analysis excluding mothers with GD** | | | | |
| --- | --- | --- | --- | --- | --- | --- | --- | --- | --- | --- | --- | --- |
| **CpG** | **Gene name** | **Gene Region** | **coefficient** | **se** | **p-value** | **I^2^** | **I^2^ p-value** | **coefficient** | **se** | **p-value** | **I^2^** | **I^2^ p-value** |
| cg25953130 | *ARID5B* | Body | 0.43 | 0.07 | **2.36E-10** | 0.00 | 0.63 | 0.41 | 0.08 | **5.73E-08** | 0.00 | 0.64 |
| cg14459032 | *-* | - | 0.69 | 0.09 | **1.81E-15** | 0.00 | 0.63 | 0.67 | 0.13 | **8.42E-08** | 45.82 | 0.14 |
| cg00049440 | *KLF9* | Body | 0.40 | 0.09 | 1.14E-05 | 0.00 | 0.68 | 0.56 | 0.11 | *2.07E-07* | 0.00 | 0.59 |
| cg00442282 | *RARA* | 5'UTR | 1.23 | 0.16 | **1.48E-14** | 0.00 | 0.68 | 1.30 | 0.22 | **6.43E-09** | 0.00 | 0.96 |
| cg02863179 | *ARID5B* | Body | 0.49 | 0.07 | **1.36E-13** | 31.24 | 0.22 | 0.42 | 0.08 | *3.06E-07* | 30.74 | 0.23 |
| cg15203632 | *KCNK1* | Body | 0.76 | 0.18 | 1.80E-05 | 6.97 | 0.36 | 0.96 | 0.21 | *4.84E-06* | 0.00 | 0.77 |
| cg13710814 | *ARNTL2* | Body | 0.84 | 0.23 | 2.20E-04 | 68.23 | 0.02 | 1.36 | 0.29 | *2.14E-06* | 40.05 | 0.17 |
| cg05455036 | *-* | - | 0.71 | 0.09 | **2.52E-15** | 24.93 | 0.26 | 0.77 | 0.15 | *4.45E-07* | 0.00 | 0.80 |
| cg11061434 | *ODZ2* | Body | 0.86 | 0.16 | **3.02E-08** | 52.14 | 0.10 | 0.93 | 0.19 | 5.58E-07 | 69.01 | 0.02 |
| cg09294084 | *MCF2L* | Body | -0.26 | 0.09 | 2.98E-03 | 49.25 | 0.12 | -0.50 | 0.10 | *6.22E-07* | 0.00 | 0.59 |
| cg20038219 | *-* | - | 0.45 | 0.09 | **3.03E-07** | 0.00 | 0.42 | 0.46 | 0.10 | *1.20E-06* | 0.00 | 0.68 |
| cg20810396 | *-* | - | 1.04 | 0.24 | 1.27E-05 | 17.26 | 0.30 | 1.30 | 0.27 | *1.47E-06* | 0.00 | 0.73 |
| cg02573176 | *SLC10A5* | TSS1500 | 0.61 | 0.08 | **1.18E-14** | 0.00 | 0.61 | 0.51 | 0.11 | *2.47E-06* | 0.00 | 0.59 |
| cg00494183 | *-* | - | 0.88 | 0.15 | **2.40E-09** | 0.00 | 0.76 | 0.83 | 0.17 | *7.69E-07* | 0.00 | 0.88 |
| cg00531137 | *-* | - | 0.17 | 0.07 | 0.02 | 0.00 | 0.55 | 0.47 | 0.09 | *8.01E-07* | 0.00 | 0.74 |
| cg24455365 | *PINK1* | Body | -0.77 | 0.23 | 7.29E-04 | 0.00 | 0.77 | -1.07 | 0.26 | 2.73E-05 | 21.24 | 0.28 |
| cg06176471 | *ATP1B1* | Body | 0.74 | 0.20 | 1.95E-04 | 0.00 | 0.50 | 1.14 | 0.25 | *4.65E-06* | 0.00 | 0.79 |
| cg04031093 | *-* | - | 0.52 | 0.07 | **1.56E-13** | 0.00 | 0.83 | 0.43 | 0.09 | *2.03E-06* | 0.00 | 0.94 |
| cg26306530 | *-* | - | 0.79 | 0.19 | 4.38E-05 | 0.00 | 0.50 | 1.00 | 0.23 | 1.09E-05 | 0.00 | 0.86 |
| cg05439724 | *-* | - | 1.14 | 0.30 | 1.30E-04 | 44.55 | 0.14 | 1.58 | 0.35 | *5.81E-06* | 0.00 | 0.45 |
| cg00747477 | *ACCN1* | 3'UTR | 0.74 | 0.18 | 5.10E-05 | 47.45 | 0.13 | 0.93 | 0.21 | 1.40E-05 | 0.00 | 0.47 |
| cg07810039 | *TGFB2* | Body | 0.55 | 0.08 | **5.19E-11** | 0.00 | 0.91 | 0.43 | 0.10 | *6.03E-06* | 0.00 | 0.55 |
| cg20782117 | *SLC25A13* | Body | 0.72 | 0.11 | **9.56E-11** | 0.00 | 0.78 | 0.60 | 0.13 | *5.03E-06* | 0.00 | 0.99 |
| cg19311055 | *RNF5P1* | TSS1500 | 0.77 | 0.19 | 6.74E-05 | 28.34 | 0.24 | 0.99 | 0.23 | 1.47E-05 | 0.00 | 0.97 |
| cg15061231 | *-* | - | 0.85 | 0.22 | 1.21E-04 | 0.00 | 0.64 | 1.23 | 0.27 | *3.92E-06* | 0.00 | 0.98 |
| cg26682904 | *ANGPTL5* | TSS200 | 0.68 | 0.11 | **8.96E-11** | 0.00 | 0.56 | 0.58 | 0.13 | *5.69E-06* | 45.69 | 0.14 |
| cg20938359 | *SLC6A12* | 5'UTR | 0.80 | 0.11 | **2.64E-13** | 0.00 | 0.92 | 0.57 | 0.13 | 1.59E-05 | 0.00 | 0.46 |
| cg00813135 | *ITPR2* | Body | 0.86 | 0.25 | 6.43E-04 | 0.00 | 0.69 | 1.29 | 0.30 | 1.89E-05 | 1.69 | 0.38 |
| cg20068209 | *TMEM30A* | Body | 0.50 | 0.08 | **1.66E-10** | 0.00 | 0.44 | 0.45 | 0.09 | *1.37E-06* | 0.00 | 0.41 |
| cg23404711 | *FOS* | Body | -0.52 | 0.11 | *2.60E-06* | 0.00 | 0.48 | -0.51 | 0.12 | 1.88E-05 | 2.82 | 0.38 |
| cg21844291 | *-* | - | 0.78 | 0.19 | 5.36E-05 | 59.35 | 0.06 | 0.95 | 0.23 | 3.34E-05 | 0.00 | 0.42 |
| cg08228249 | *YPEL1* | Body | 0.49 | 0.17 | 4.19E-03 | 71.84 | 0.01 | 0.97 | 0.22 | 1.24E-05 | 19.91 | 0.29 |
| cg07807757 | *-* | - | 0.90 | 0.13 | **5.42E-12** | 0.00 | 0.44 | 0.70 | 0.16 | 1.32E-05 | 0.00 | 0.77 |
| cg00355235 | *-* | - | 0.75 | 0.21 | 3.68E-04 | 0.00 | 0.74 | 1.16 | 0.26 | 1.20E-05 | 0.00 | 0.68 |
| cg07353306 | *TAP2* | Body | 0.96 | 0.25 | 9.93E-05 | 32.57 | 0.22 | 1.20 | 0.29 | 4.49E-05 | 50.78 | 0.11 |
| cg24718197 | *-* | - | -0.59 | 0.11 | **2.95E-07** | 0.00 | 0.94 | -0.49 | 0.12 | 8.68E-05 | 0.00 | 0.69 |
| cg20076442 | *-* | - | 0.49 | 0.09 | **6.09E-09** | 0.00 | 0.96 | 0.47 | 0.10 | *2.68E-06* | 0.00 | 0.83 |
| cg16072126 | *-* | - | 0.75 | 0.22 | 8.71E-04 | 43.06 | 0.15 | 1.09 | 0.28 | 8.13E-05 | 0.00 | 0.69 |
| cg03747456 | *KRT80* | TSS1500 | 0.73 | 0.10 | **1.14E-12** | 0.00 | 0.70 | 0.62 | 0.13 | *3.76E-06* | 0.00 | 0.62 |
| cg12614113 | *KCNMA1* | Body | 0.74 | 0.19 | 9.12E-05 | 45.77 | 0.14 | 0.94 | 0.22 | 2.34E-05 | 0.00 | 0.54 |
| cg04246167 | *C3orf67* | 5'UTR | 0.53 | 0.10 | **1.51E-07** | 0.00 | 0.97 | 0.56 | 0.13 | 9.33E-06 | 0.00 | 0.79 |
| cg26433582 | *TPCN2* | Body | 0.21 | 0.09 | 0.02 | 0.00 | 0.67 | 0.49 | 0.11 | *3.83E-06* | 17.68 | 0.30 |
| cg06846833 | *LYPD6B* | TSS1500 | 0.88 | 0.24 | 2.91E-04 | 0.00 | 0.57 | 1.33 | 0.30 | 1.04E-05 | 0.00 | 0.60 |
| cg21731239 | *MAFF* | 5'UTR | 0.61 | 0.19 | 1.40E-03 | 0.27 | 0.39 | 1.06 | 0.24 | *7.98E-06* | 0.00 | 0.94 |

P values are bolded if P_Bonferroni_ <1.25e-07 and italics if P_Significant_ <1e-05 p-values. Result are coloured in red have high heterogeneity with I^2^ >50%. GD= gestational diabetes; I^2^= heterogeneity; TSS=transcriptional start site; UTR=untranslated region

|  |  |  | **Meta-analysis excluding non-white European children** | | | | |
| --- | --- | --- | --- | --- | --- | --- | --- |
| **CpG** | **Gene name** | **Gene Region** | **coefficient** | **se** | **p-value** | **I^2^** | **I^2^ p-value** |
| cg25953130 | *ARID5B* | Body | 0.42 | 0.08 | **1.54E-08** | 0.00 | 0.64 |
| cg14459032 | *-* | - | 0.65 | 0.12 | *1.67E-07* | 41.58 | 0.16 |
| cg00049440 | *KLF9* | Body | 0.57 | 0.11 | **1.07E-07** | 2.21 | 0.38 |
| cg00442282 | *RARA* | 5'UTR | 1.21 | 0.22 | **3.73E-08** | 0.00 | 0.77 |
| cg02863179 | *ARID5B* | Body | 0.40 | 0.08 | *4.00E-07* | 38.42 | 0.18 |
| cg15203632 | *KCNK1* | Body | 1.06 | 0.21 | *2.33E-07* | 0.00 | 0.88 |
| cg13710814 | *ARNTL2* | Body | 1.36 | 0.28 | *1.25E-06* | 47.30 | 0.13 |
| cg05455036 | *-* | - | 0.77 | 0.15 | *2.57E-07* | 0.00 | 0.65 |
| cg11061434 | *ODZ2* | Body | 0.88 | 0.18 | *1.07E-06* | 40.12 | 0.17 |
| cg09294084 | *MCF2L* | Body | -0.50 | 0.10 | *5.22E-07* | 0.00 | 0.51 |
| cg20038219 | *-* | - | 0.45 | 0.09 | *1.18E-06* | 0.00 | 0.69 |
| cg20810396 | *-* | - | 1.27 | 0.26 | *1.41E-06* | 0.00 | 0.78 |
| cg02573176 | *SLC10A5* | TSS1500 | 0.53 | 0.11 | *6.63E-07* | 0.00 | 0.64 |
| cg00494183 | *-* | - | 0.81 | 0.16 | *8.38E-07* | 0.00 | 0.81 |
| cg00531137 | *-* | - | 0.45 | 0.09 | *8.90E-07* | 0.00 | 0.51 |
| cg24455365 | *PINK1* | Body | -1.30 | 0.26 | *3.71E-07* | 29.95 | 0.23 |
| cg06176471 | *ATP1B1* | Body | 1.13 | 0.24 | *3.39E-06* | 4.70 | 0.37 |
| cg04031093 | *-* | - | 0.46 | 0.09 | *4.94E-07* | 0.00 | 0.83 |
| cg26306530 | *-* | - | 1.06 | 0.22 | *2.17E-06* | 0.00 | 0.47 |
| cg05439724 | *-* | - | 1.55 | 0.34 | *5.92E-06* | 0.00 | 0.40 |
| cg00747477 | *ACCN1* | 3'UTR | 0.90 | 0.21 | 1.53E-05 | 0.00 | 0.60 |
| cg07810039 | *TGFB2* | Body | 0.45 | 0.09 | *1.77E-06* | 0.00 | 0.69 |
| cg20782117 | *SLC25A13* | Body | 0.66 | 0.13 | *4.40E-07* | 0.00 | 0.93 |
| cg19311055 | *RNF5P1* | TSS1500 | 1.00 | 0.22 | *6.58E-06* | 0.00 | 0.81 |
| cg15061231 | *-* | - | 1.16 | 0.26 | *8.81E-06* | 0.00 | 0.94 |
| cg26682904 | *ANGPTL5* | TSS200 | 0.53 | 0.12 | 1.75E-05 | 26.41 | 0.25 |
| cg20938359 | *SLC6A12* | 5'UTR | 0.57 | 0.13 | 1.22E-05 | 0.00 | 0.43 |
| cg00813135 | *ITPR2* | Body | 1.34 | 0.30 | *5.93E-06* | 19.99 | 0.29 |
| cg20068209 | *TMEM30A* | Body | 0.43 | 0.09 | *2.79E-06* | 0.00 | 0.52 |
| cg23404711 | *FOS* | Body | -0.53 | 0.12 | *5.83E-06* | 22.04 | 0.28 |
| cg21844291 | *-* | - | 0.98 | 0.22 | *9.01E-06* | 0.00 | 0.41 |
| cg08228249 | *YPEL1* | Body | 0.94 | 0.22 | 1.85E-05 | 18.65 | 0.30 |
| cg07807757 | *-* | - | 0.79 | 0.16 | *6.27E-07* | 0.00 | 0.45 |
| cg00355235 | *-* | - | 1.06 | 0.25 | 2.84E-05 | 0.00 | 0.67 |
| cg07353306 | *TAP2* | Body | 1.24 | 0.29 | 1.60E-05 | 37.31 | 0.19 |
| cg24718197 | *-* | - | -0.55 | 0.12 | *7.21E-06* | 0.00 | 0.78 |
| cg20076442 | *-* | - | 0.46 | 0.10 | *3.87E-06* | 0.00 | 0.77 |
| cg16072126 | *-* | - | 1.21 | 0.27 | *8.67E-06* | 8.00 | 0.35 |
| cg03747456 | *KRT80* | TSS1500 | 0.60 | 0.13 | *5.16E-06* | 0.00 | 0.52 |
| cg12614113 | *KCNMA1* | Body | 0.93 | 0.21 | 1.37E-05 | 1.38 | 0.39 |
| cg04246167 | *C3orf67* | 5'UTR | 0.54 | 0.12 | 1.41E-05 | 0.00 | 0.81 |
| cg26433582 | *TPCN2* | Body | 0.50 | 0.10 | *1.53E-06* | 31.92 | 0.22 |
| cg06846833 | *LYPD6B* | TSS1500 | 1.26 | 0.29 | 1.72E-05 | 0.00 | 0.47 |
| cg21731239 | *MAFF* | 5'UTR | 1.04 | 0.23 | *7.75E-06* | 0.00 | 0.85 |

P values are bolded if P_Bonferroni_ <1.25e-07 and italics if P_Significant_ <1e-05 p-values. Result are coloured in red have high heterogeneity with I^2^ >50%. I^2^= heterogeneity; TSS=transcriptional start site; UTR=untranslated region

# Table S4. Information available on maternal gestational diabetes, and white European ethnicity in the study population.

|  |  | **Maternal gestational diabetes** | | **White European ethnicity** | |
| --- | --- | --- | --- | --- | --- |
| **Study population** | **N** | **Yes** | **Missing** | **No** | **Missing** |
| **ALSPAC** | 729 | 7 (0.97) | 5 (0.69) | 1 (0.14) | 8 (1.10) |
| **ENVIR*ON*AGE** | 247 | 14 (5.67) | 0 (0.00) | 1 (0.41) | 2 (0.81) |
| **EXPOsOMICS** | 362 | 23 (6.41) | 3 (0.83) | - | - |
| **GXXI** | 665 | 28 (4.43) | 33 (4.96) | 39 (5.88) | 2 (0.30) |
| **Meta-analysis** | 2003 | 72 (3.67) | 41 (2.05) | 41 (2.05) | 12 (0.60) |

# Table S5. Results from sensitivity analyses of differentially methylated regions adding birthweight and delivery mode, removing cell types from confounders, excluding mothers with gestational diabetes and non-white European children. Results are shown only if the region has FDR- and Siddak adjusted p-value < 0.01 in DMRcate and ENmix-comb-p, respectively, in sensitivity analyses.

|  | **Sensitivity analysis additionally adjusted for birthweight** | | | | | | **Sensitivity analysis adjusted for delivery mode** | | | | | |
| --- | --- | --- | --- | --- | --- | --- | --- | --- | --- | --- | --- | --- |
|  | **Comb-p** | | | **DMRcate** | | | **Comb-p** | | | **DMRcate** | | |
| **Gene annotation** | **Genomic coordinates** | **N** | **Siddak p-value** | **Genomic coordinates** | **N** | **FDR p-value** | **Genomic coordinates** | **N** | **Siddak p-value** | **Genomic coordinates** | **N** | **FDR p-value** |
| *THEM5* | 1:151825973-151826205 | 4 | 2.64E-08 | chr1:151825974-151826533 | 5 | 2.01E-04 | 1:151825973-151826205 | 4 | 4.57E-08 | chr1:151825974-151826533 | 5 | 6.46E-06 |
| *CLDN4* | - | - | - | - | - | - | 7:73241728-73242028 | 4 | 2.75E-07 | chr7:73241729-73242028 | 4 | 4.83E-05 |
| *FETUB* | - | - | - | - | - | - | 3:186353574-186353721 | 2 | 2.03E-07 | chr3:186353350-186353721 | 3 | 1.51E-04 |
| *LOXL1-AS1* | - | - | - | - | - | - | 15:74218696-74218921 | 7 | 2.03E-07 | chr15:74218418-74219307 | 11 | 4.83E-06 |
| *PRDM16* | - | - | - | - | - | - | 1:3239991-3240227 | 3 | 2.33E-07 | chr1:3239712-3240227 | 4 | 1.17E-04 |
| *STK10* | - | - | - | - | - | - | 5:171616387-171616574 | 3 | 2.75E-07 | chr5:171616388-171616574 | 3 | 9.22E-05 |
| *C17orf64* | - | - | - | - | - | - | 17:58499678-58499911 | 7 | 3.46E-07 | chr17:58499300-58500186 | 9 | 4.83E-05 |
| *TGM3* | - | - | - | - | - | - | 20:2276434-2276663 | 5 | 7.56E-07 | chr20:2275700-2277040 | 8 | 1.30E-04 |
| *GPX6* | - | - | - | - | - | - | 6:29454671-29454954 | 5 | 1.13E-06 | chr6:29454557-29455256 | 8 | 3.24E-04 |
| *ACTG1* | - | - | - | - | - | - | 17:79485528-79485709 | 2 | 1.53E-06 | chr17:79485529-79485934 | 3 | 5.18E-04 |
| *PDE4D* | - | - | - | - | - | - | 5:58883391-58883430 | 2 | 1.69E-06 | chr5:58882939-58883430 | 3 | 4.71E-04 |
| *CDHR4* | - | - | - | - | - | - | 3:49837528-49837654 | 2 | 9.39E-05 | chr3:49837348-49837689 | 4 | 9.67E-04 |
| *SPATA33* | - | - | - | - | - | - | 16:89734985-89735184 | 2 | 1.98E-06 | chr16:89734986-89735184 | 2 | 1.95E-03 |
| *ALOX12-AS1* | - | - | - | - | - | - | 17:6899296-6899380 | 6 | 1.47E-05 | chr17:6898738-6899758 | 13 | 4.54E-04 |
| *AURKC* | 19:57742111-57742444 | 8 | 1.72E-11 | chr19:57741988-57743416 | 10 | 5.31E-09 | - | - | - | - | - | - |
| *GNMT* | - | - | - | - | - | - | 6:42927939-42927959 | 2 | 4.79E-03 | chr6:42927940-42928144 | 8 | 2.56E-03 |

N= number of CpGs in the region; FDR= false discovery rate

|  | **Sensitivity analysis removing cell types from confounders** | | | | | | **Sensitivity analysis excluding mothers with GD** | | | | | |
| --- | --- | --- | --- | --- | --- | --- | --- | --- | --- | --- | --- | --- |
|  | **Comb-p** | | | **DMRcate** | | | **Comb-p** | | | **DMRcate** | | |
| **Gene annotation** | **Genomic coordinates** | **N** | **Siddak p-value** | **Genomic coordinates** | **N** | **FDR p-value** | **Genomic coordinates** | **N** | **Siddak p-value** | **Genomic coordinates** | **N** | **FDR p-value** |
| *THEM5* | 1:151825973-151826205 | 4 | 2.67E-05 | chr1:151825974-151826533 | 5 | 2.35E-05 | 1:151825973-151826205 | 4 | 7.97E-08 | chr1:151825974-151826533 | 5 | 1.36E-04 |
| *CLDN4* | 7:73241728-73242028 | 4 | 5.27E-09 | chr7:73241729-73242028 | 4 | 1.25E-11 | 7:73241728-73242028 | 4 | 3.79E-07 | chr7:73241729-73242028 | 4 | 3.05E-04 |
| *FETUB* | - | - | - | - | - | - | 3:186353574-186353721 | 2 | 3.79E-07 | chr3:186353350-186353721 | 3 | 3.93E-04 |
| *LOXL1-AS1* | - | - | - | - | - | - | - | - | - | - | - | - |
| *PRDM16* | - | - | - | - | - | - | 1:3239991-3240227 | 3 | 3.79E-07 | chr1:3239712-3240227 | 4 | 4.68E-04 |
| *STK10* | - | - | - | - | - | - | 5:171616387-171616574 | 3 | 4.13E-07 | chr5:171616388-171616574 | 3 | 4.01E-04 |
| *C17orf64* | - | - | - | - | - | - | 17:58499678-58499854 | 6 | 7.34E-07 | chr17:58499300-58500186 | 9 | 6.42E-04 |
| *TGM3* | 20:2276434-2276663 | 5 | 6.45E-05 | chr20:2275700-2277040 | 8 | 4.76E-06 | 20:2276434-2276663 | 5 | 5.08E-07 | chr20:2276032-2277040 | 7 | 3.51E-04 |
| *GPX6* | 6:29454556-29454954 | 7 | 3.33E-08 | chr6:29454557-29455532 | 14 | 9.69E-14 | 6:29454671-29454954 | 5 | 1.14E-06 | chr6:29454557-29454954 | 7 | 8.46E-04 |
| *ACTG1* | 17:79485528-79485709 | 2 | 6.28E-09 | chr17:79484960-79486170 | 6 | 1.57E-10 | - | - | - | - | - | - |
| *PDE4D* | 5:58883391-58883430 | 2 | 1.33E-07 | chr5:58882753-58883430 | 4 | 2.73E-09 | 5:58883391-58883430 | 2 | 1.26E-06 | chr5:58882939-58883430 | 3 | 7.86E-04 |
| *CDHR4* | 3:49837347-49837654 | 3 | 1.95E-06 | chr3:49837348-49837689 | 4 | 1.43E-07 | 3:49837528-49837654 | 2 | 0.000129 | chr3:49837348-49837689 | 4 | 2.31E-03 |
| *SPATA33* | 16:89734985-89735184 | 2 | 4.33E-08 | chr16:89734445-89735184 | 3 | 2.24E-08 | 16:89734985-89735184 | 2 | 4.13E-07 | chr16:89734986-89735184 | 2 | 5.41E-04 |
| *ALOX12-AS1* | - | - | - | - | - | - | - | - | - | - | - | - |
| *AURKC* | 19:57742111-57742444 | 8 | 3.18E-06 | chr19:57741988-57743416 | 10 | 5.73E-09 | 19:57742216-57742444 | 7 | 7.86E-07 | chr19:57741988-57742444 | 9 | 3.51E-04 |
| *GNMT* |  | - | - | - | - | - | 1:151825973-151826205 | 4 | 7.97E-08 | chr1:151825974-151826533 | 5 | 1.36E-04 |

GD= gestational diabetes; N= number of CpGs in the region; FDR= false discovery rate

|  | **Sensitivity analysis excluding non-white European children** | | | | | |
| --- | --- | --- | --- | --- | --- | --- |
|  | **Comb-p** | | | **DMRcate** | | |
| **Gene annotation** | **Genomic coordinates** | **N** | **Siddak p-value** | **Genomic coordinates** | **N** | **FDR p-value** |
| *THEM5* | 1:151825973-151826205 | 4 | 5.38E-08 | chr1:151825974-151826533 | 5 | 7.53E-06 |
| *CLDN4* | 7:73241728-73242028 | 4 | 5.13E-07 | chr7:73241729-73242028 | 4 | 7.97E-05 |
| *FETUB* | - | - | - | - | - | - |
| *LOXL1-AS1* | 15:74218696-74218921 | 7 | 1.81E-07 | chr15:74218418-74219307 | 11 | 7.42E-06 |
| *PRDM16* | 1:3239991-3240227 | 3 | 7.27E-07 | chr1:3239712-3240227 | 4 | 5.02E-04 |
| *STK10* | 5:171616387-171616574 | 3 | 1.81E-07 | chr5:171616388-171616574 | 3 | 3.44E-05 |
| *C17orf64* | 17:58499678-58499911 | 7 | 7.16E-07 | chr17:58499300-58500186 | 9 | 7.97E-05 |
| *TGM3* | 20:2276434-2276663 | 5 | 2.39E-06 | chr20:2276032-2276663 | 6 | 9.48E-04 |
| *GPX6* | 6:29454671-29454954 | 5 | 1.68E-06 | chr6:29454557-29454954 | 7 | 7.20E-04 |
| *ACTG1* | 17:79485528-79485709 | 2 | 1.68E-06 | chr17:79485529-79485934 | 3 | 6.57E-04 |
| *PDE4D* | 5:58883391-58883430 | 2 | 2.04E-06 | chr5:58882939-58883430 | 3 | 6.40E-04 |
| *CDHR4* | 3:49837347-49837654 | 3 | 2.26E-06 | chr3:49837348-49837689 | 4 | 7.20E-04 |
| *SPATA33* | 16:89734985-89735184 | 2 | 2.39E-06 | chr16:89734986-89735184 | 2 | 2.18E-03 |
| *ALOX12-AS1* | 17:6899084-6899522 | 10 | 1.68E-06 | chr17:6898738-6899865 | 14 | 3.17E-04 |
| *AURKC* | 19:57742216-57742444 | 7 | 2.87E-06 | chr19:57741988-57742444 | 9 | 7.20E-04 |
| *GNMT* | 6:42927939-42927959 | 2 | 6.50E-03 | chr6:42927940-42927986 | 4 | 7.94E-03 |

N= number of CpGs in the region; FDR= false discovery rate

# Table S6. Count of missing CpGs for calculation of gestational age clocks and Spearman’s correlation coefficients between DNA methylation and chronological gestational age.

|  |  | **Missing CpGs for clock calculation in each cohort (% over the total CpGs in the clock)** | | | | **Spearman’s correlation coefficients between DNA methylation gestational age and chronological gestational age per each cohort** | | | |
| --- | --- | --- | --- | --- | --- | --- | --- | --- | --- |
| **Clock type** | **Total CpGs in the clock** | **ALSPAC** | **ENVIR *ON*AGE** | **EXPOs OMICS** | **GXXI** | **ALSPAC** | **ENVIR *ON*AGE** | **EXPOs OMICS** | **GXXI** |
| Knight | 148 | 0 (0%) | 7 (5%) | 0 (0%) | 6 (4%) | 0.33 | 0.53 | 0.48 | 0.55 |
| Bohlin | 96 | 0 (0%) | 8 (8%) | 1 (0%) | 8 (8%) | 0.63 | 0.70 | 0.73 | 0.73 |

# Table S7. Results from meta-analyses of total, natural direct and indirect effect of prenatal exposures on rapid weight growth via gestational age acceleration.

| **Mediator (CpG, gene name or DMR genomic allocation)** | **Prenatal exposure** | **TE** | | | | **NDE** | | | | **NIE** | | | |
| --- | --- | --- | --- | --- | --- | --- | --- | --- | --- | --- | --- | --- | --- |
|  |  | **OR (95%CI)** | **p-value** | **I_2_** | **I_2_ p-value** | **OR (95%CI)** | **p-value** | **I_2_** | **I_2_ p-value** | **OR (95%CI)** | **p-value** | **I_2_** | **I_2_ p-value** |
| GAA | Maternal education level, *Low vs high* | 1.27 (0.97-1.65) | 0.08 | 15.42 | 0.31 | 1.25 (0.96-1.63) | 0.10 | 0.00 | 0.40 | 1.01 (0.98-1.03) | 0.73 | 0 | 0.51 |
| GAA | Pre-pregnancy maternal BMI, Kilograms/ meters^2^ | 1.00 (0.97-1.02) | 0.76 | 4.32 | 0.37 | 1.00 (0.98-1.03) | 0.95 | 2.25 | 0.38 | 1.00 (0.99-1.00) | 0.09 | 47.45 | 0.13 |
| GAA | Maternal pregnancy tobacco smoke, yes | 1.45 (1.15-1.83) | **1.95E-03** | 13.67 | 0.32 | 1.45 (1.15-1.84) | **1.68E-03** | 21.26 | 0.28 | 0.99 (0.66-1.02) | 0.45 | 0 | 0.75 |
| GAA | Maternal age, years | 1.03 (1.00-1.05) | **0.03** | 0.00 | 0.88 | 1.03 (1.01-1.06) | **0.02** | 0.00 | 0.91 | 1.00 (1.00-1.00) | 0.67 | 0 | 0.94 |
| GAA | Parity, primiparous | 2.31 (1.84-2.90) | **5.66e-13** | 5.80 | 0.36 | 2.36 (1.88-2.95) | **7.16e-14** | 4.63 | 0.37 | 0.99 (0.97-1.02) | 0.43 | 0.00 | 0.80 |

P-values<0.05 are bolded. GAA=gestational age acceleration; I_2_= heterogeneity; NDE= natural direct effect; NIE= natural indirect effect; OR= odds ratio; TE= total effect

# Table S8. Results from overrepresentation analyses (ORA) of transcripts associated at P_Suggestive_ < 1e-05 with the 44 CpGs related to RWG and the 96 CpGs belonging to the 16 DMRs related to RWG.

| **ORA for the gene expression signals associated at PSuggestive < 1e-05 with the 44 CpGs related to RWG** | | | |
| --- | --- | --- | --- |
| **Source database** | **Pathway** | **p-value** | **Gene expression input overlaps** |
| KEGG | Cell adhesion molecules - Homo sapiens (human) | 4.03E-03 | CD226; PVR; SDC4; ESAM |
| Reactome | MAPK family signaling cascades | 6.53E-03 | RASAL1; PEA15; JAK3; CDC14A; GFRA2 |
| Reactome | Cell junction organization | 6.99E-03 | FLNA; LAMA3; PVR |
| Reactome | Cell-Cell communication | 0.02 | FLNA; LAMA3; PVR |
| Reactome | RAF/MAP kinase cascade | 0.02 | RASAL1; PEA15; JAK3; GFRA2 |
| Reactome | MAPK1/MAPK3 signaling | 0.02 | RASAL1; PEA15; JAK3; GFRA2 |
| Reactome | SLC-mediated transmembrane transport | 0.02 | SLC27A1; SLC39A3; SLC25A26; SLC22A8 |
| **ORA for the gene expression signals associated at PSuggestive < 1e-05 with the 96 CpGs belonging to the 16 DMRs related to RWG** | | | |
| KEGG | Axon guidance - Homo sapiens (human) | 3.64E-04 | ROBO3; PIK3CD; MAPK3; SSH2; SEMA6B; PAK2 |
| KEGG | Type II diabetes mellitus - Homo sapiens (human) | 1.75E-03 | MAPK3; PIK3CD; SOCS2 |
| KEGG | Cholinergic synapse - Homo sapiens (human) | 2.84E-03 | KCNQ2; PIK3CD; CHRM1; MAPK3 |
| KEGG | Regulation of actin cytoskeleton - Homo sapiens (human) | 5.53E-03 | SSH2; PIK3CD; PAK2; CHRM1; MAPK3 |
| KEGG | Renal cell carcinoma - Homo sapiens (human) | 5.55E-03 | MAPK3; PIK3CD; PAK2 |
| KEGG | Insulin signaling pathway - Homo sapiens (human) | 5.64E-03 | MAPK3; PPP1R3D; PIK3CD; SOCS2 |
| KEGG | Prolactin signaling pathway - Homo sapiens (human) | 5.78E-03 | MAPK3; PIK3CD; SOCS2 |
| Reactome | Regulation of HSF1-mediated heat shock response | 8.65E-03 | COL4A6; BAG1; MAPK3 |
| KEGG | ErbB signaling pathway - Homo sapiens (human) | 9.87E-03 | MAPK3; PIK3CD; PAK2 |
| Reactome | Cellular response to heat stress | 0.01 | COL4A6; BAG1; MAPK3 |
| KEGG | AGE-RAGE signaling pathway in diabetic complications - Homo sapiens (human) | 0.02 | COL4A6; PIK3CD; MAPK3 |
| KEGG | T cell receptor signaling pathway - Homo sapiens (human) | 0.02 | MAPK3; PIK3CD; PAK2 |
| KEGG | TNF signaling pathway - Homo sapiens (human) | 0.02 | CX3CL1; MAPK3; PIK3CD |
| KEGG | Focal adhesion - Homo sapiens (human) | 0.02 | COL4A6; PIK3CD; PAK2; MAPK3 |
| KEGG | Sphingolipid signaling pathway - Homo sapiens (human) | 0.02 | MAPK3; ADORA3; PIK3CD |
| KEGG | Growth hormone synthesis, secretion and action - Homo sapiens (human) | 0.02 | MAPK3; PIK3CD; SOCS2 |
| KEGG | Human immunodeficiency virus 1 infection - Homo sapiens (human) | 0.02 | HLA-C; MAPK3; PIK3CD; PAK2 |
| Reactome | Immunoregulatory interactions between a Lymphoid and a non-Lymphoid cell | 0.03 | HLA-C; CD160; CD226; SIGLEC5 |
| KEGG | Human papillomavirus infection - Homo sapiens (human) | 0.03 | HLA-C; PIK3CD; MFNG; MAPK3; COL4A6 |
| KEGG | Relaxin signaling pathway - Homo sapiens (human) | 0.03 | COL4A6; PIK3CD; MAPK3 |
| KEGG | Human cytomegalovirus infection - Homo sapiens (human) | 0.03 | HLA-C; MAPK3; CX3CL1; PIK3CD |
| KEGG | Natural killer cell mediated cytotoxicity - Homo sapiens (human) | 0.03 | HLA-C; MAPK3; PIK3CD |
| KEGG | FoxO signaling pathway - Homo sapiens (human) | 0.03 | IL7R; MAPK3; PIK3CD |
| KEGG | Ras signaling pathway - Homo sapiens (human) | 0.03 | PIK3CD; PLA2G10; PAK2; MAPK3 |
| Reactome | Adaptive Immune System | 0.03 | LNX1; HLA-C; CD160; PIK3CD; CD226; SIGLEC5; SPSB1; PAK2 |
| Reactome | Cytokine Signaling in Immune system | 0.04 | IL7R; SOCS2; HLA-C; SDC1; PIK3CD; MAPK3 |
| Reactome | Signaling by Interleukins | 0.04 | IL7R; SDC1; PIK3CD; MAPK3 |
| KEGG | PI3K-Akt signaling pathway - Homo sapiens (human) | 0.04 | IL7R; COL4A6; PIK3CD; CHRM1; MAPK3 |
| KEGG | Cell adhesion molecules - Homo sapiens (human) | 0.04 | HLA-C; SDC1; CD226 |
| Reactome | Immune System | 0.05 | IL7R; LNX1; BPI; CD160; HLA-C; SOCS2; PIK3CD; MAPK3; SIGLEC5; CD226; SDC1; MME; ZBP1; SPSB1; PAK2 |
| Reactome | Nervous system development | 0.05 | MAPK3; PMP22; KCNQ2; ROBO3; PAK2 |
| KEGG | Cellular senescence - Homo sapiens (human) | 0.05 | HLA-C; MAPK3; PIK3CD |

DMRs= differentially methylated regions; ORA= overrepresentation analysis; RWG= rapid weight growth

# Table S9. List of CpGs belonging to each of the 16 DMRs associated with RWG with FDR- and Siddak adjusted p-value <0.01 in DMRcate and ENmix-comb-p, respectively.

|  | **Comb-p** | | **DMRcate** | |  |
| --- | --- | --- | --- | --- | --- |
| **DMR Gene annotation** | **Genomic coordinates** | **N** | **Genomic coordinates** | **N** | **CpGs in the DMRs** |
| *THEM5* | 1:151825973-151826205 | 4 | chr1:151825974-151826533 | 5 | cg01062247,cg11344950,cg18081104,cg26292918,cg07874520 |
| *CLDN4* | 7:73241728-73242028 | 4 | chr7:73241729-73242028 | 4 | cg04663842,cg12856114,cg20038219,cg22455527 |
| *FETUB* | 3:186353574-186353721 | 2 | chr3:186353350-186353721 | 3 | cg15061231,cg25735294,cg17813225 |
| *LOXL1-AS1* | 15:74218696-74218921 | 7 | chr15:74218418-74219307 | 11 | cg00028013,cg04436755,cg10372921,cg12594244,cg14435807,cg20652404,cg22590761,cg00071887,cg00527825,cg02812767,cg03682712 |
| *PRDM16* | 1:3239991-3240227 | 3 | chr1:3239712-3240227 | 4 | cg01418153,cg03254465,cg11138362,cg12753787 |
| *STK10* | 5:171616387-171616574 | 3 | chr5:171616388-171616574 | 3 | cg16675054,cg19873297,cg26006084 |
| *C17orf64* | 17:58499678-58499911 | 7 | chr17:58499300-58500186 | 9 | cg00443981,cg02172058,cg04413853,cg06752482,cg12131208,cg17628249,cg21122199,cg12788108,cg25910083 |
| *TGM3* | 20:2276434-2276663 | 5 | chr20:2275700-2277040 | 8 | cg09561663,cg11195933,cg12761282,cg24138234,cg24731241,cg13932768,cg21611708,cg25273860 |
| *GPX6* | 6:29454671-29454954 | 5 | chr6:29454557-29455256 | 8 | cg09083279,cg20103692,cg25730428,cg25798600,cg26371172,cg00237606,cg12423733,cg13305823 |
| *ACTG1* | 17:79485528-79485709 | 2 | chr17:79485529-79485934 | 3 | cg14316944,cg24718197,cg02010342 |
| *PDE4D* | 5:58883391-58883430 | 2 | chr5:58882939-58883430 | 3 | cg05593411,cg18804667,cg01174811 |
| *CDHR4* | 3:49837347-49837654 | 3 | chr3:49837348-49837689 | 4 | cg00514486,cg10677324,cg20026633,cg04455265 |
| *SPATA33* | 16:89734985-89735184 | 2 | chr16:89734986-89735184 | 2 | cg07835443,cg16725984 |
| *ALOX12-AS1* | 17:6899296-6899380 | 6 | chr17:6898738-6899758 | 13 | cg03404566,cg03407747,cg03760483,cg03762994,cg09737314,cg21237687,cg02493798,cg05215272,cg05215748,cg05657792,cg12262378,cg14375499,cg19536664 |
| *AURKC* | 19:57742344-57742421 | 3 | chr19:57741988-57742444 | 9 | cg06643849,cg19603903,cg23371413,cg15128510,cg19568003,cg22711741,cg25432232,cg25802888,cg26332114 |
| *GNMT* | 6:42927939-42927959 | 2 | chr6:42927940-42928079 | 7 | cg04706597,cg24815792,cg10862848,cg16682276,cg21280719,cg23093754,cg25671484 |

DMR= differentially methylated regions; N= number of CpGs in the region

# Table S10. Study population characteristics of the four studies (ALSPAC, ENVIRONAGE, EXPOsOMICS and GXXI) and total population included in the meta-analysis of EWAS of childhood overweight.

|  | **N** | **Girls** | **Rapid weight growth** | **Childhood overweight** | **Age at measurement of childhood BMI** | **Gestational age, weeks** | **Birthweight, grams** | **Nulliparity** | **Maternal age, years** | **Low maternal education** | **Maternal smoking** | **Maternal BMI, kg/m2** |
| --- | --- | --- | --- | --- | --- | --- | --- | --- | --- | --- | --- | --- |
| **ALSPAC** | 729 | 355 (48.70) | 227 (31.14) | 145 (20.11) | 7.14 (0.81) | 39.55 (1.52) | 3486.35 (479.98) | 344 (47.19) | 29.72 (4.41) | 358 (49.11) | 281 (38.55) | 22.83 (3.71) |
| **ENVIRONAGE** | 265 | 131 (49.43) | 75 (28.30) | 18 (6.87) | 4.58 (0.39) | 39.20 (1.44) | 3429.57 (434.19) | 146 (55.09) | 30.33 (4.09) | 15 (5.66) | 26 (9.81) | 23.81 (4.24) |
| **EXPOsOMICS** | 257 | 133 (51.75) | 71 (27.63) | 51 (21.89) | 5.36 (1.02) | 39.26 (1.55) | 3252.37 (429.76) | 108 (42.02) | 31.70 (4.68) | 31 (12.06) | 57 (22.18) | 23.88 (4.66) |
| **GXXI** | 665 | 356 (53.53) | 279 (41.95) | 221 (34.53) | 6.83 (0.57) | 38.87 (1.39) | 3230.72 (447.88) | 375 (56.39) | 29.58 (5.22) | 326 (49.02) | 142 (21.35) | 24.08 (4.41) |
| **Meta-analysis population** | 1916 | 975 (50.89) | 652 (34.03) | 435 (23.44) | 6.45 (1.18) | 39.22 (1.49) | 3358.39 (471.08) | 973 (50.78) | 30.02 (4.75) | 730 (38.10) | 506 (26.41) | 23.54 (4.21) |

N= number of observations

# Table S11. Look-up analysis of childhood overweight for the 44 CpGs associated at P_Suggestive_ <1e-05 in the meta-analysis of EWASs of rapid weight growth.

| **CpG** | **Gene name** | **Gene Region** | **coefficient** | **se** | **p-value** | **I^2^** | **I^2^ p-value** |
| --- | --- | --- | --- | --- | --- | --- | --- |
| cg25953130 | *ARID5B* | Body | 0.09 | 0.08 | 0.25 | 0.00 | 0.83 |
| cg14459032 | *-* | - | 0.19 | 0.13 | 0.14 | 0.00 | 0.52 |
| cg00049440 | *KLF9* | Body | 0.09 | 0.11 | 0.42 | 22.14 | 0.28 |
| cg00442282 | *RARA* | 5'UTR | 0.13 | 0.24 | 0.60 | 44.37 | 0.15 |
| cg02863179 | *ARID5B* | Body | 0.09 | 0.09 | 0.28 | 0.00 | 0.87 |
| cg15203632 | *KCNK1* | Body | 0.39 | 0.21 | 0.06 | 0.00 | 0.55 |
| cg13710814 | *ARNTL2* | Body | 0.21 | 0.28 | 0.45 | 0.00 | 0.41 |
| cg05455036 | *-* | - | 0.13 | 0.16 | 0.42 | 77.38 | 0.00 |
| cg11061434 | *ODZ2* | Body | -0.18 | 0.19 | 0.34 | 44.71 | 0.14 |
| cg09294084 | *MCF2L* | Body | -0.09 | 0.10 | 0.35 | 0.00 | 0.54 |
| cg20038219 | *-* | - | 0.19 | 0.10 | 0.06 | 4.72 | 0.37 |
| cg20810396 | *-* | - | 0.33 | 0.28 | 0.23 | 0.00 | 0.68 |
| cg02573176 | *SLC10A5* | TSS1500 | -0.13 | 0.11 | 0.25 | 67.49 | 0.03 |
| cg00494183 | *-* | - | 0.18 | 0.17 | 0.30 | 19.31 | 0.29 |
| cg00531137 | *-* | - | 0.19 | 0.10 | 0.06 | 0.00 | 0.52 |
| cg24455365 | *PINK1* | Body | -0.27 | 0.26 | 0.30 | 0.00 | 0.94 |
| cg06176471 | *ATP1B1* | Body | 0.46 | 0.25 | 0.07 | 0.00 | 0.39 |
| cg04031093 | *-* | - | 0.15 | 0.10 | 0.13 | 0.00 | 0.52 |
| cg26306530 | *-* | - | 0.11 | 0.23 | 0.63 | 0.00 | 0.40 |
| cg05439724 | *-* | - | 0.05 | 0.35 | 0.88 | 0.00 | 0.55 |
| cg00747477 | *ACCN1* | 3'UTR | 0.11 | 0.22 | 0.62 | 0.00 | 0.72 |
| cg07810039 | *TGFB2* | Body | 0.19 | 0.10 | 0.06 | 0.00 | 0.49 |
| cg20782117 | *SLC25A13* | Body | 0.01 | 0.14 | 0.95 | 31.64 | 0.22 |
| cg19311055 | *RNF5P1* | TSS1500 | 0.26 | 0.23 | 0.25 | 0.00 | 0.51 |
| cg15061231 | *-* | - | 0.20 | 0.27 | 0.45 | 0.00 | 0.92 |
| cg26682904 | *ANGPTL5* | TSS200 | 0.08 | 0.14 | 0.54 | 0.00 | 0.92 |
| cg20938359 | *SLC6A12* | 5'UTR | 0.18 | 0.14 | 0.20 | 64.89 | 0.04 |
| cg00813135 | *ITPR2* | Body | 0.11 | 0.30 | 0.72 | 0.00 | 0.58 |
| cg20068209 | *TMEM30A* | Body | -0.14 | 0.10 | 0.17 | 44.54 | 0.14 |
| cg23404711 | *FOS* | Body | -0.21 | 0.13 | 0.10 | 0.00 | 0.67 |
| cg21844291 | *-* | - | 0.44 | 0.23 | 0.05 | 45.66 | 0.14 |
| cg08228249 | *YPEL1* | Body | 0.12 | 0.23 | 0.61 | 0.00 | 0.80 |
| cg07807757 | *-* | - | 0.07 | 0.17 | 0.67 | 0.00 | 0.73 |
| cg00355235 | *-* | - | -0.16 | 0.25 | 0.53 | 0.00 | 0.93 |
| cg07353306 | *TAP2* | Body | 0.37 | 0.30 | 0.22 | 43.88 | 0.15 |
| cg24718197 | *-* | - | -0.12 | 0.14 | 0.37 | 0.00 | 0.96 |
| cg20076442 | *-* | - | -0.01 | 0.10 | 0.89 | 0.00 | 0.50 |
| cg16072126 | *-* | - | 0.33 | 0.28 | 0.24 | 0.00 | 0.66 |
| cg03747456 | *KRT80* | TSS1500 | 0.15 | 0.14 | 0.28 | 0.00 | 0.73 |
| cg12614113 | *KCNMA1* | Body | 0.32 | 0.22 | 0.15 | 0.00 | 0.51 |
| cg04246167 | *C3orf67* | 5'UTR | 0.02 | 0.13 | 0.88 | 24.81 | 0.26 |
| cg26433582 | *TPCN2* | Body | 0.19 | 0.11 | 0.09 | 57.07 | 0.07 |
| cg06846833 | *LYPD6B* | TSS1500 | 0.21 | 0.30 | 0.49 | 0.00 | 0.42 |
| cg21731239 | *MAFF* | 5'UTR | 0.15 | 0.23 | 0.52 | 47.05 | 0.13 |

Bolded p values are <0.05. Result are coloured in red have high heterogeneity with I^2^ >50%. I^2^= heterogeneity; TSS=transcriptional start site; UTR=untranslated region

# Table S12. DMR that is associated with rapid growth and which is also significantly associated with childhood overweight.

|  | **Comb-p** | | | **DMRcate** | | |
| --- | --- | --- | --- | --- | --- | --- |
| **Gene annotation** | **Genomic coordinates** | **N** | **Siddak p-value** | **Genomic coordinates** | **N** | **FDR p-value** |
| *AURKC* | 19:57741987-57742444 | 9 | 1.86E-15 | chr19:57741988-57743416 | 10 | 2.64E-13 |

N= number of CpGs
