# Supplementary material for: Cord blood epigenome-wide meta-analysis in six European-based child cohorts identifies signatures linked to rapid weight growth
Source: BMC Med. 2023 Jan 11;21:17. doi: 10.1186/s12916-022-02685-7 (PMC9831885; doi:10.1186/s12916-022-02685-7)
Supplement: Supplementary file 3 — Additional file 3: Figures S1-S12 [60]. Fig. S1. QQ-plot of I2p-values of the meta-analysis of EWASs of rapid weight growth. Fig. S2. Forrest plots and plots from leave out one cohort at time meta-analysis from EWASs of rapid weight growth for the CpGs with PSuggestive < 1e-05. Fig. S3. Quality control of cohort specific EWAS of rapid weight growth. Coefficients, standard errors and p-values distributions visualized via box plots and QQ-plot. Fig. S4. Cohort specific correlation plots between chronological and DNA methylation gestational age. Fig. S5. Forrest plot of the meta-analysis of gestational age acceleration and rapid weight growth in sensitivity analyses adding (A) delivery mode, (B) removing cell types from confounders, excluding mothers with (C) gestational diabetes and (D) non-white European children. Fig. S6. Calibration plots of Random Forest models of rapid weight growth including (A) conventional risk factors, (B) CpGs related to rapid weight growth and (C) both. Fig. S7. Calibration plots of Random Forest models of rapid weight growth including (A) conventional risk factors, (B) CpGs belonging to DMRs related to rapid weight growth and (C) both. Fig. S8. Heatmap shows Pearson’s correlation between methylation levels of the 44 CpGs associated with PSuggestive <1e-05 in the meta-analysis of EWAS of rapid weight growth. Red boxes indicate the three CpGs identified in mediation analyses. Fig. S9. Volcano plots of the association between the 44 CpGs associated with rapid weight growth at PSuggestive < 1e-05/the 96 CpGs belonging to the 16 DMRs associated with rapid weight growth at FDR-adjusted p-value in DMRcate and Siddak p-values in ENmix-comb-p <0.01 and the entire transcriptome (A/C) and restricted to cis transcripts (B/D). Red lines represent Bonferroni-significant threshold, and black lines suggestive threshold (p-value=10-e05). For analyses restricted to cis transcripts only Bonferroni-significant threshold is represented. Fig. S10. Volcano plots [file 12916_2022_2685_MOESM3_ESM.docx]

Additional File 3: Figures S1-S12 of

**Cord blood epigenome-wide meta-analysis in six European-based child cohorts identifies signatures linked to rapid weight growth**

Alfano R et al

**Figures S1-S12:**

[FigS1. QQ-plot of I^2^ p-values of the meta-analysis of EWASs of rapid weight growth. 3](#_Toc120522281)

[FigS2. Forrest plots and plots from leave out one cohort at time meta-analysis from EWASs of rapid weight growth for the CpGs with P_Suggestive_ < 1e-05. 4](#_Toc120522282)

[FigS3. Quality control of cohort specific EWAS of rapid weight growth. Coefficients, standard errors and p-values distributions visualized via box plots and QQ-plot. 13](#_Toc120522283)

[FigS4. Cohort specific correlation plots between chronological and DNA methylation gestational age. 14](#_Toc120522284)

[FigS5. Forrest plot of the meta-analysis of gestational age acceleration and rapid weight growth in sensitivity analyses adding (A) delivery mode, (B) removing cell types from confounders, excluding mothers with (C) gestational diabetes and (D) non-white European children. 15](#_Toc120522285)

[FigS6. Calibration plots of Random Forest models of rapid weight growth including (A) conventional risk factors, (B) CpGs related to rapid weight growth and (C) both. 16](#_Toc120522286)

[FigS7. Calibration plots of Random Forest models of rapid weight growth including (A) conventional risk factors, (B) CpGs belonging to DMRs related to rapid weight growth and (C) both. 17](#_Toc120522287)

[FigS8. Heatmap shows Pearson’s correlation between methylation levels of the 44 CpGs associated with P_Suggestive_ <1e-05 in the meta-analysis of EWAS of rapid weight growth. Red boxes indicate the three CpGs identified in mediation analyses. 18](#_Toc120522288)

[FigS9. Volcano plots of the association between the 44 CpGs associated with rapid weight growth at P_Suggestive_ < 1e-05/the 96 CpGs belonging to the 16 DMRs associated with rapid weight growth at FDR-adjusted p-value in DMRcate and Siddak p-values in ENmix-comb-p <0.01 and the entire transcriptome (A/C) and restricted to cis transcripts (B/D). Red lines represent Bonferroni-significant threshold, and black lines suggestive threshold (p-value=10-e05). For analyses restricted to cis transcripts only Bonferroni-significant threshold is represented. 19](#_Toc120522289)

[FigS10. Volcano plots of the association between the 44 CpGs associated with rapid weight growth at P_Suggestive_ < 1e-05/the 96 CpGs belonging to the 16 DMRs associated with rapid weight growth at FDR-adjusted p-value in DMRcate and Siddak p-values in ENmix-comb-p <0.01 and the entire metabolome (A/B). Red lines represent Bonferroni-significant threshold, and black lines suggestive threshold (p-value=10-e05). 20](#_Toc120522290)

[FigS11. Forrest plot of the meta-analysis of the analysis of gestational age acceleration and childhood overweight. 21](#_Toc120522291)

[FigS12. Volcano plot from the look-up in the study population of the CpG sites associated with child anthropometrics in a previous systematic review by Alfano et al. [60] 22](#_Toc120522292)

# FigS1. QQ-plot of I^2^ p-values of the meta-analysis of EWASs of rapid weight growth.

**
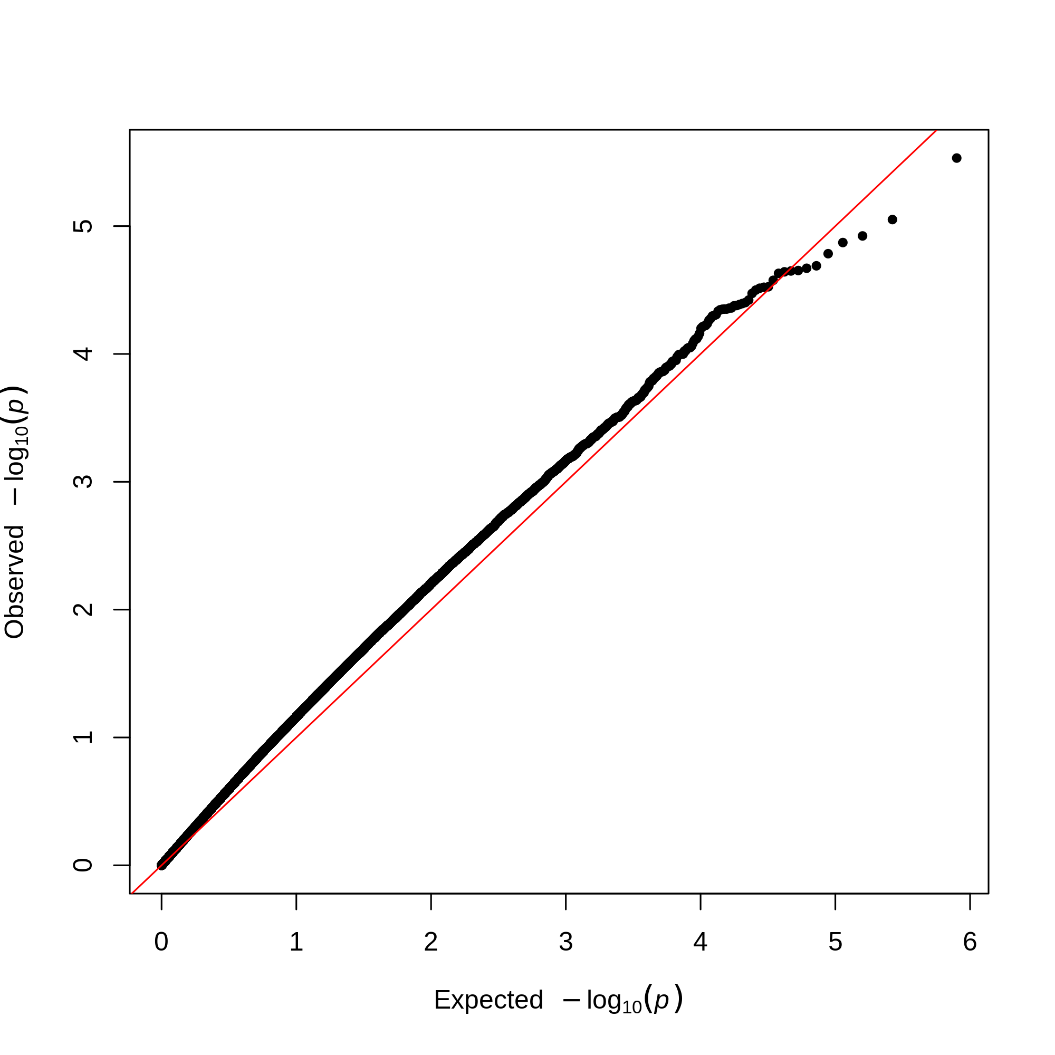
**

# FigS2. Forrest plots and plots from leave out one cohort at time meta-analysis from EWASs of rapid weight growth for the CpGs with P_Suggestive_ < 1e-05.


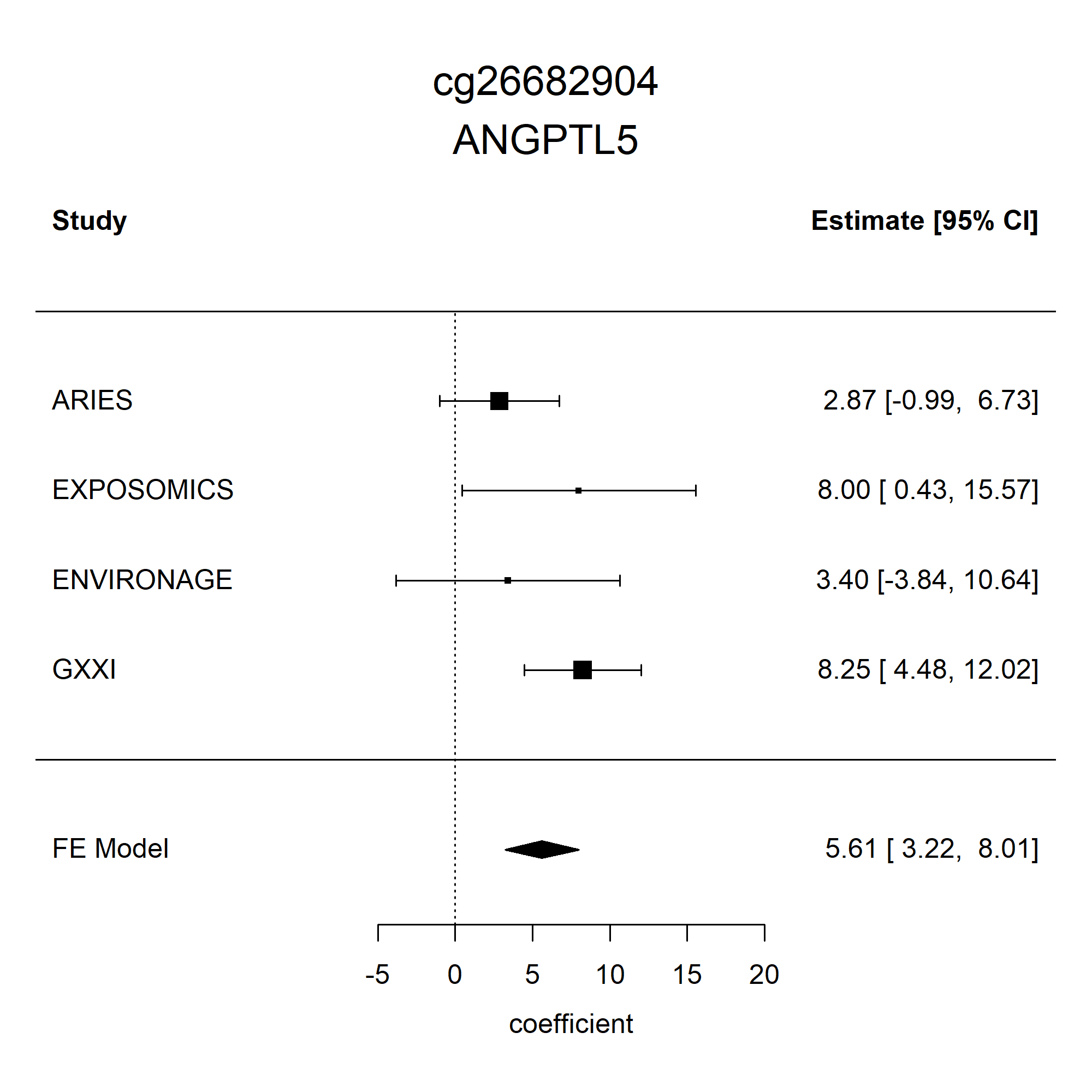

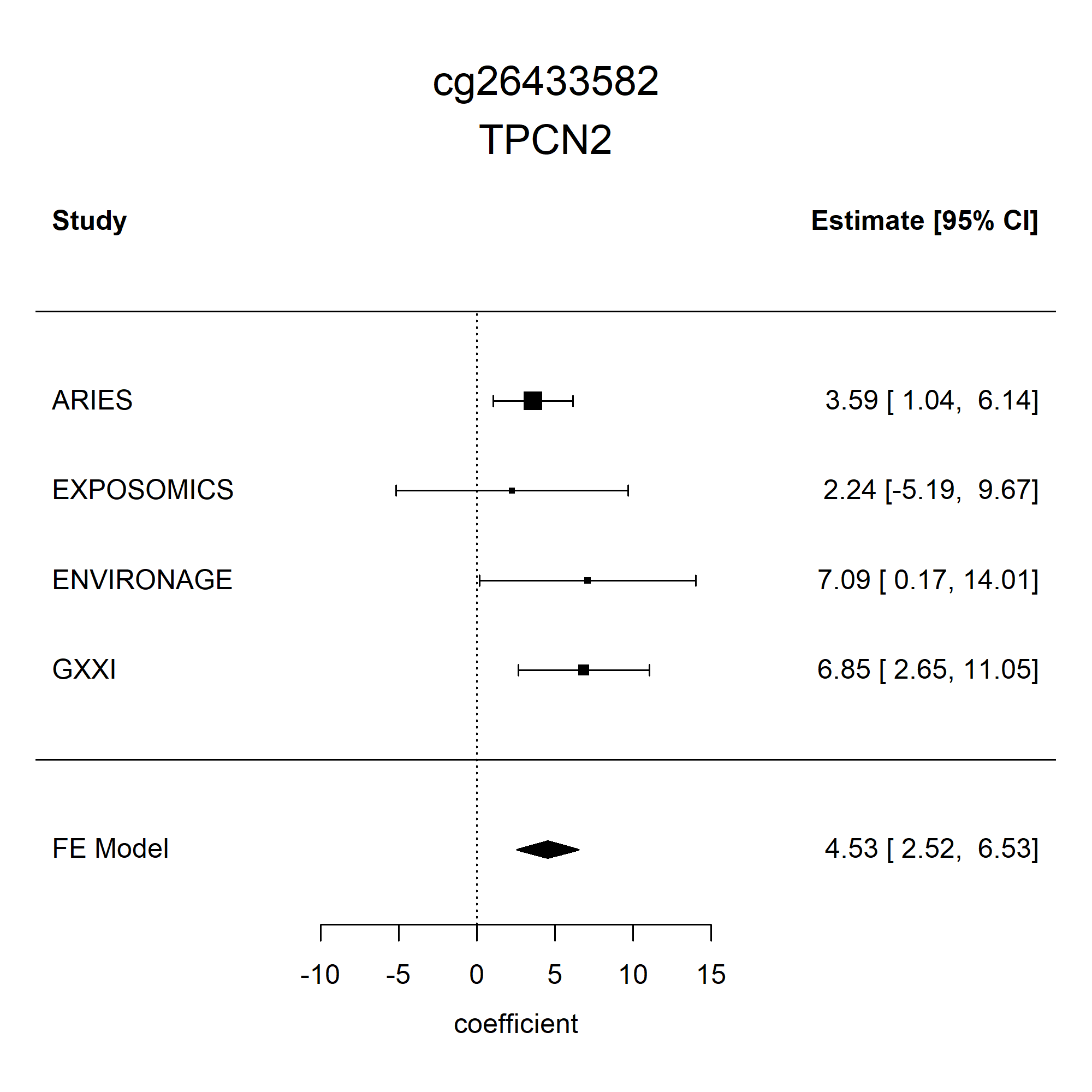

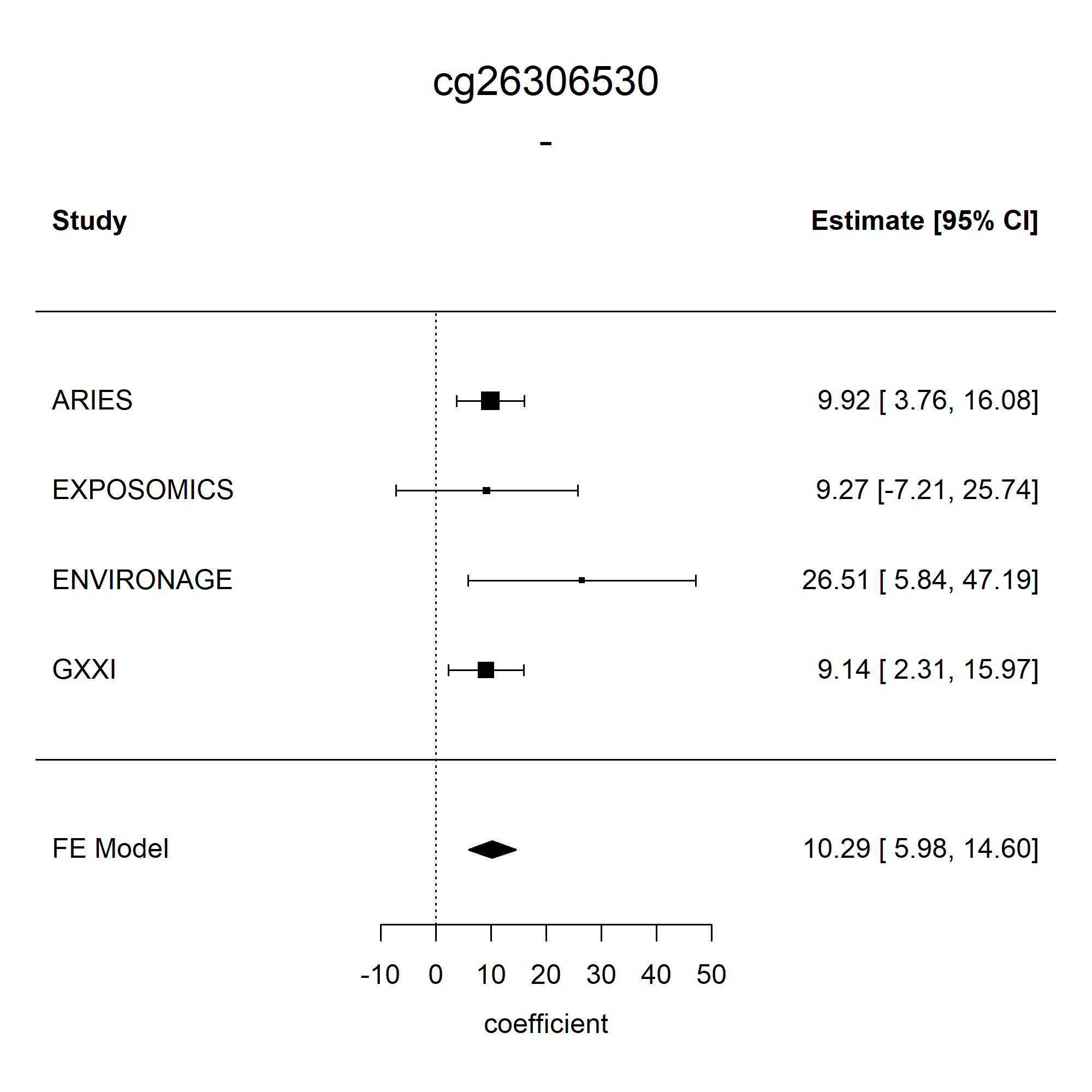

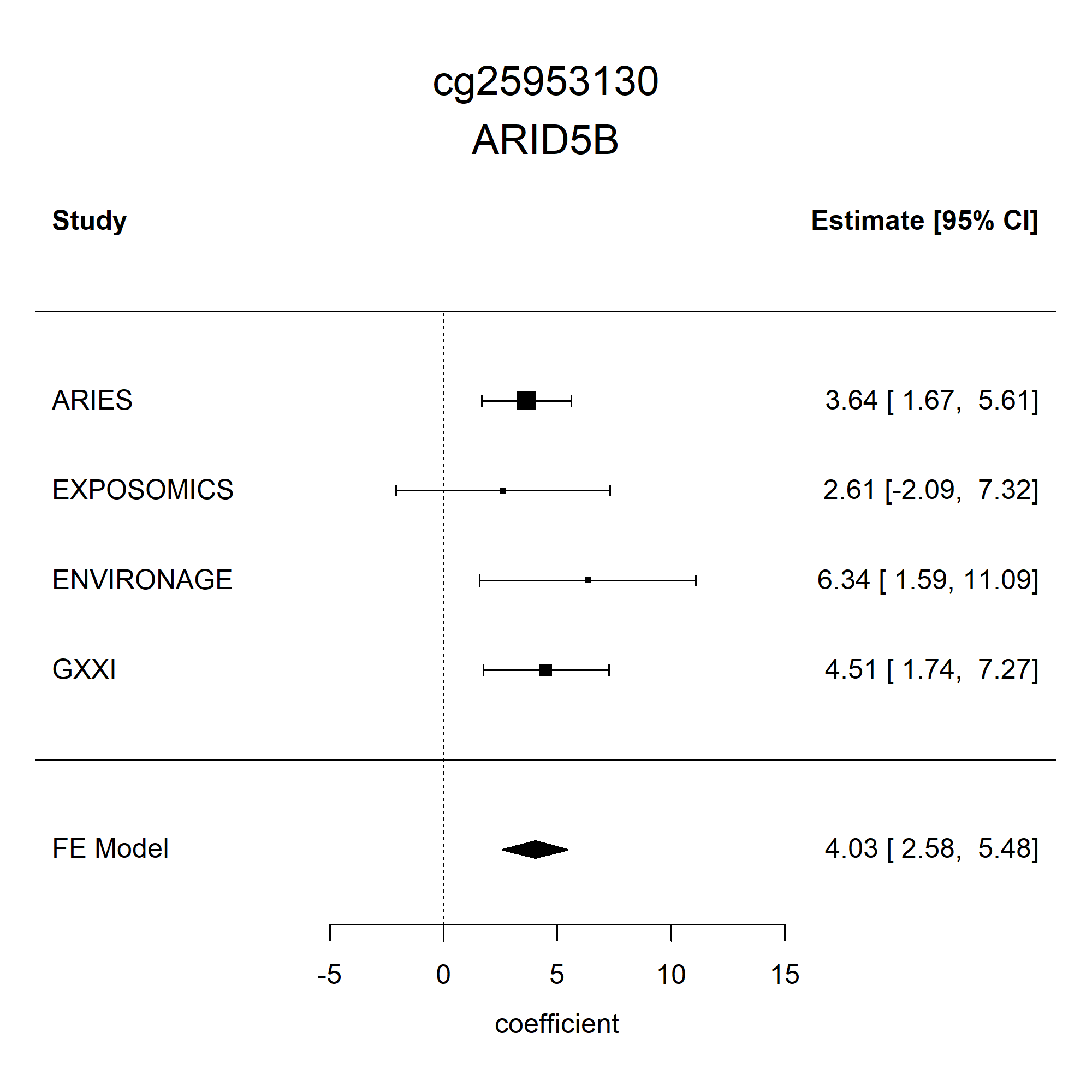

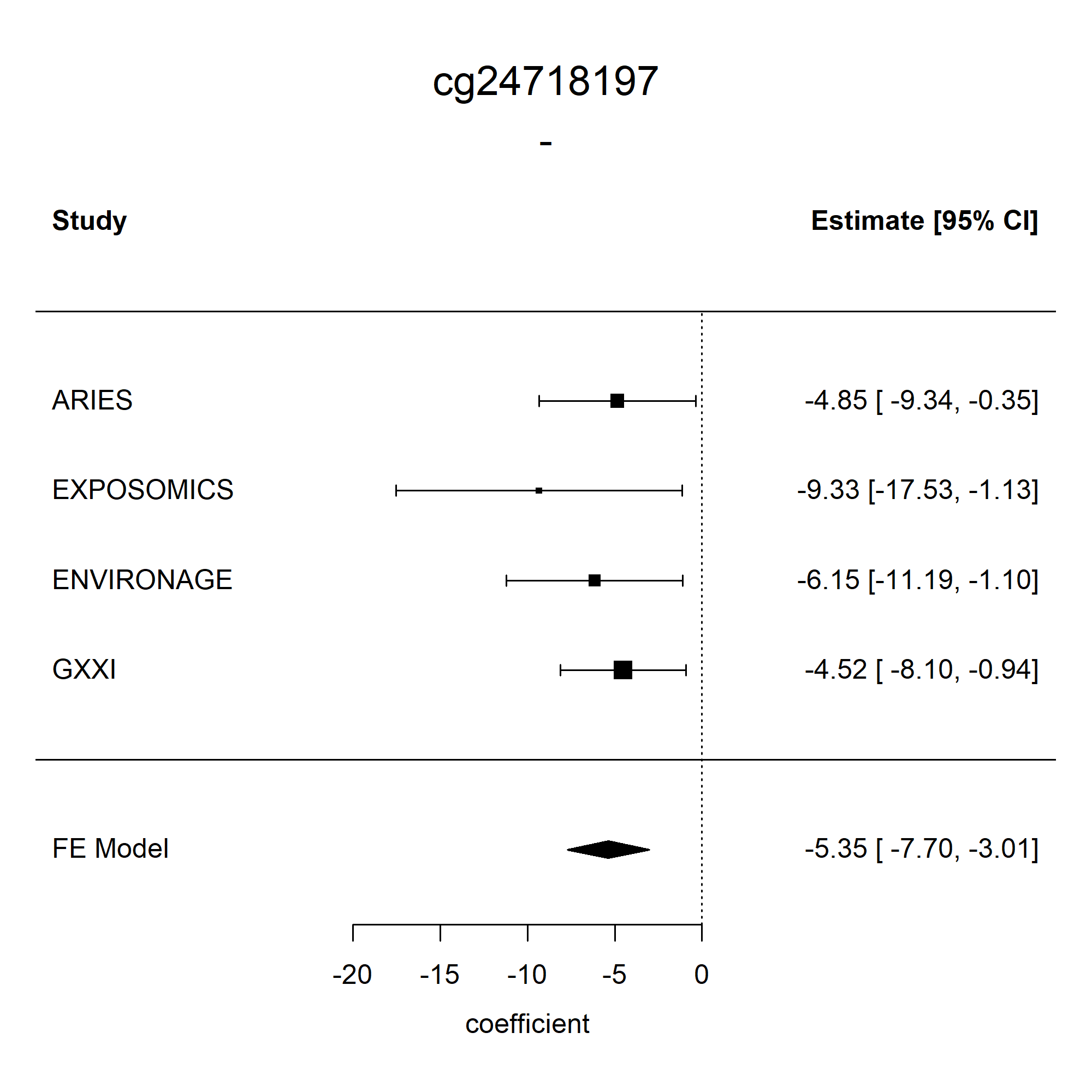

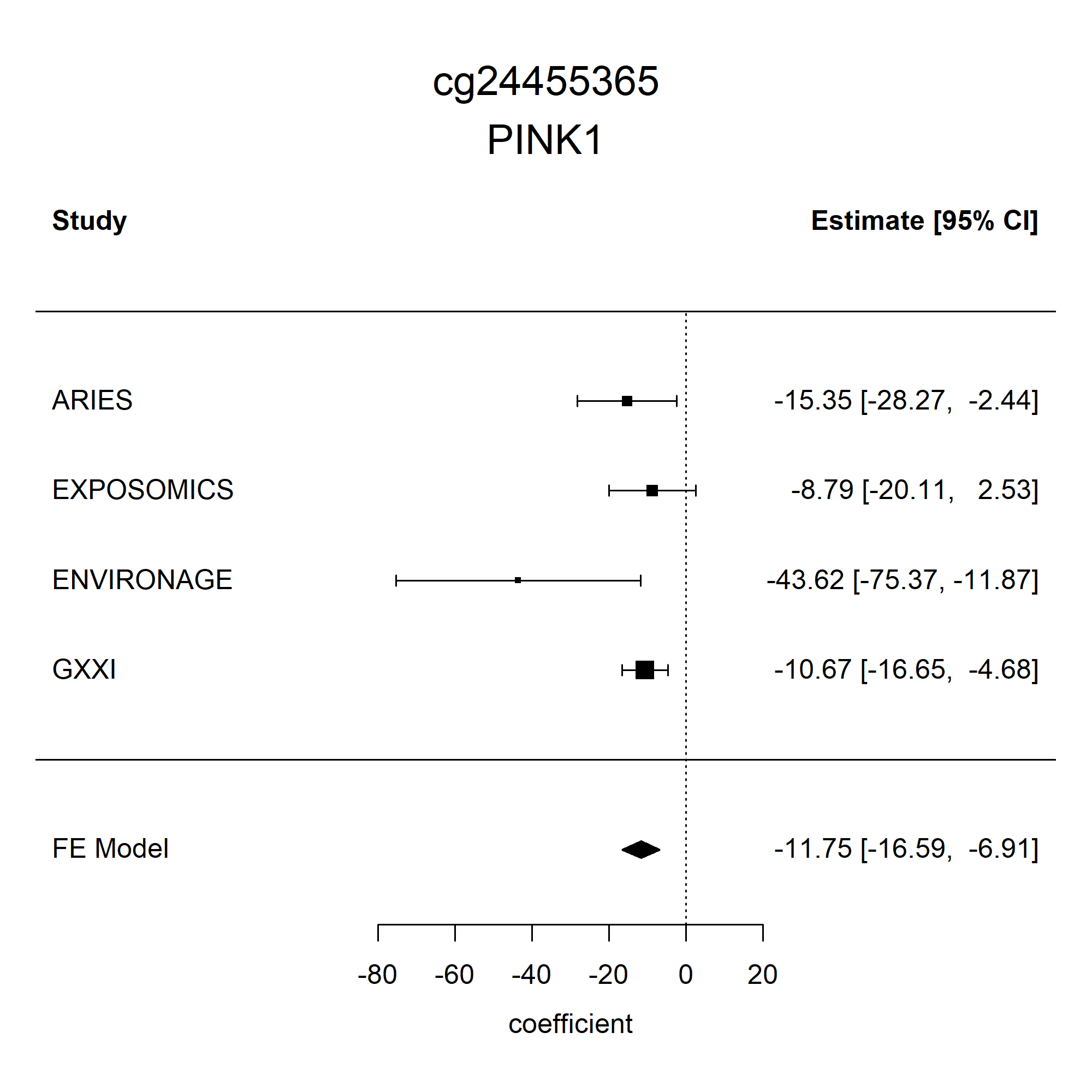

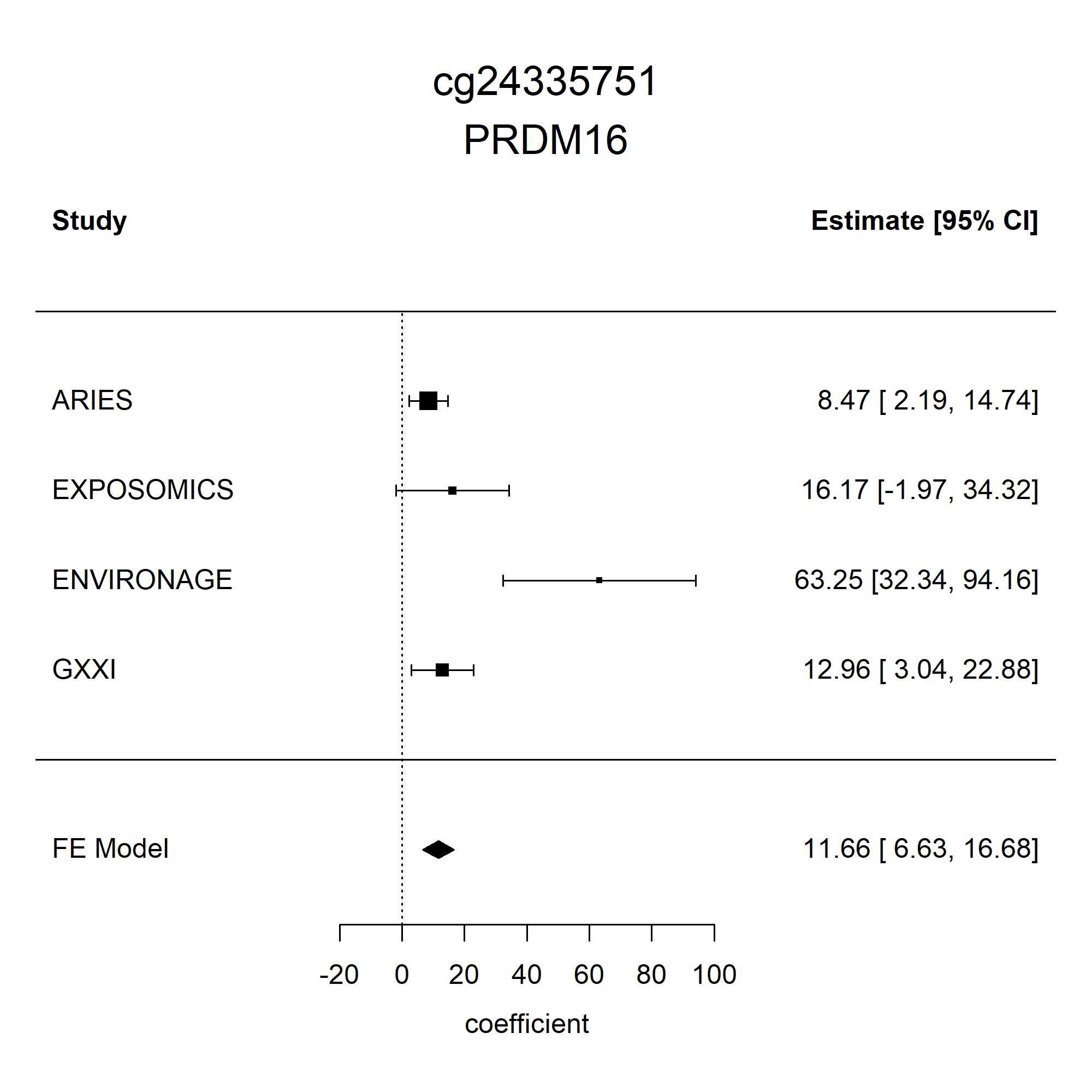

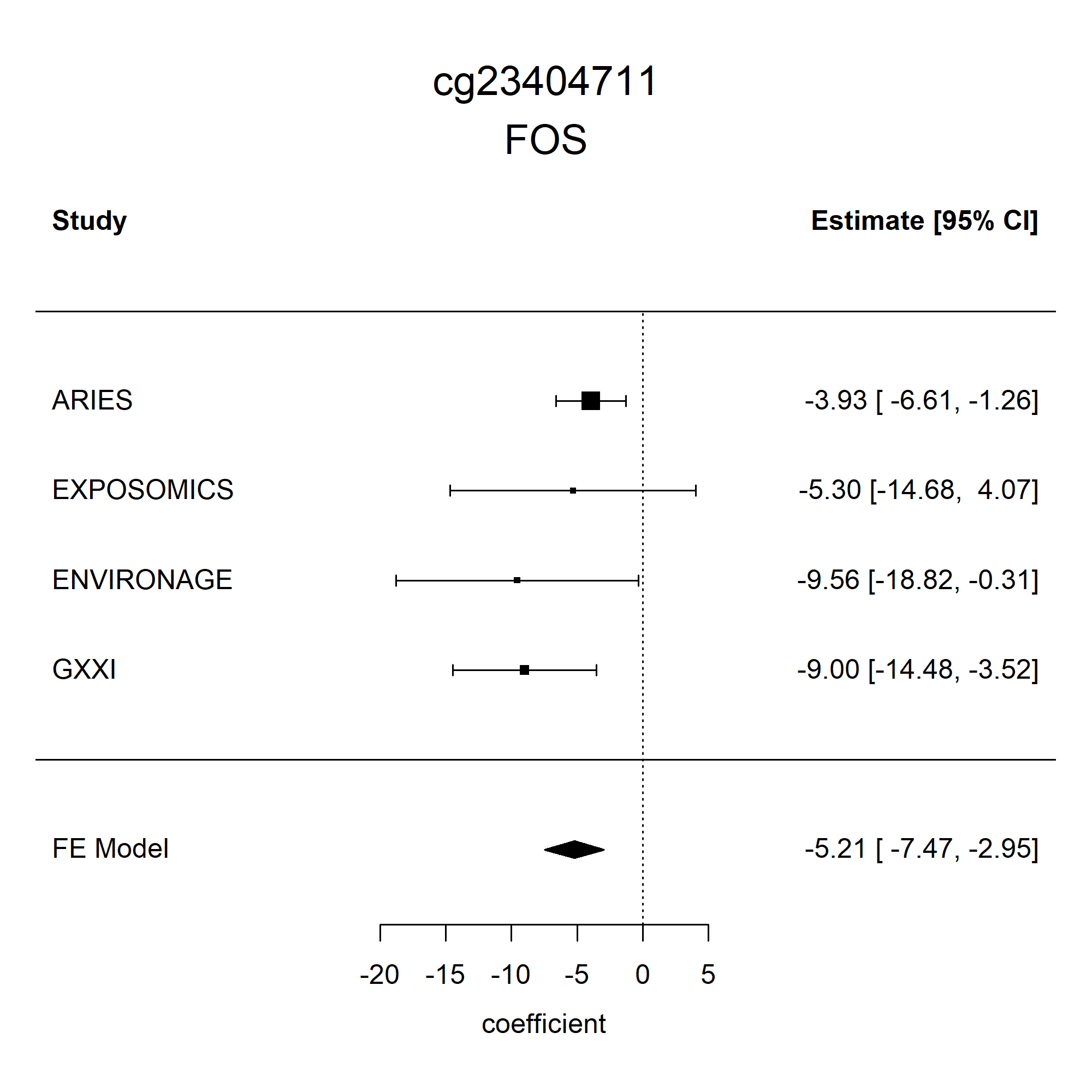

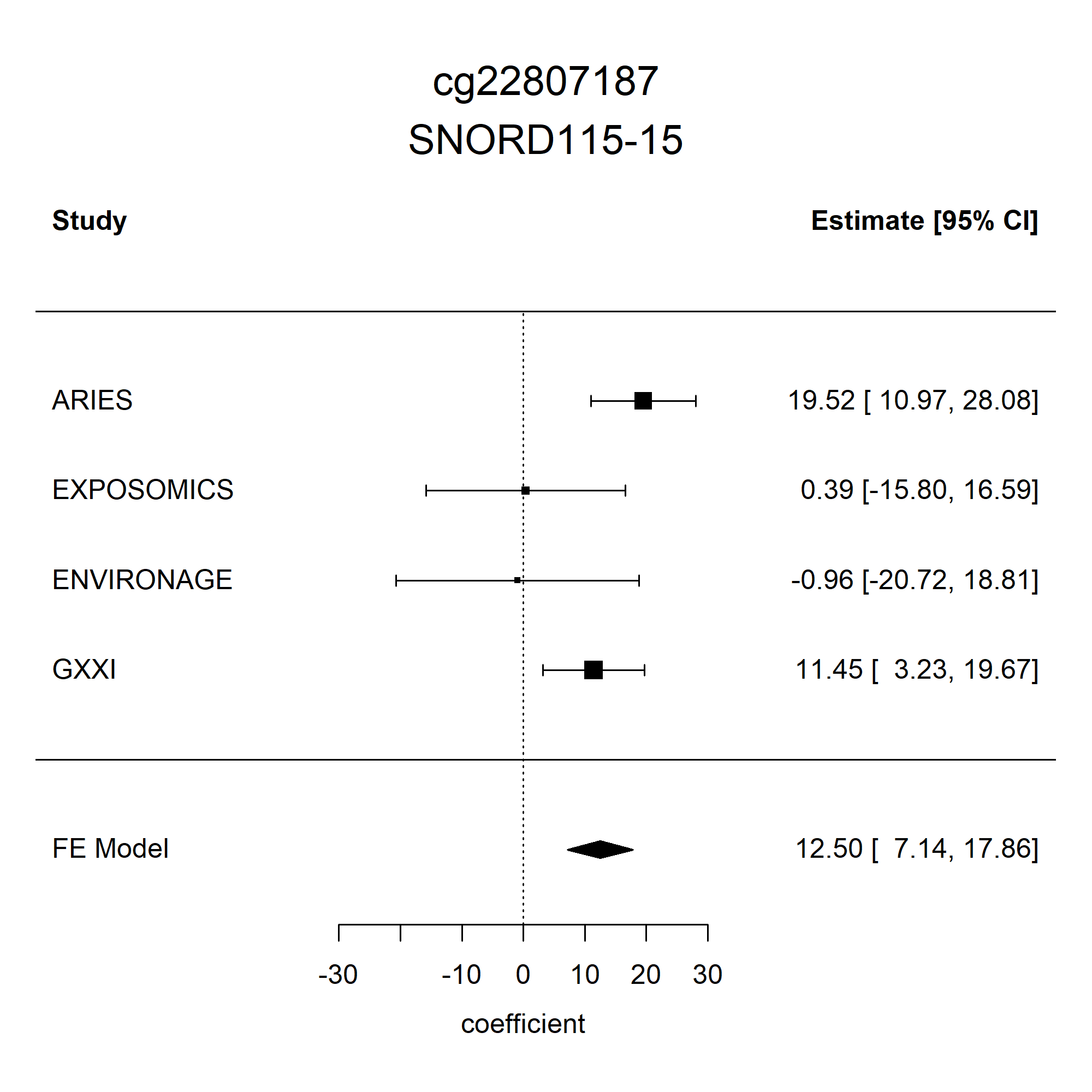

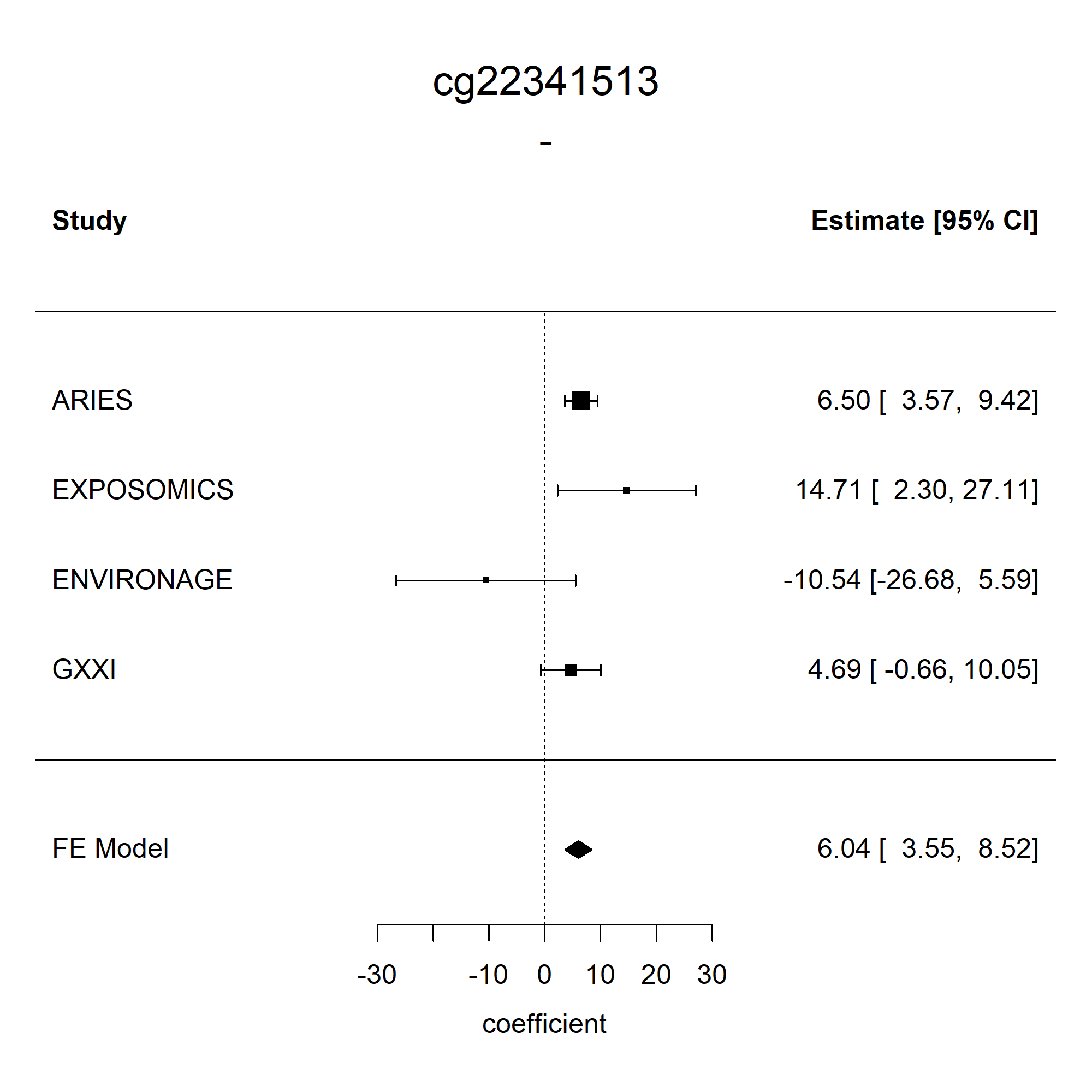

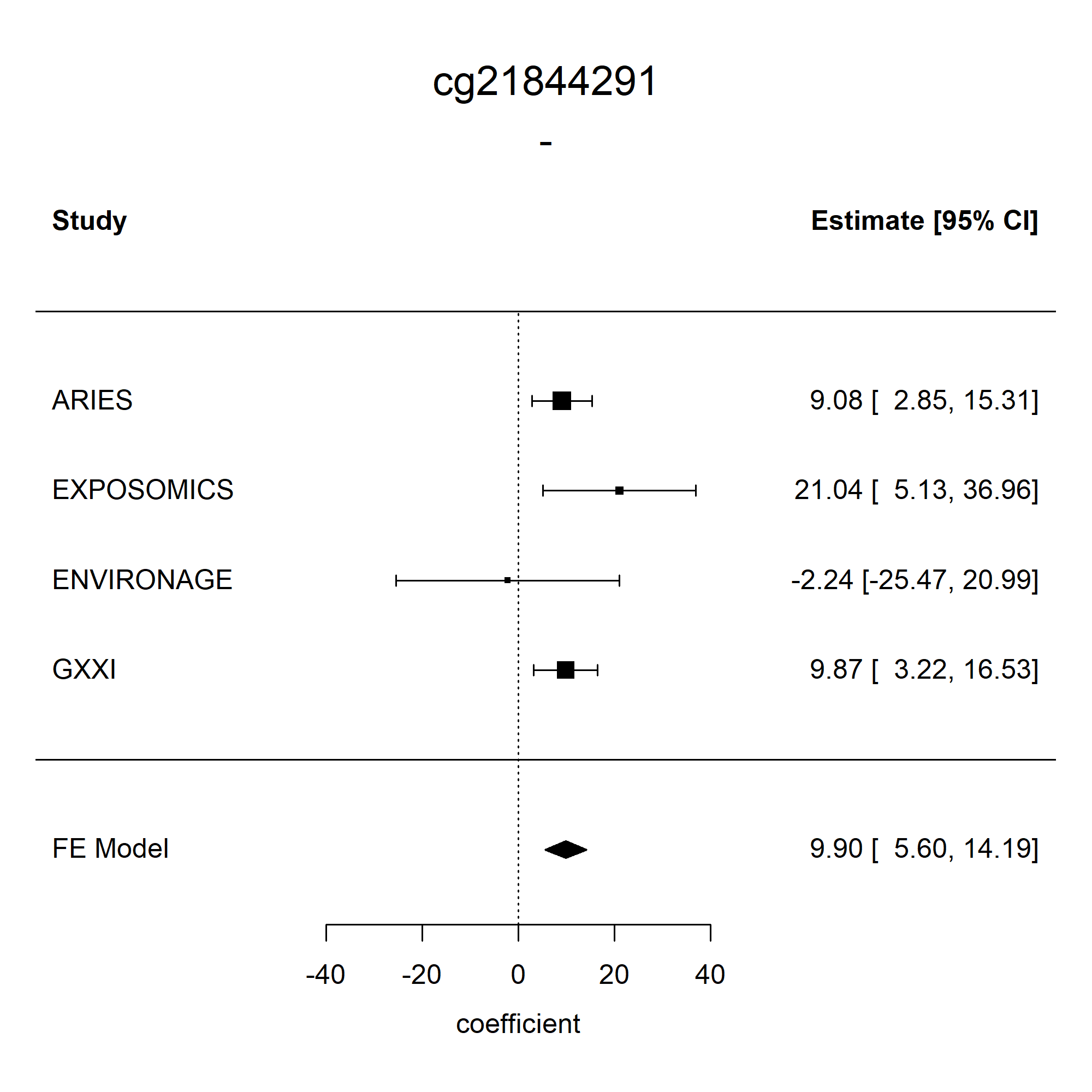

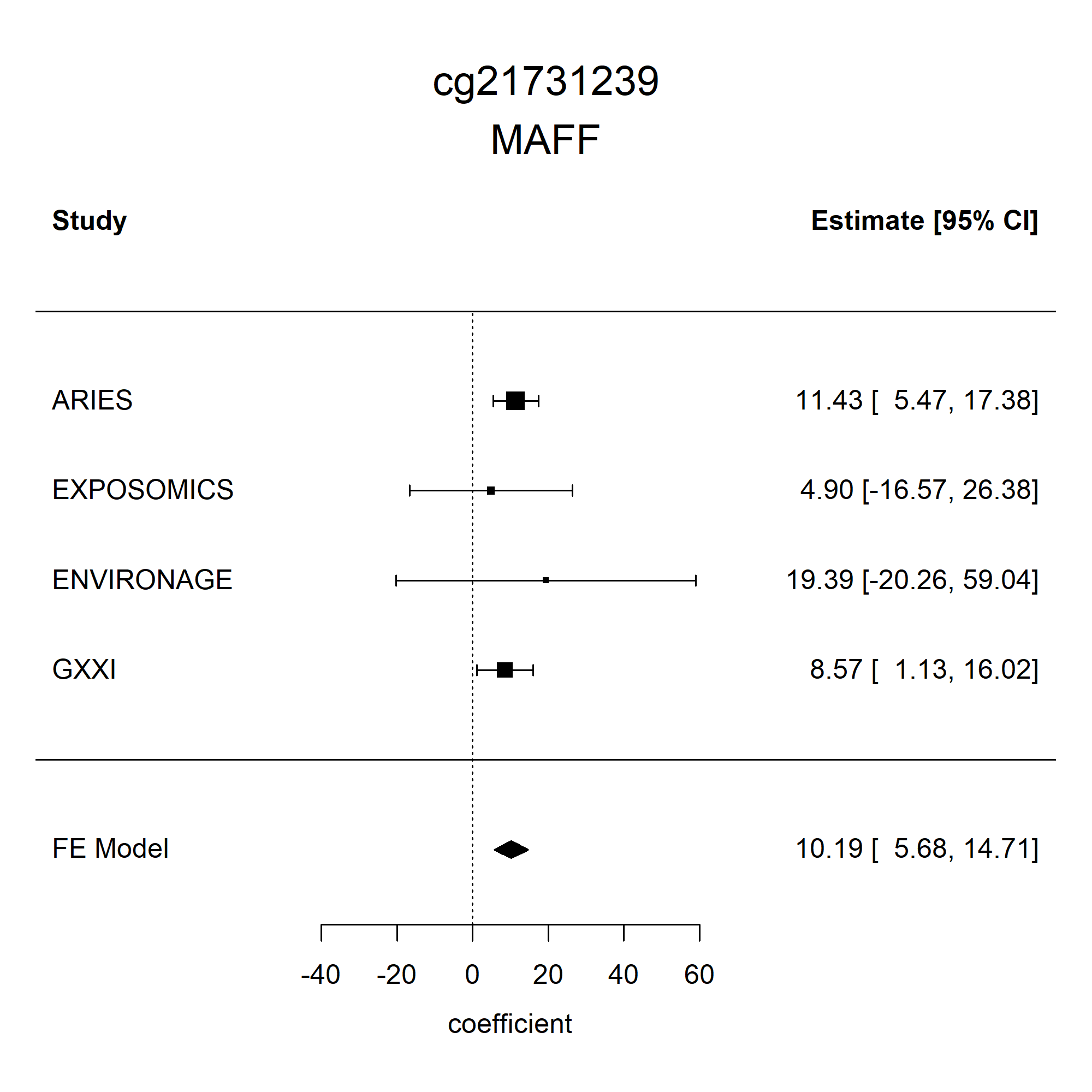

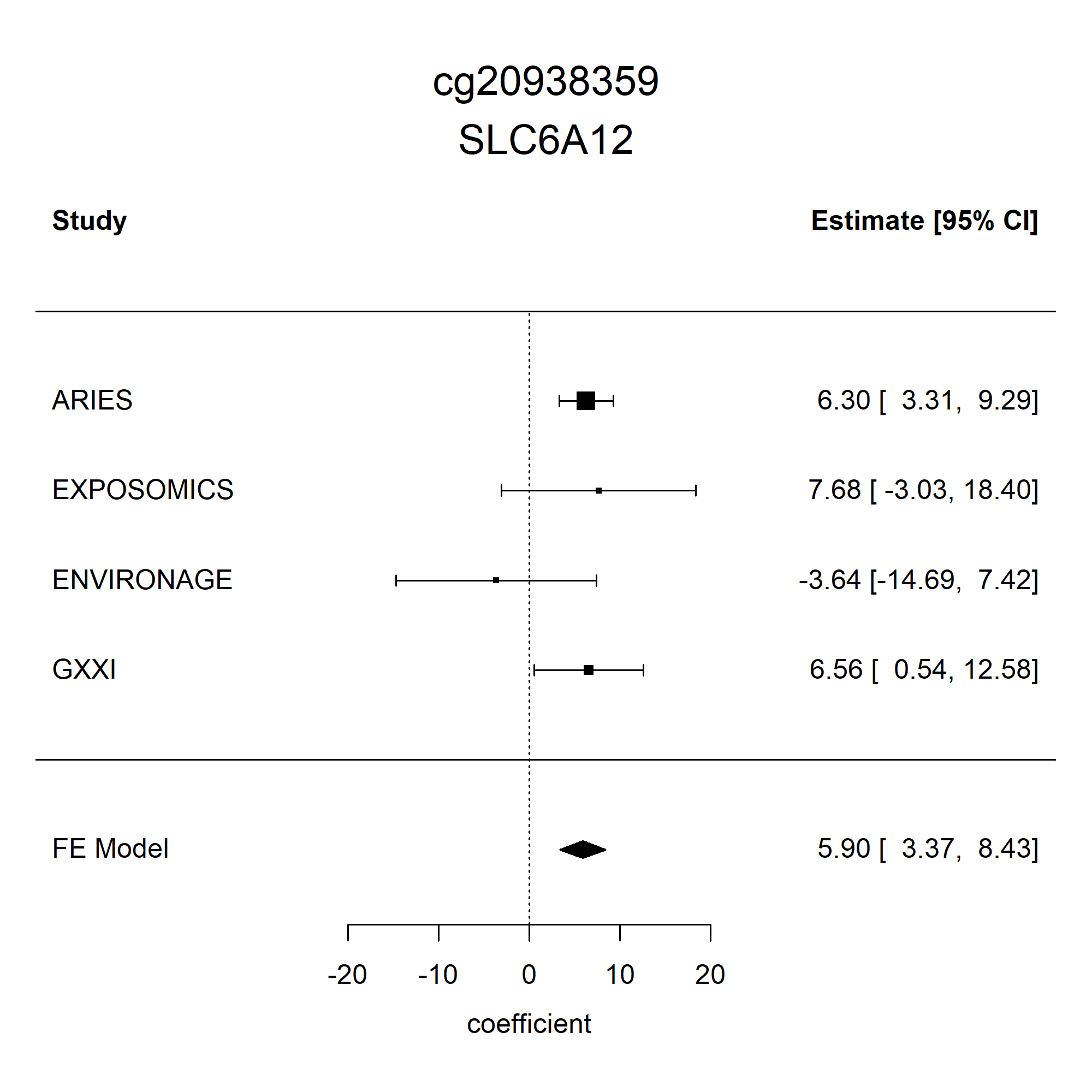

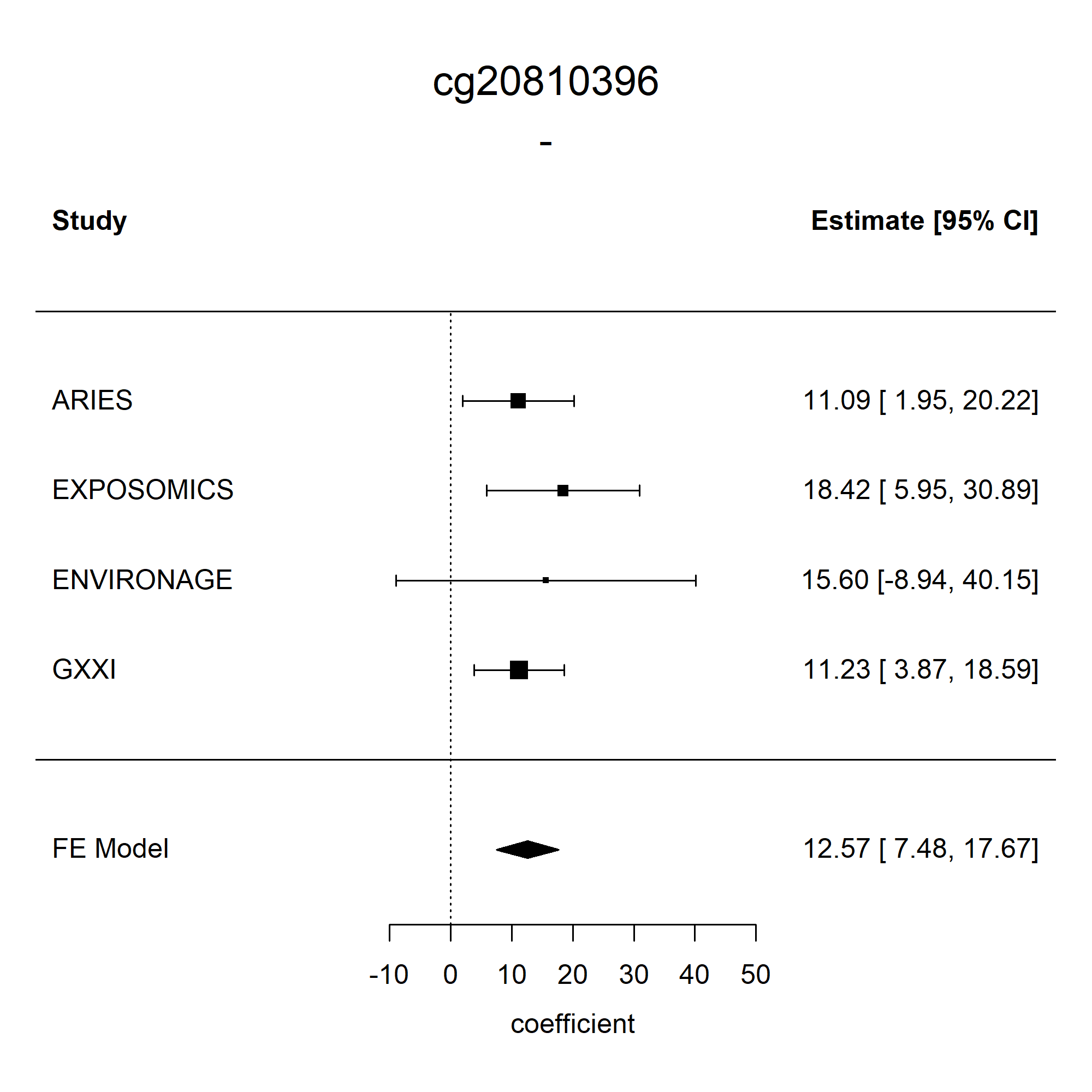

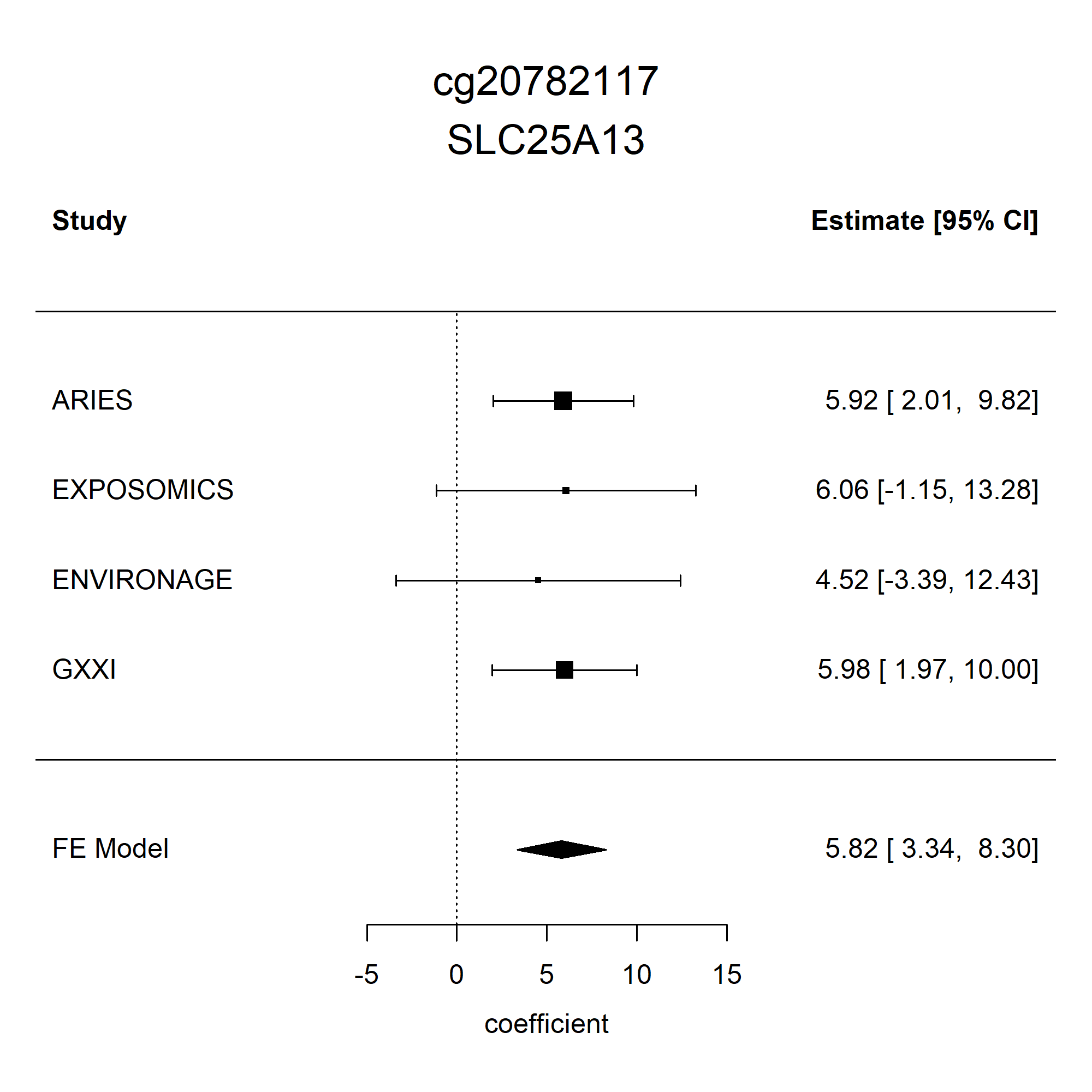

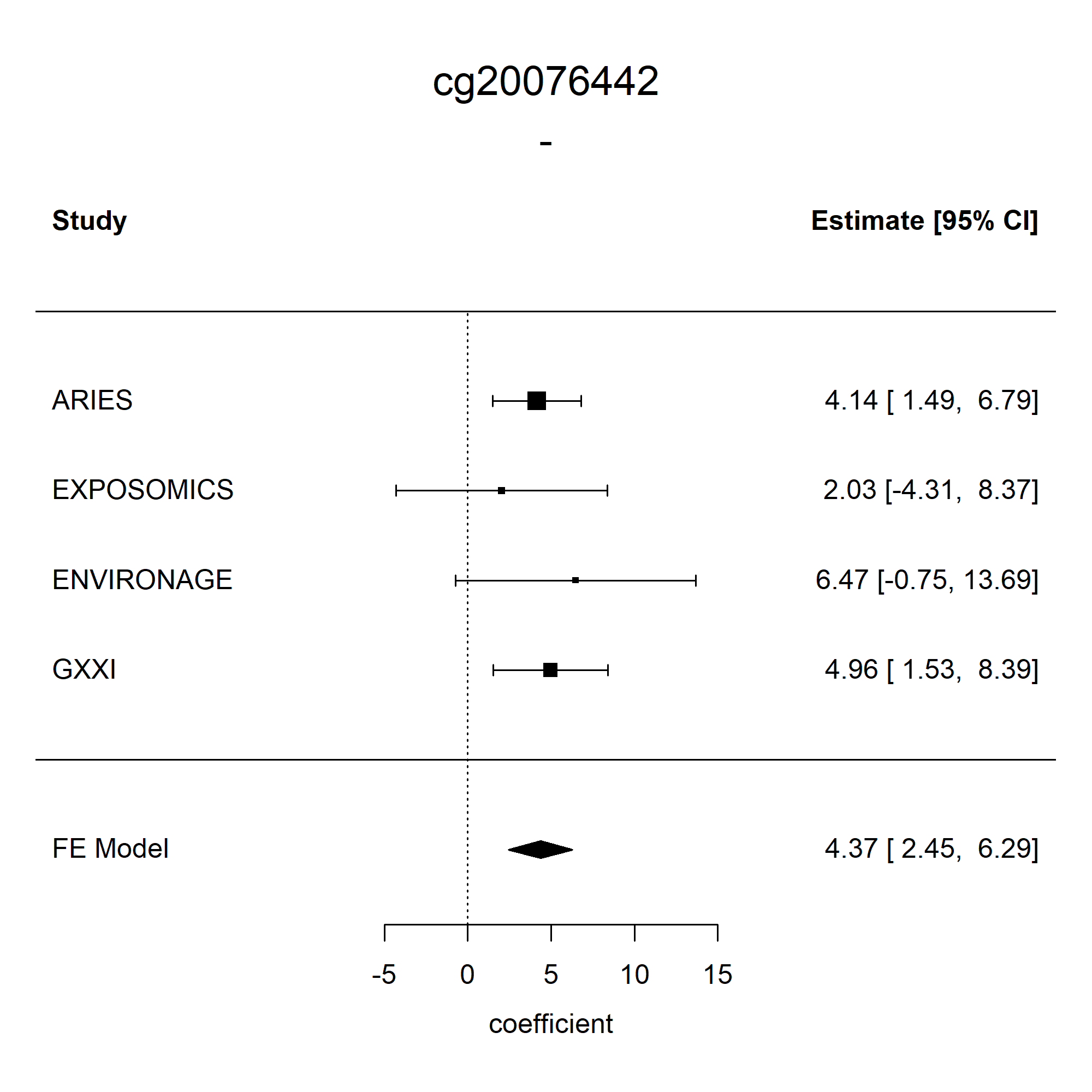

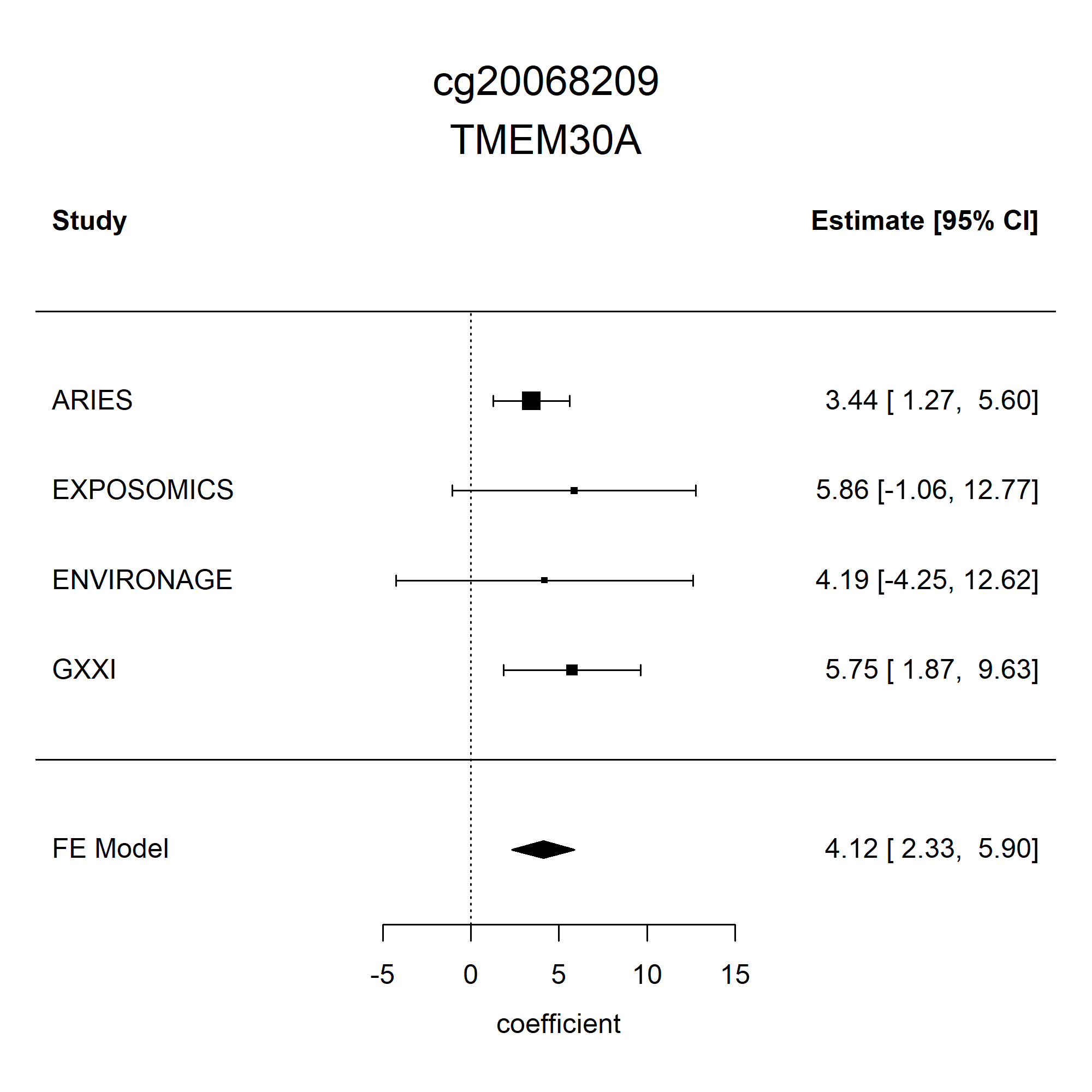

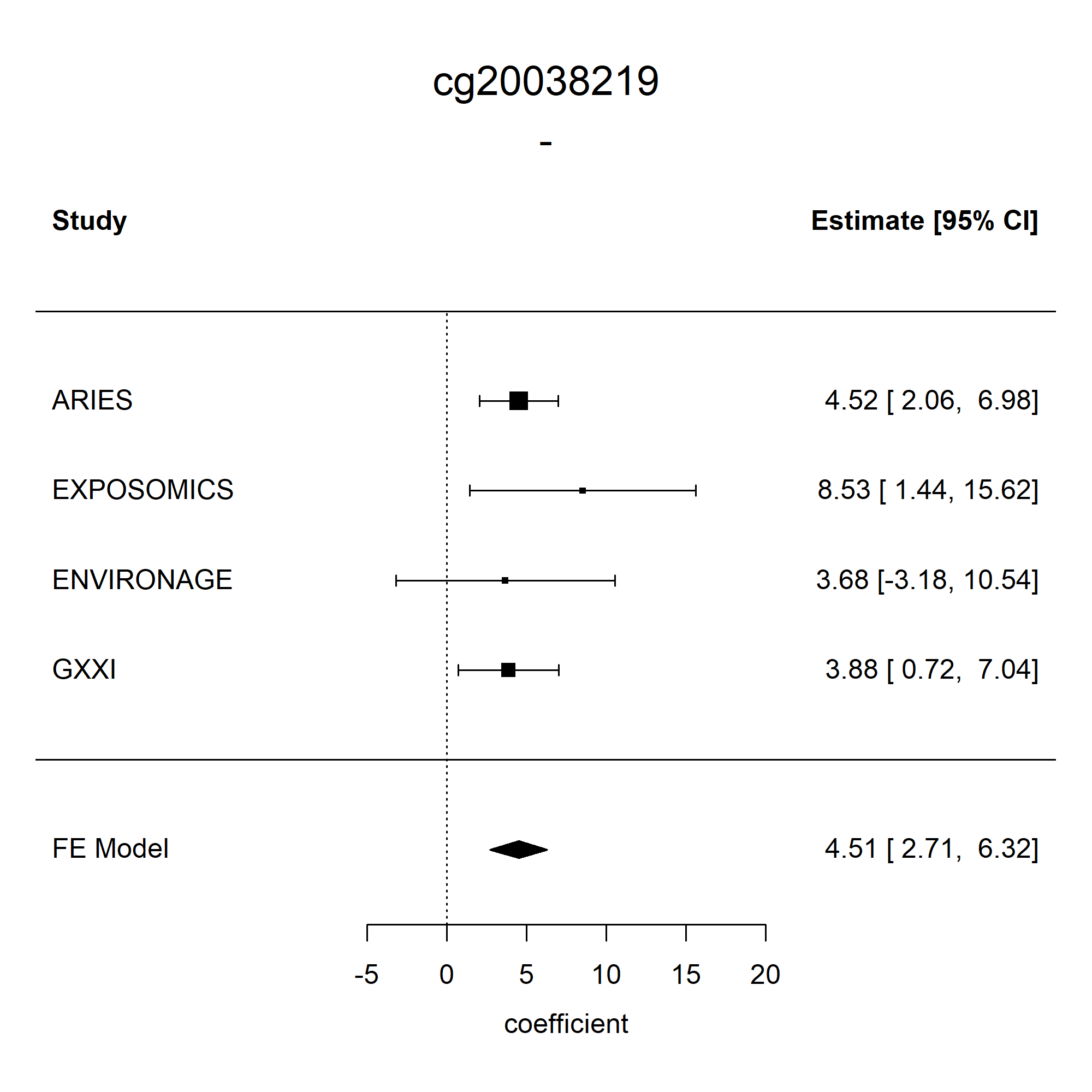

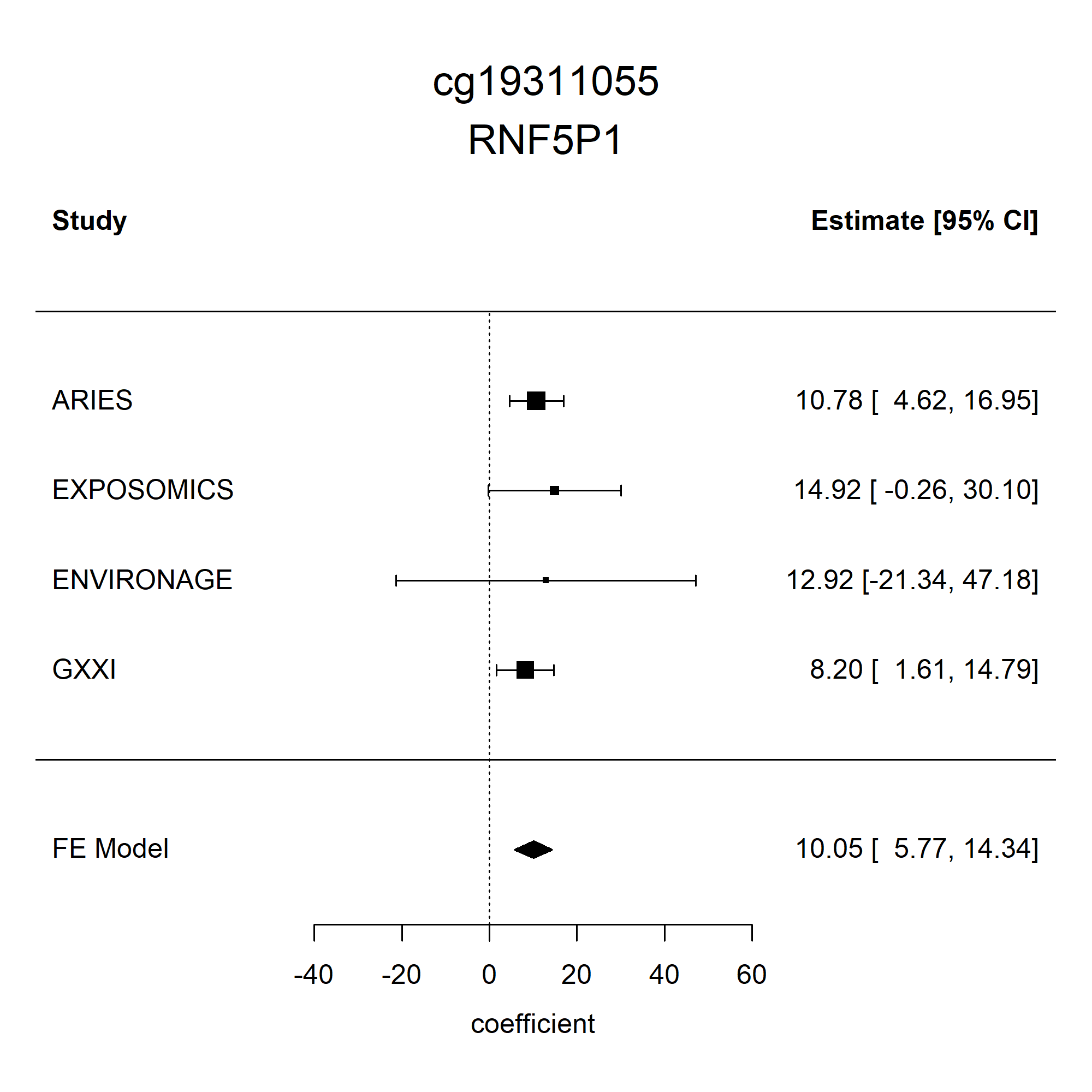

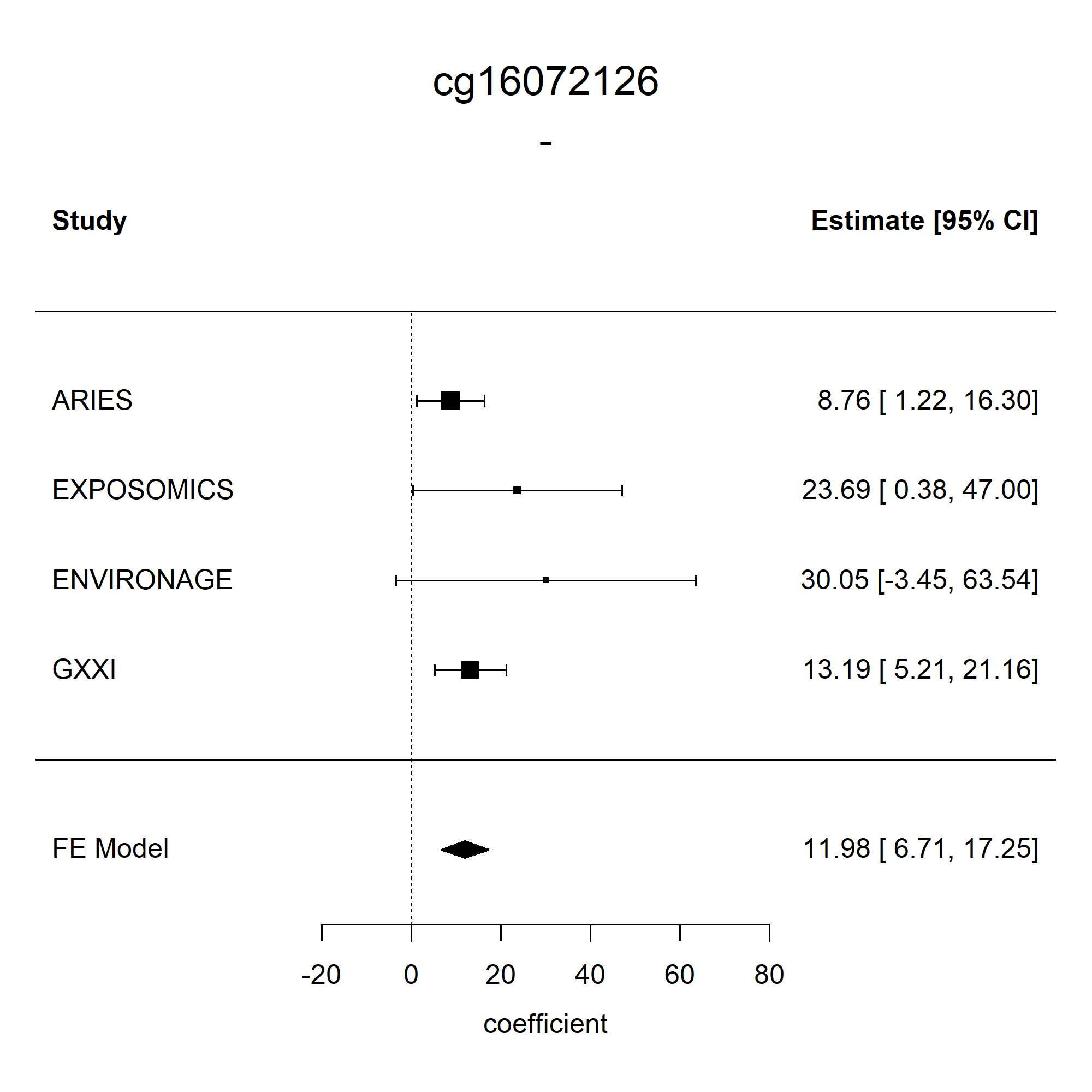

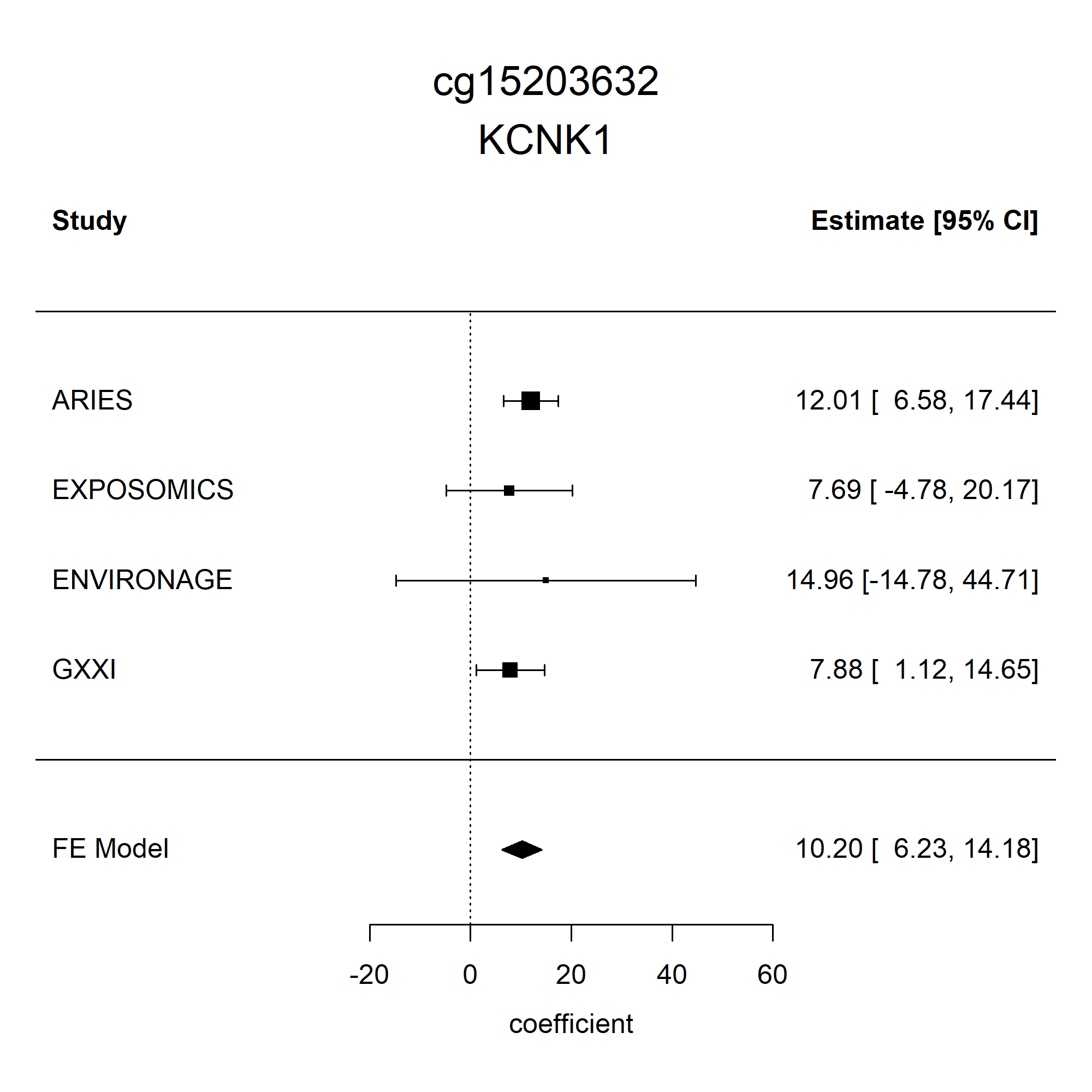

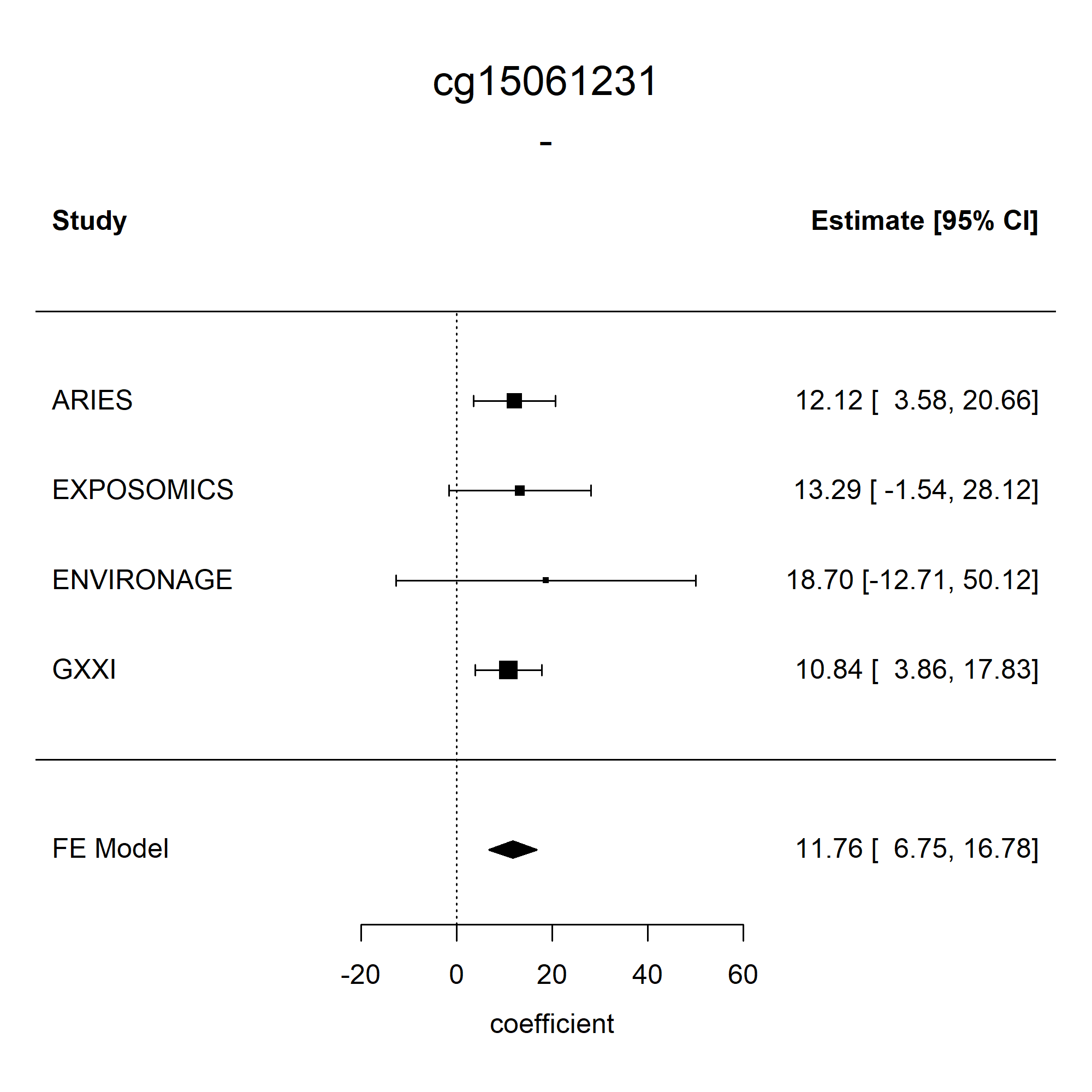

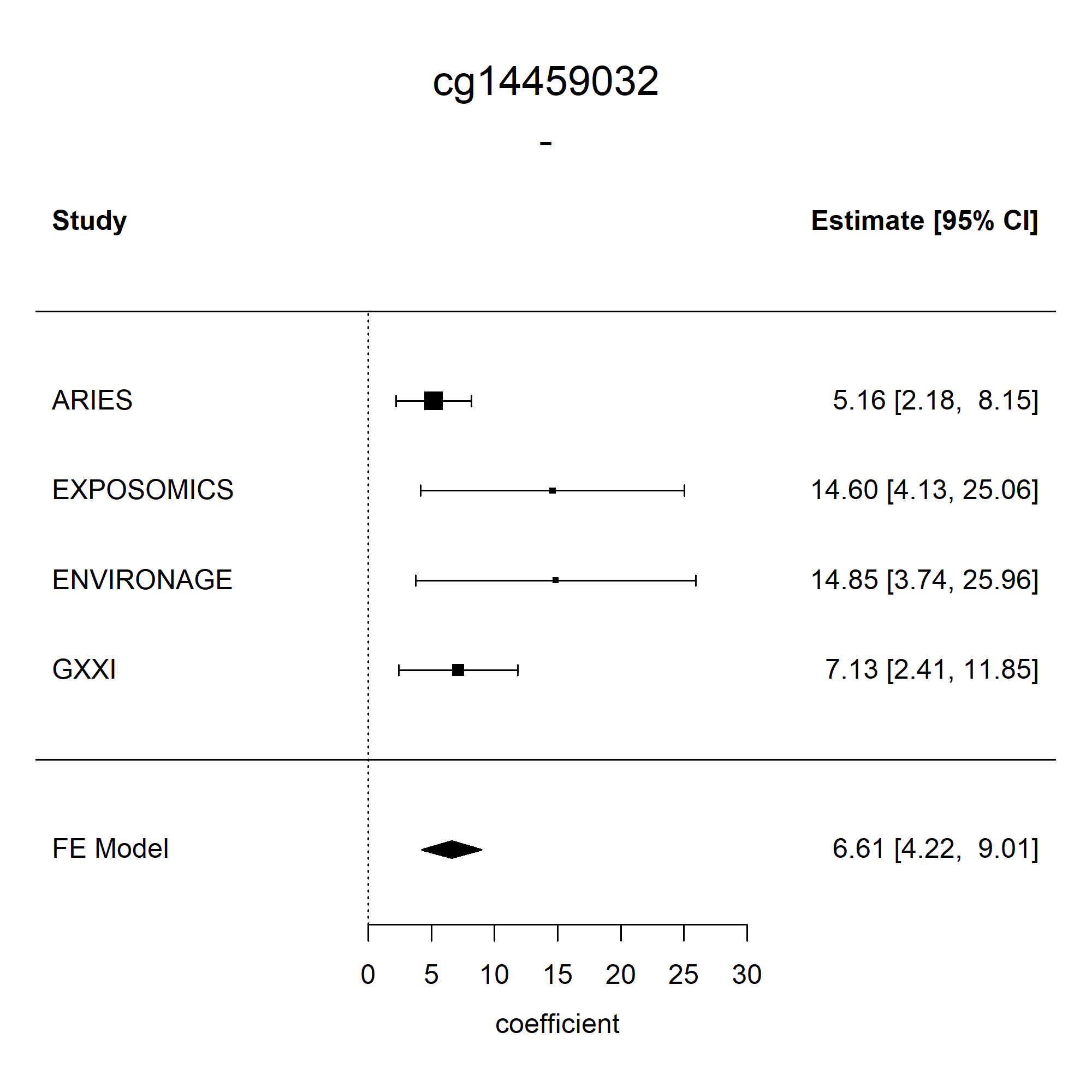

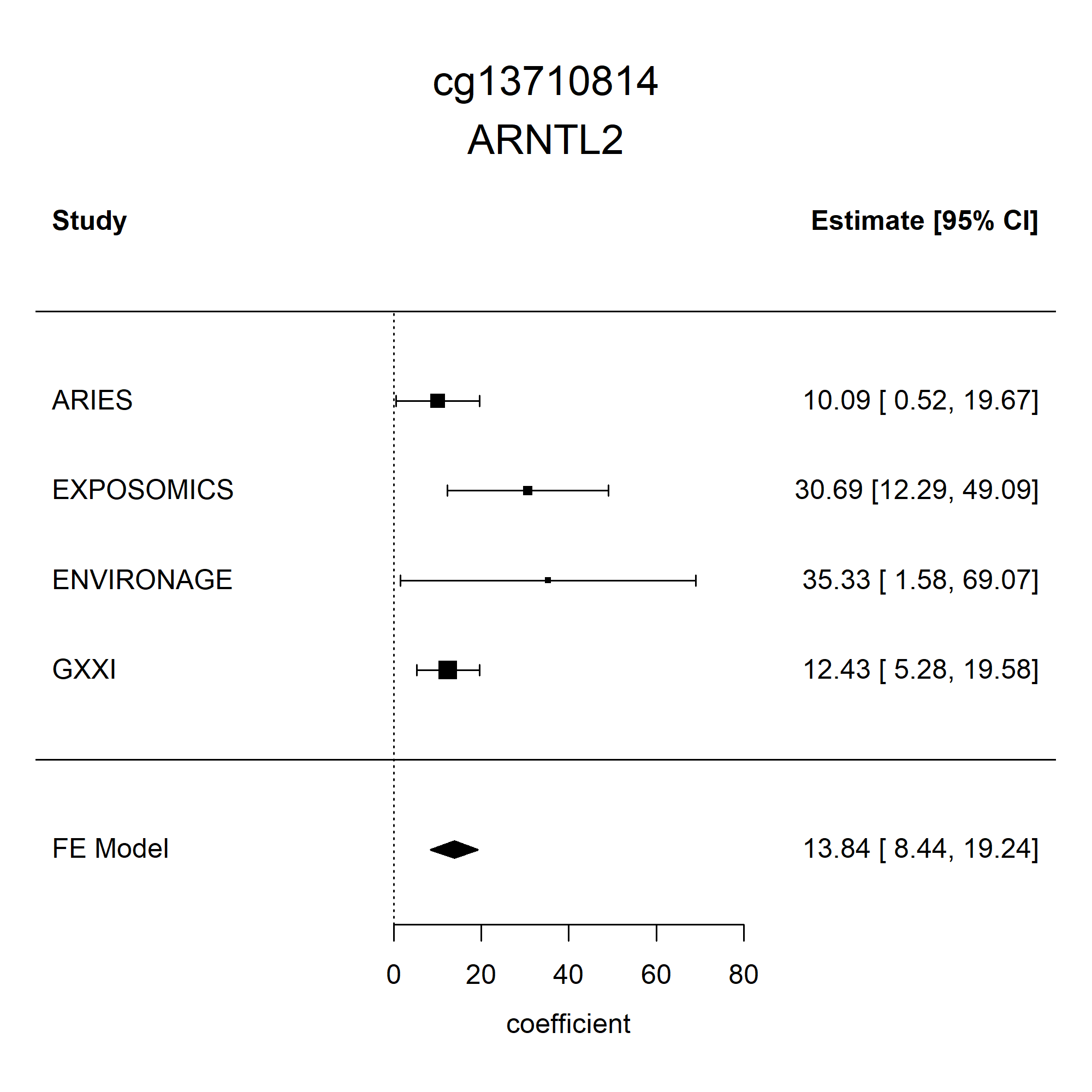

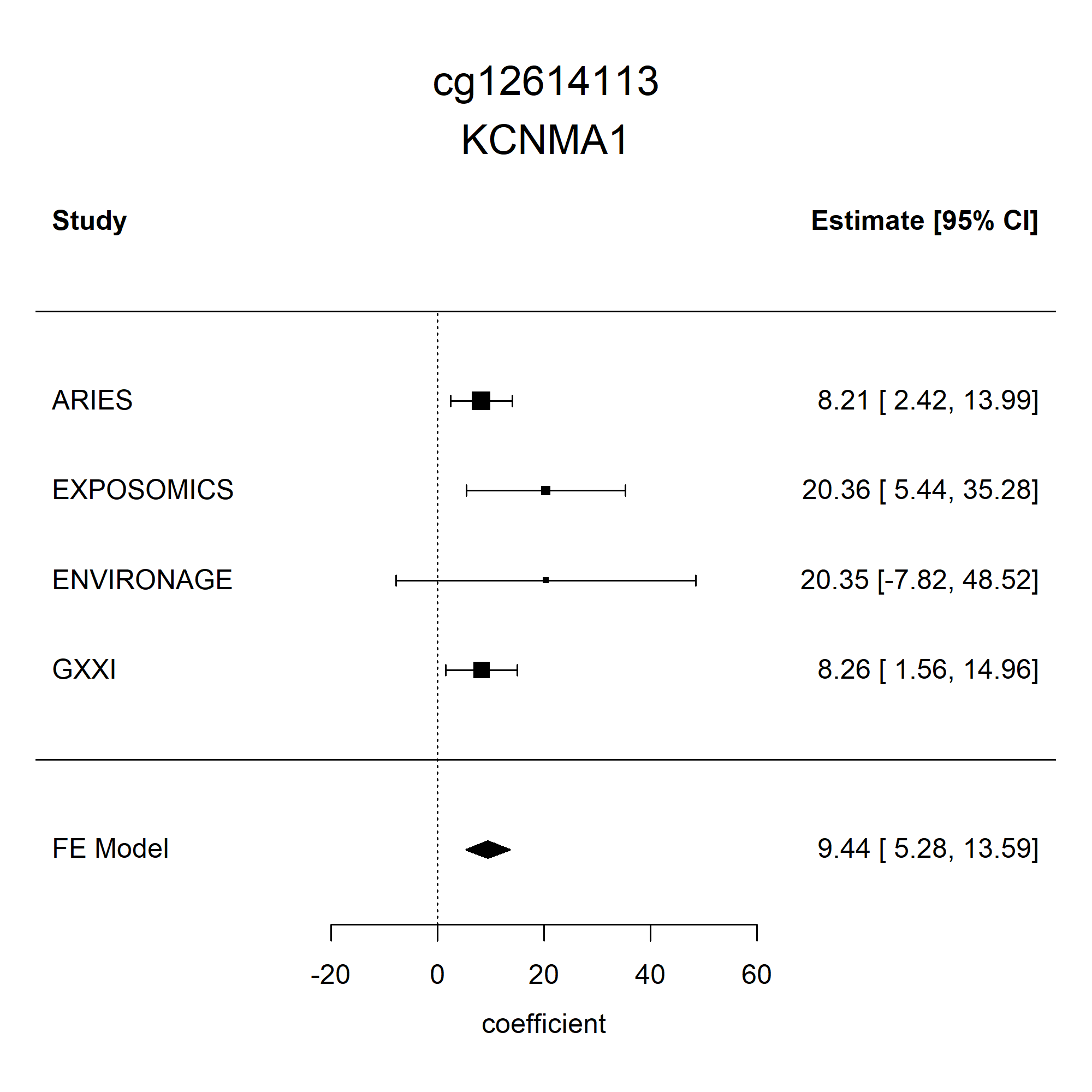

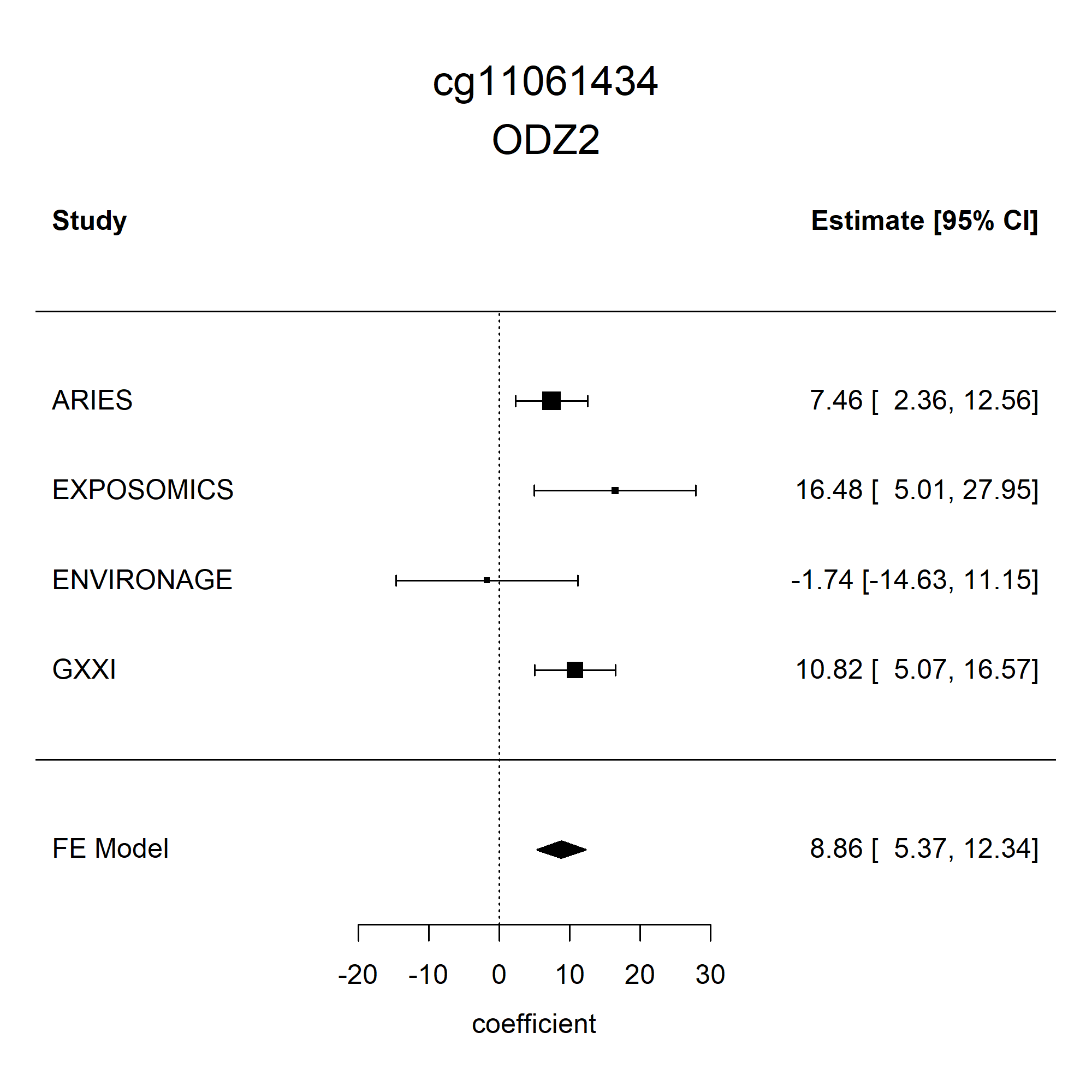

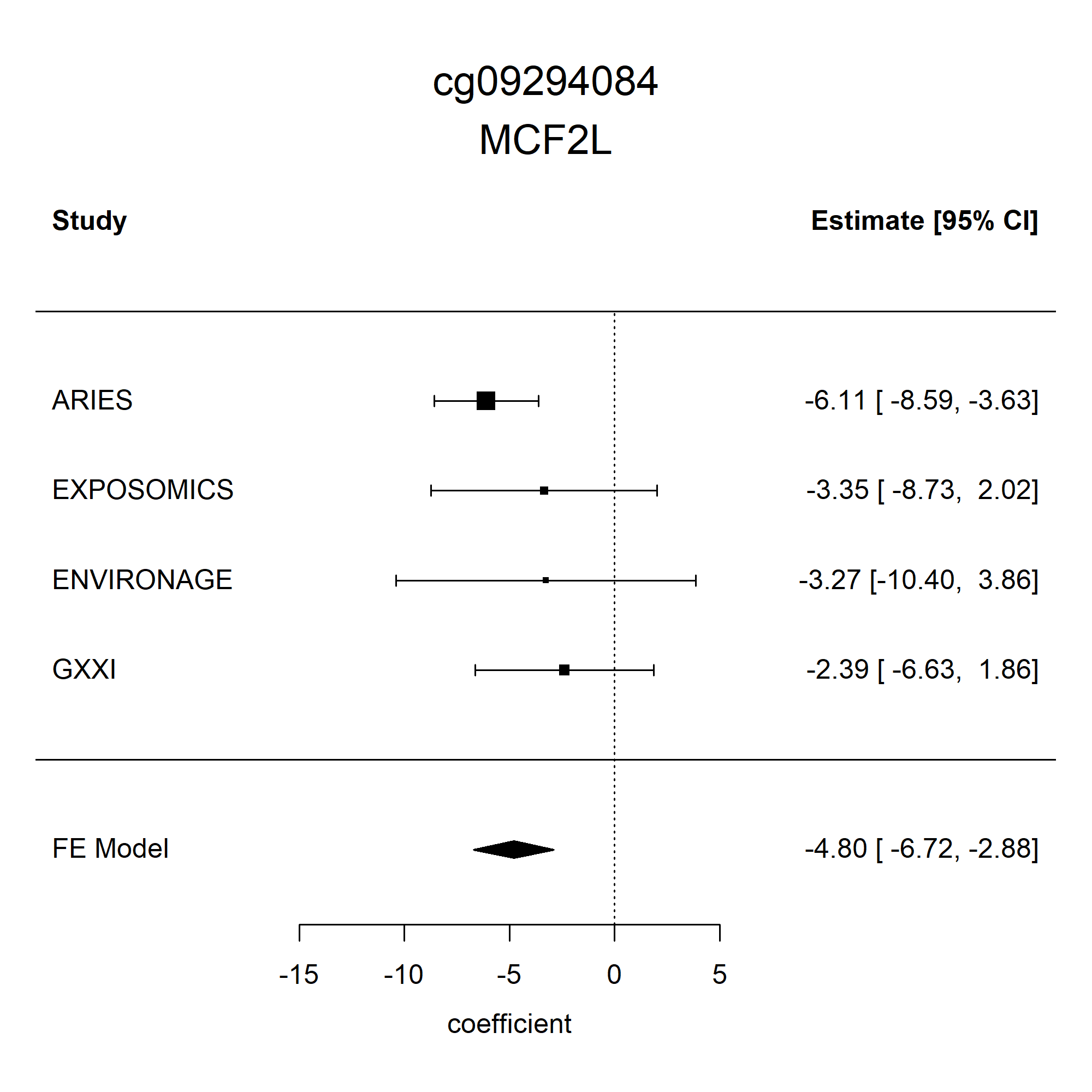

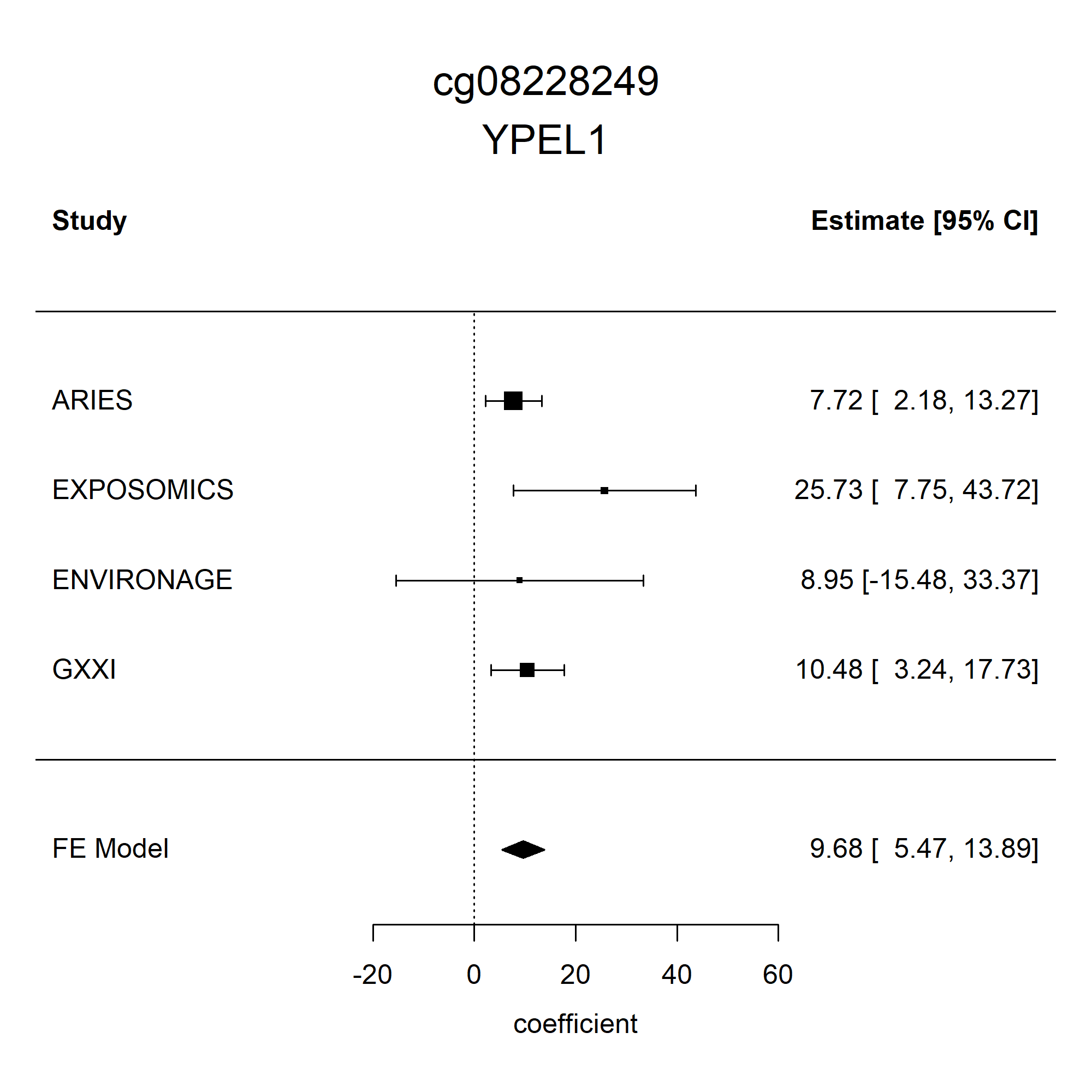

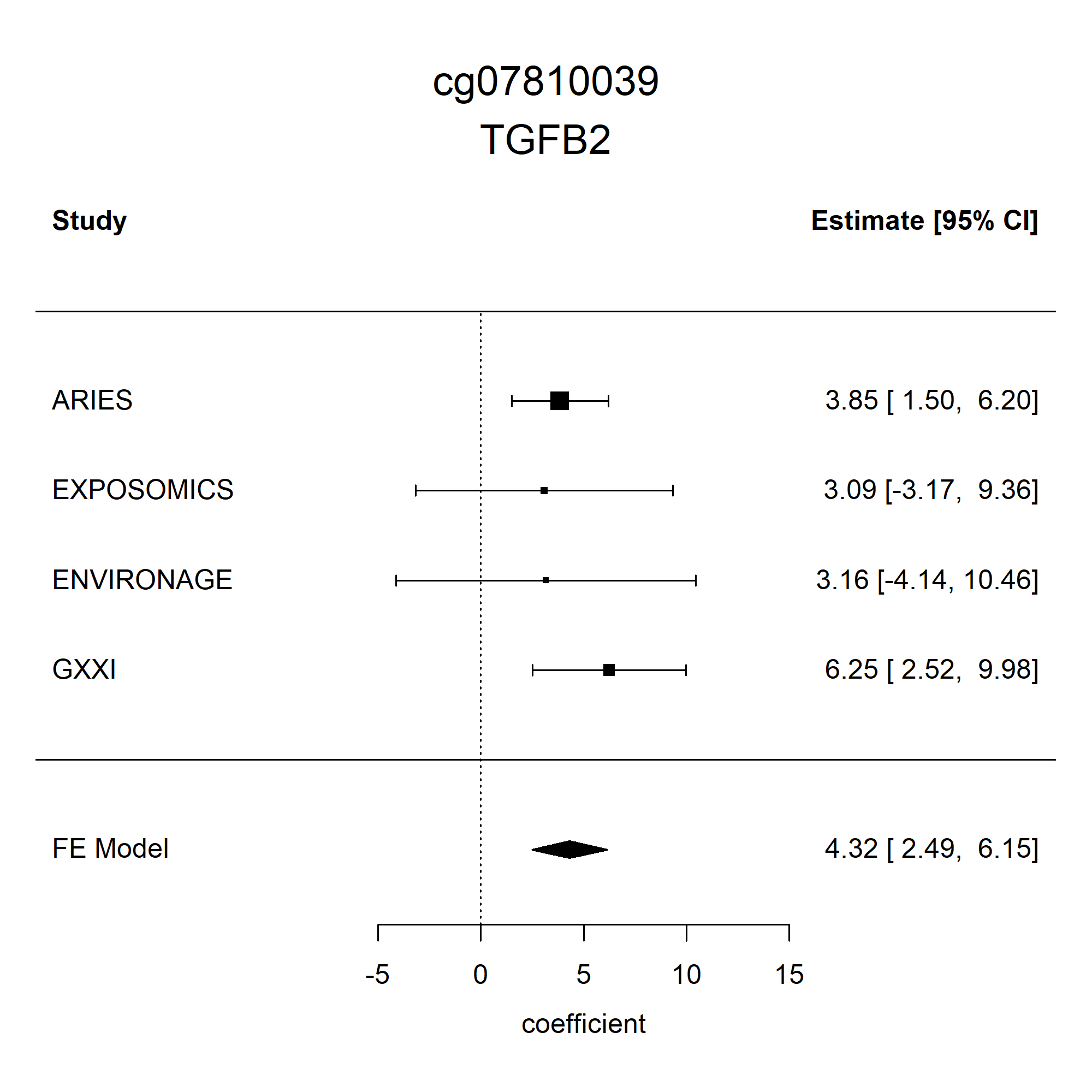

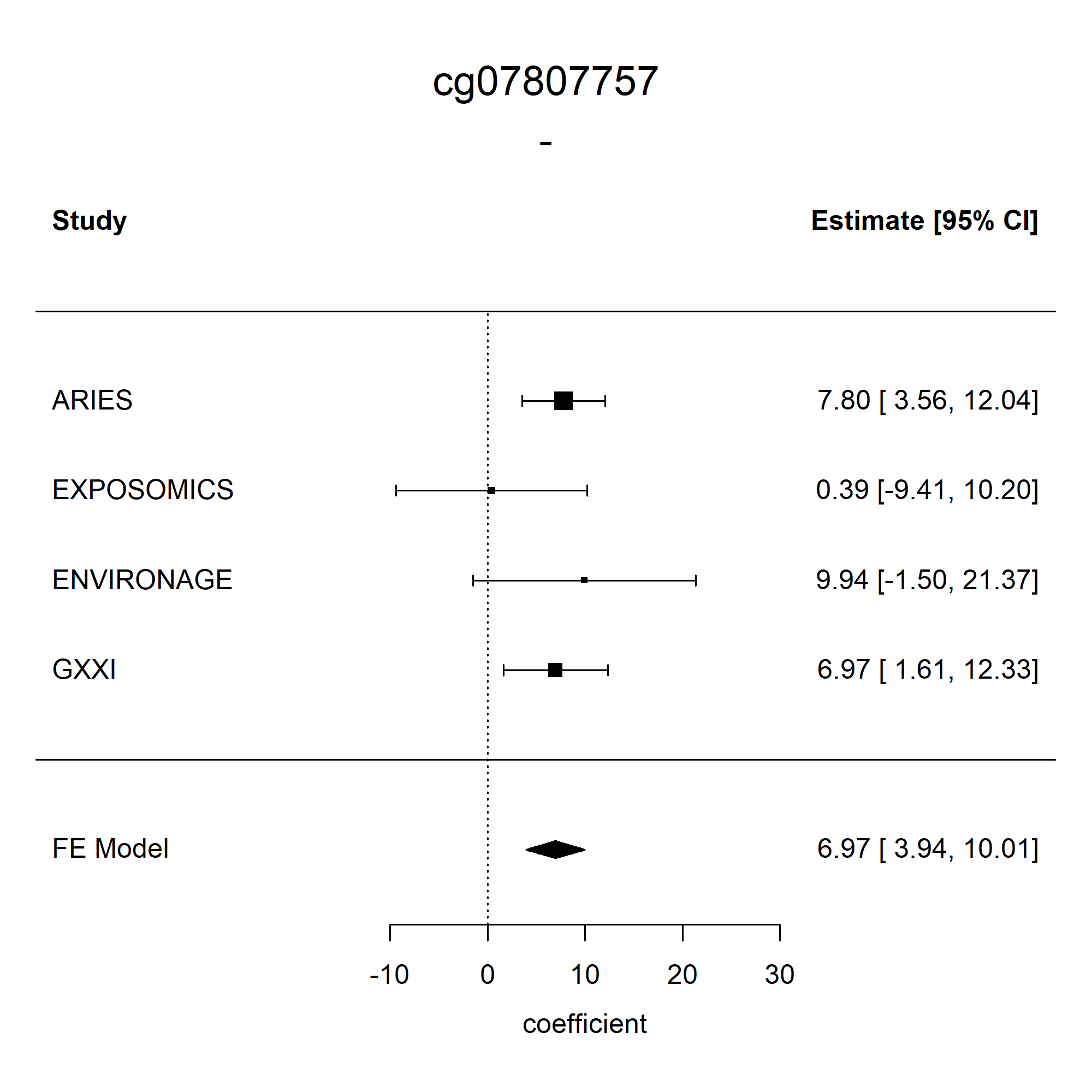

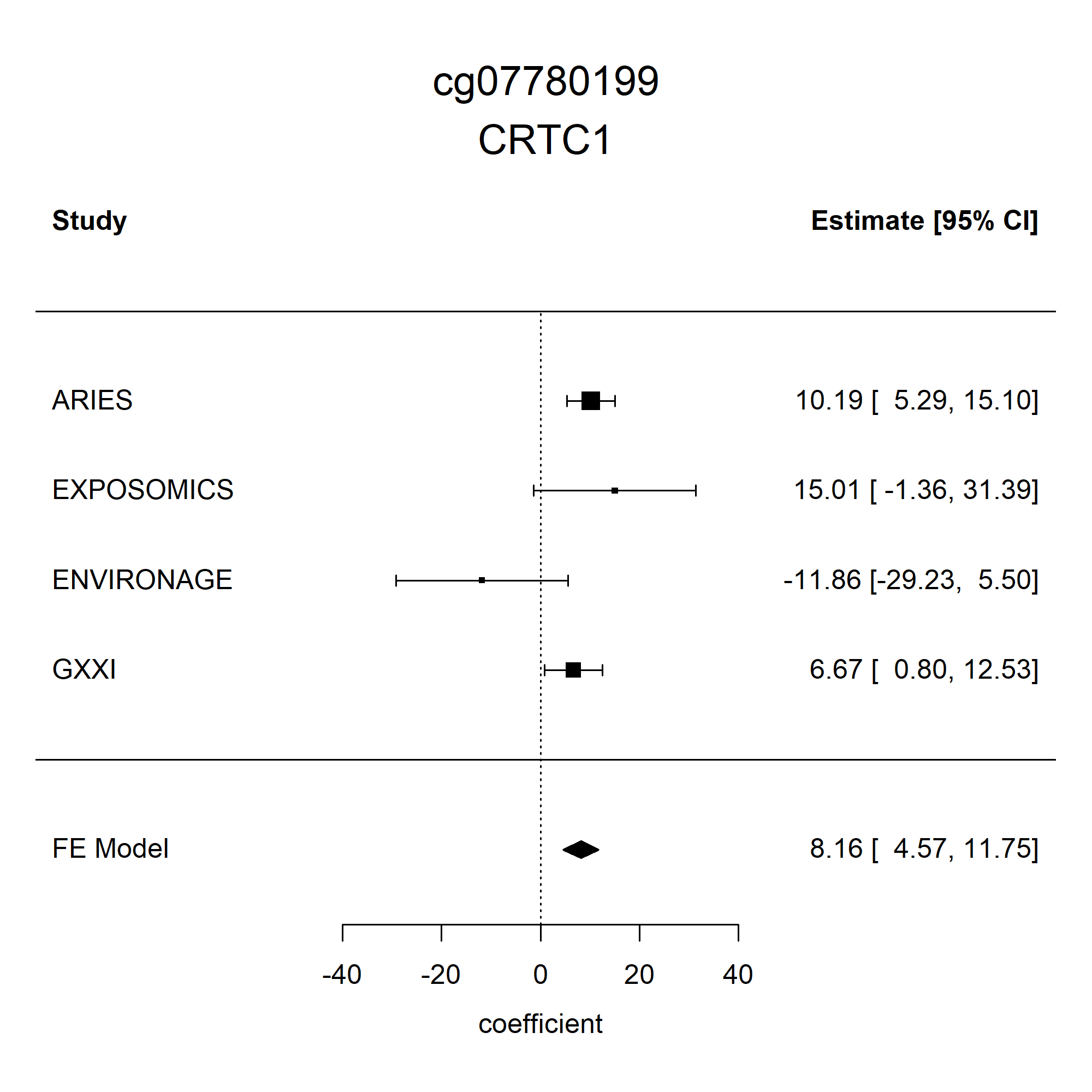

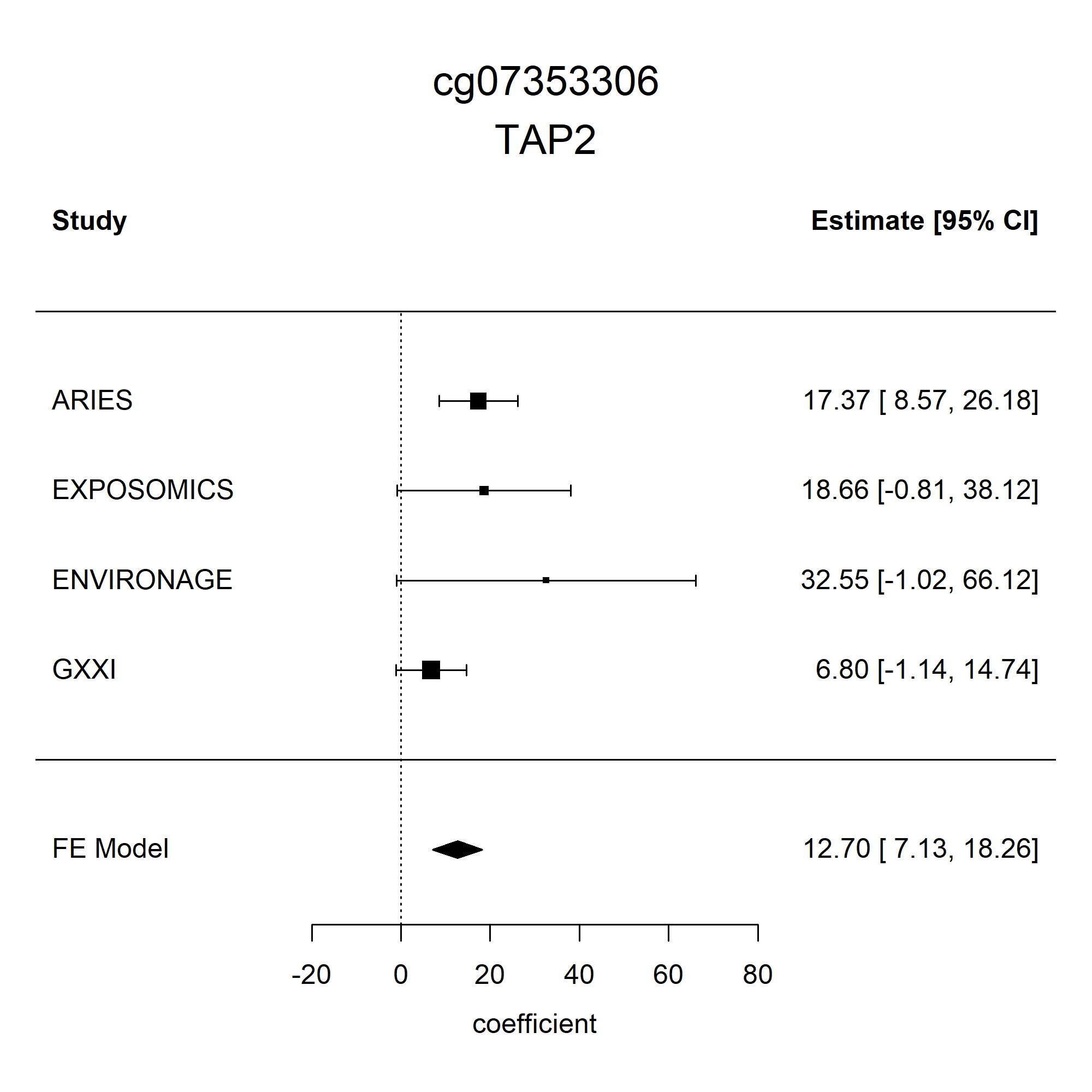

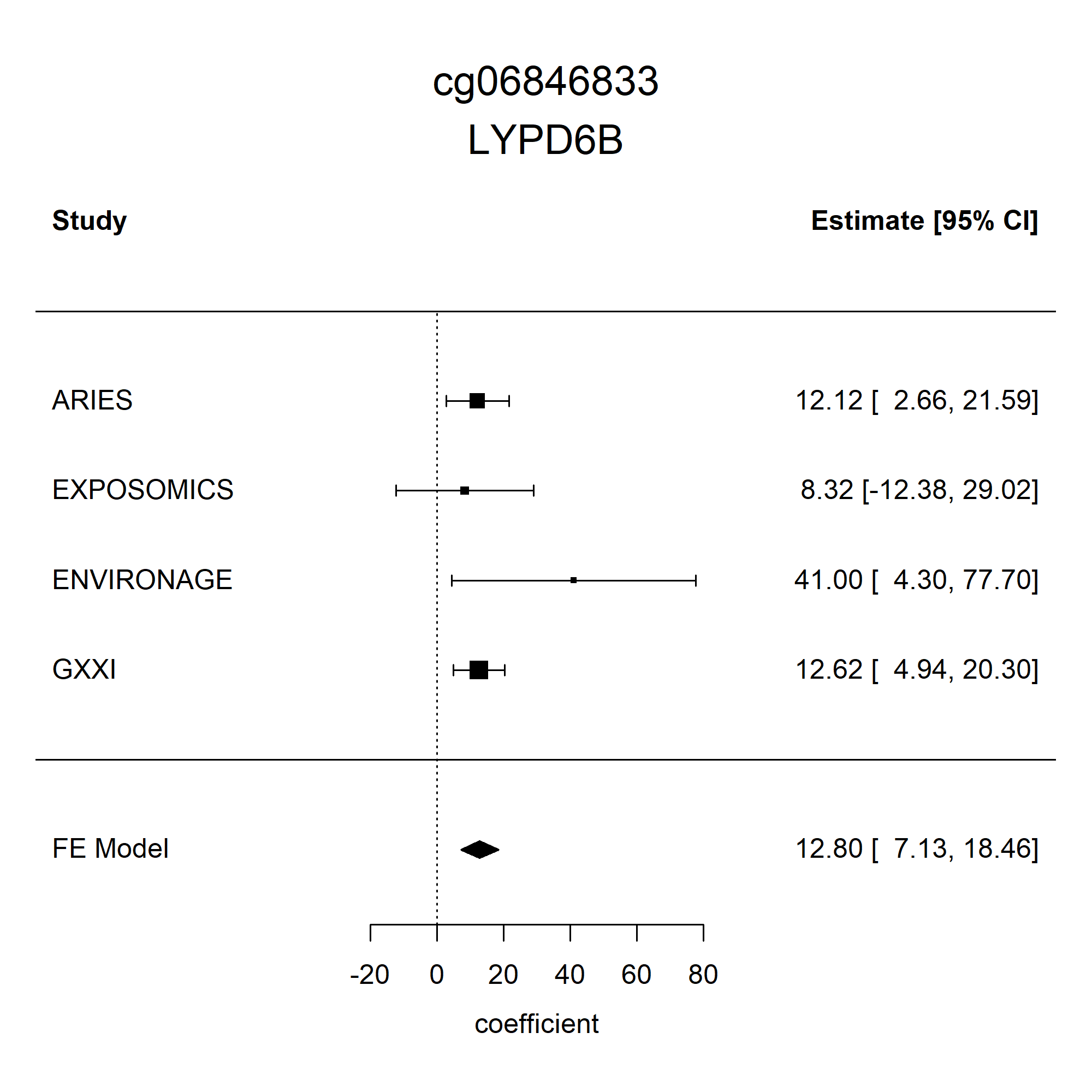

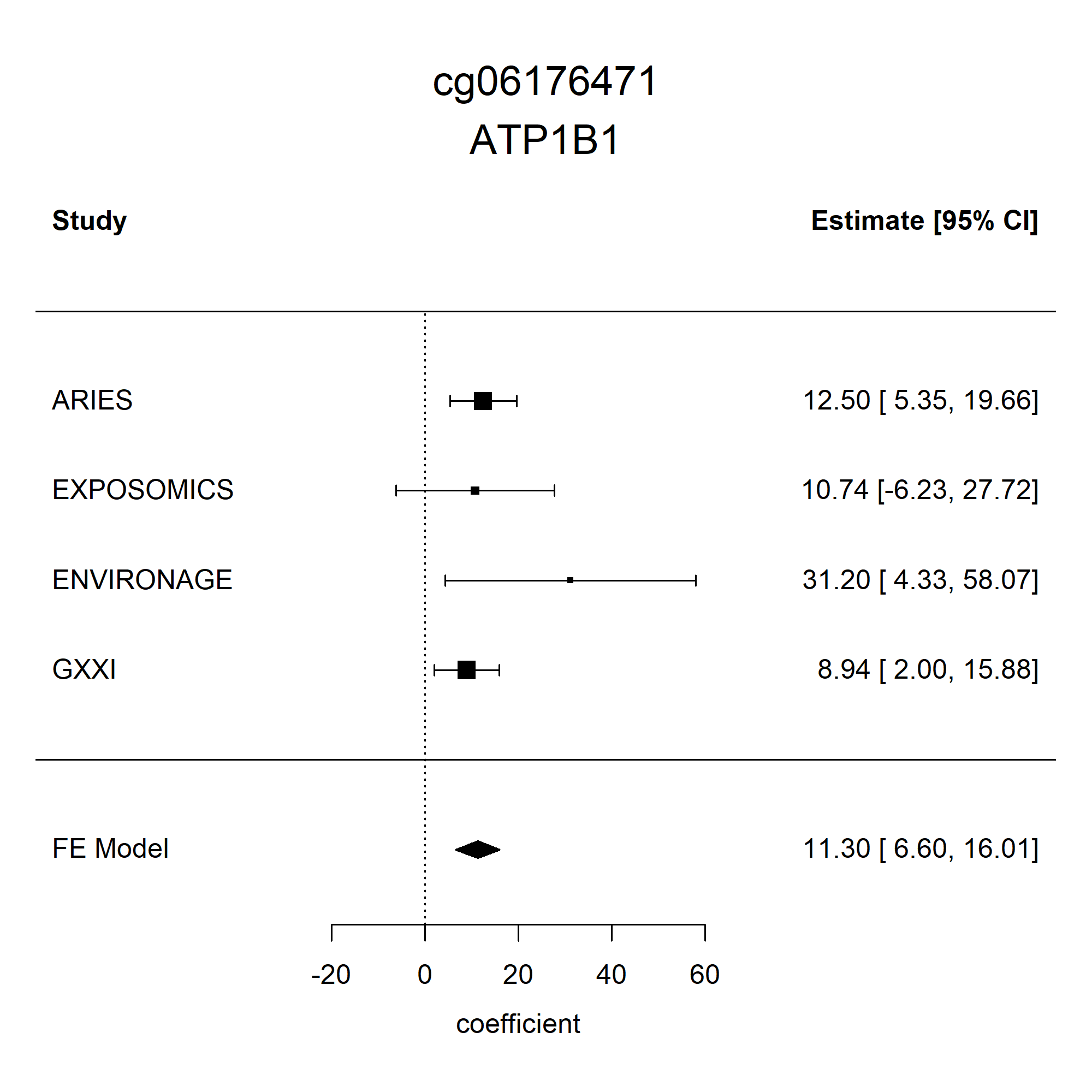

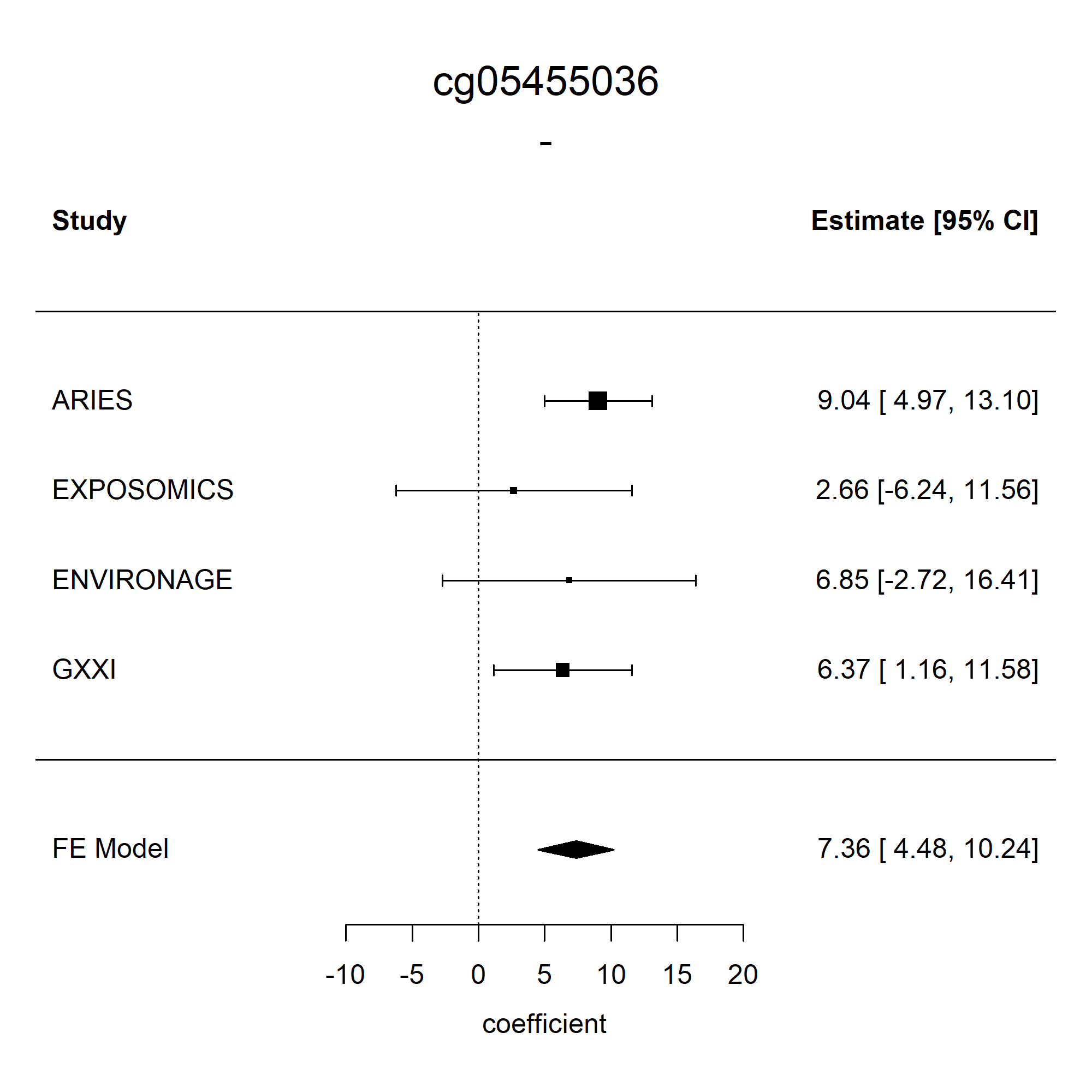

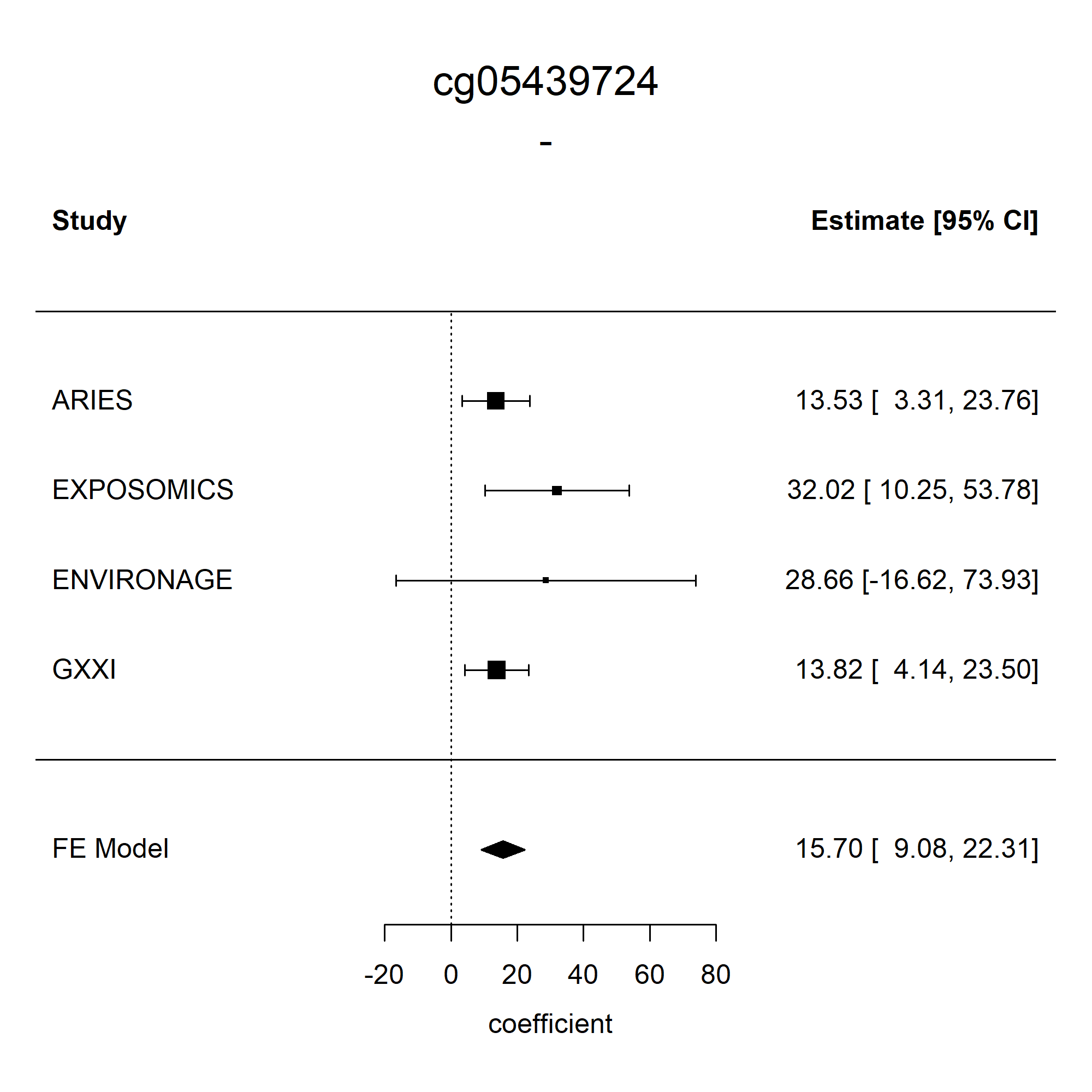

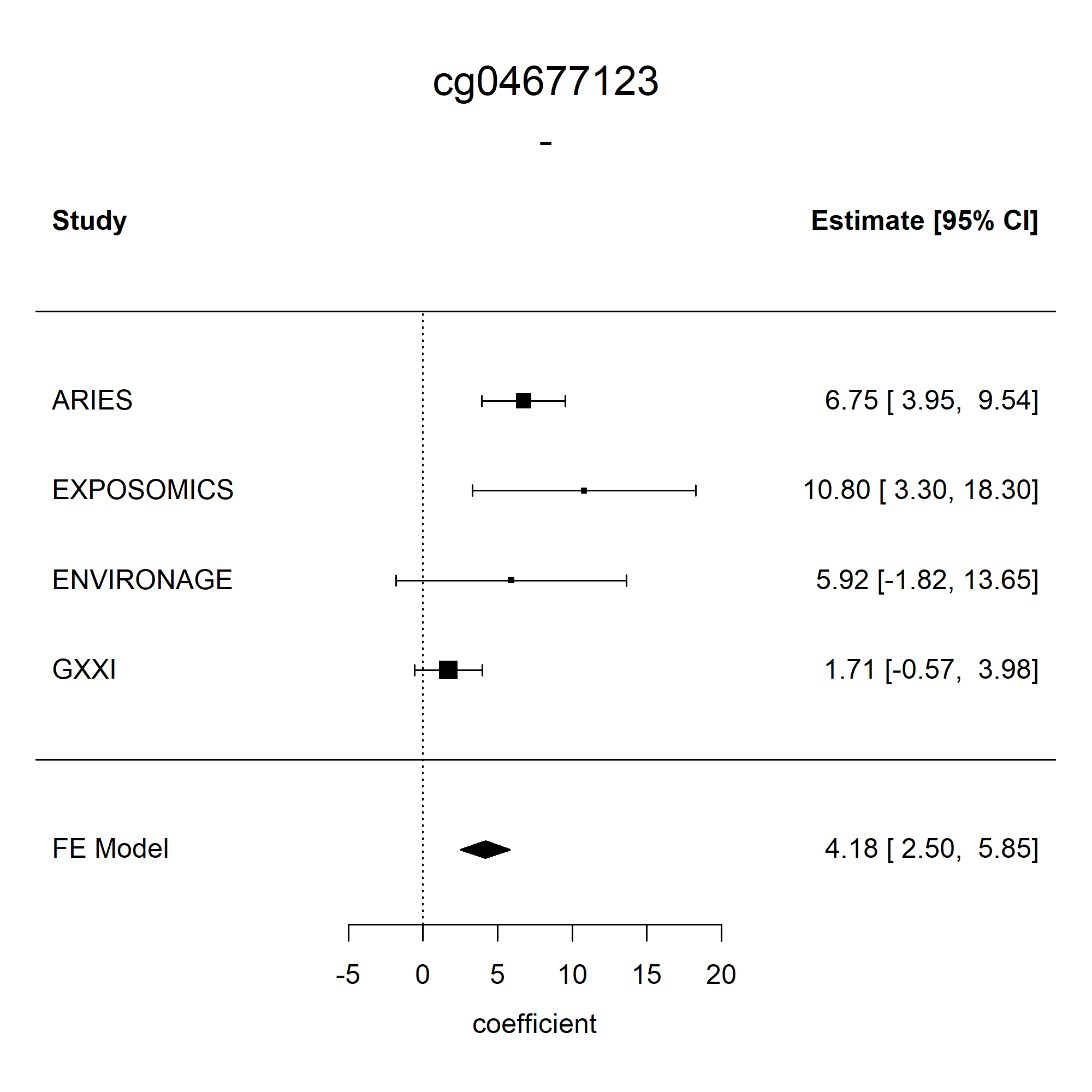

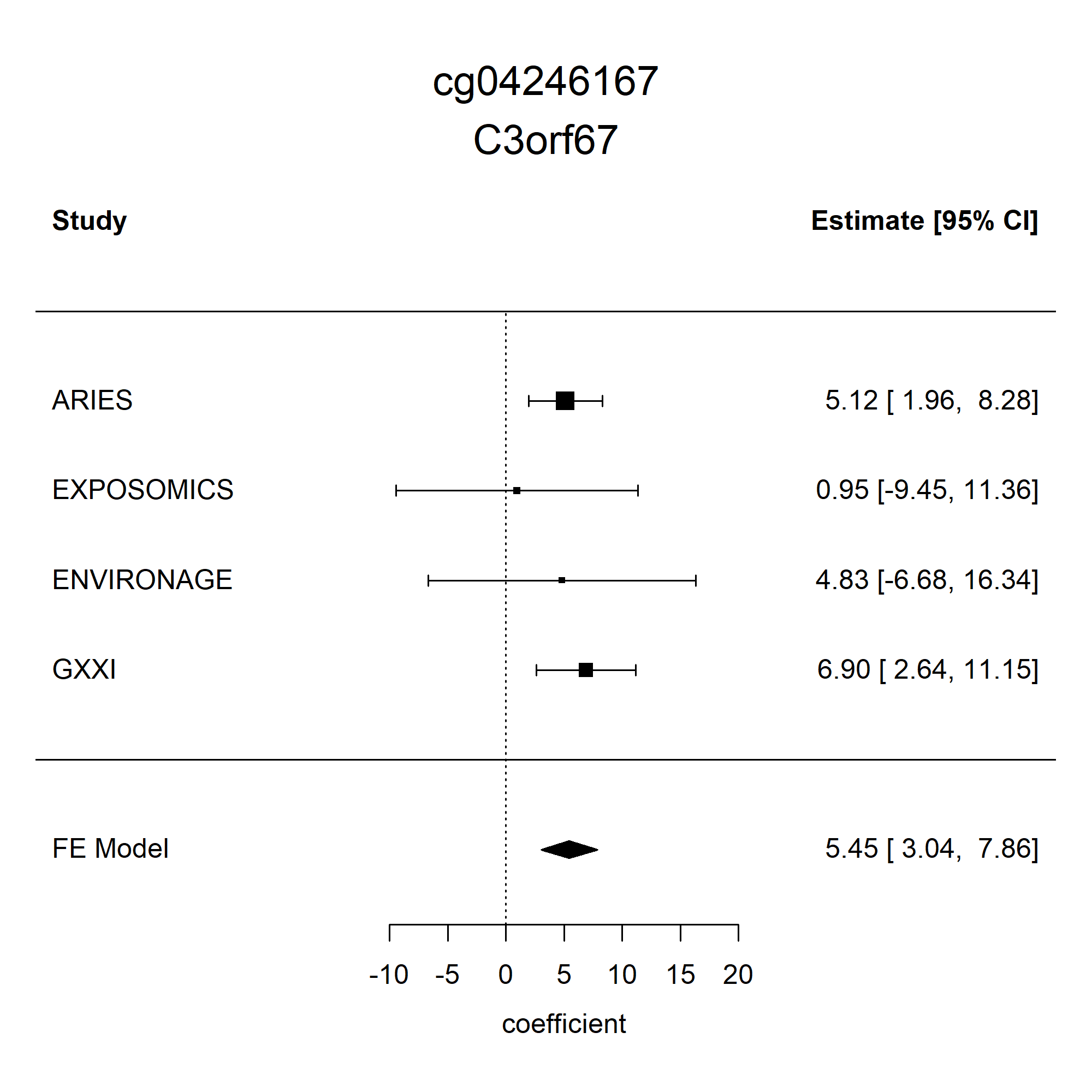

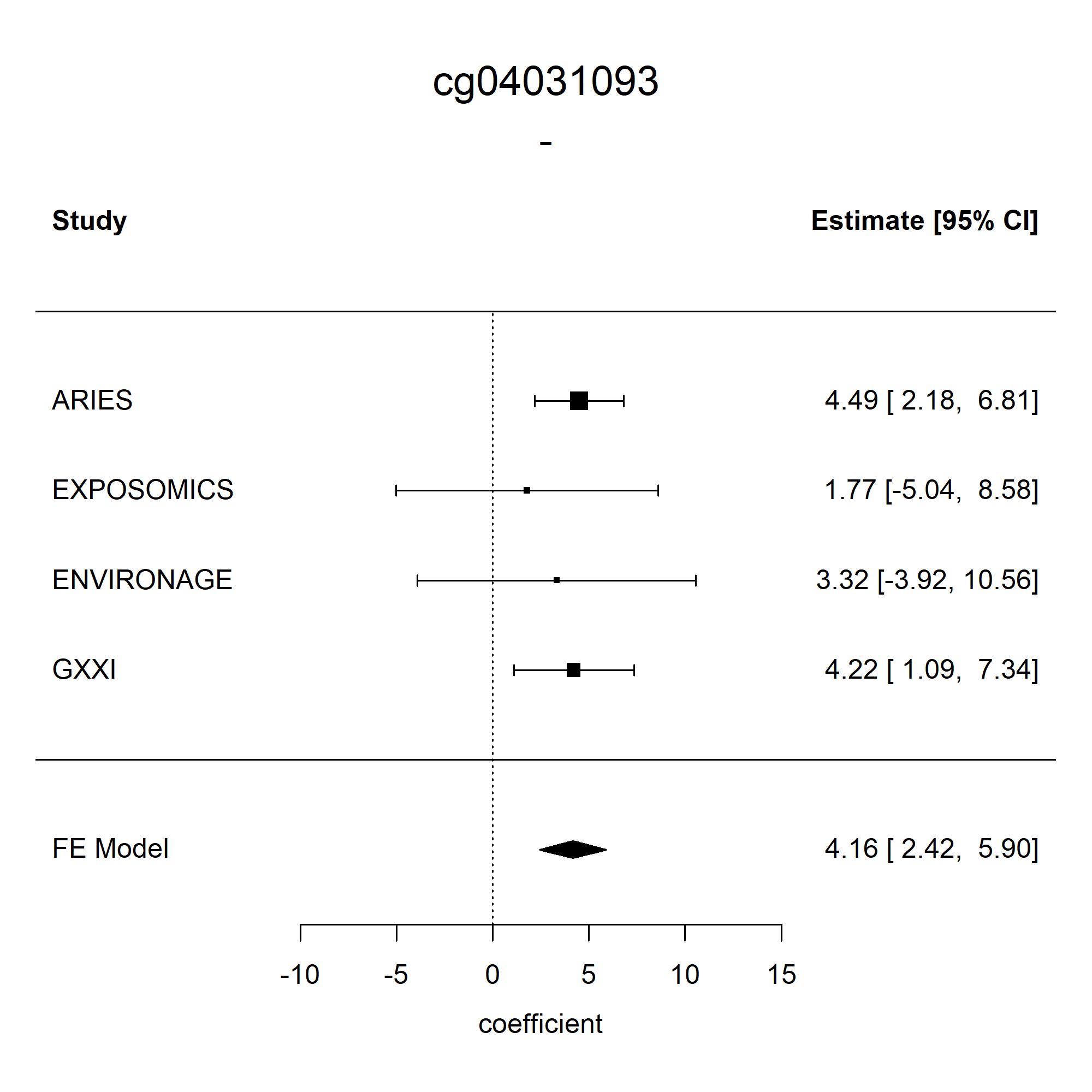

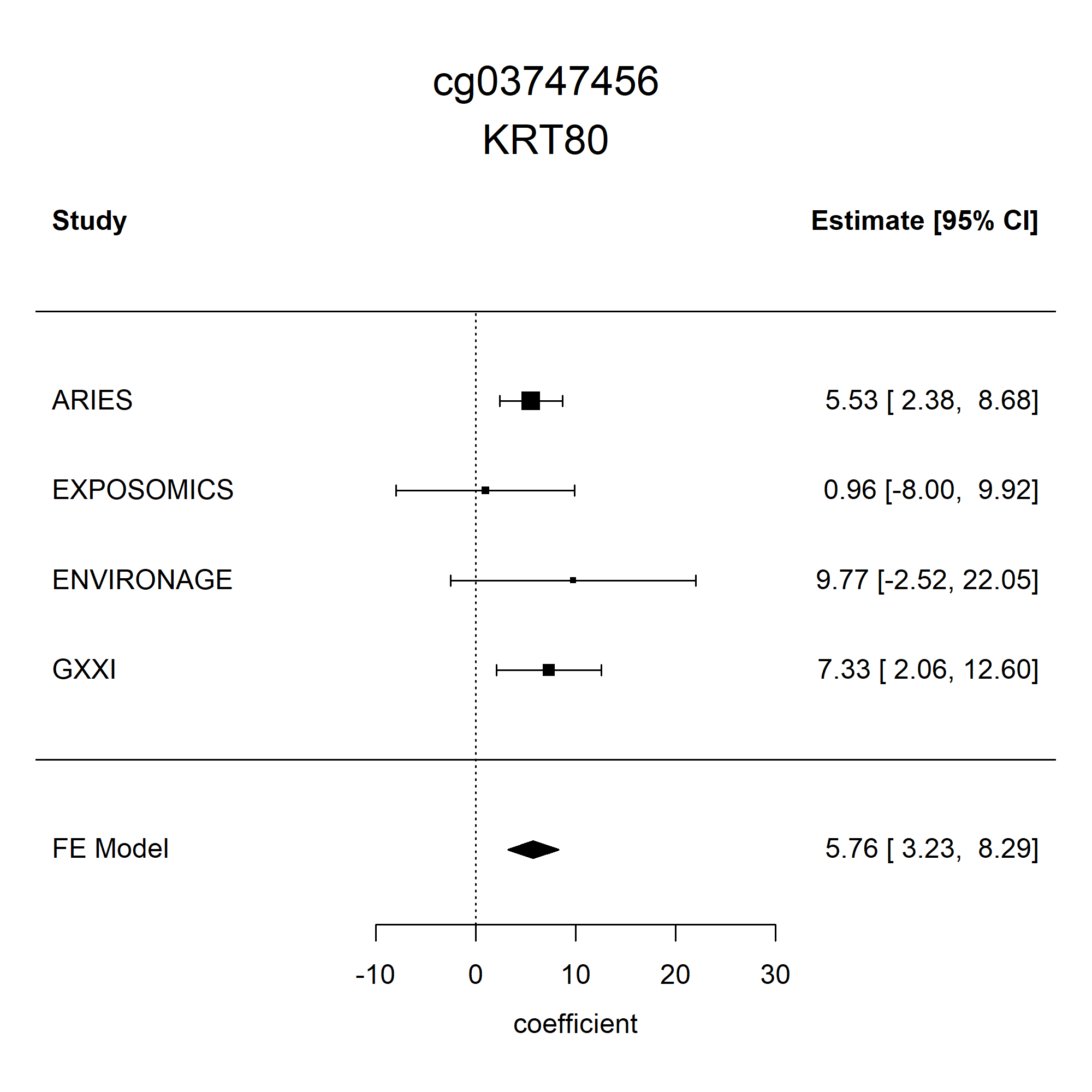

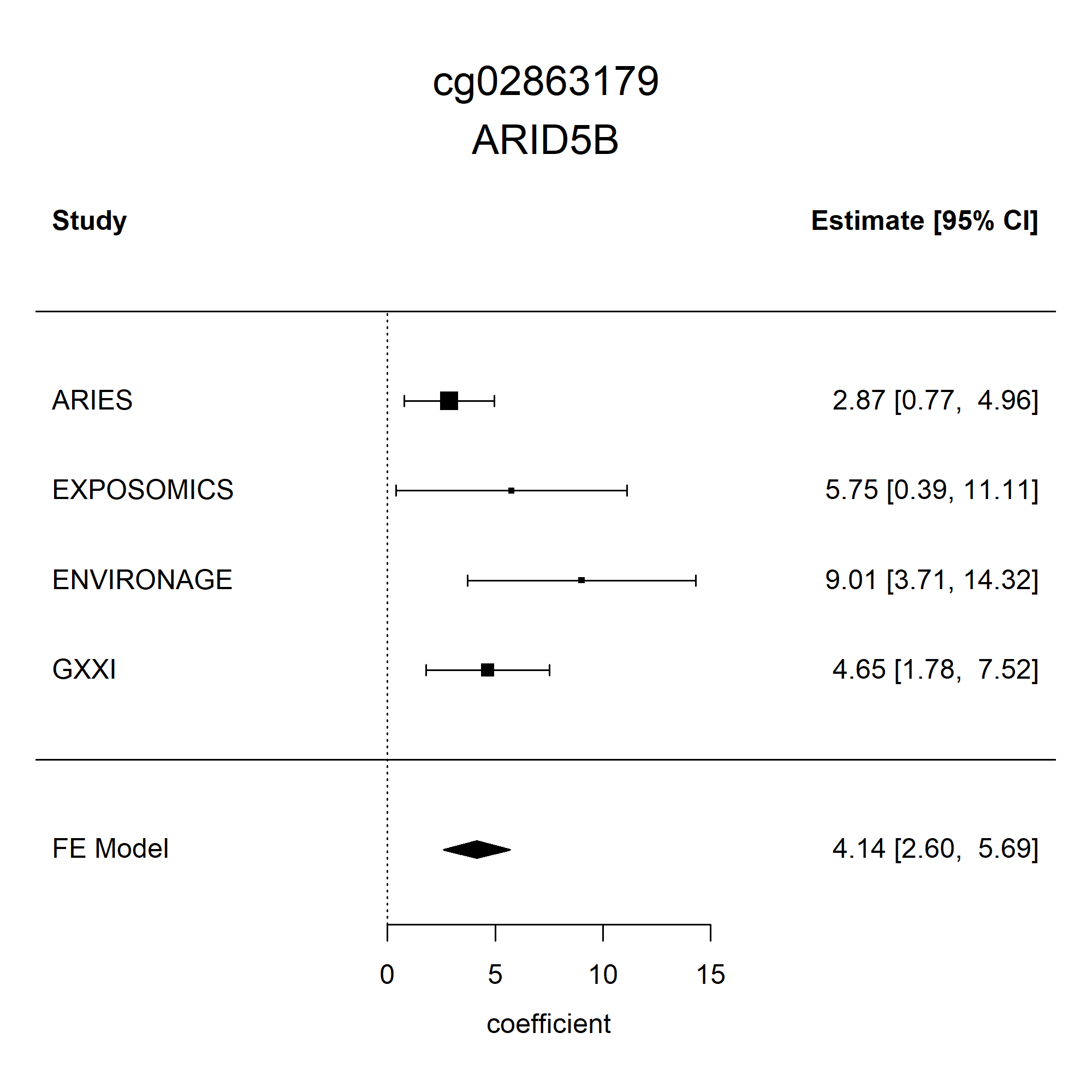

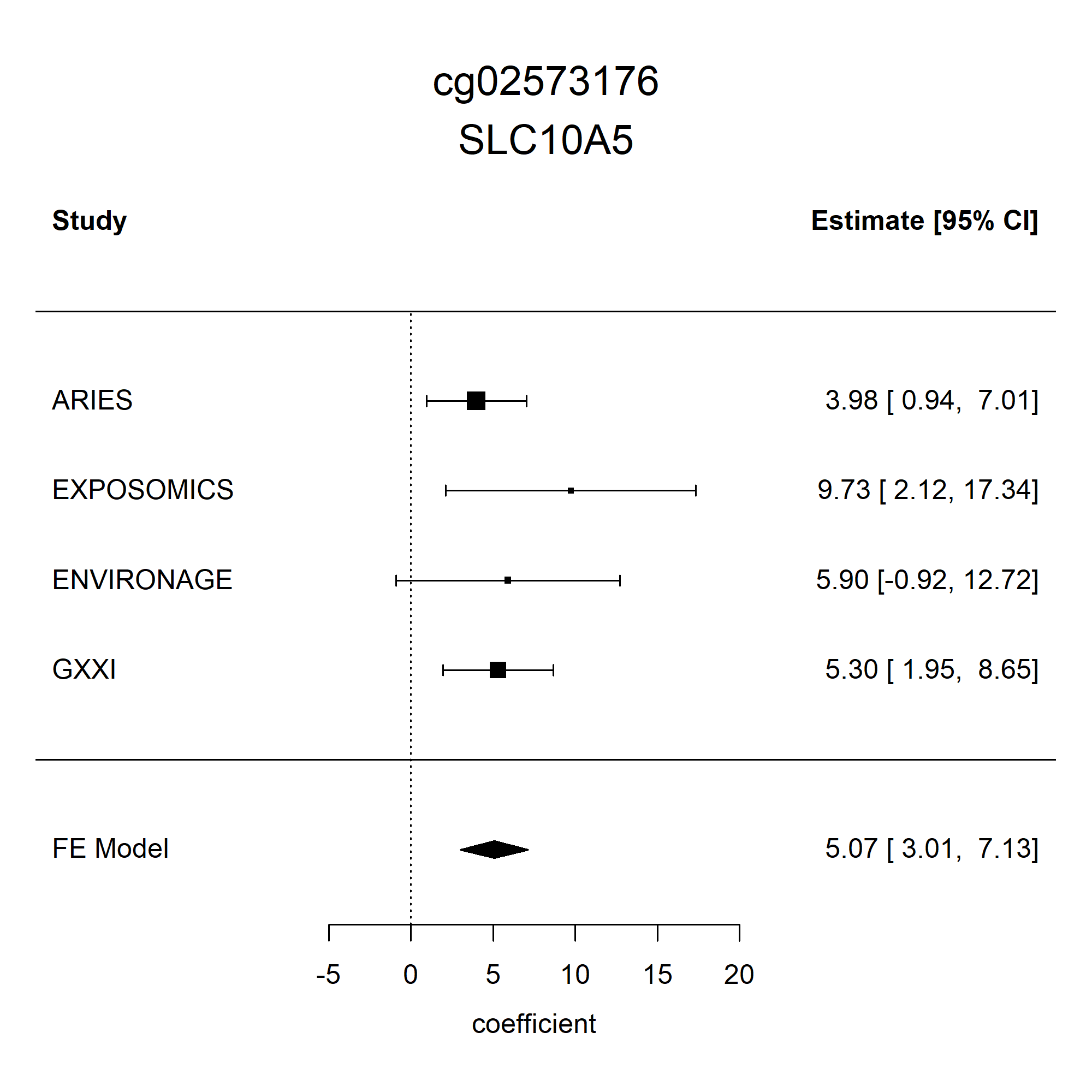

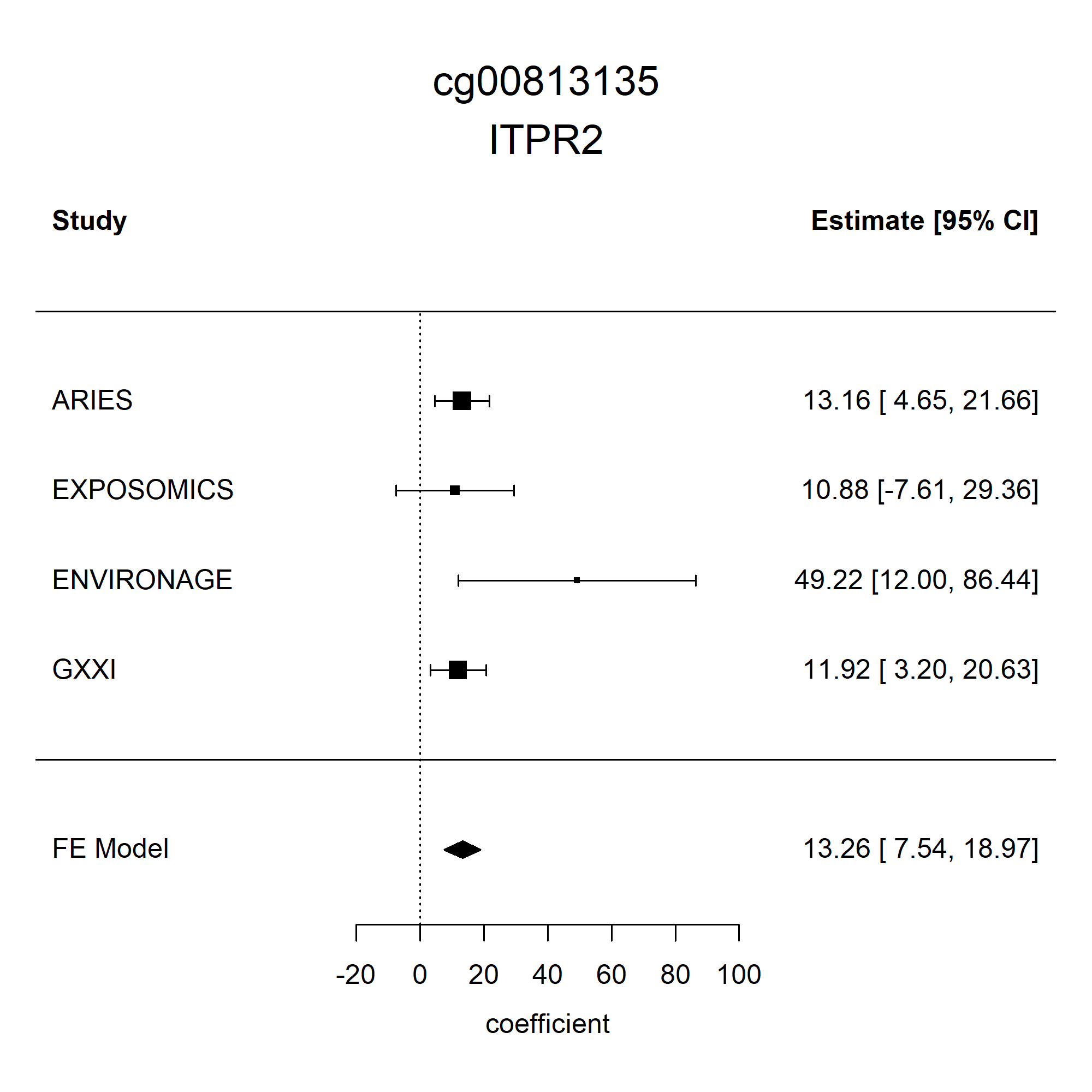

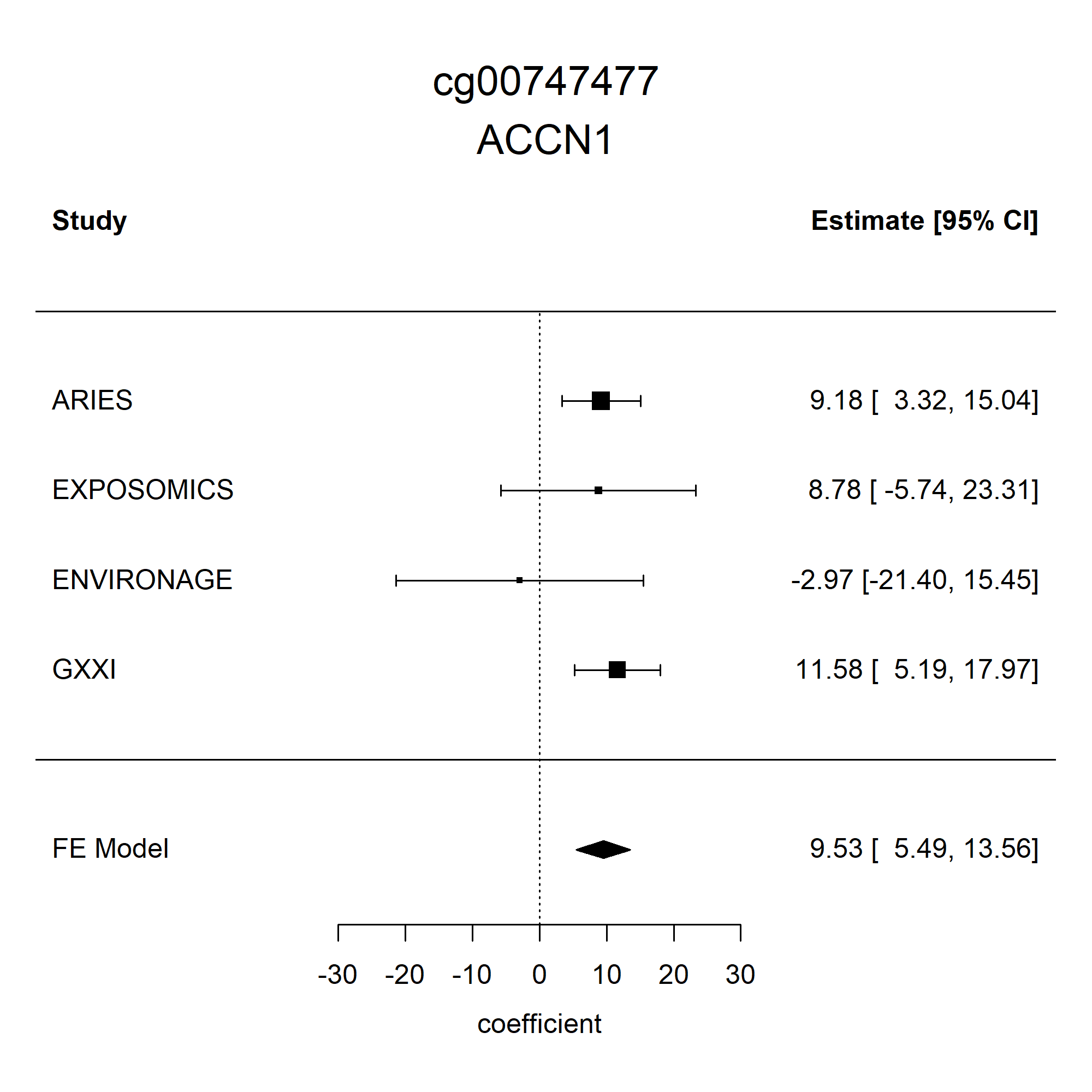

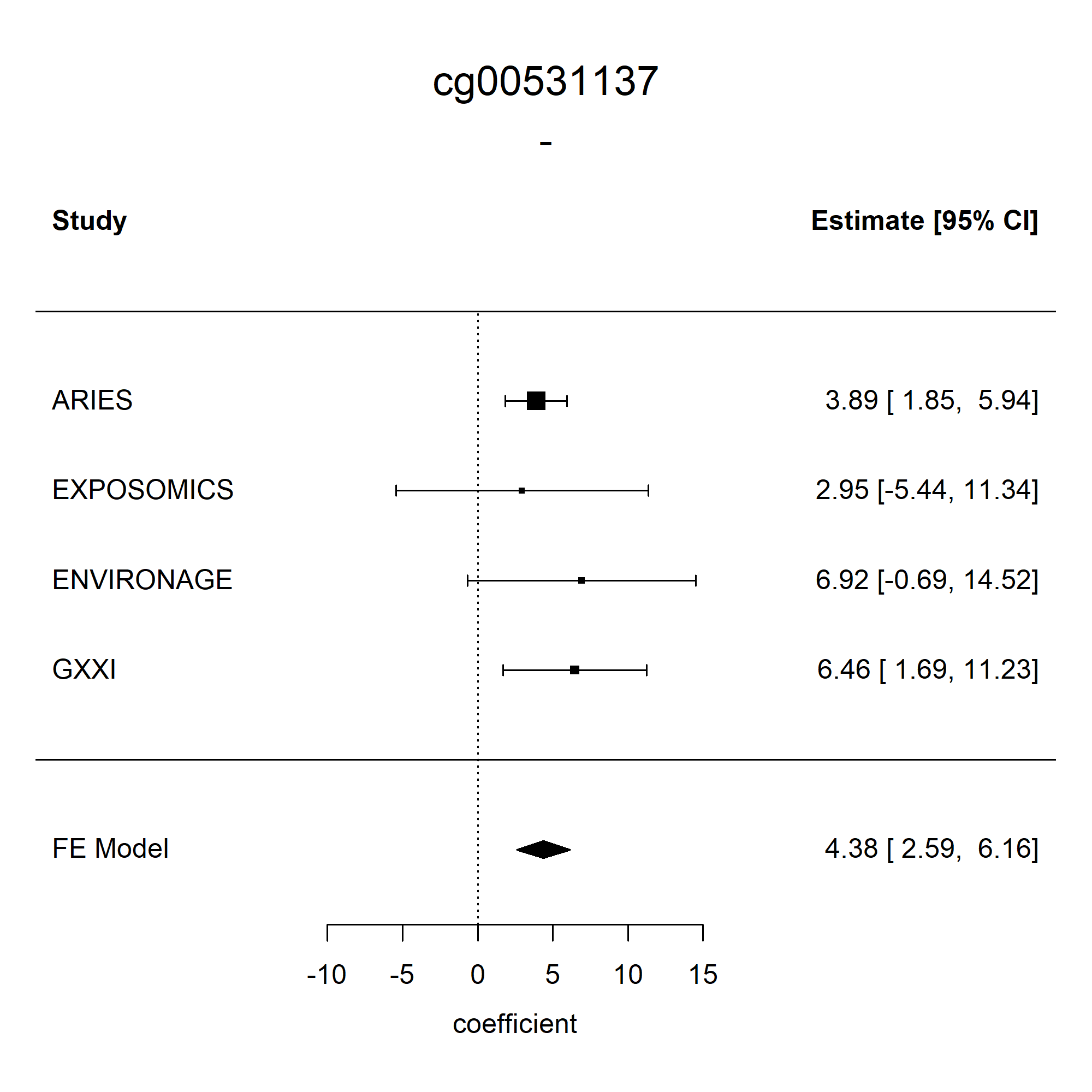

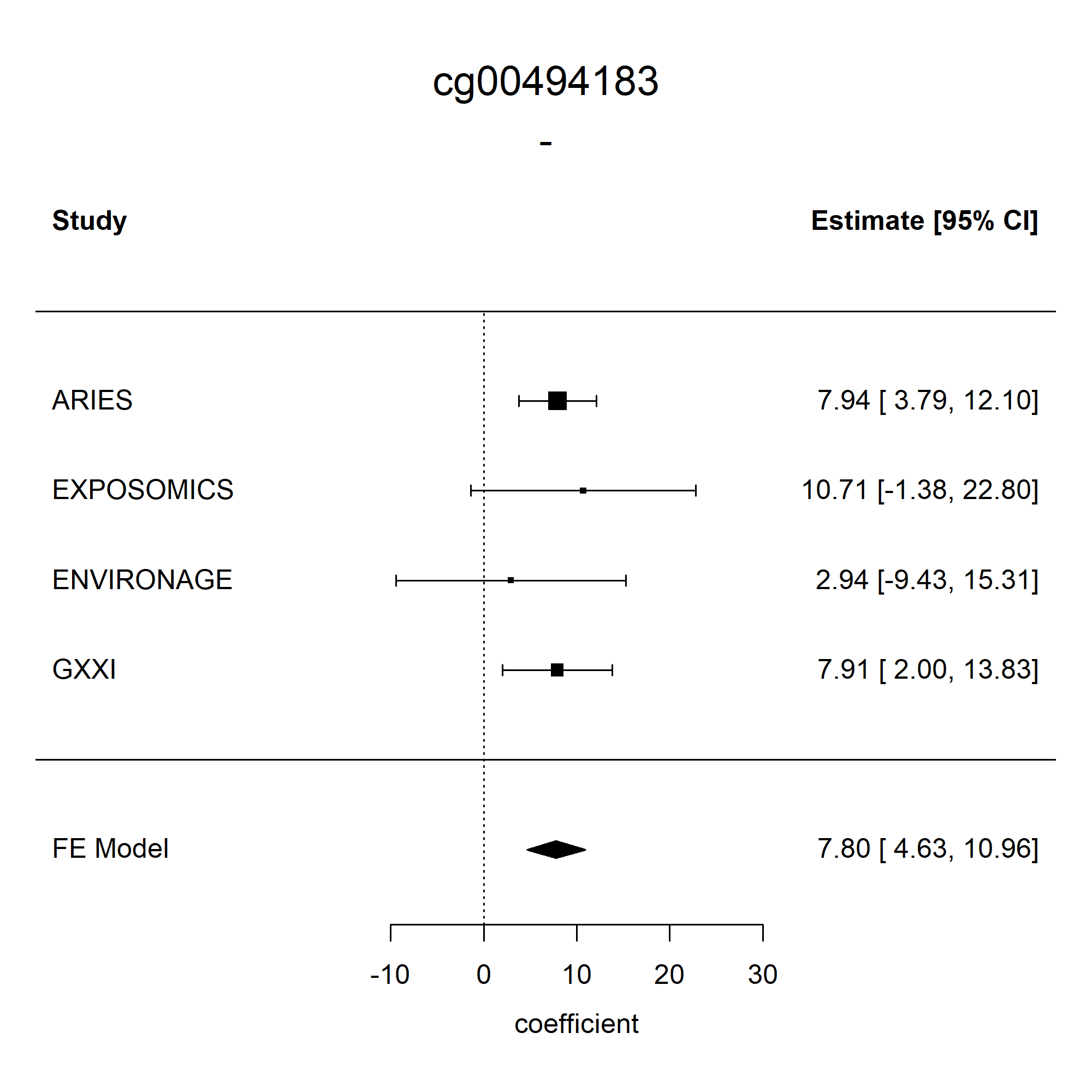

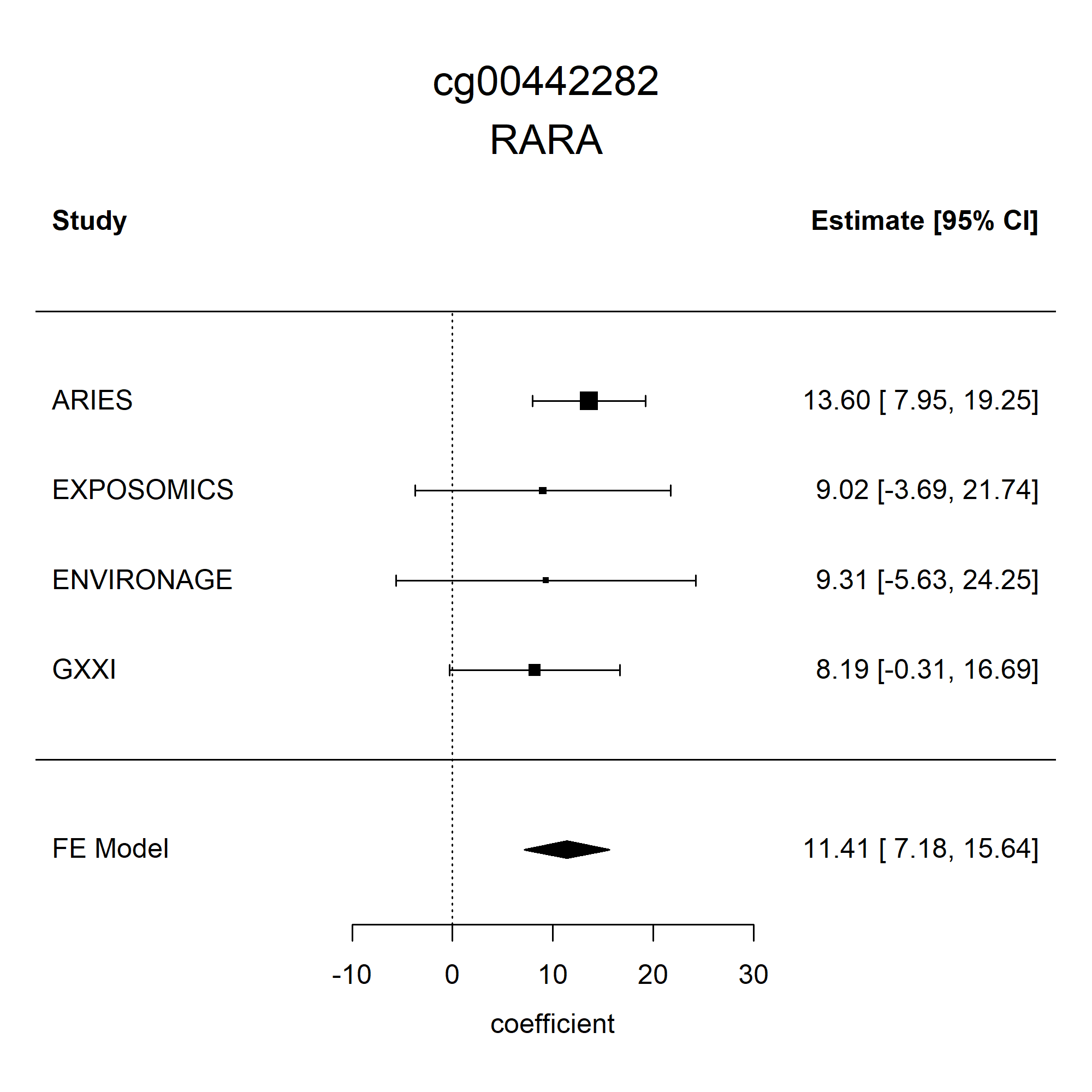

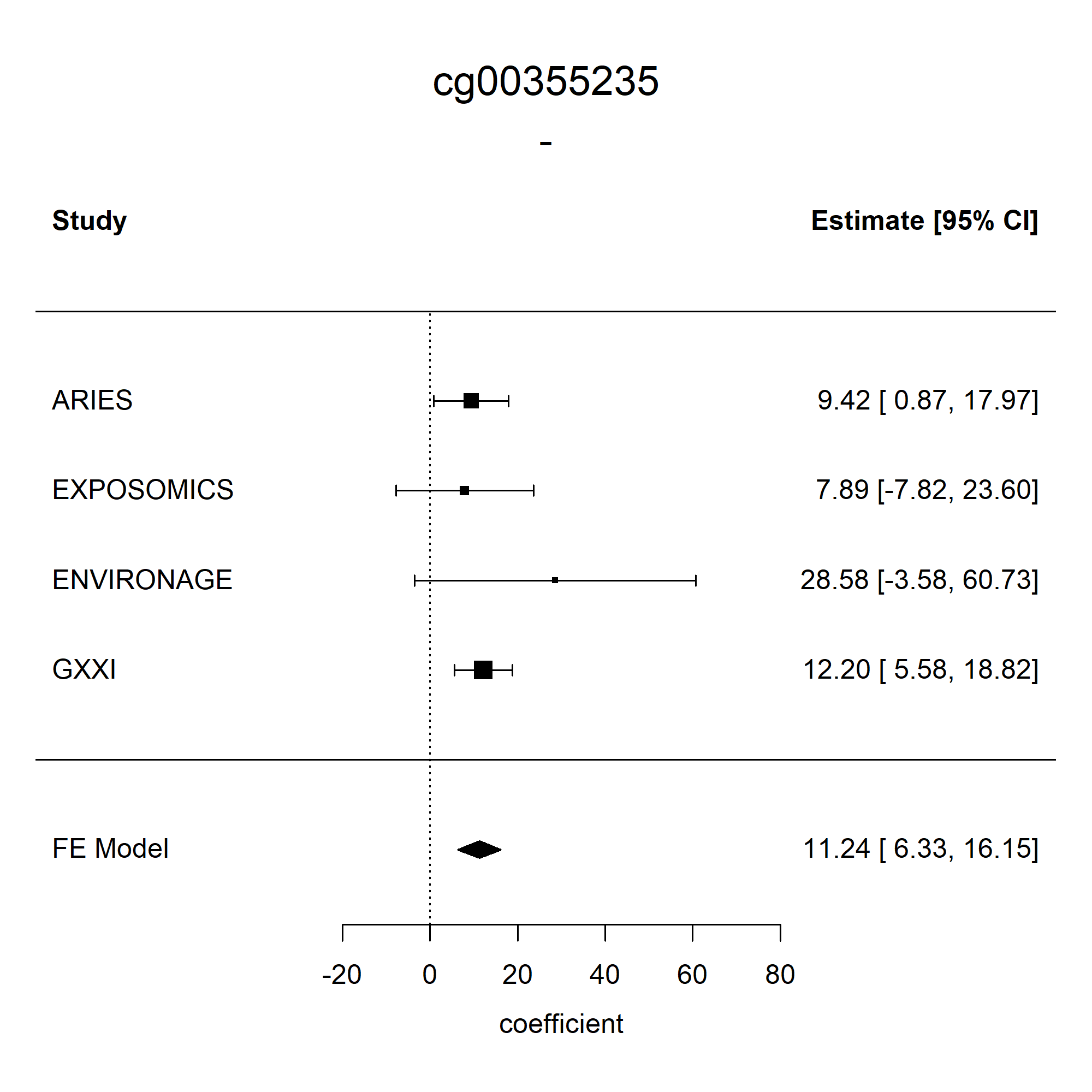

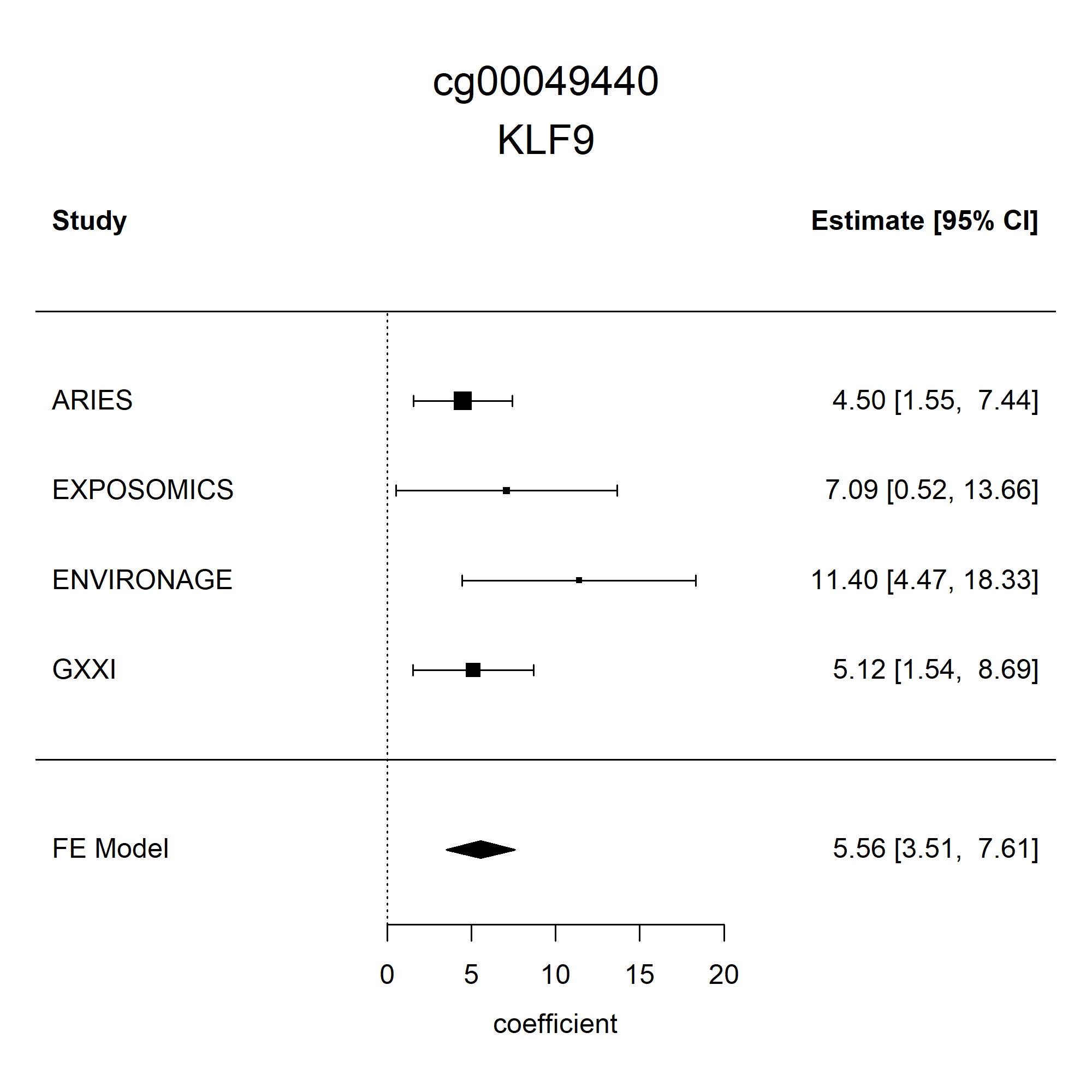


95% CI=95% confidence interval


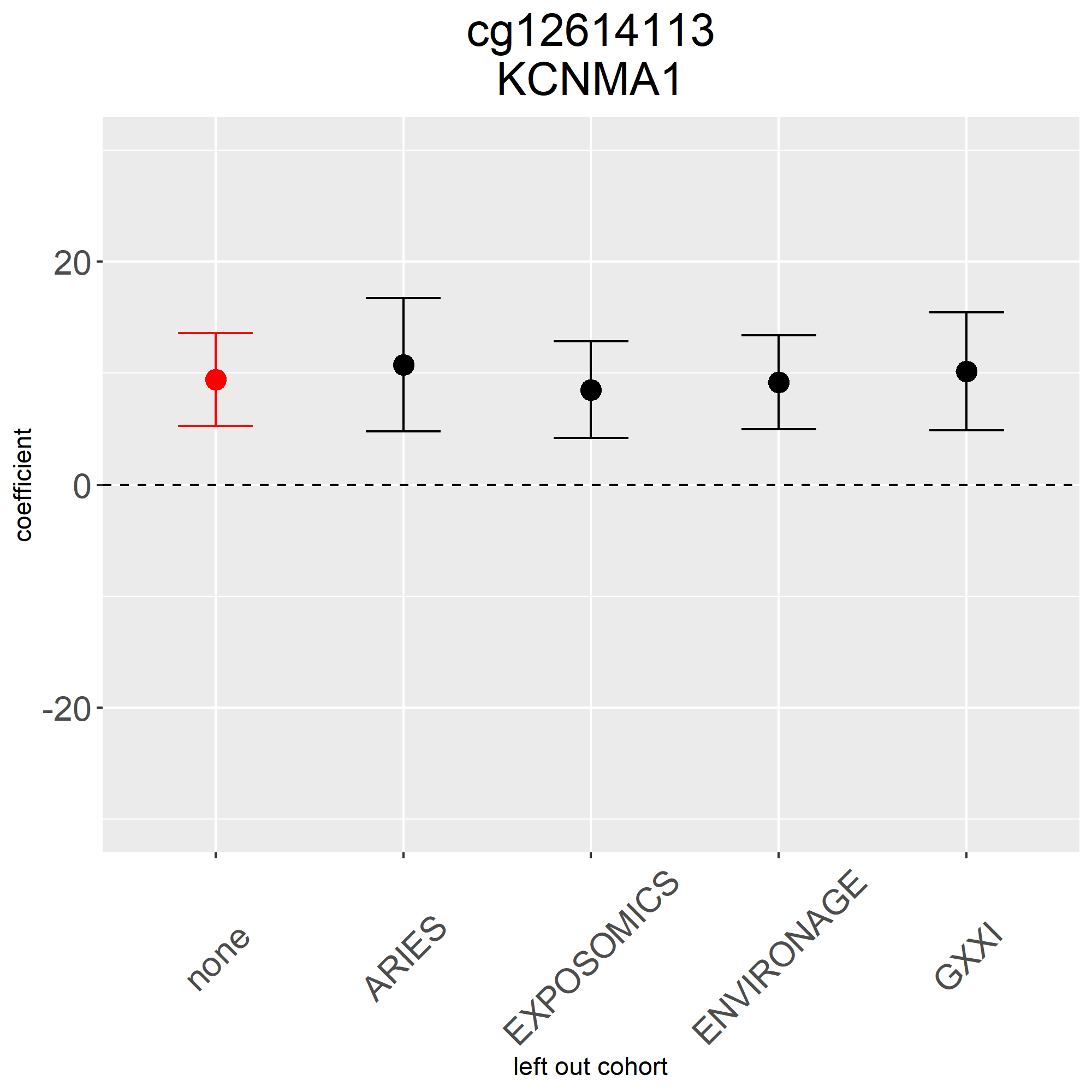

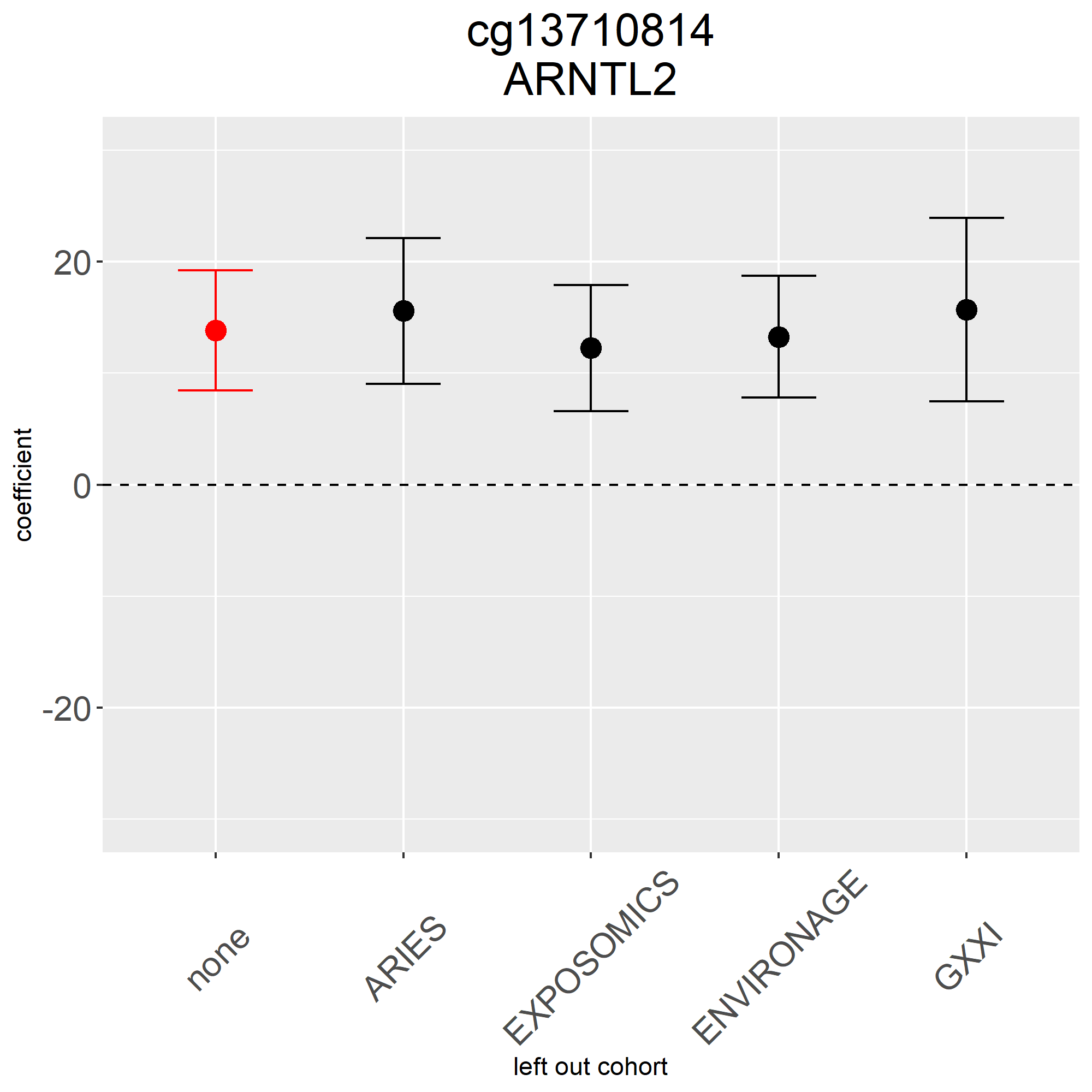

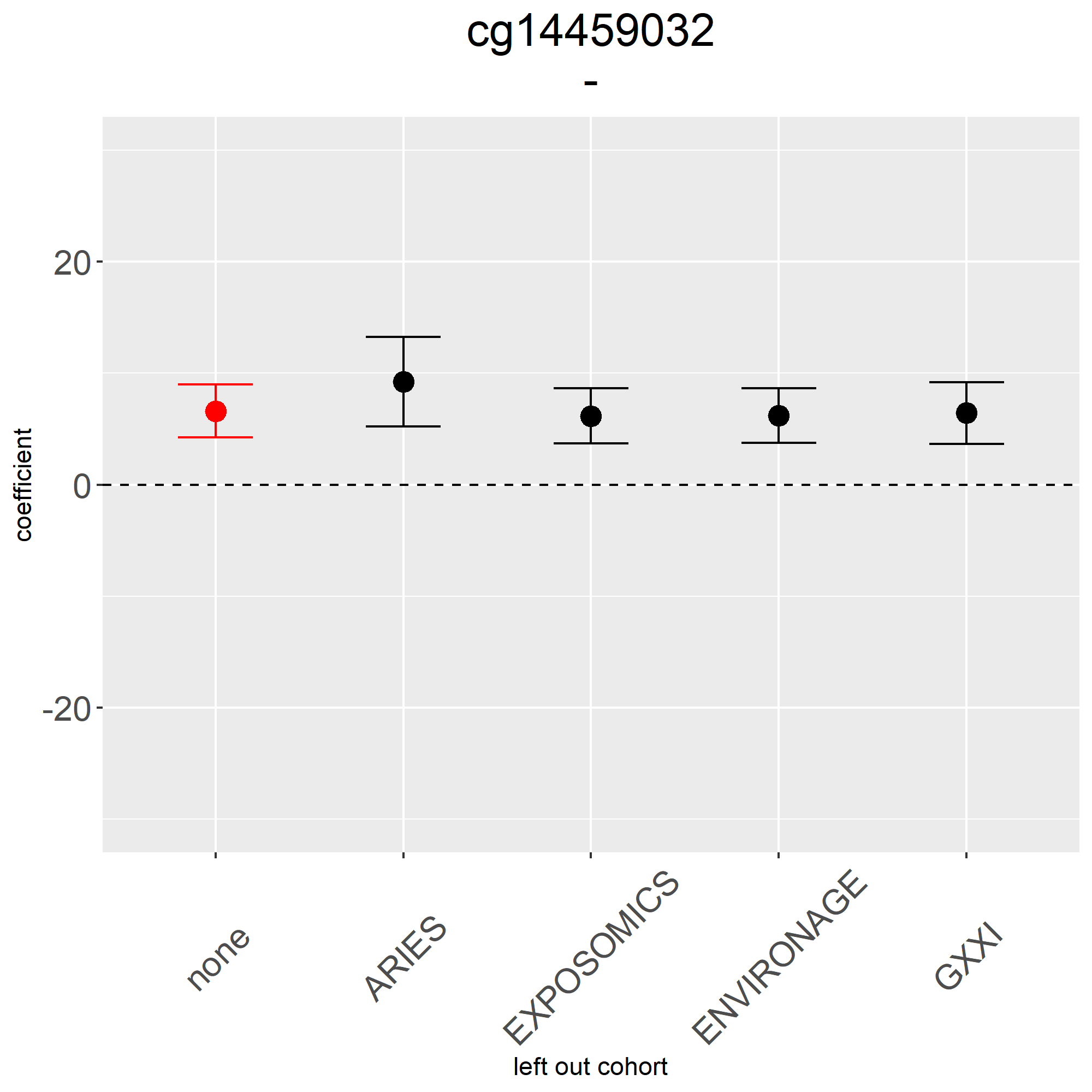

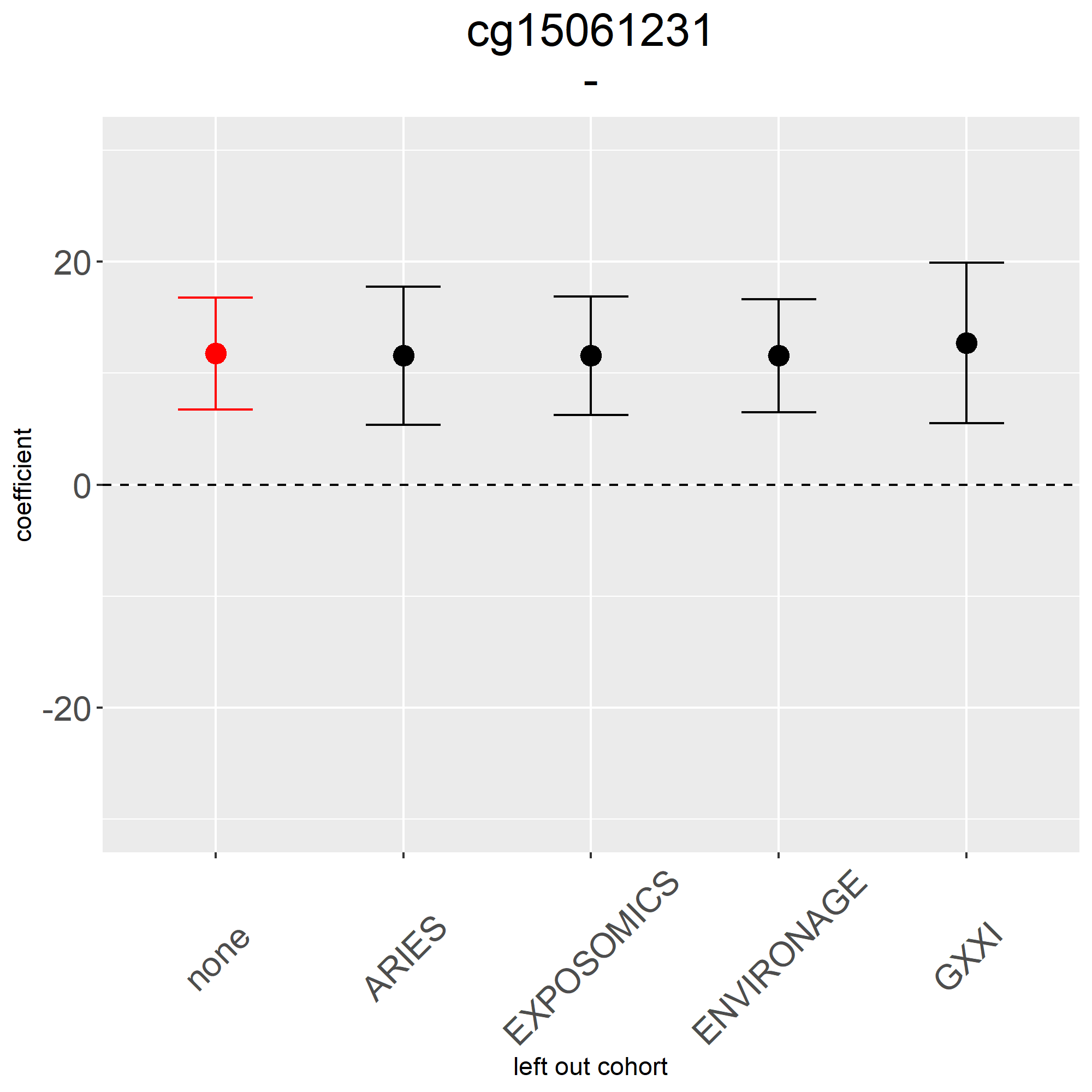

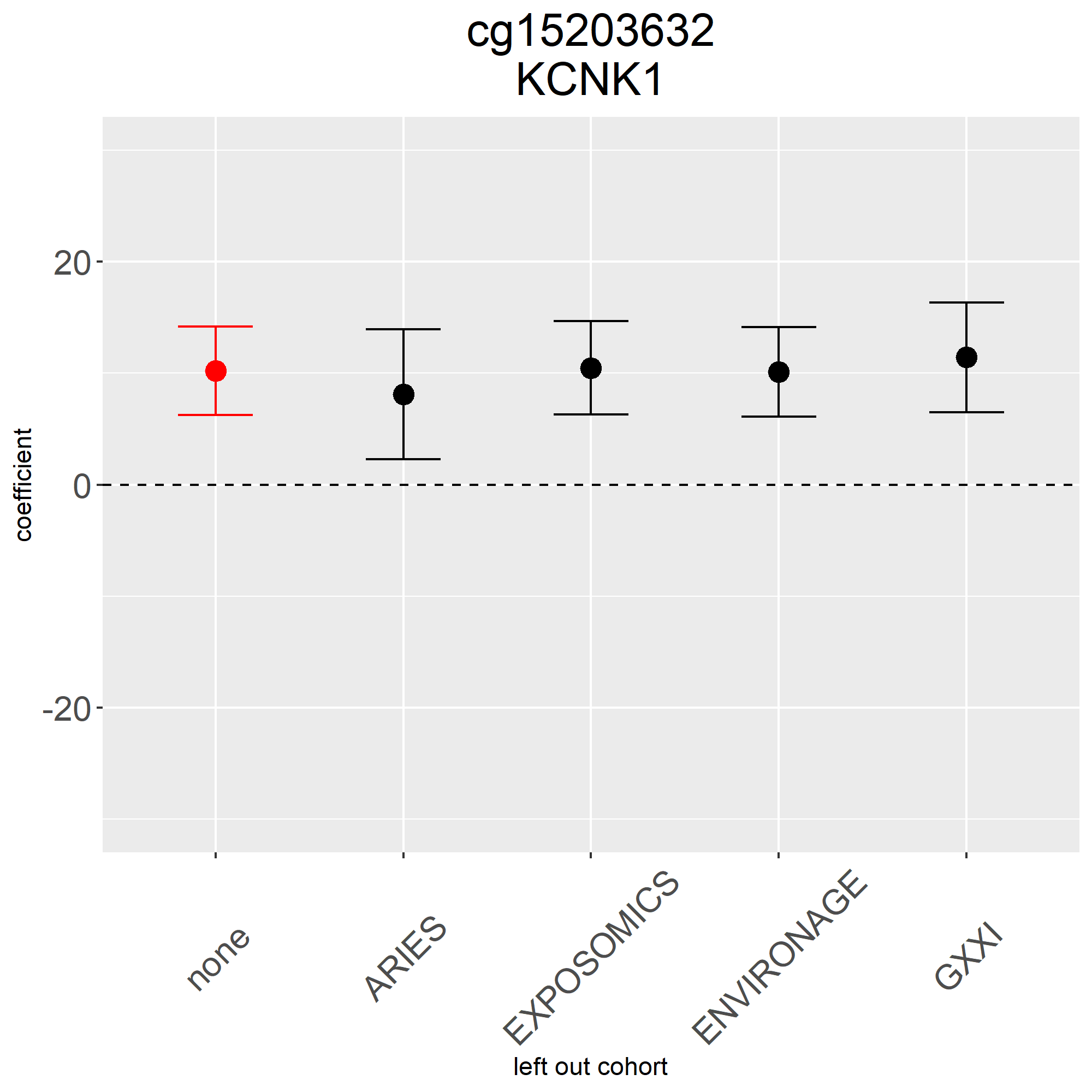

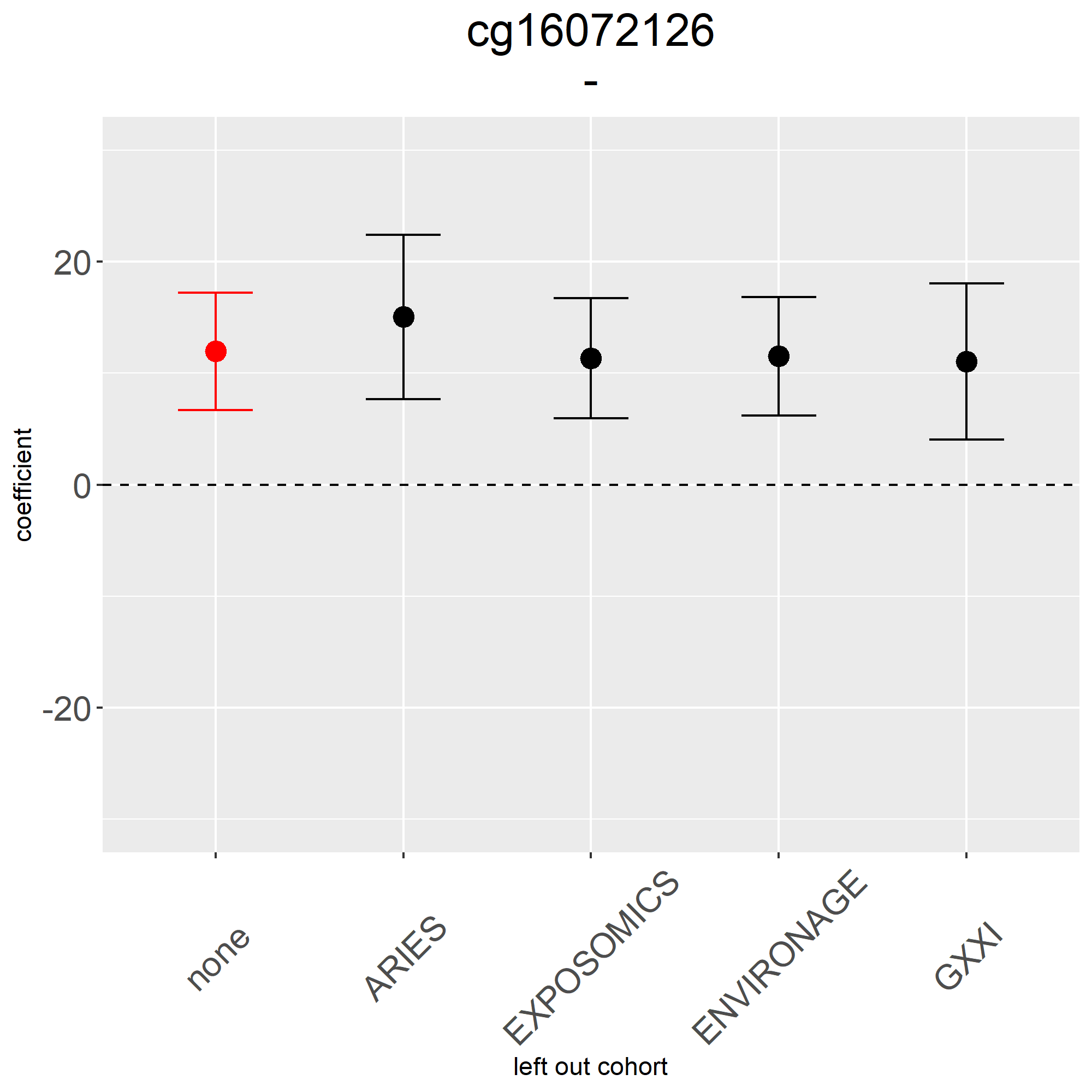

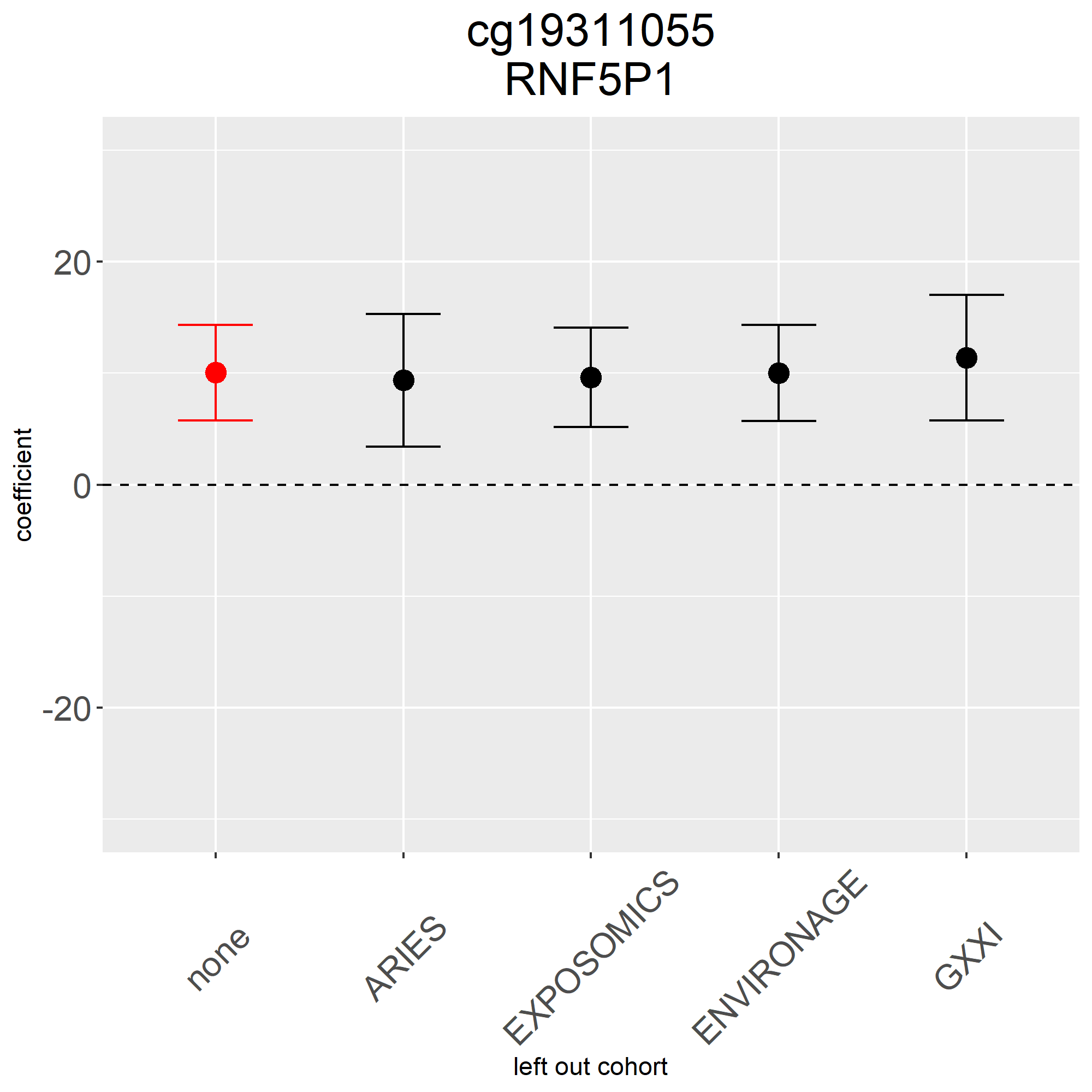

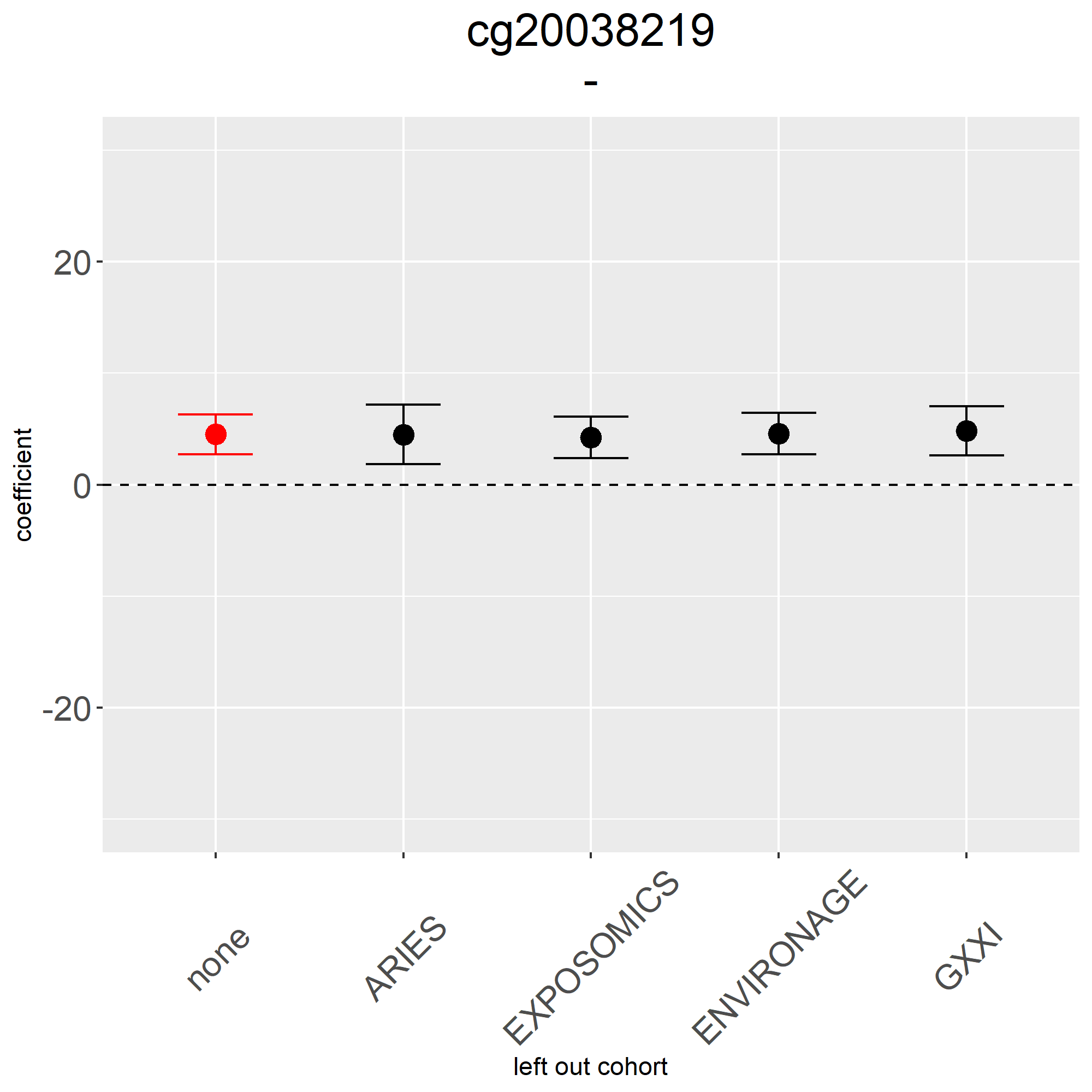

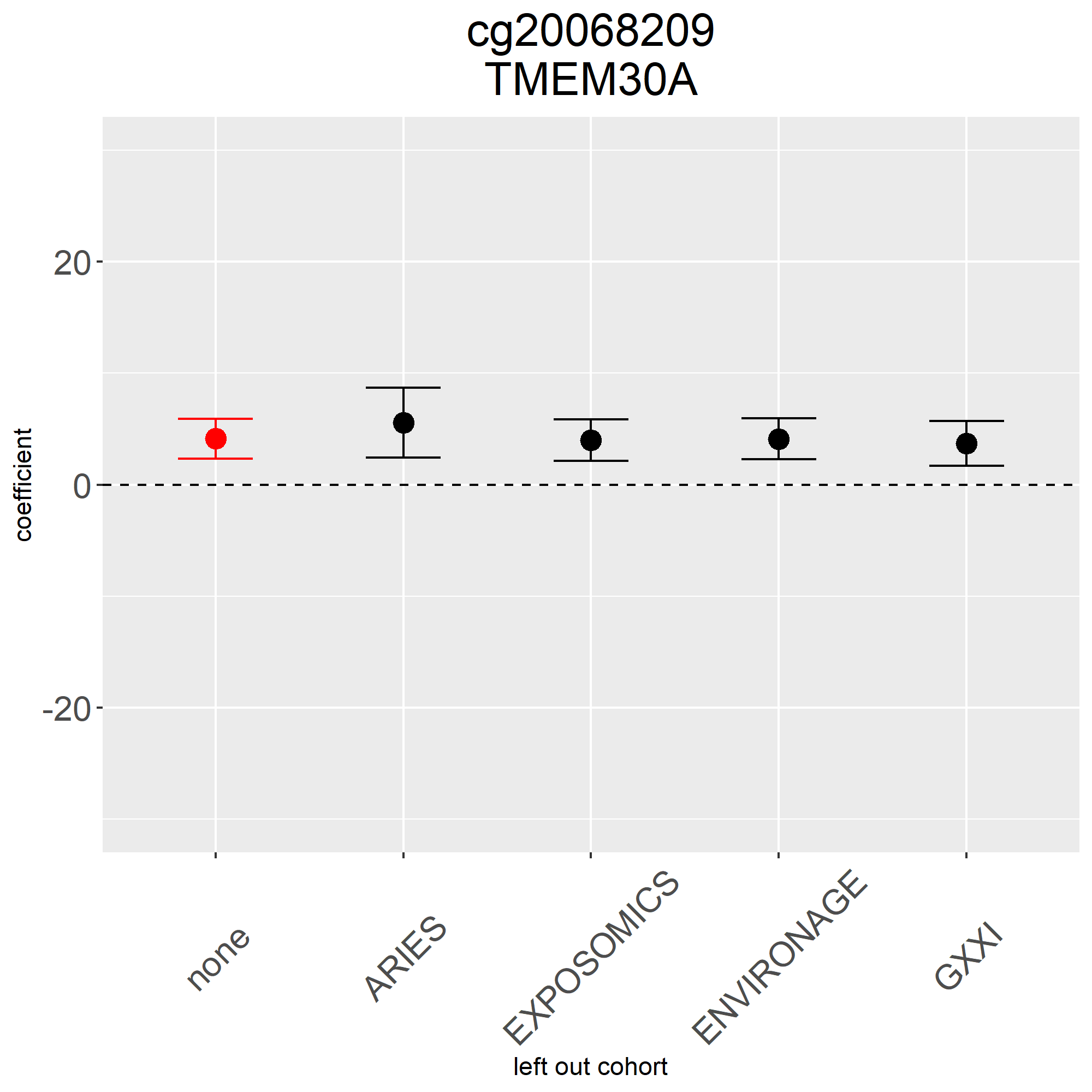

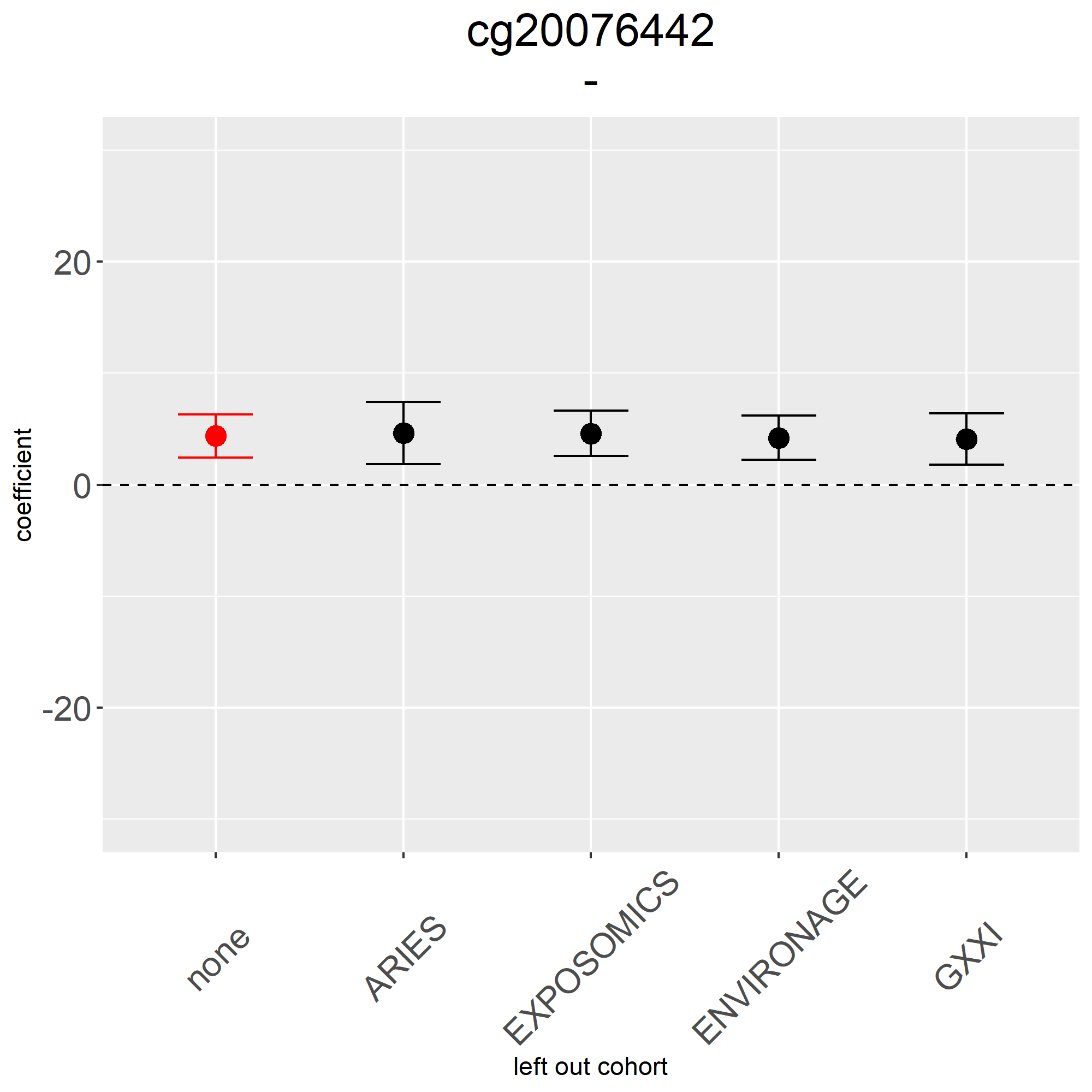

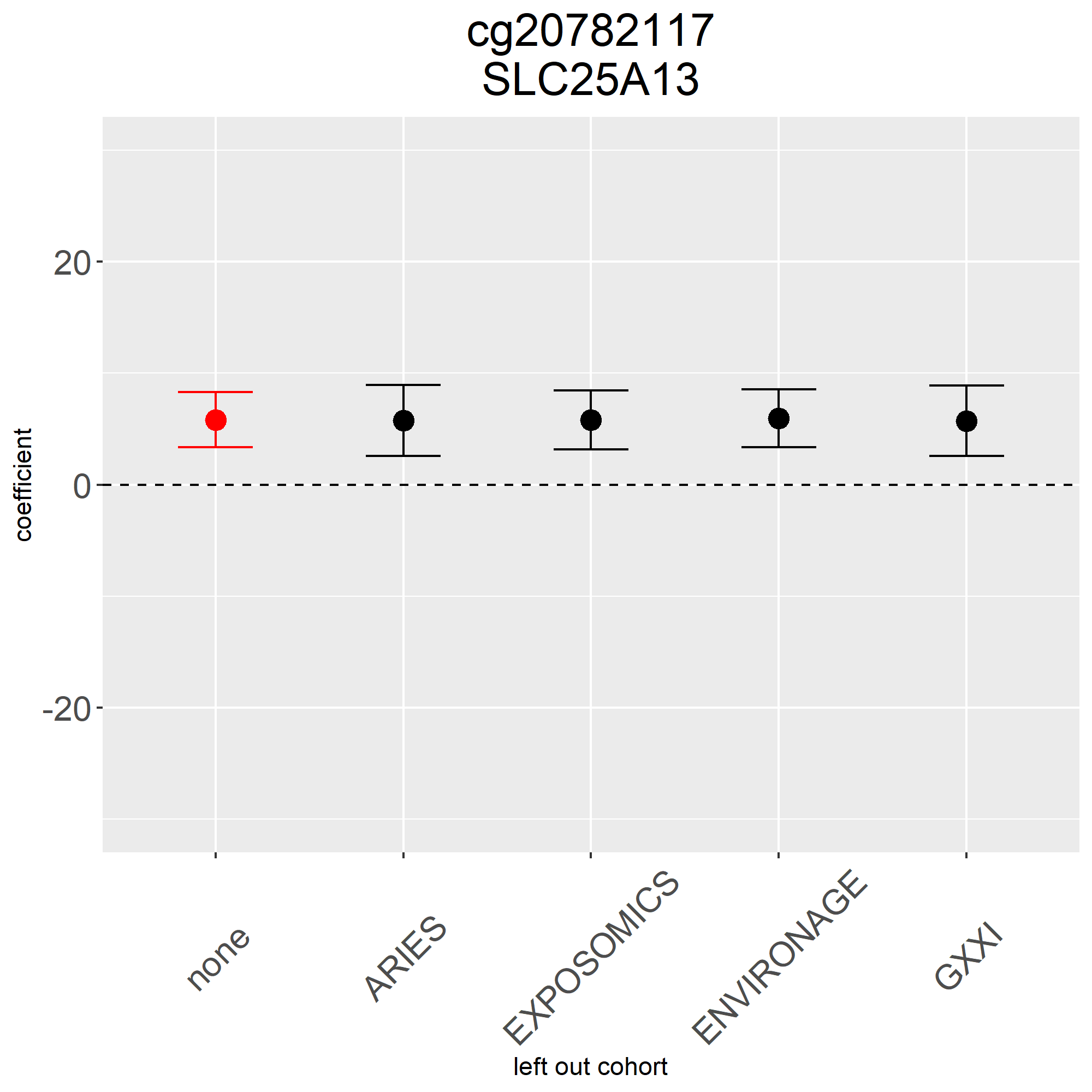

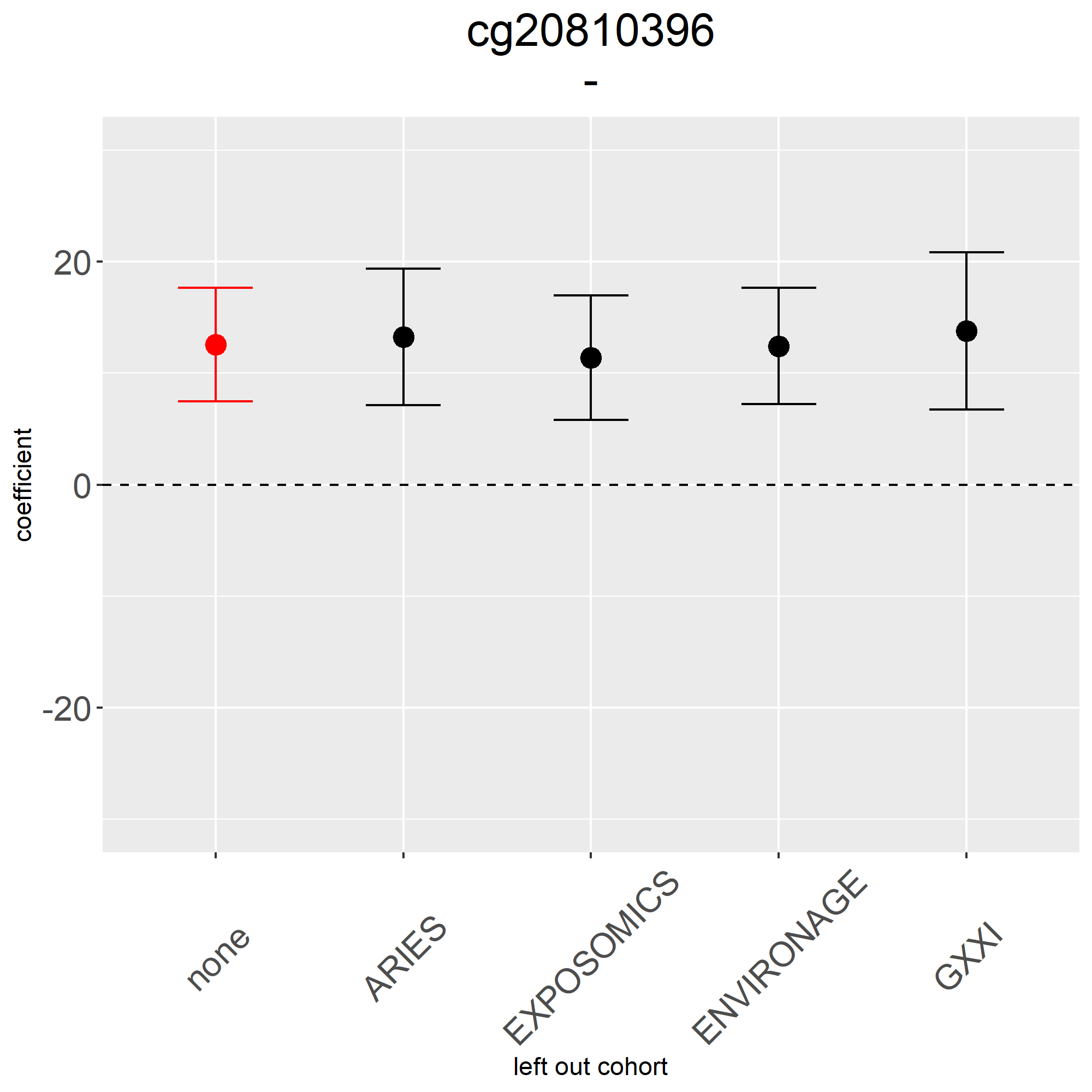

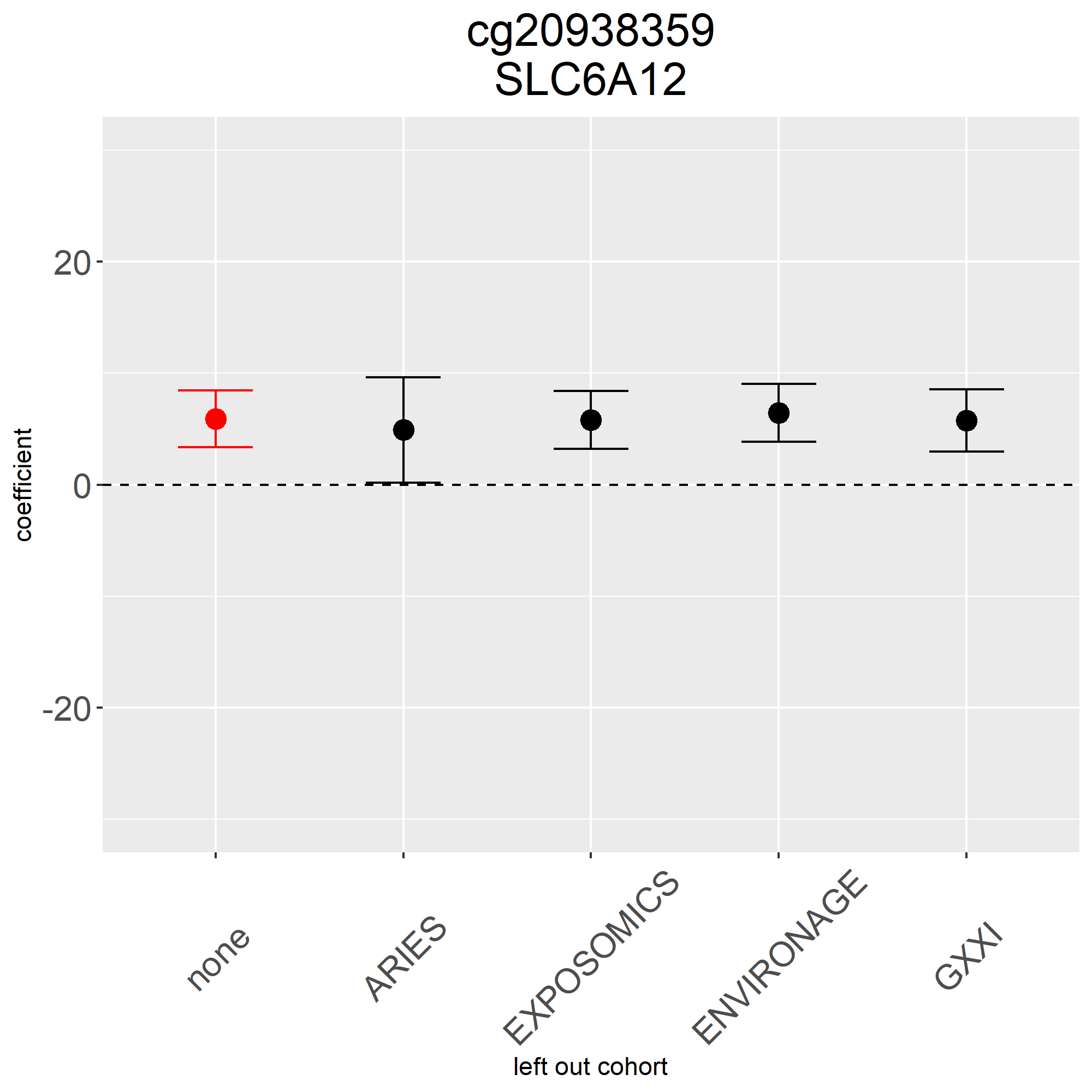

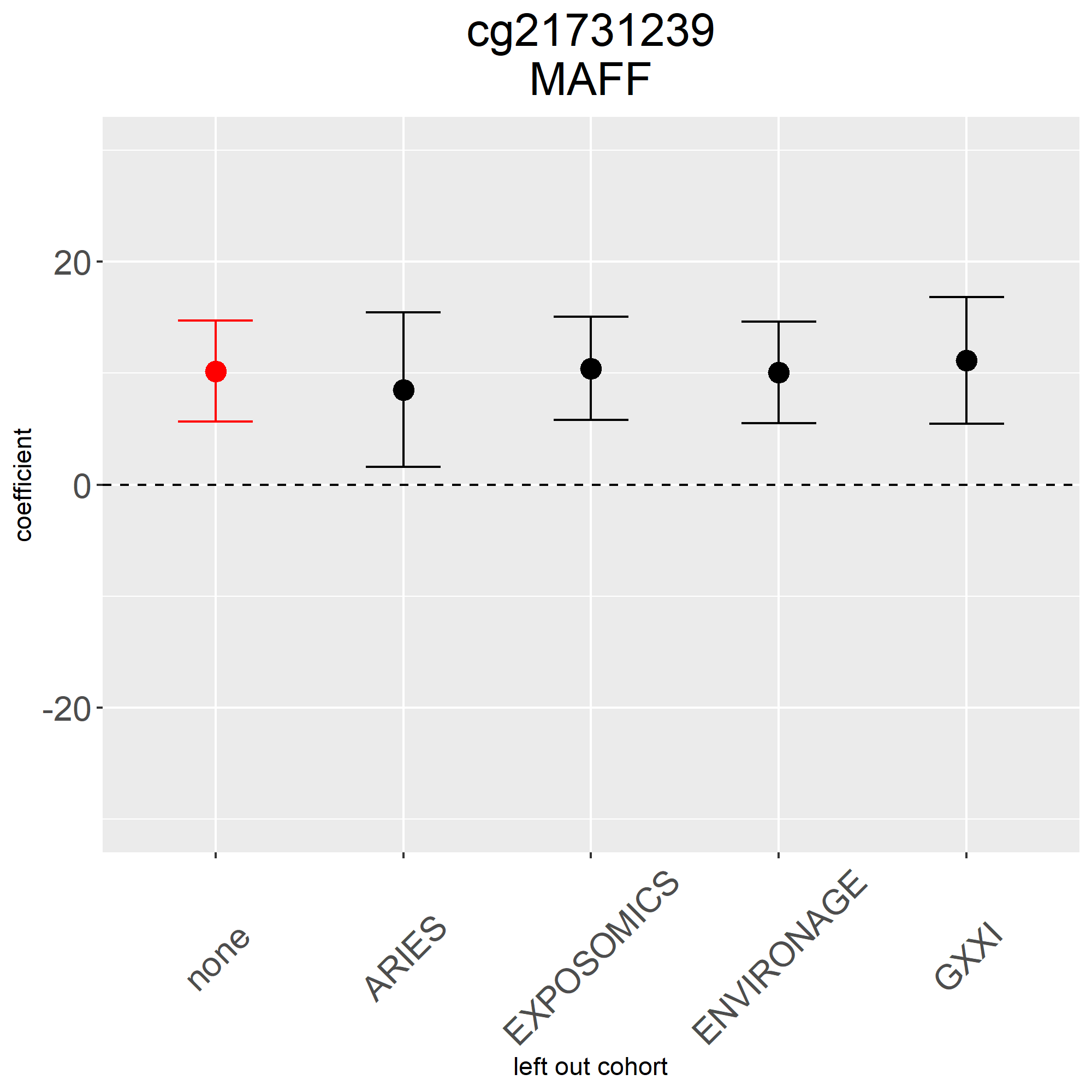

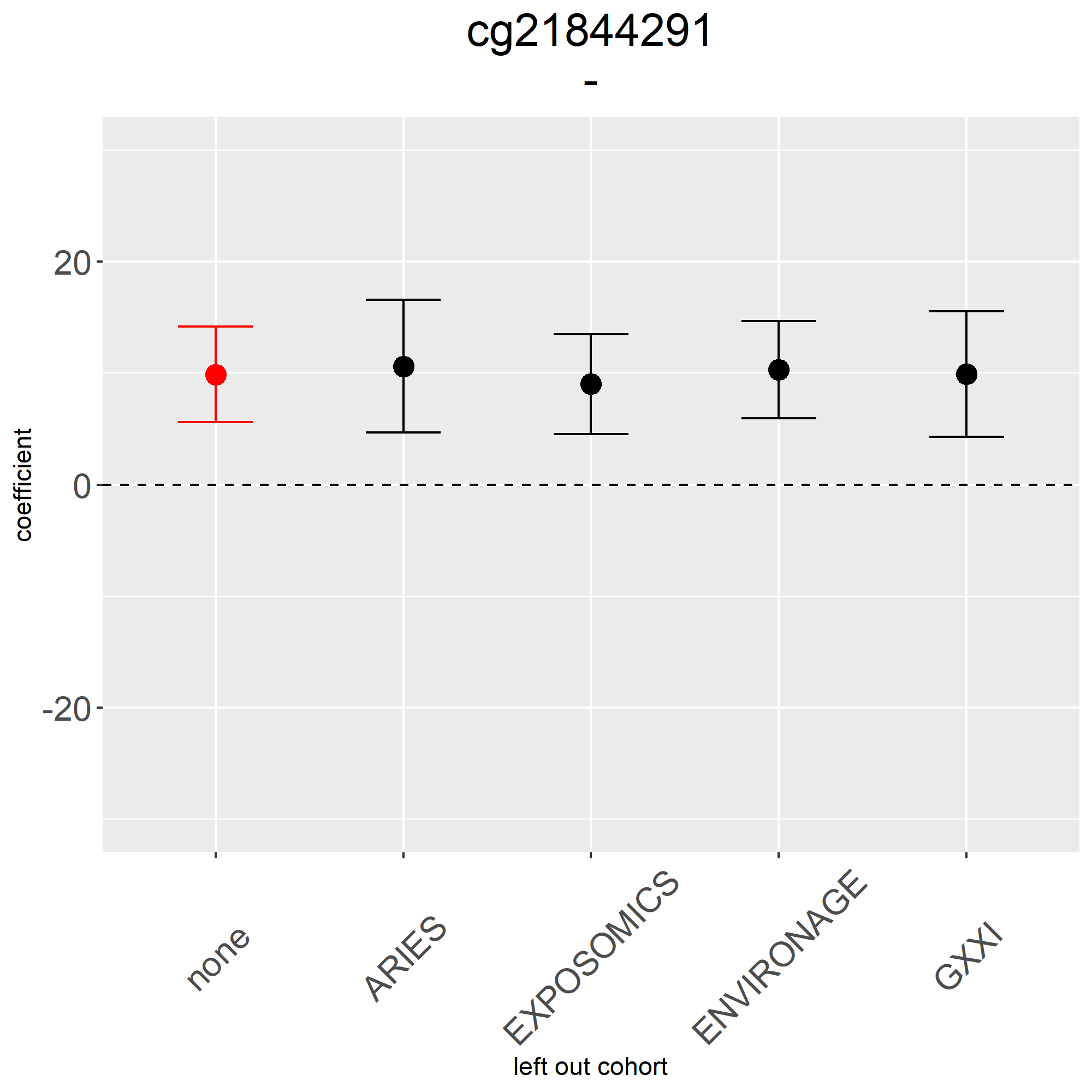

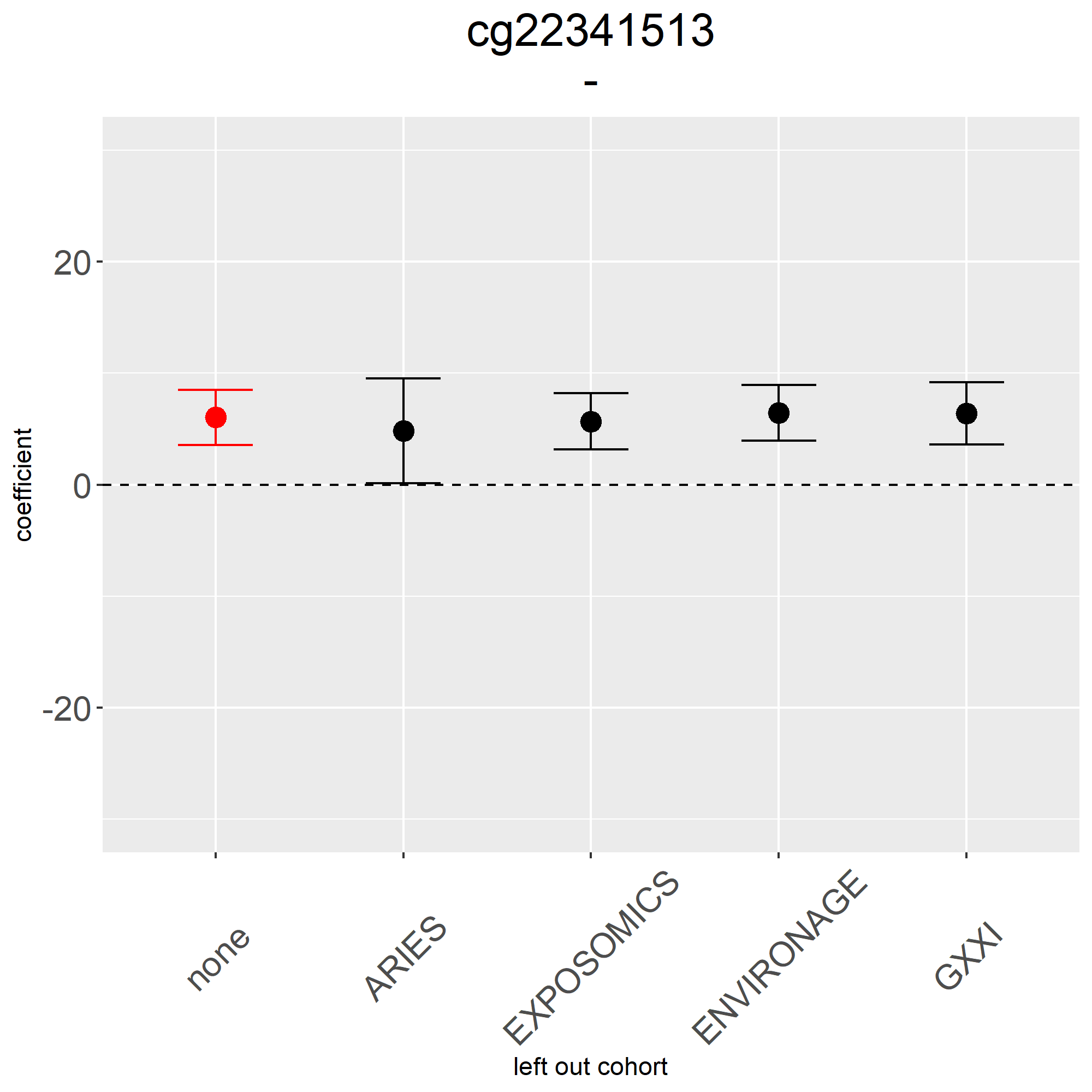

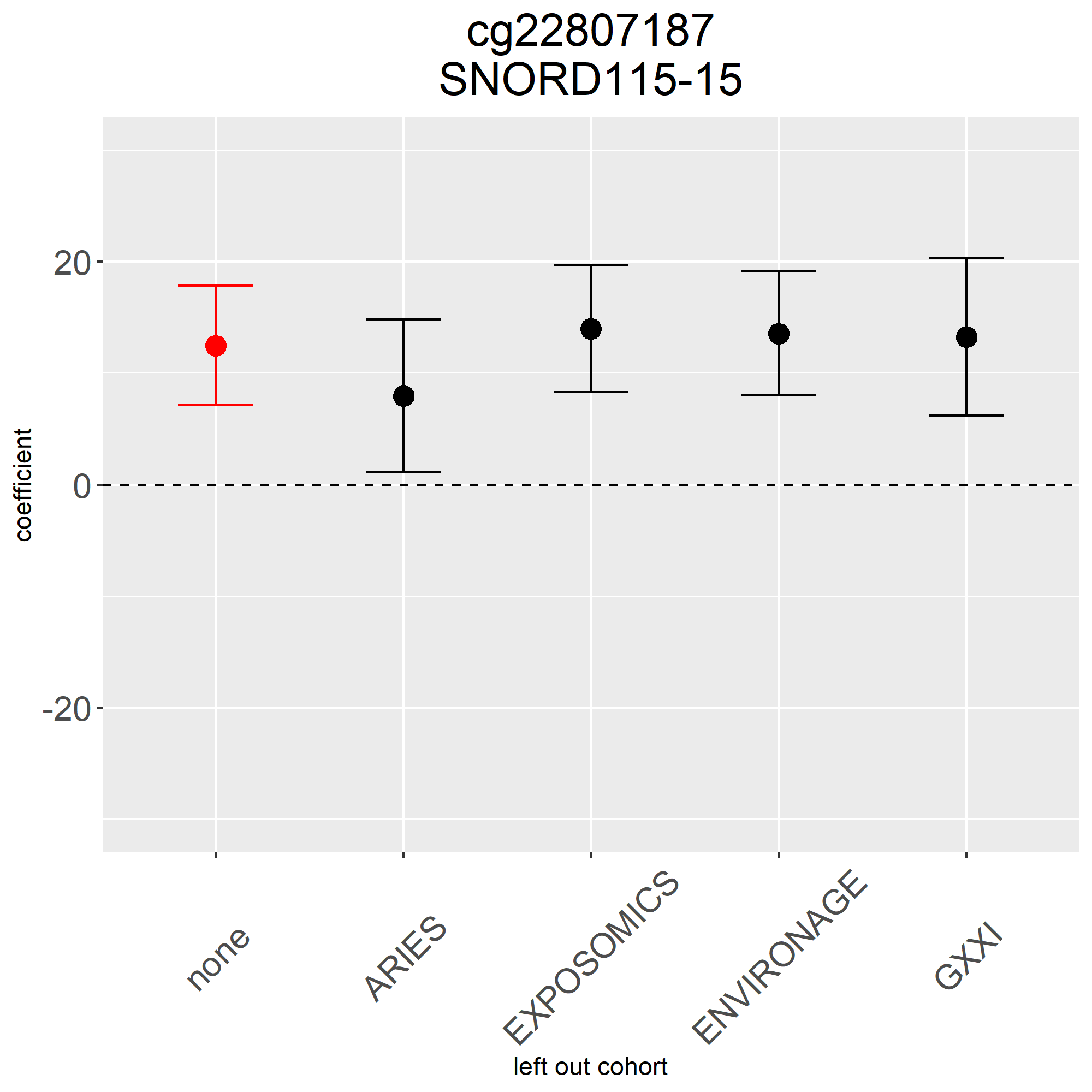

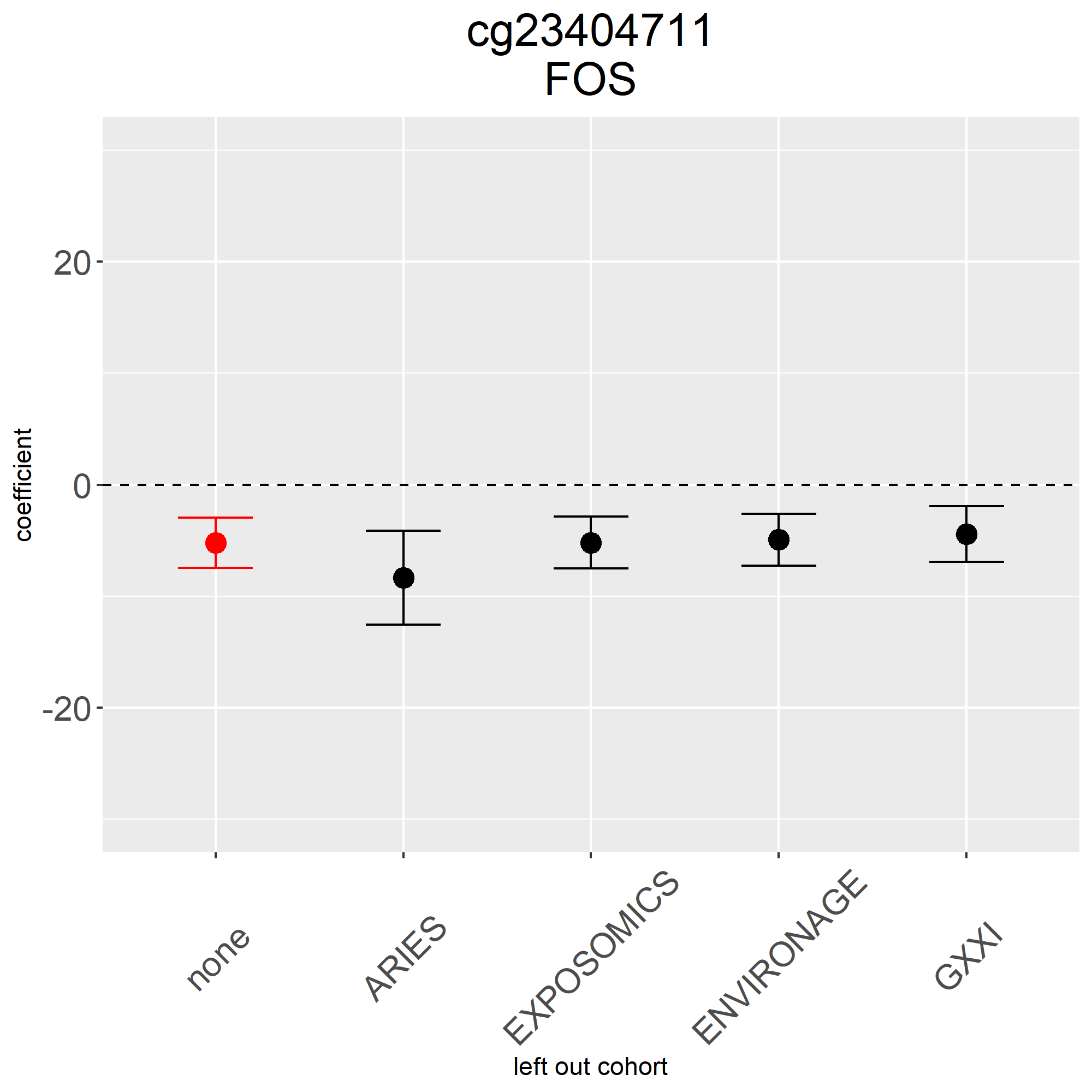

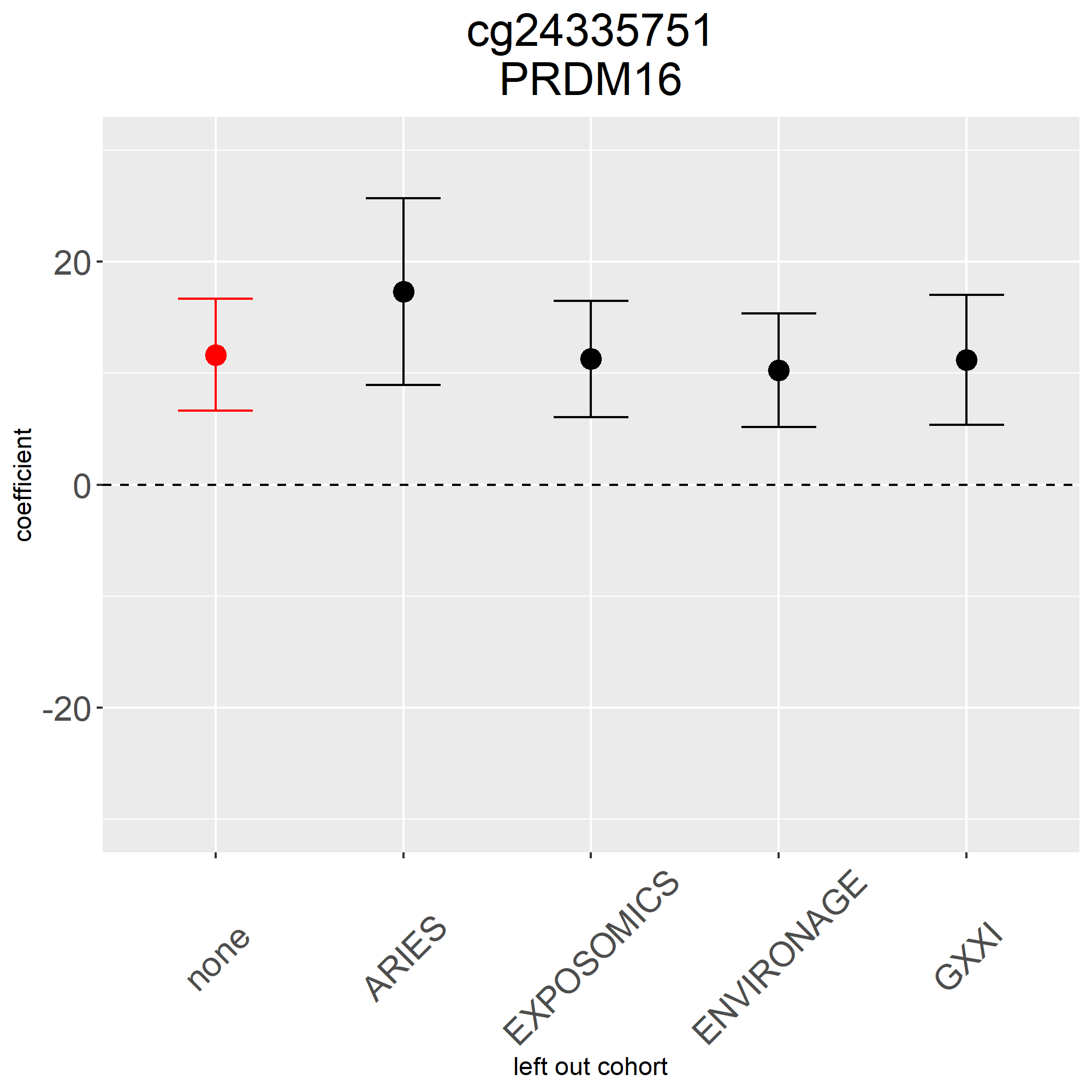

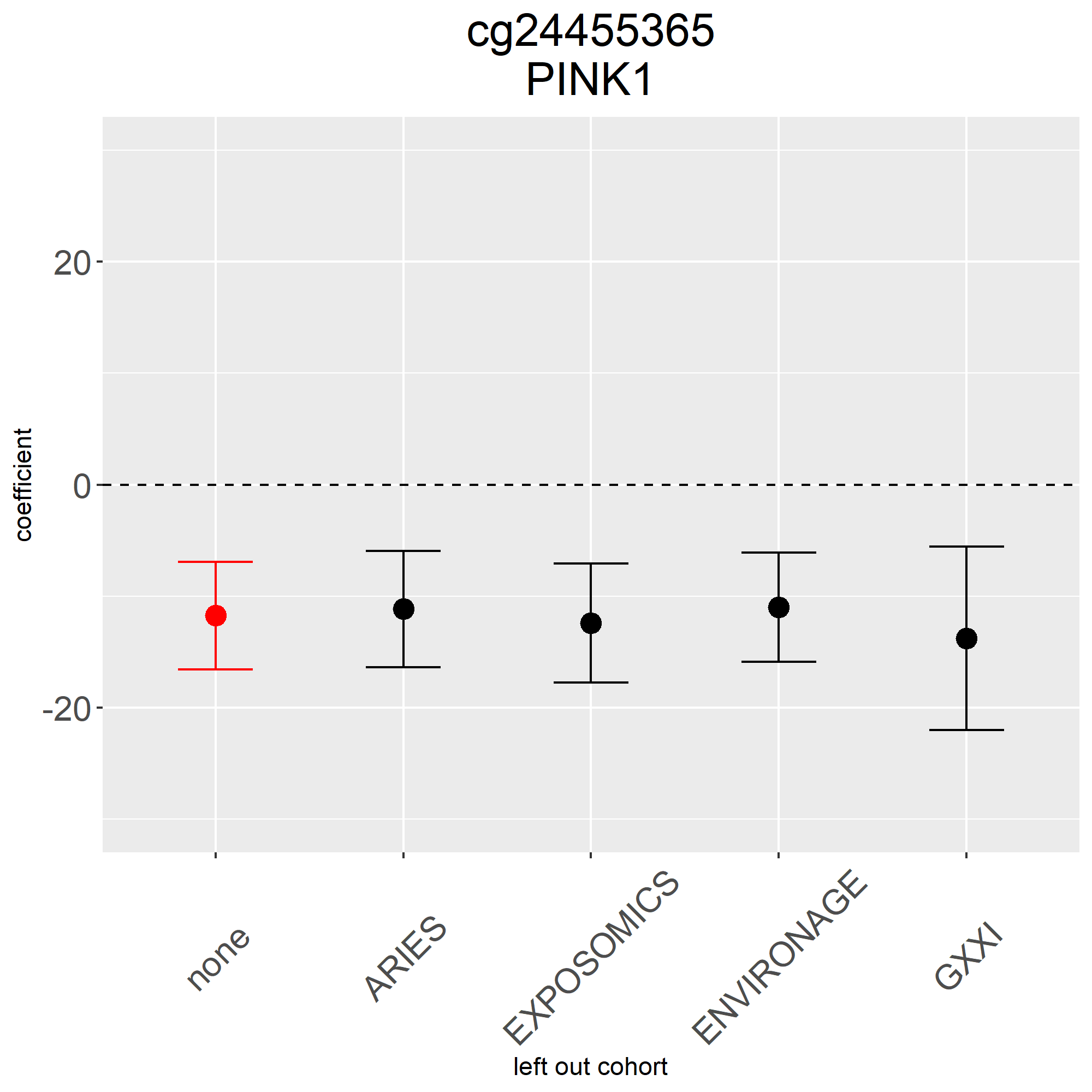

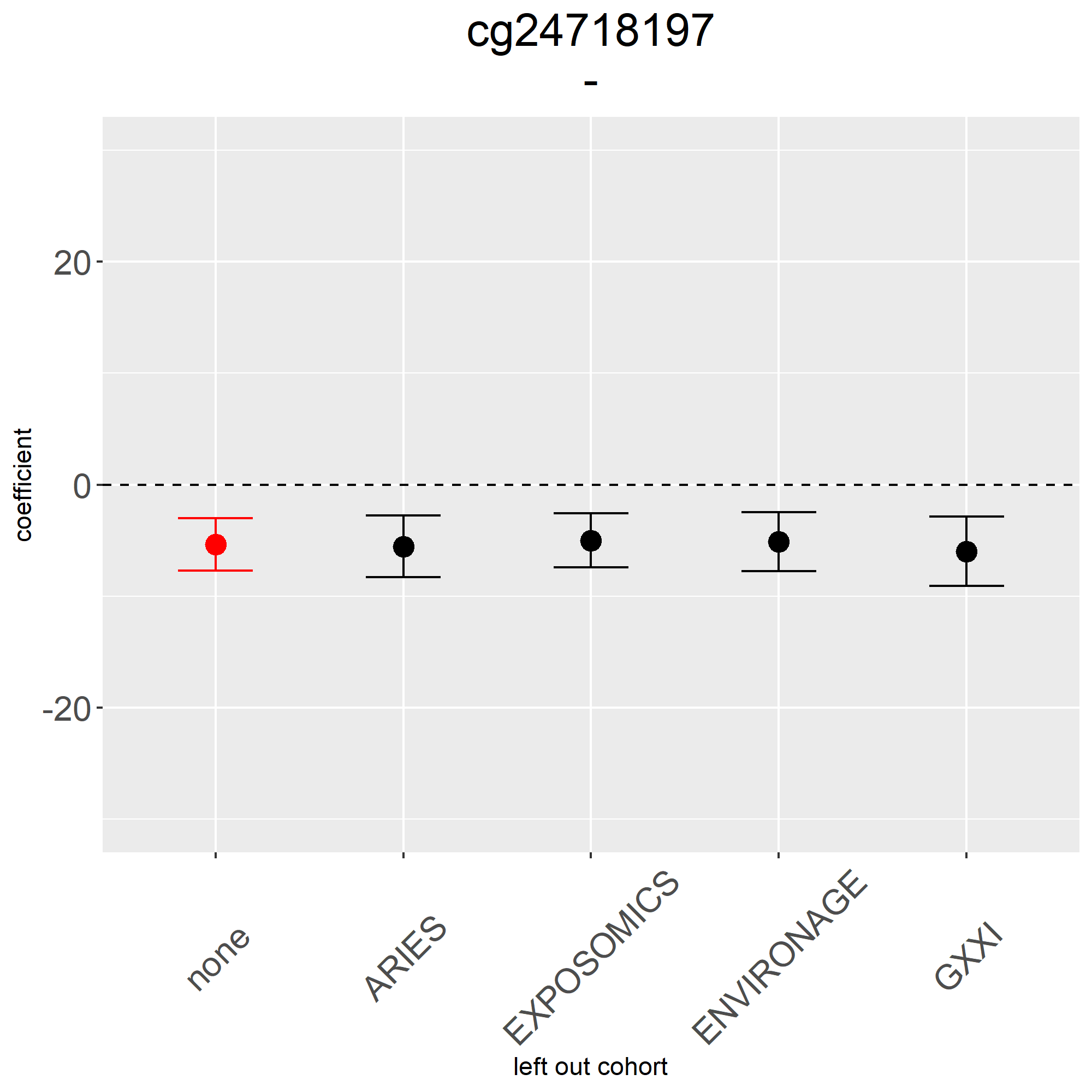

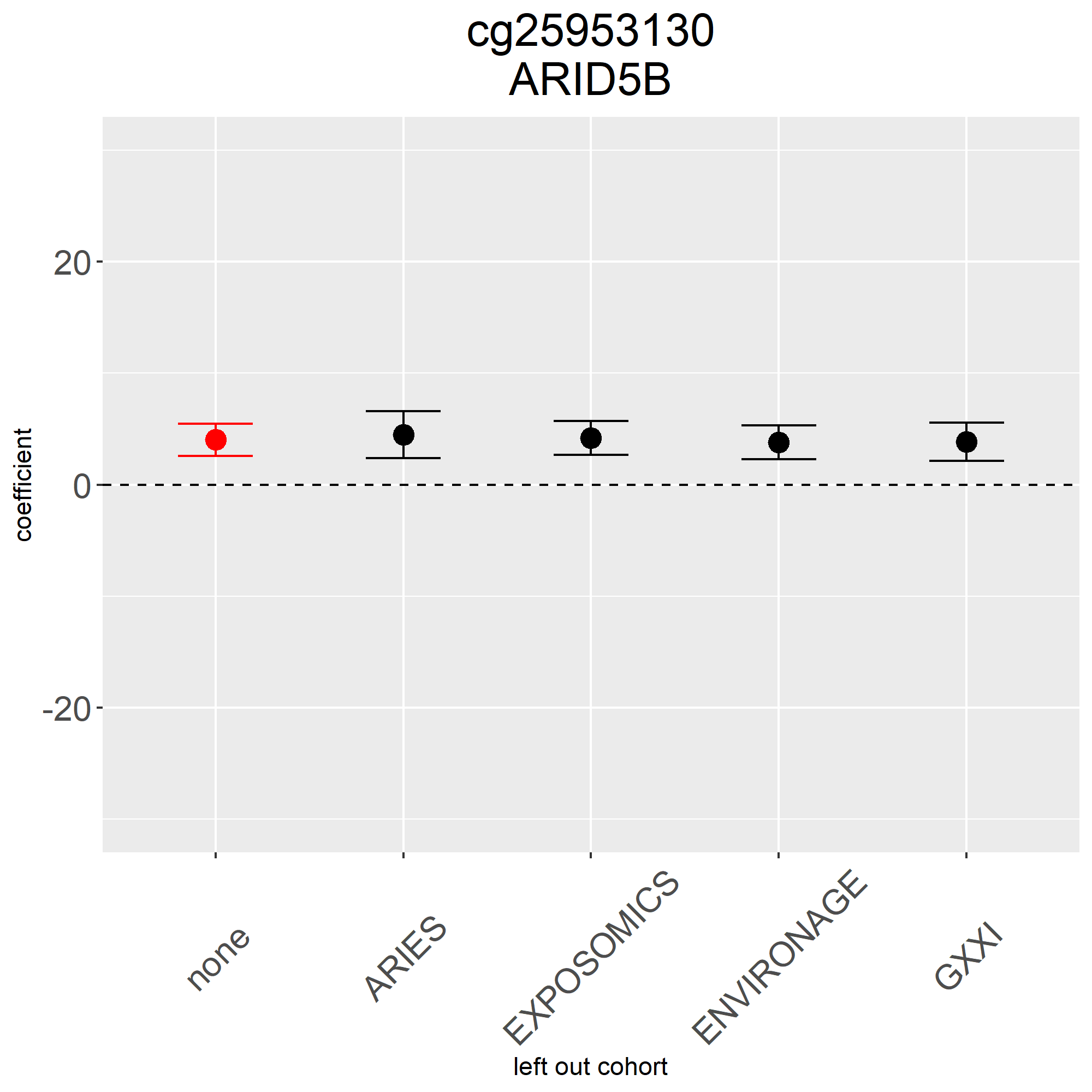

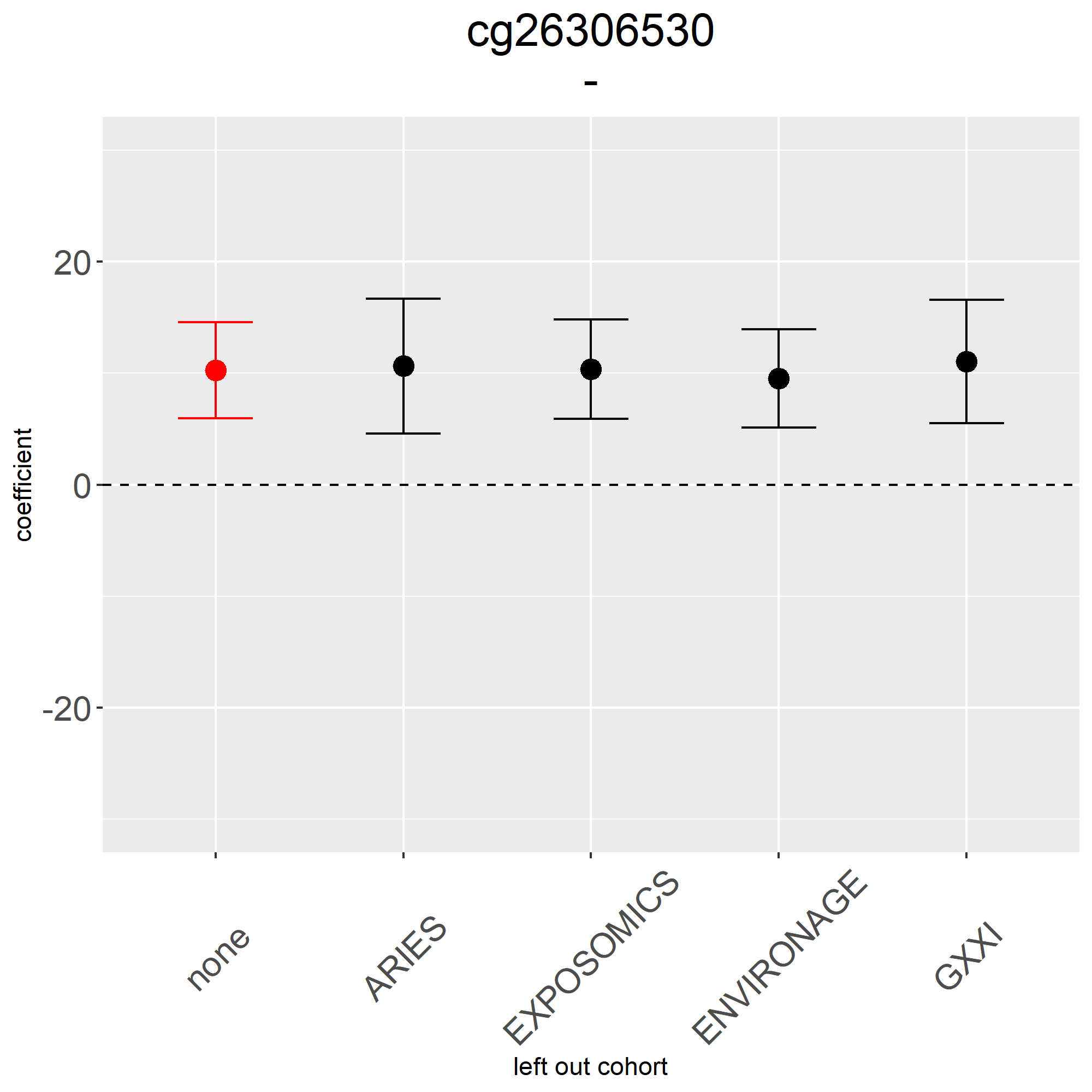

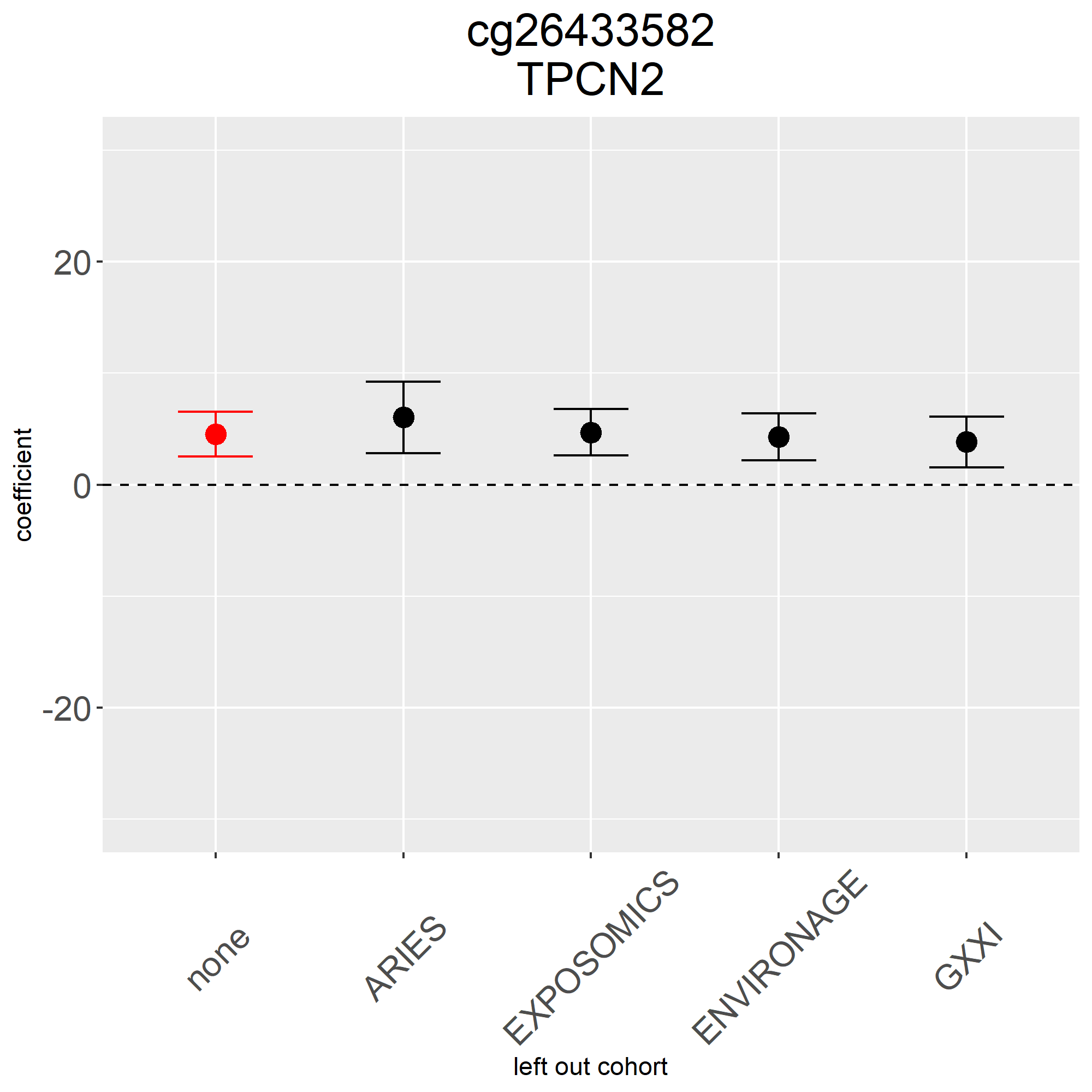

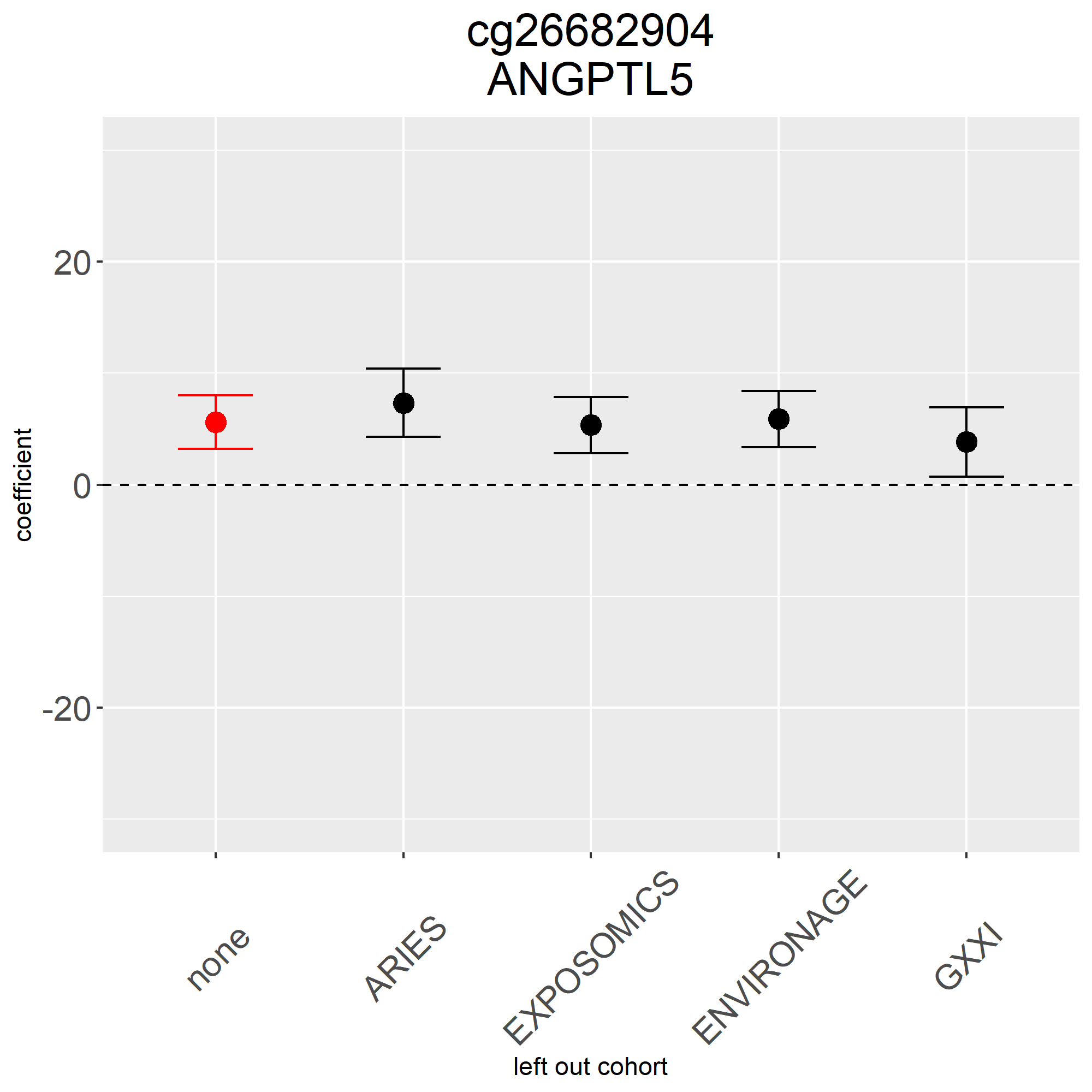

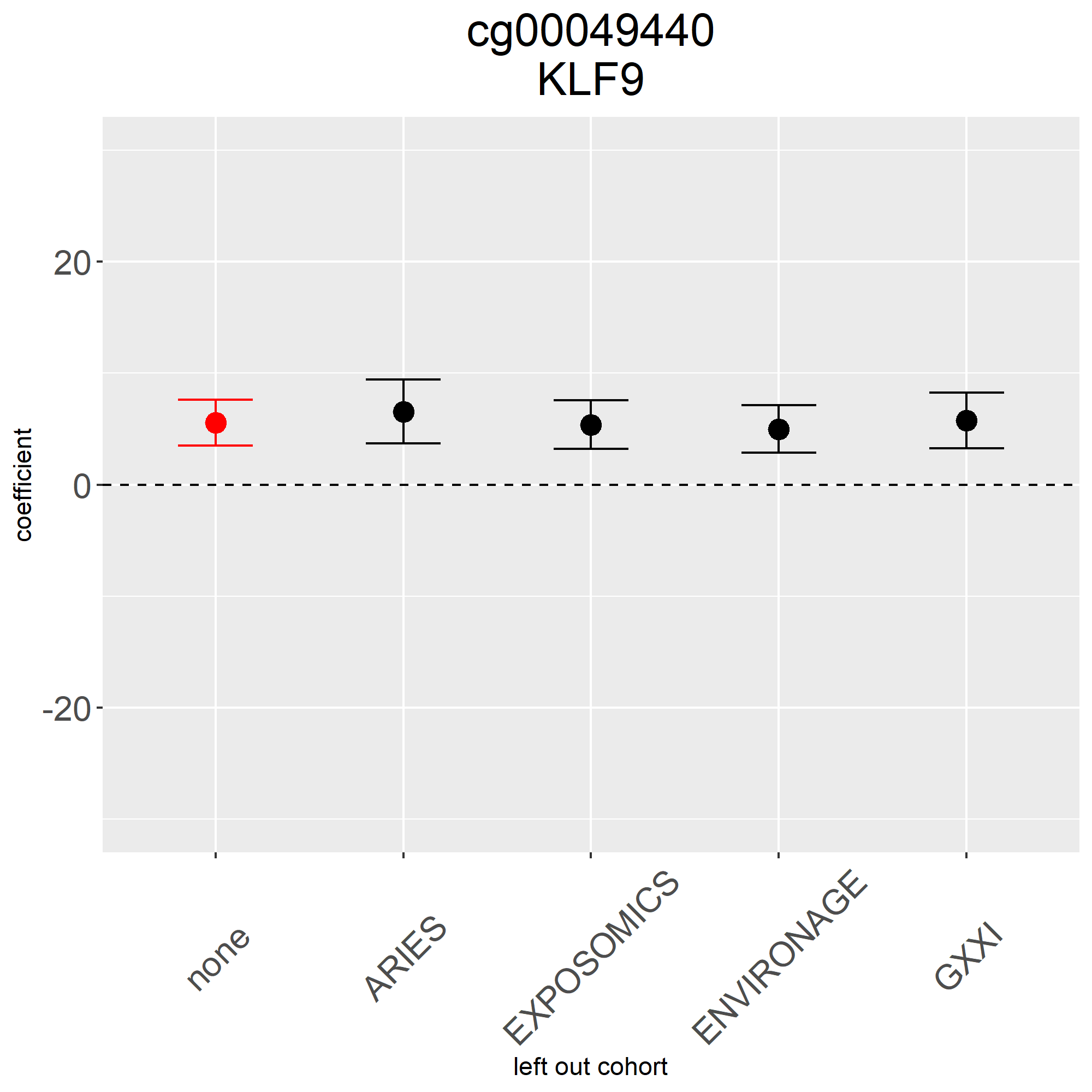

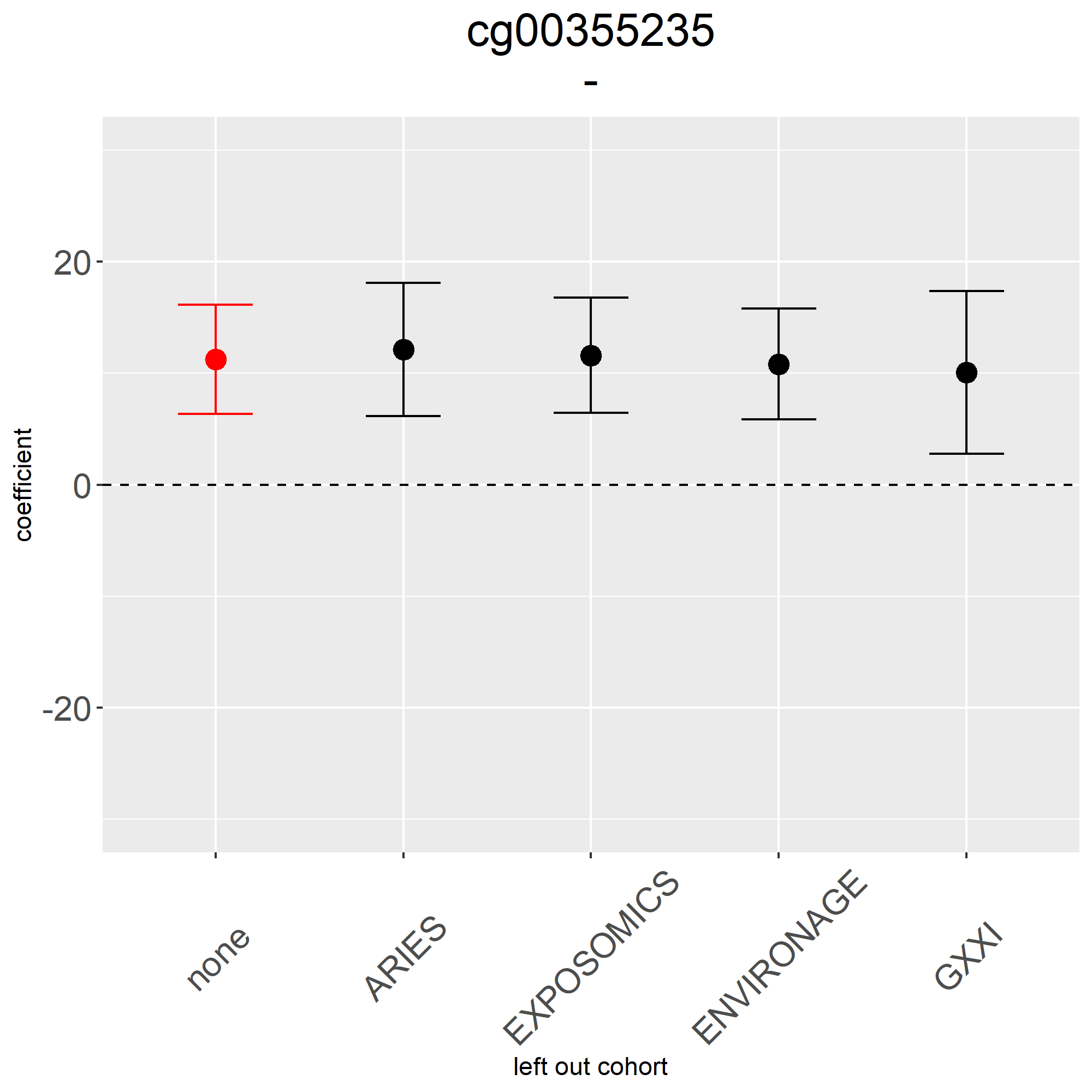

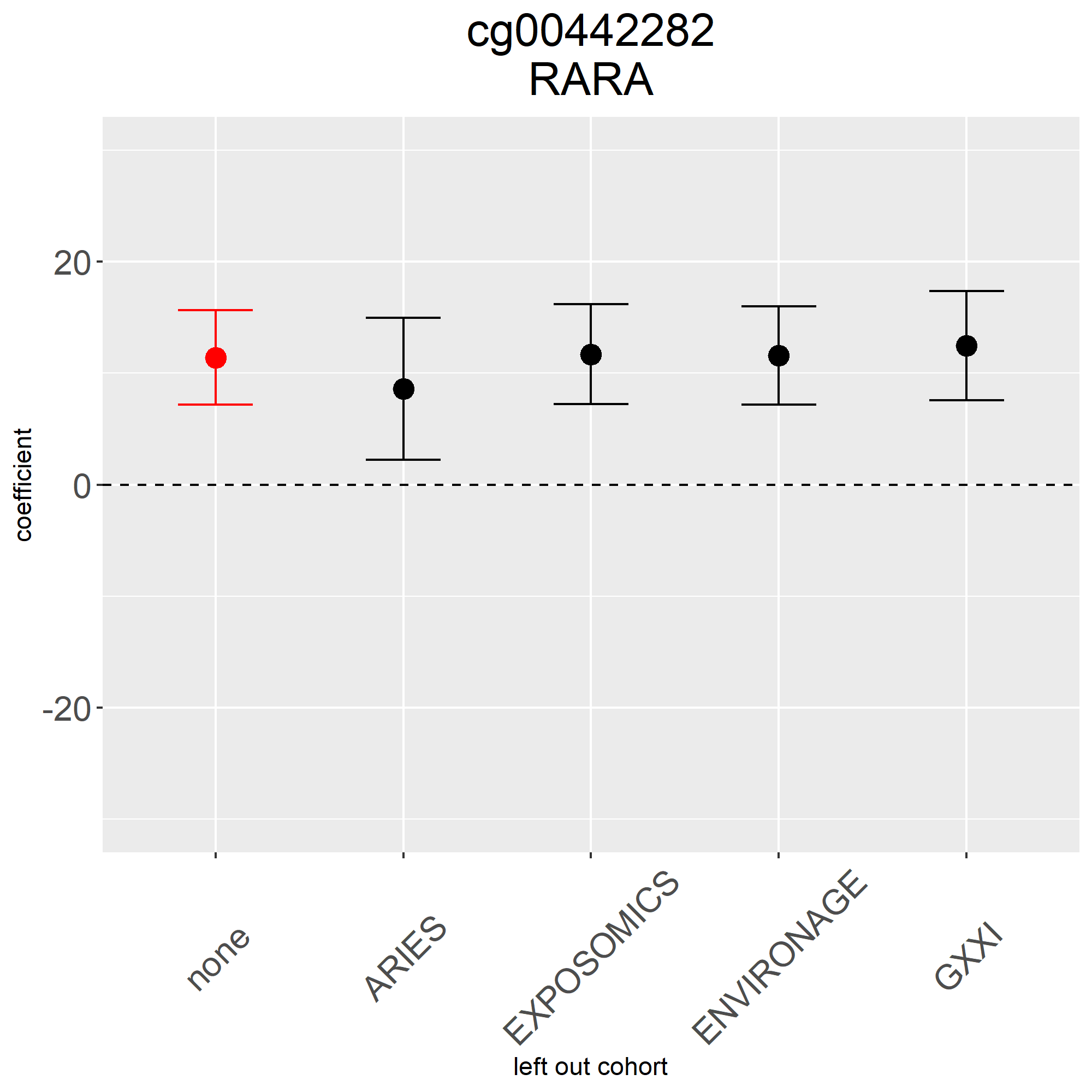

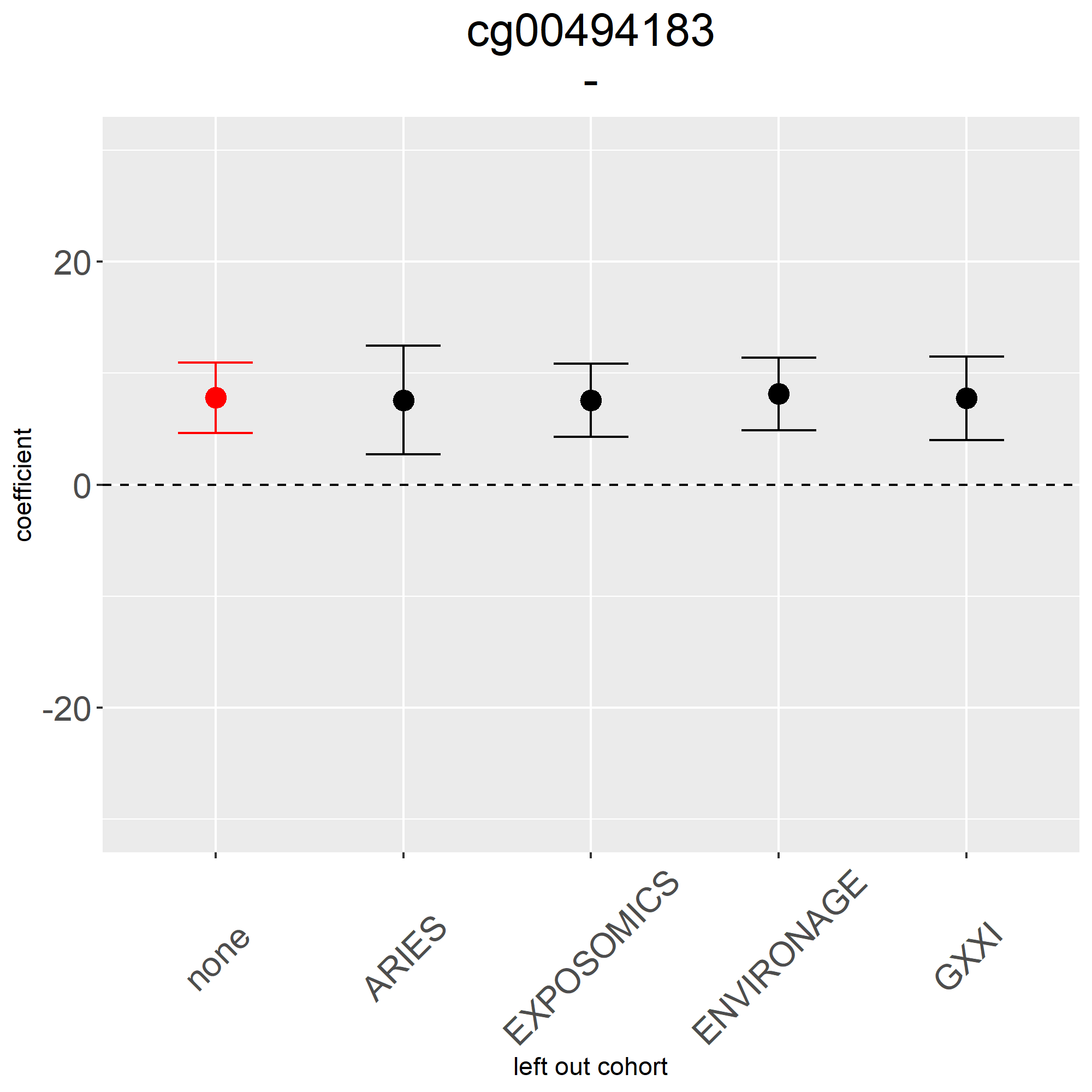

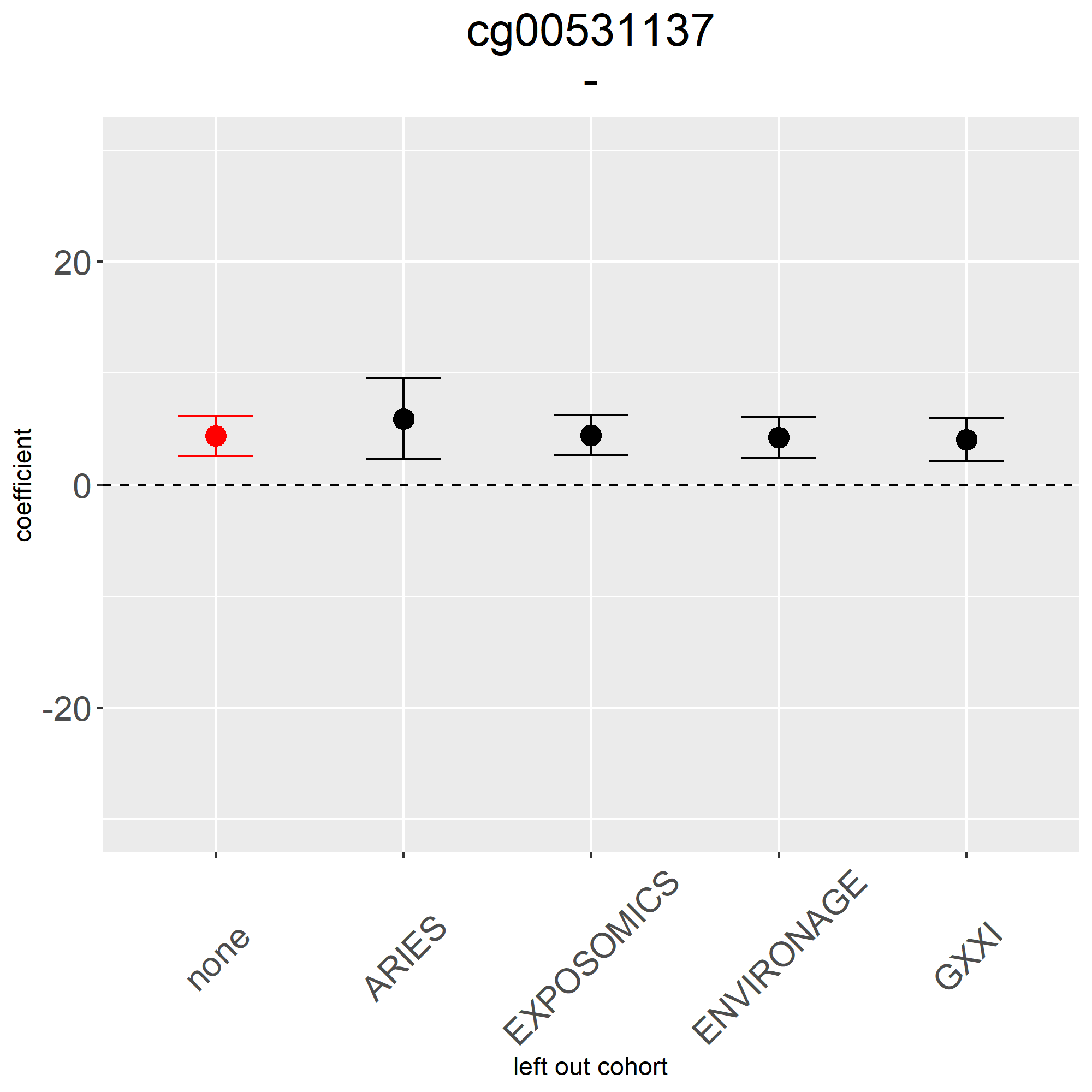

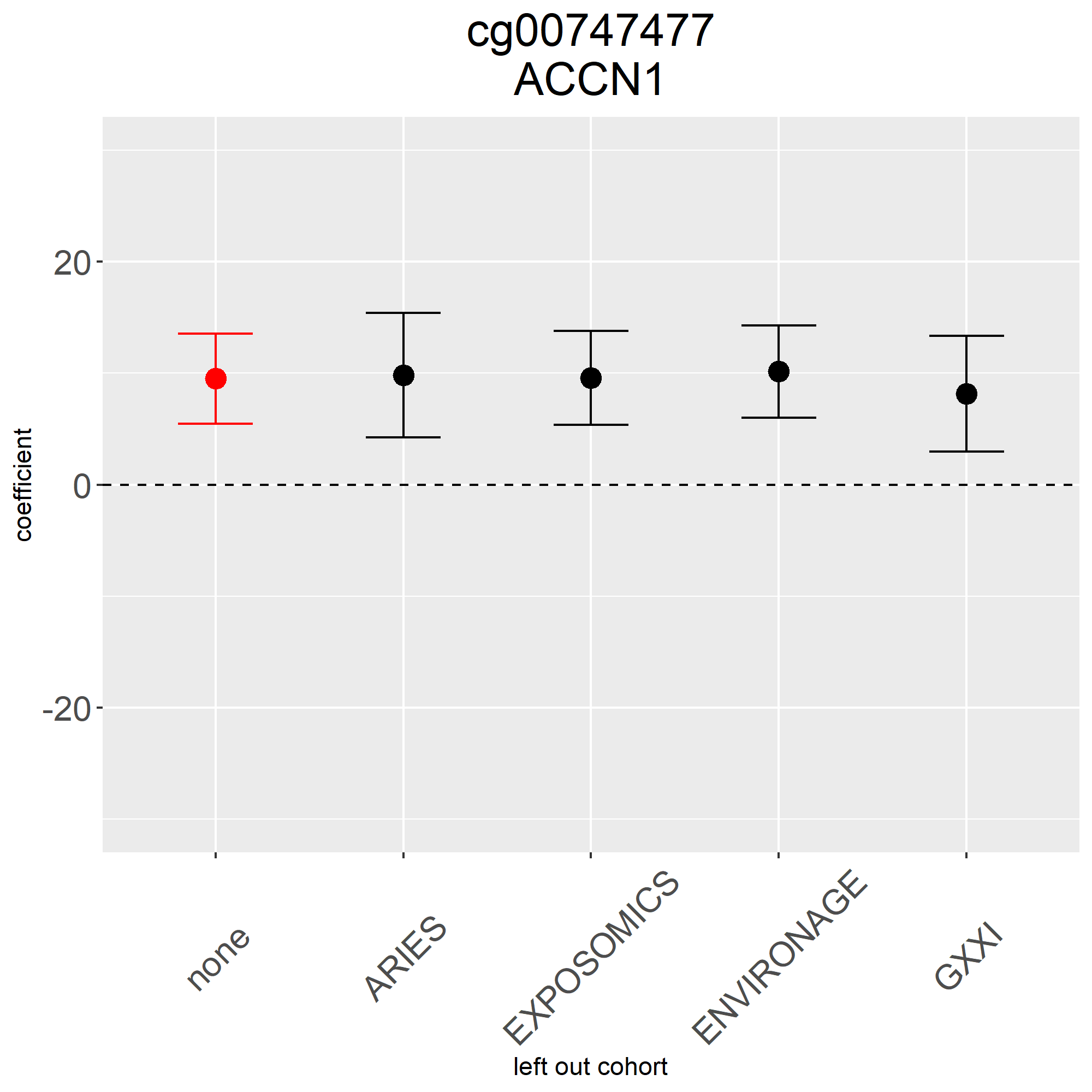

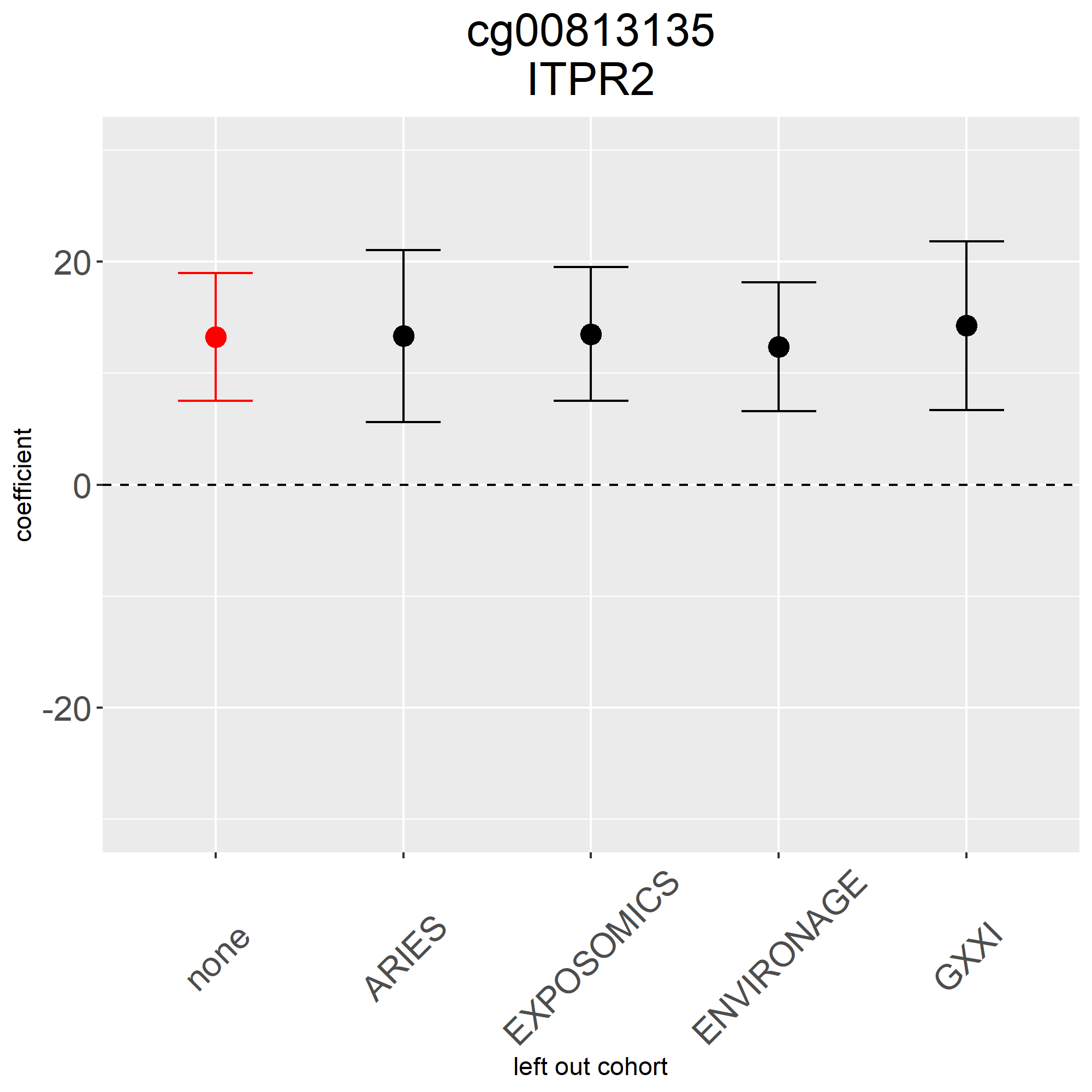

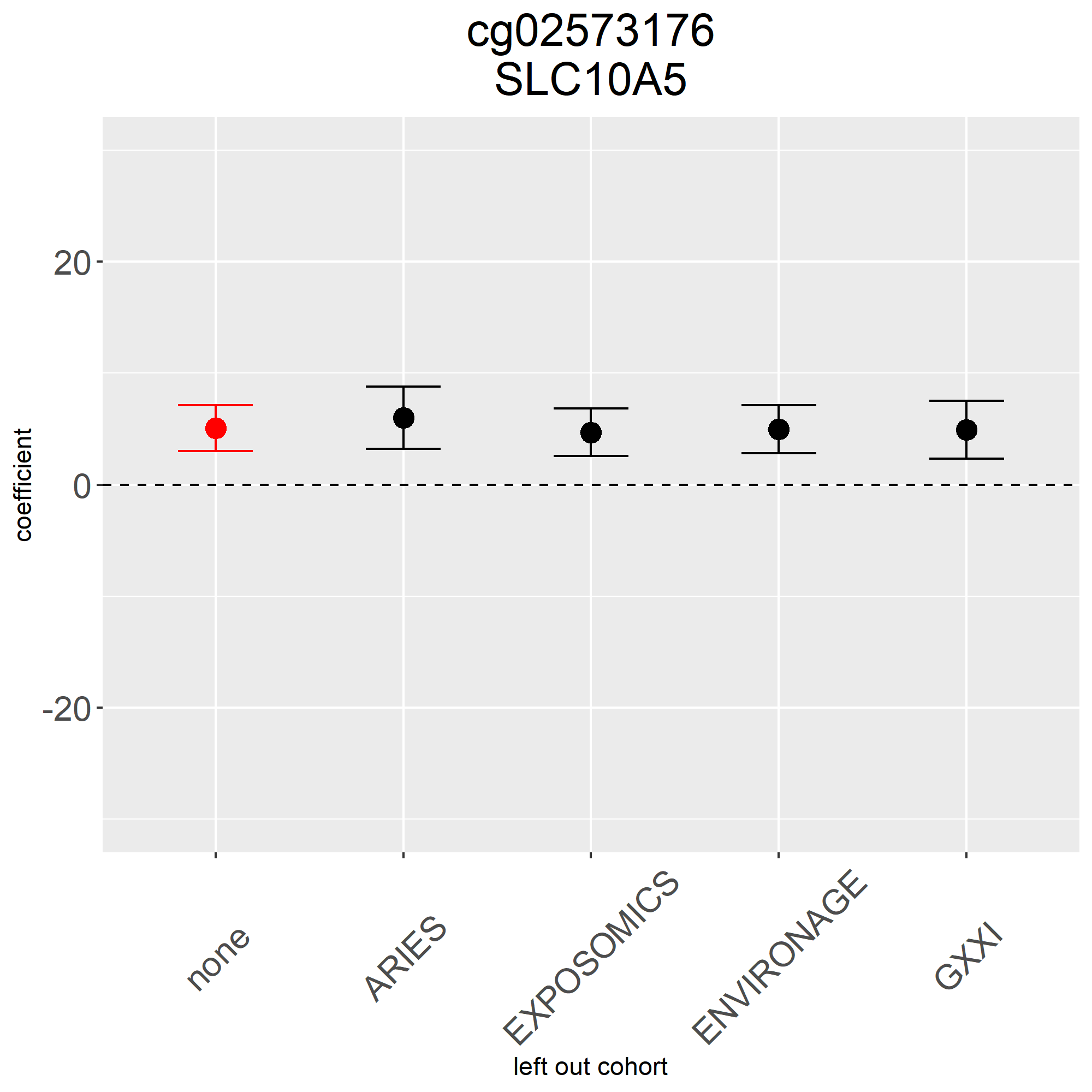

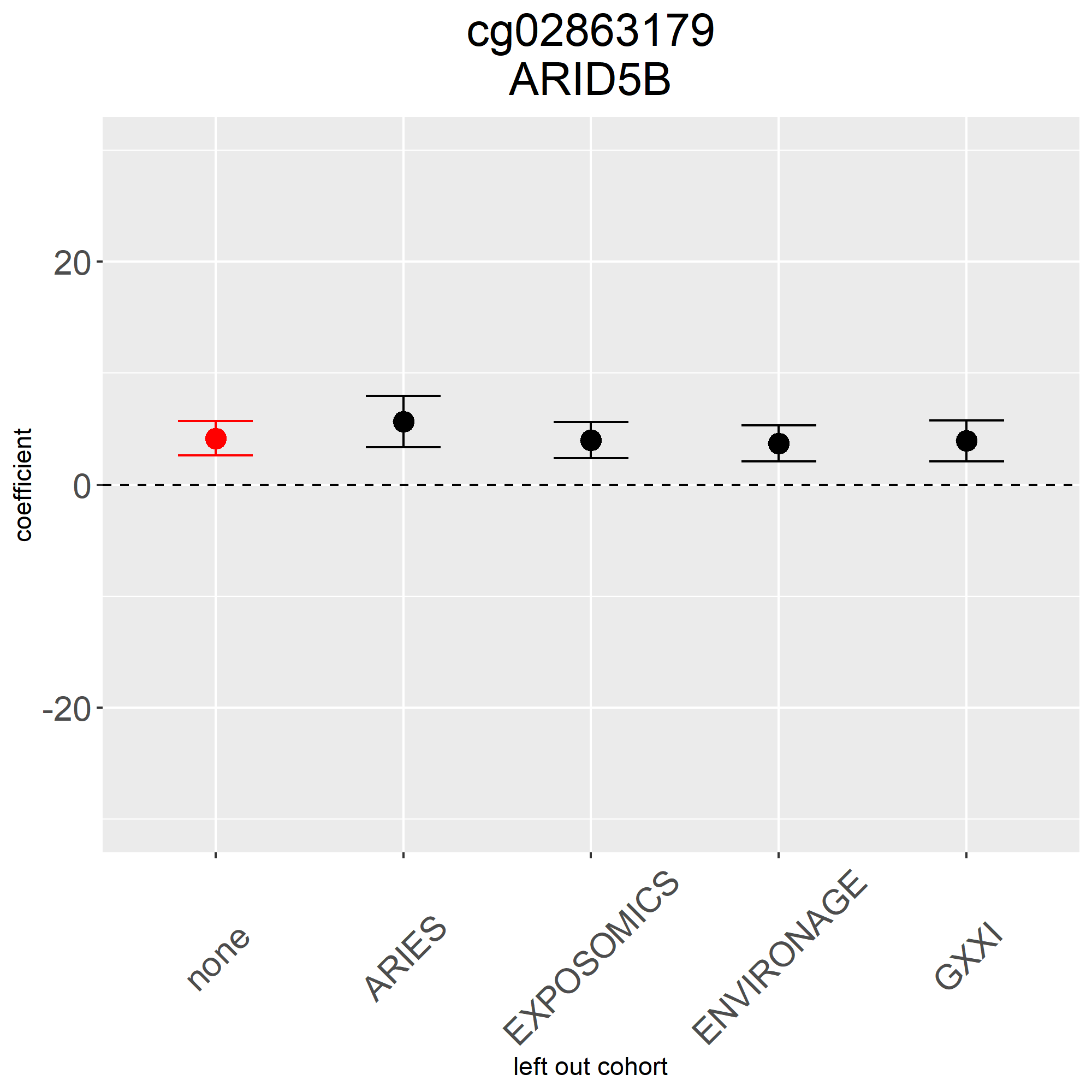

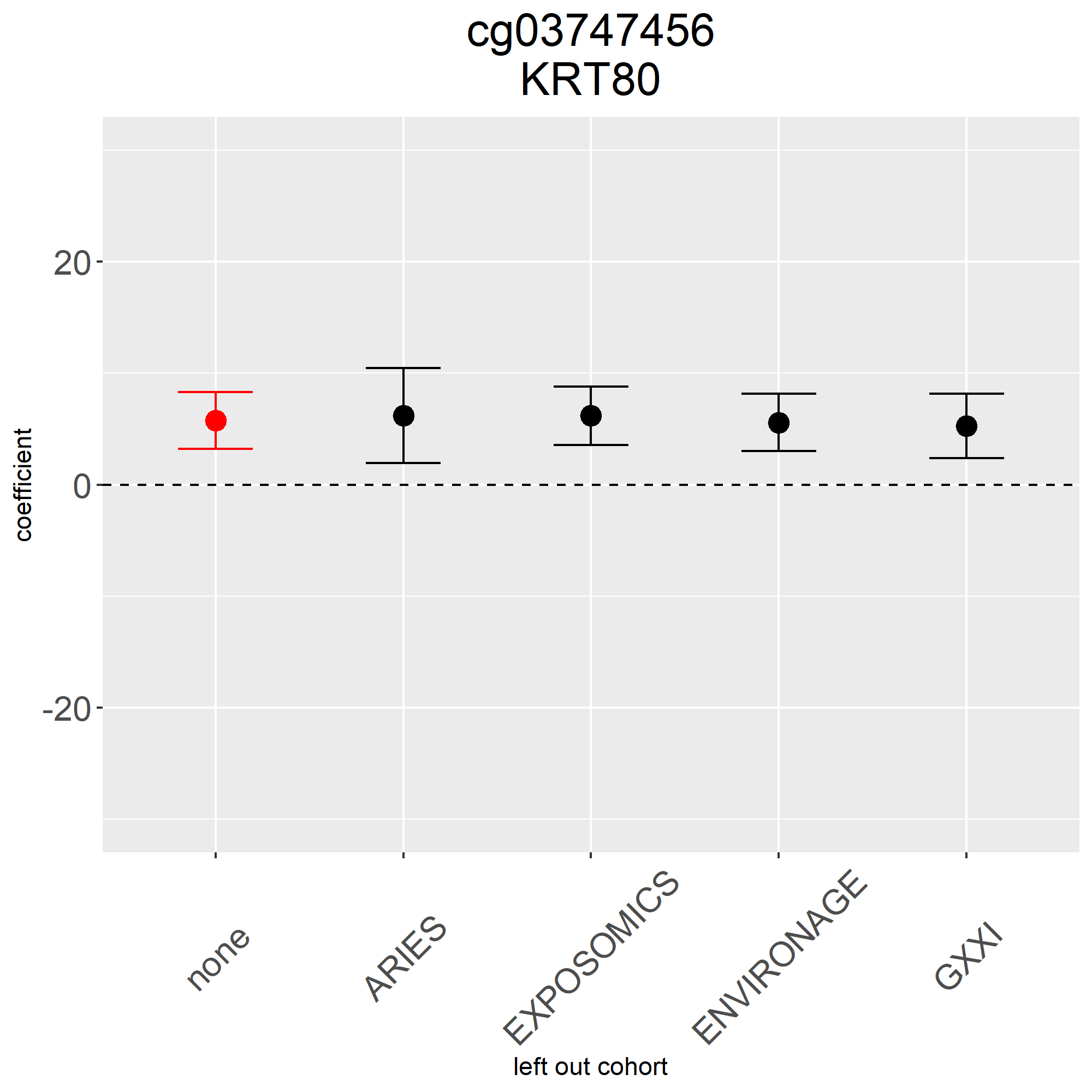

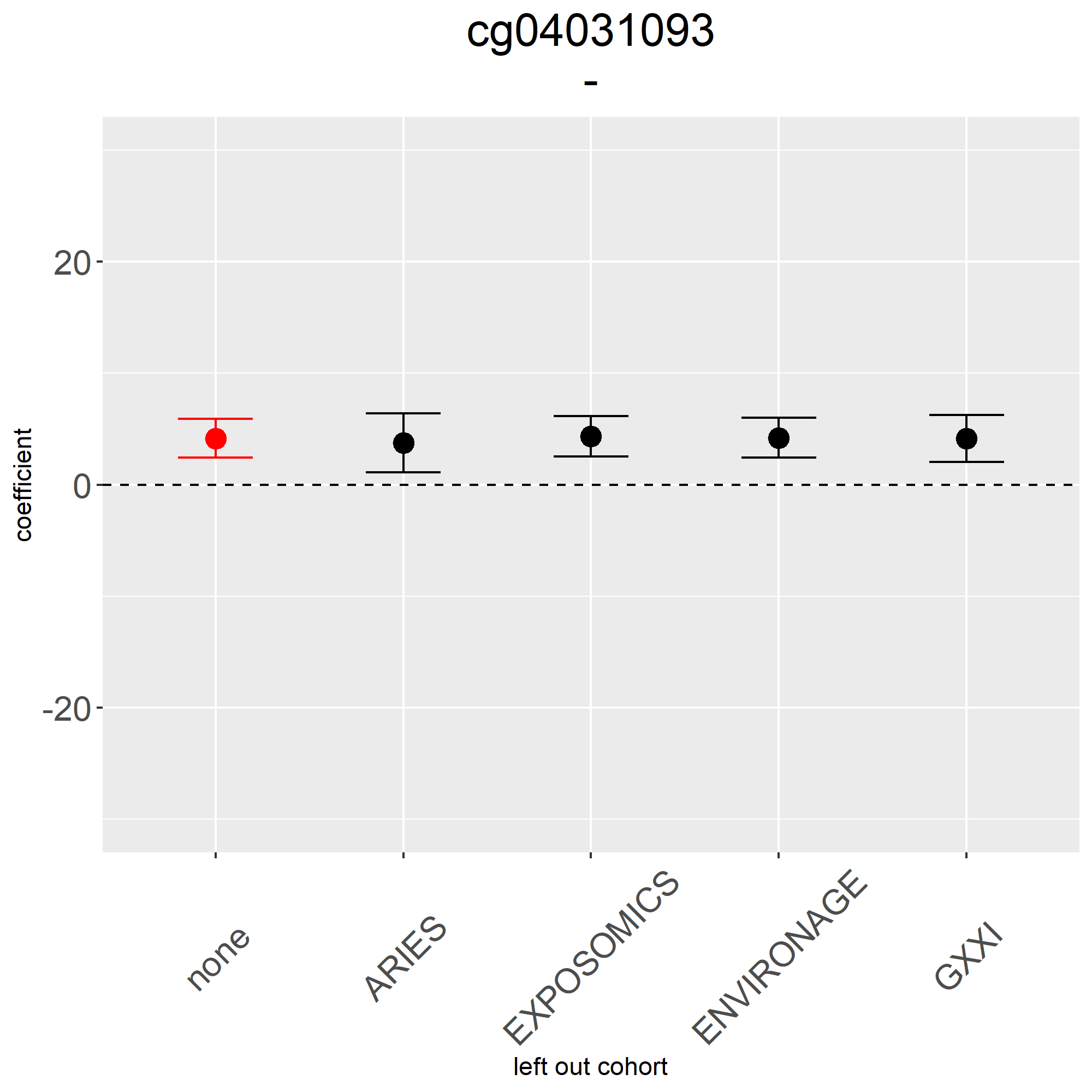

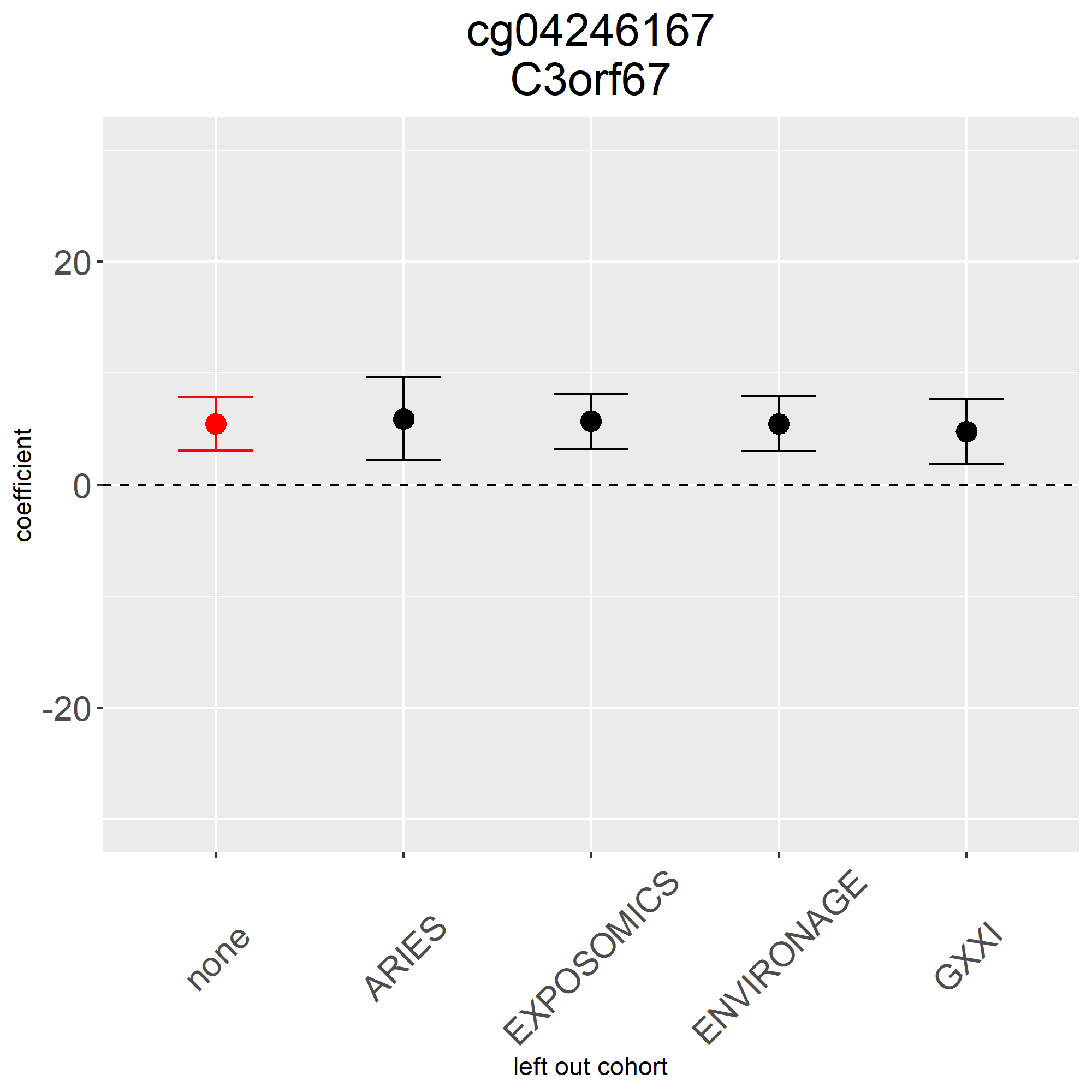

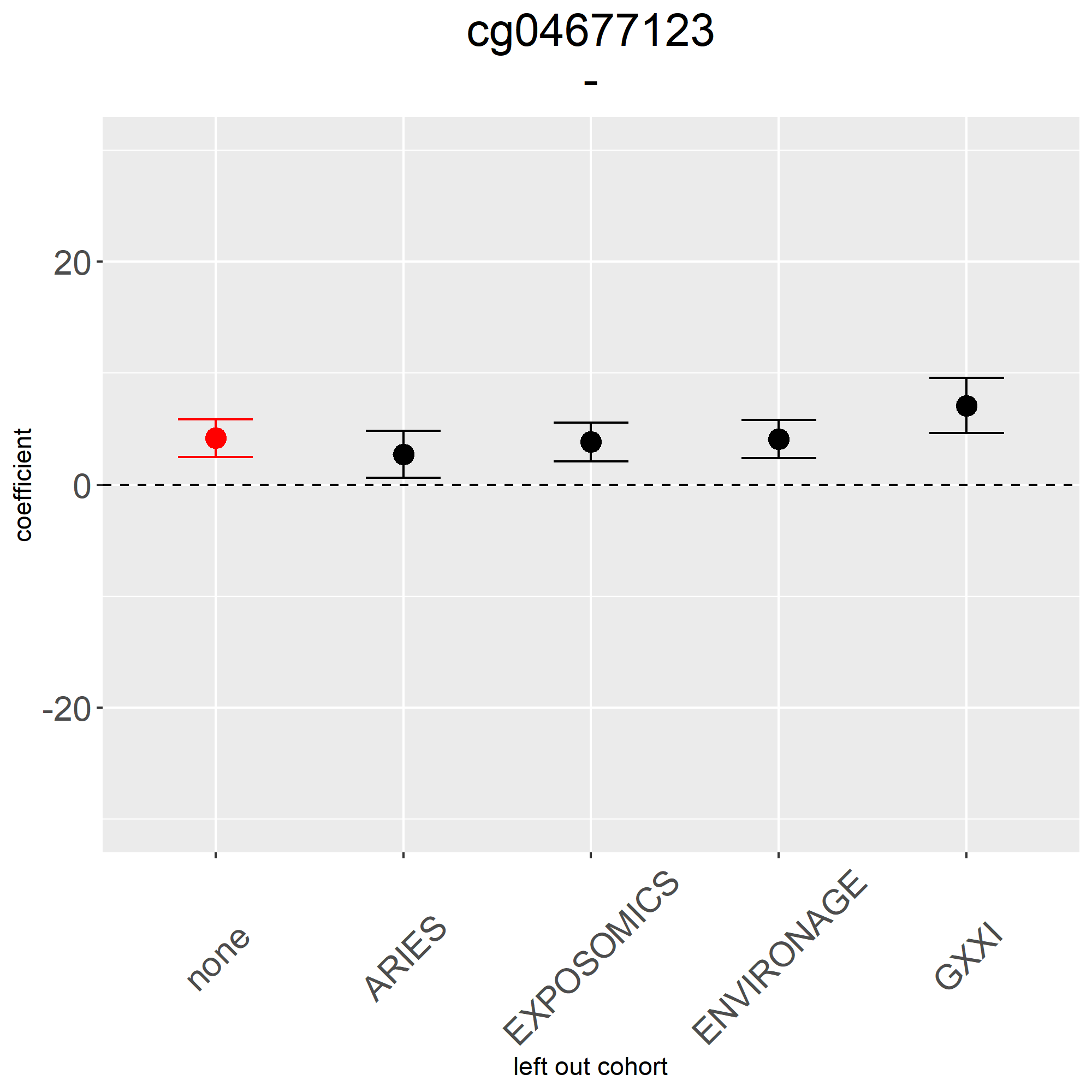

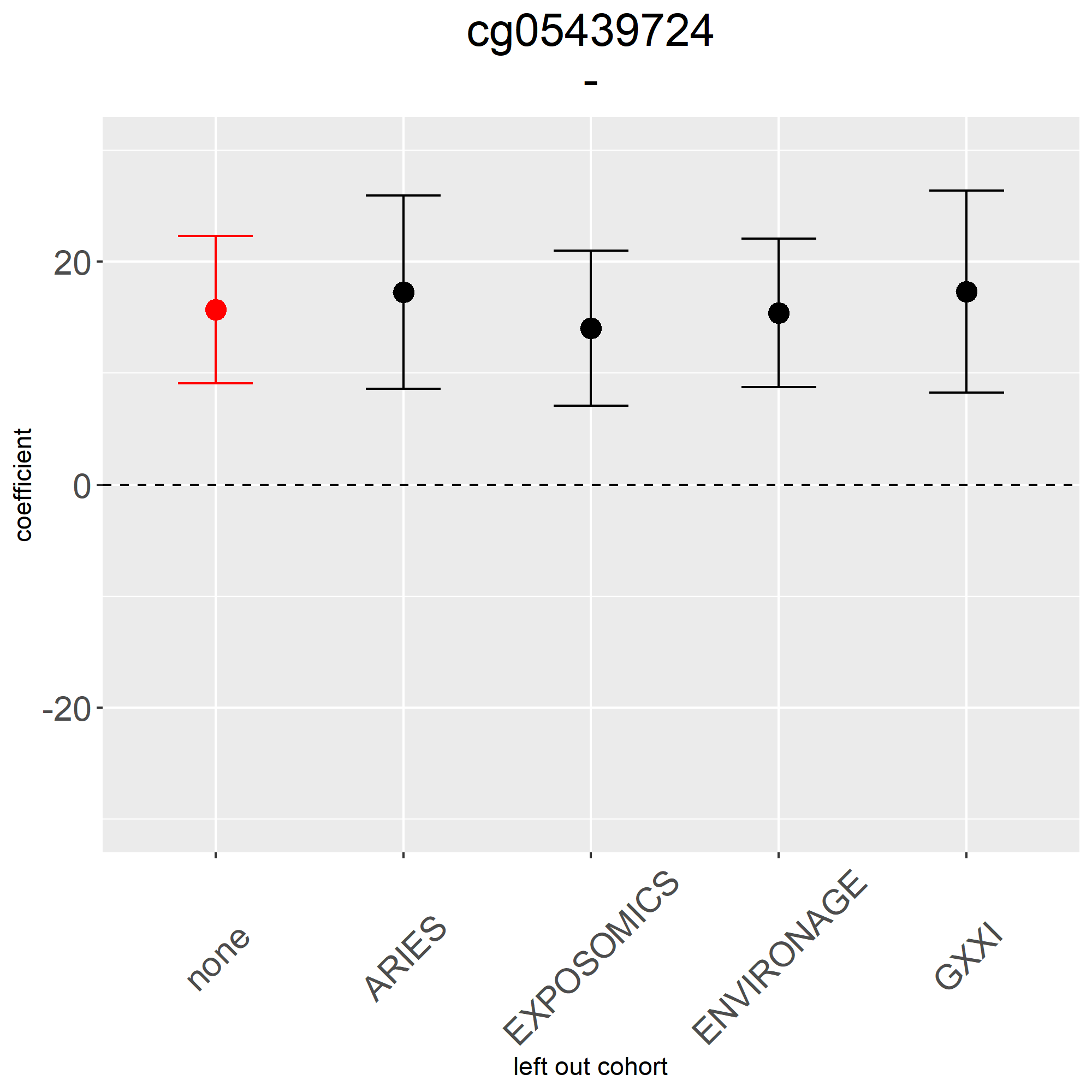

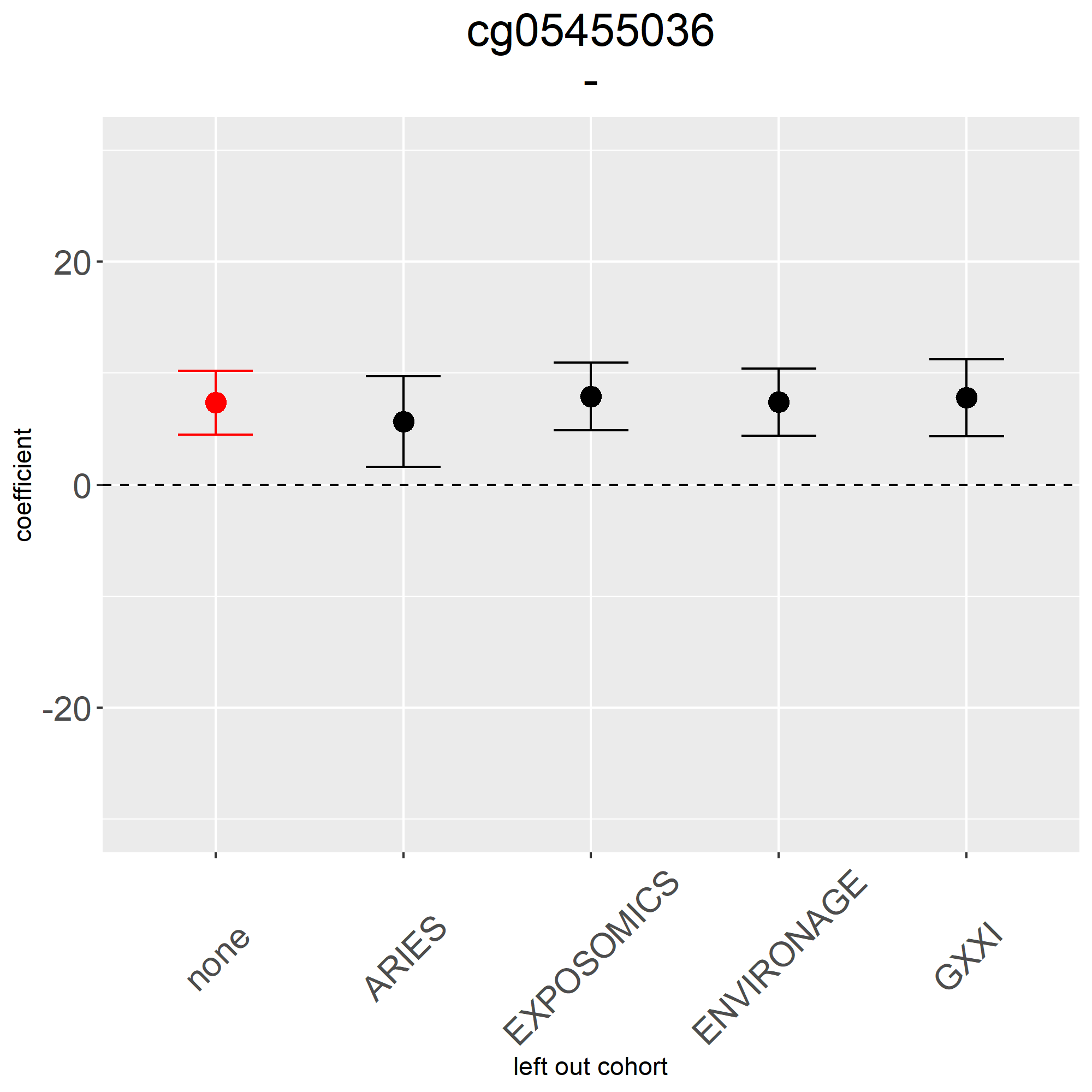

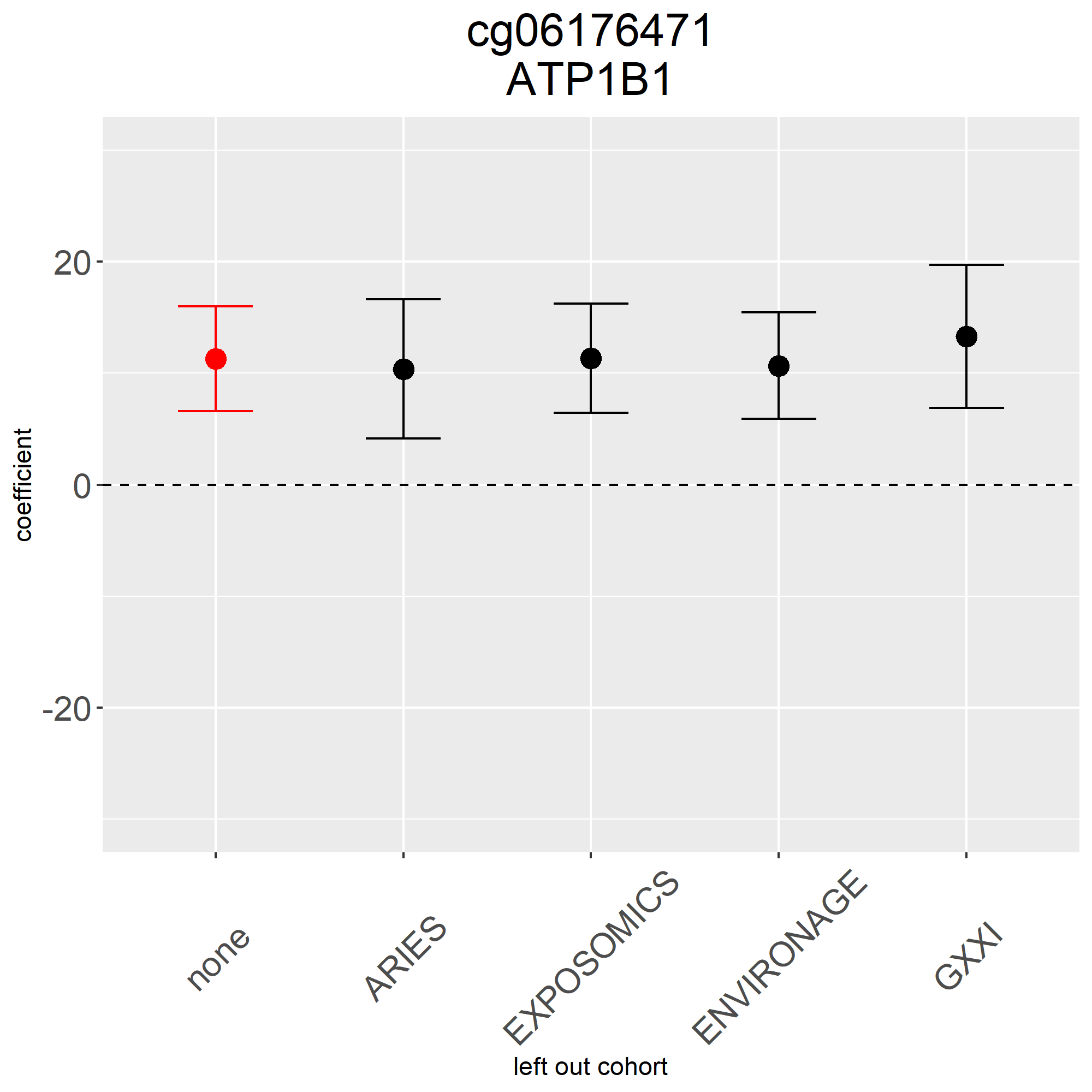

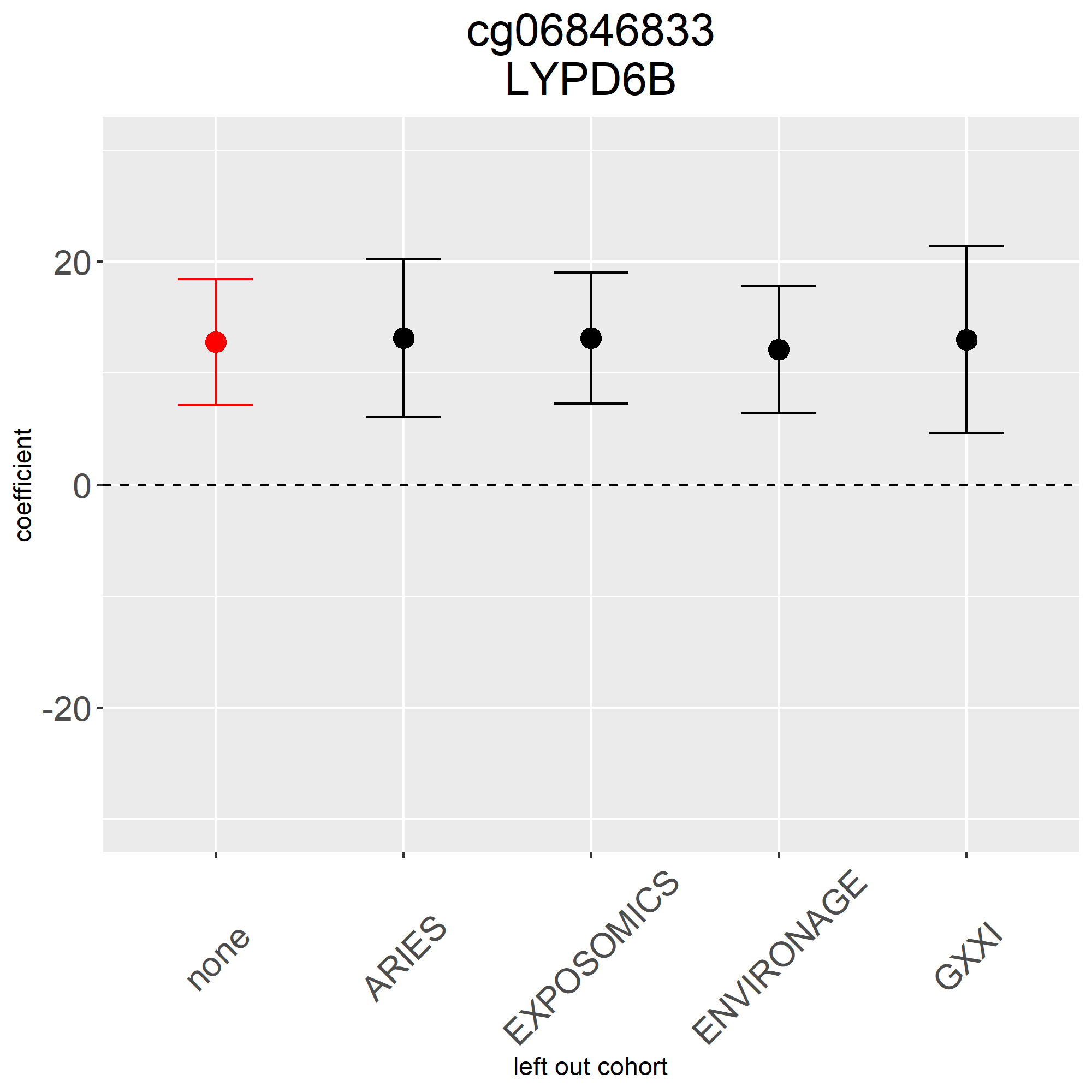

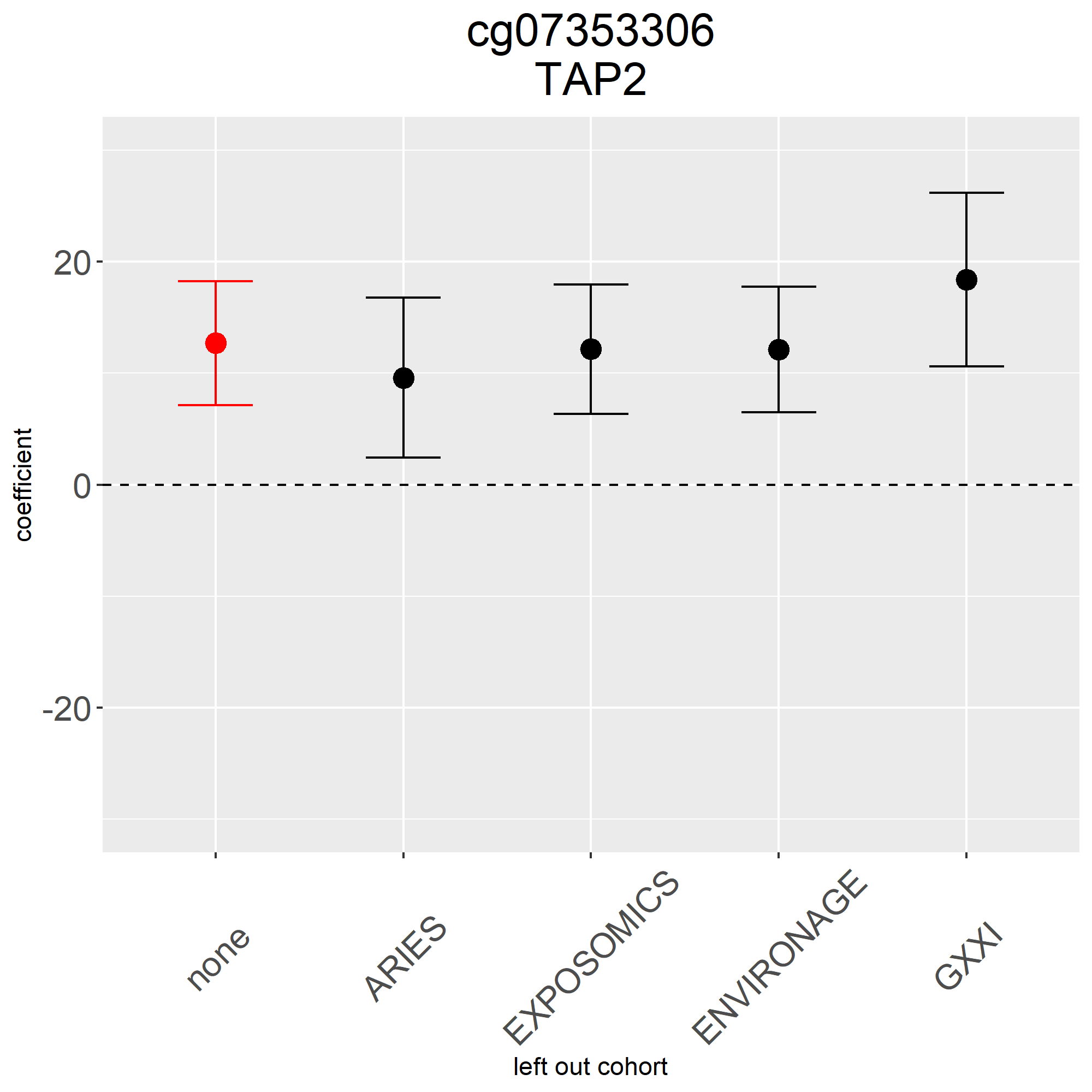

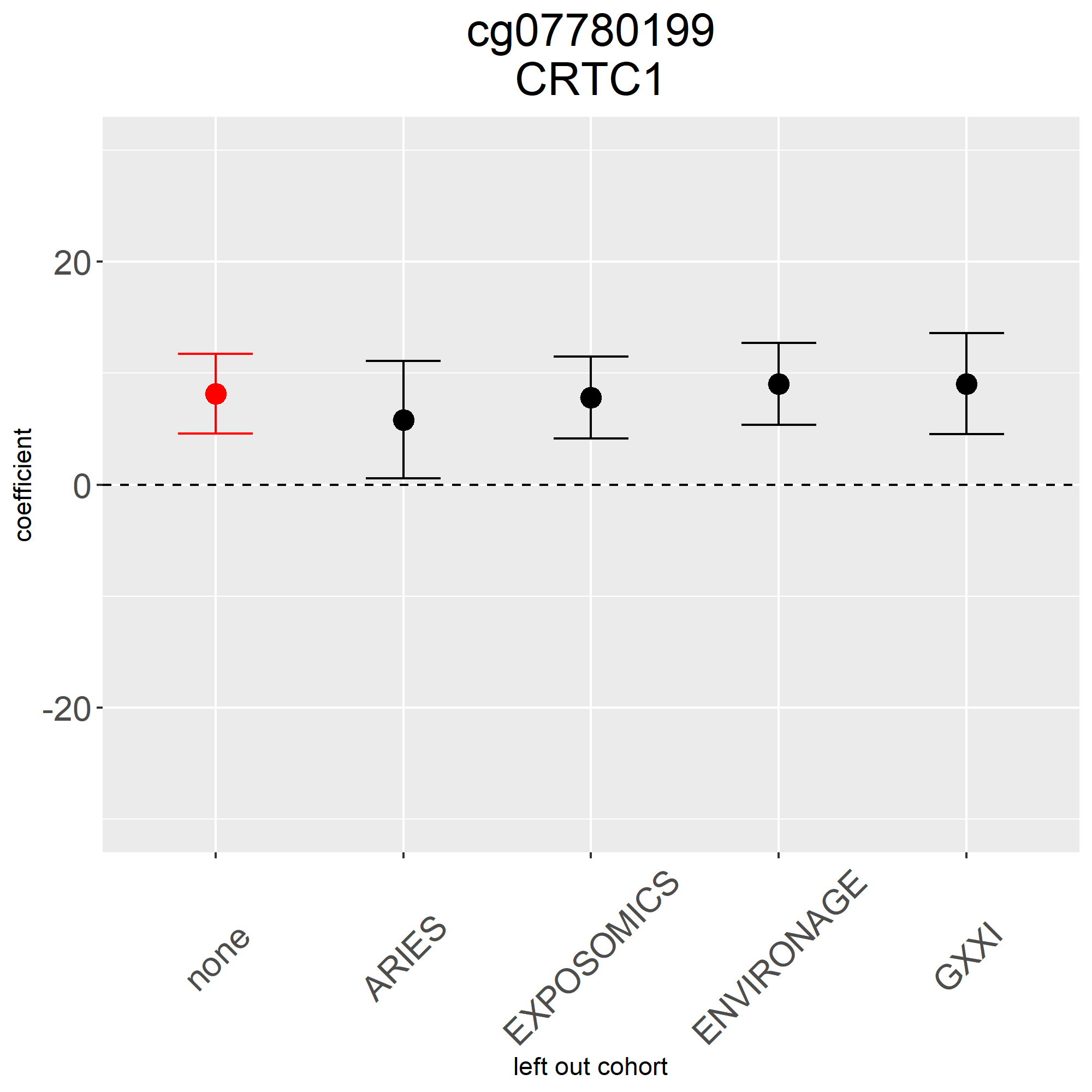

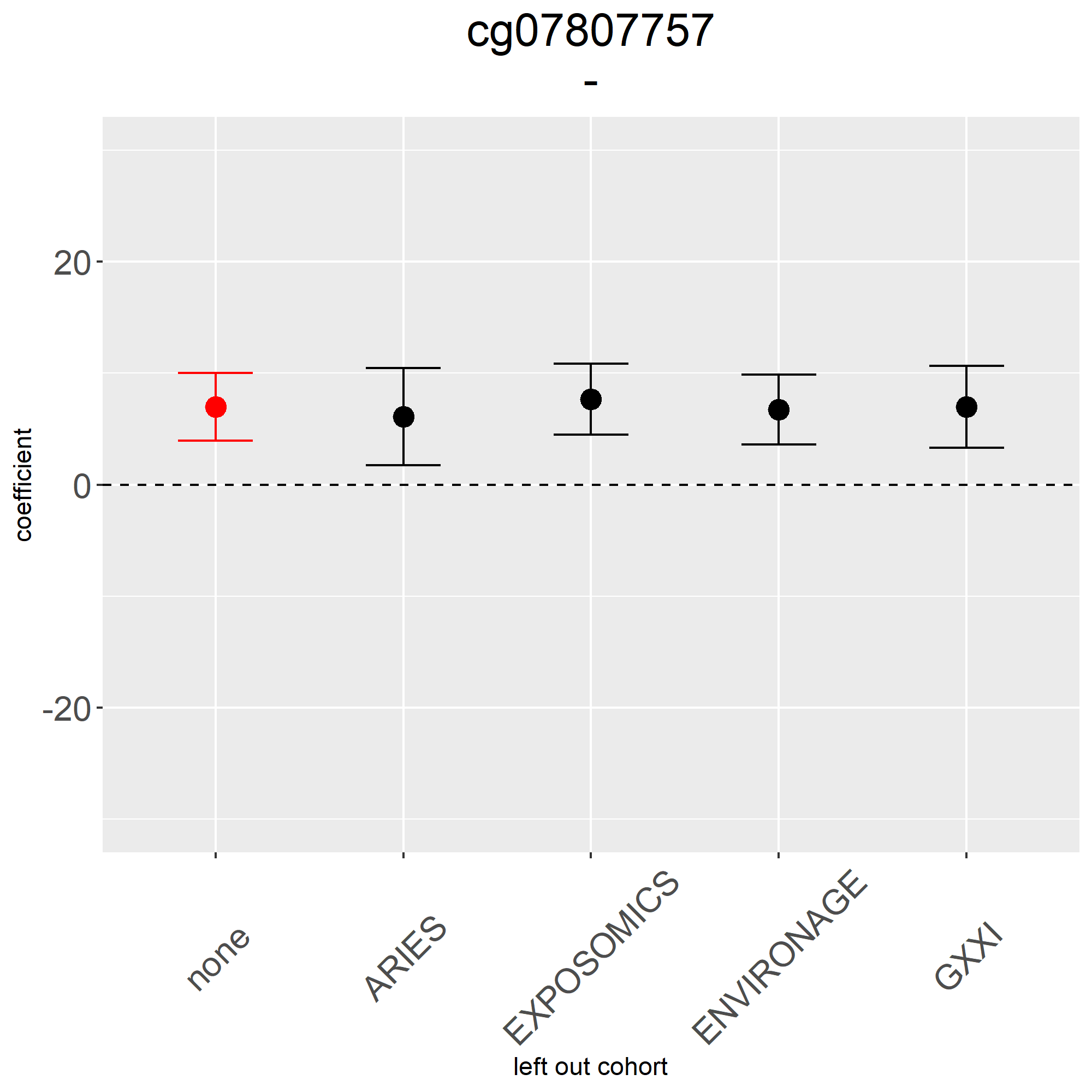

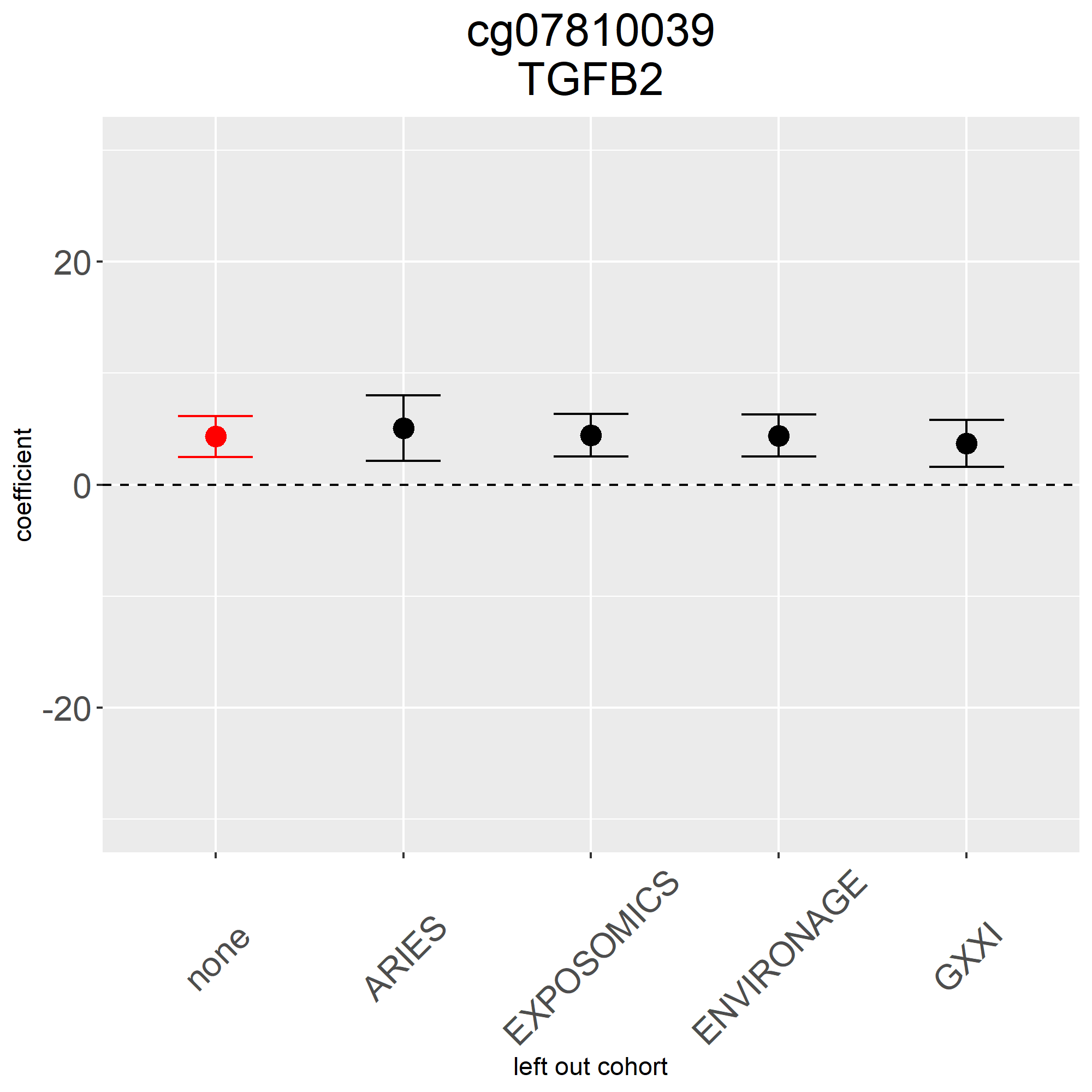

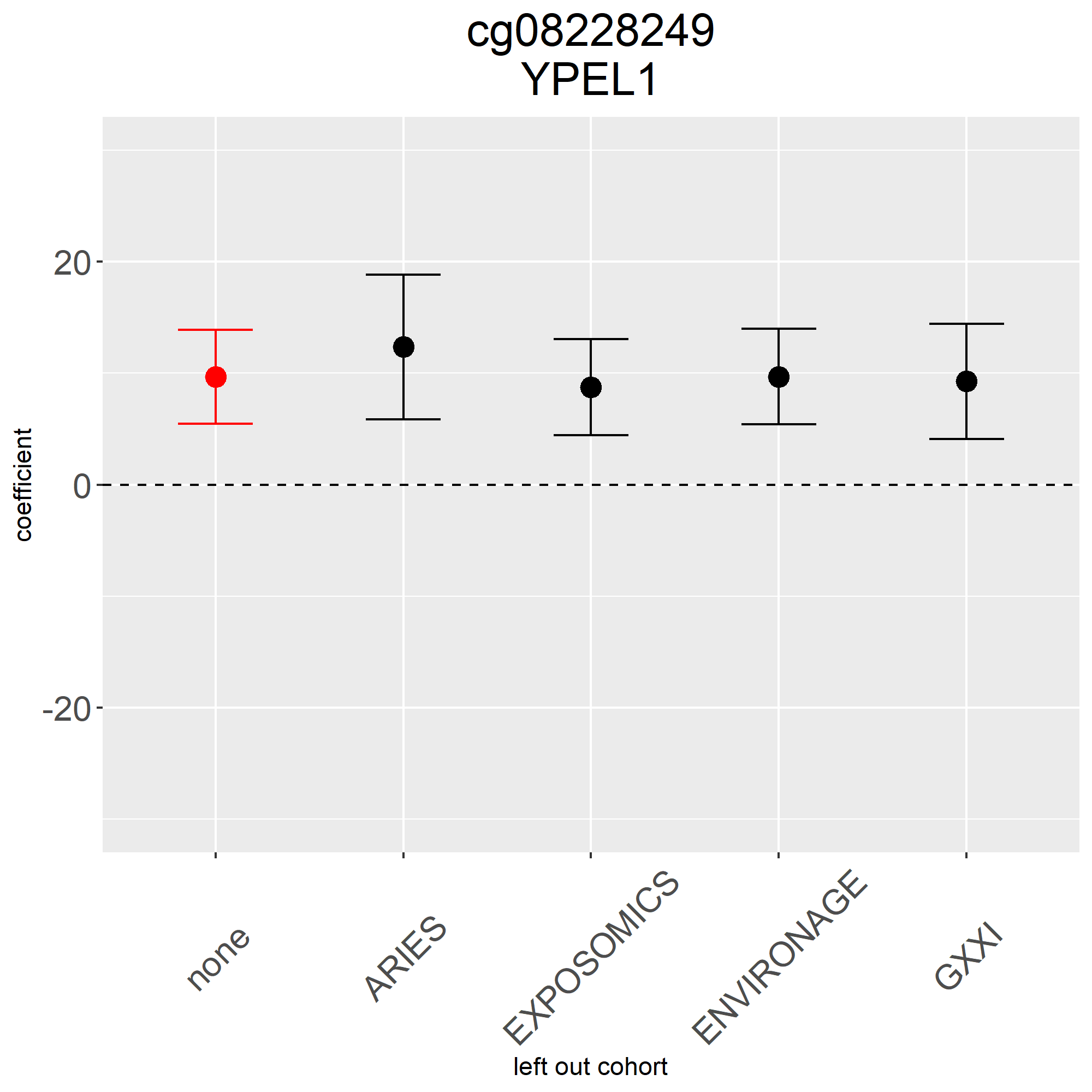

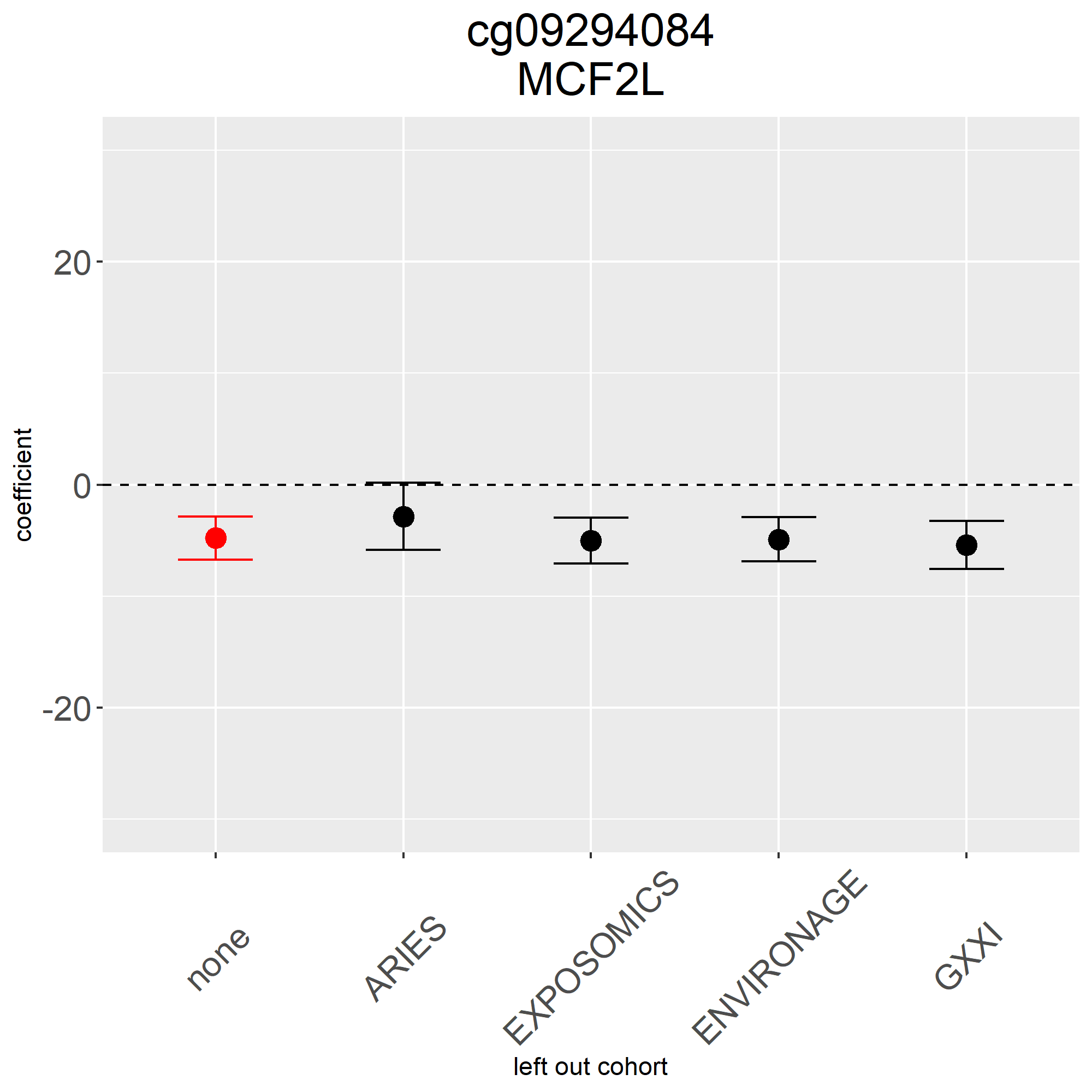

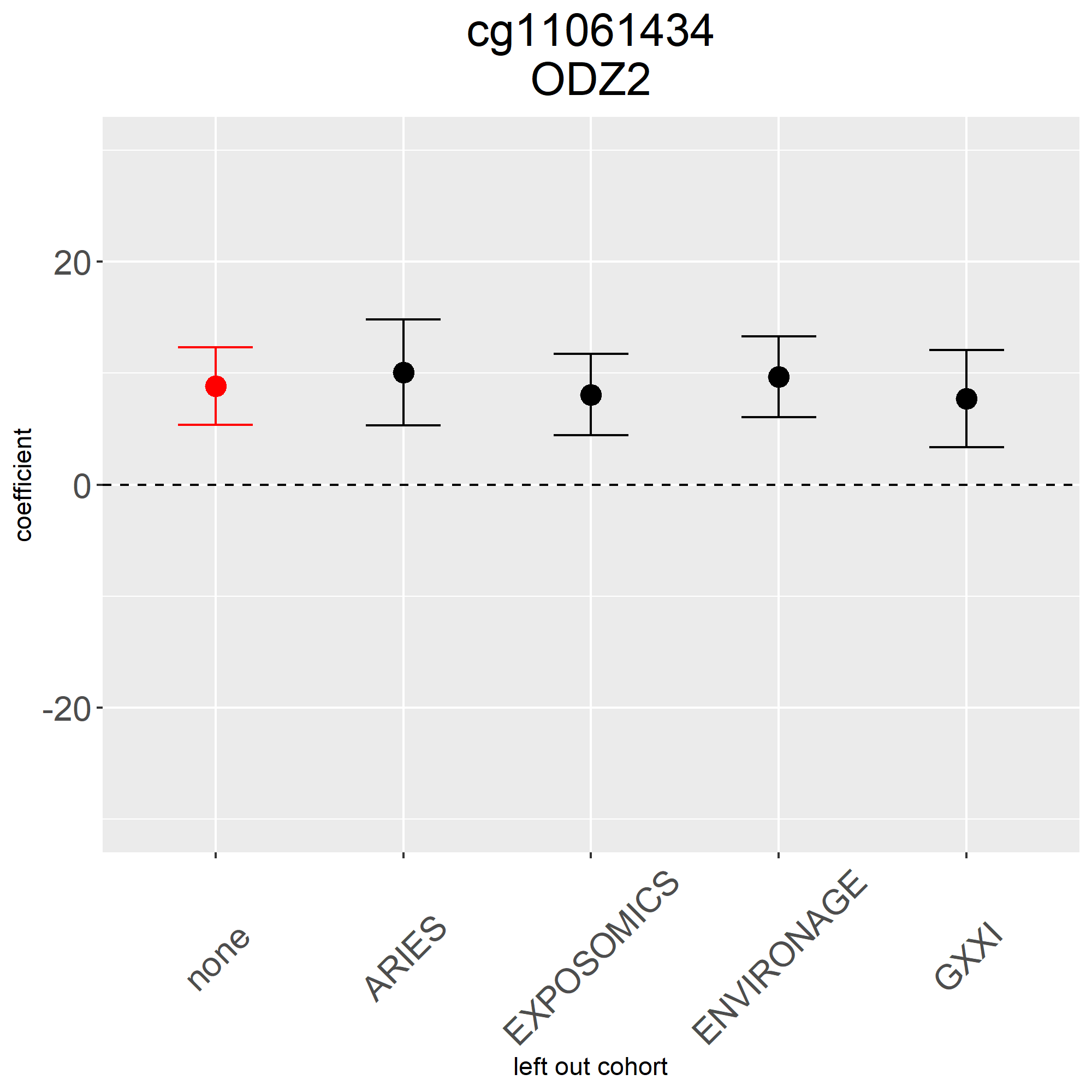


# FigS3. Quality control of cohort specific EWAS of rapid weight growth. Coefficients, standard errors and p-values distributions visualized via box plots and QQ-plot.


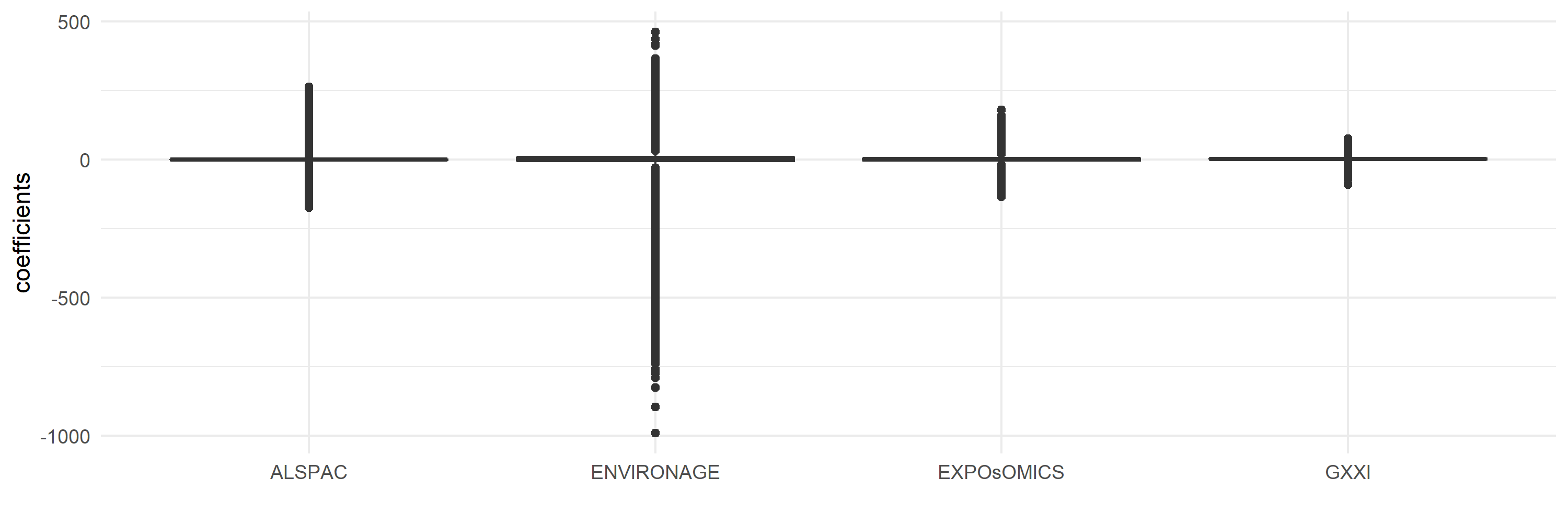


# FigS4. Cohort specific correlation plots between chronological and DNA methylation gestational age.

GA= gestational age

# FigS5. Forrest plot of the meta-analysis of gestational age acceleration and rapid weight growth in sensitivity analyses adding (A) delivery mode, (B) removing cell types from confounders, excluding mothers with (C) gestational diabetes and (D) non-white European children.

95% CI=95% confidence interval

# FigS6. Calibration plots of Random Forest models of rapid weight growth including (A) conventional risk factors, (B) CpGs related to rapid weight growth and (C) both.

# FigS7. Calibration plots of Random Forest models of rapid weight growth including (A) conventional risk factors, (B) CpGs belonging to DMRs related to rapid weight growth and (C) both.

# FigS8. Heatmap shows Pearson’s correlation between methylation levels of the 44 CpGs associated with P_Suggestive_ <1e-05 in the meta-analysis of EWAS of rapid weight growth. Red boxes indicate the three CpGs identified in mediation analyses.

# FigS9. Volcano plots of the association between the 44 CpGs associated with rapid weight growth at P_Suggestive_ < 1e-05/the 96 CpGs belonging to the 16 DMRs associated with rapid weight growth at FDR-adjusted p-value in DMRcate and Siddak p-values in ENmix-comb-p <0.01 and the entire transcriptome (A/C) and restricted to cis transcripts (B/D). Red lines represent Bonferroni-significant threshold, and black lines suggestive threshold (p-value=10-e05). For analyses restricted to cis transcripts only Bonferroni-significant threshold is represented.

DMR= differentially methylated regions; FDR= false discovery rate. Labels represent gene names for gene expression and are coloured according to the associated DNA methylation signals as reported in the legend.

# FigS10. Volcano plots of the association between the 44 CpGs associated with rapid weight growth at P_Suggestive_ < 1e-05/the 96 CpGs belonging to the 16 DMRs associated with rapid weight growth at FDR-adjusted p-value in DMRcate and Siddak p-values in ENmix-comb-p <0.01 and the entire metabolome (A/B). Red lines represent Bonferroni-significant threshold, and black lines suggestive threshold (p-value=10-e05).

# FigS11. Forrest plot of the meta-analysis of the analysis of gestational age acceleration and childhood overweight.

95% CI=95% confidence interval

# FigS12. Volcano plot from the look-up in the study population of the CpG sites associated with child anthropometrics in a previous systematic review by Alfano et al. [60]

Alfano R, Robinson O, Handakas E, Nawrot TS, Vineis P, Plusquin M. Perspectives and challenges of epigenetic determinants of childhood obesity: A systematic review. Obes Rev. 2021;23 Suppl 1:e13389.
